# Human adipose-derived mesenchymal stem cells for acute and sub-acute TBI

**DOI:** 10.1371/journal.pone.0233263

**Published:** 2020-05-26

**Authors:** Katherine A. Ruppert, Karthik S. Prabhakara, Naama E. Toledano-Furman, Sanjna Udtha, Austin Q. Arceneaux, Hyeonggeun Park, An Dao, Charles S. Cox, Scott D. Olson

**Affiliations:** 1 Department of Pediatric Surgery, McGovern Medical School at The University of Texas Health Science Center at Houston, Houston, TX, United States of America; 2 Hope Biosciences, Sugarland, TX, United States of America; Fu Jen Catholic University, TAIWAN

## Abstract

In the U.S., approximately 1.7 million people suffer traumatic brain injury each year, with many enduring long-term consequences and significant medical and rehabilitation costs. The primary injury causes physical damage to neurons, glia, fiber tracts and microvasculature, which is then followed by secondary injury, consisting of pathophysiological mechanisms including an immune response, inflammation, edema, excitotoxicity, oxidative damage, and cell death. Most attempts at intervention focus on protection, repair or regeneration, with regenerative medicine becoming an intensively studied area over the past decade. The use of stem cells has been studied in many disease and injury models, using stem cells from a variety of sources and applications. In this study, human adipose-derived mesenchymal stromal cells (MSCs) were administered at early (3 days) and delayed (14 days) time points after controlled cortical impact (CCI) injury in rats. Animals were routinely assessed for neurological and vestibulomotor deficits, and at 32 days post-injury, brain tissue was processed by flow cytometry and immunohistochemistry to analyze neuroinflammation. Treatment with HB-adMSC at either 3d or 14d after injury resulted in significant improvements in neurocognitive outcome and a change in neuroinflammation one month after injury.

## Introduction

Traumatic brain injury (TBI) is a major cause of disability in the United States, causing immediate and long-lasting effects [1]. Providing care and rehabilitation for TBI patients is a significant economic burden, estimated to be approximately $76.5 billion for both direct and indirect costs [2]. Cognitive impairments, motor and sensory dysfunction, and emotional changes are among the most common issues affecting TBI patients and their families and communities. Although the physical, emotional and economic burdens are large and can be long-lasting, there has yet to be an effective therapy to mitigate some of these issues.

Following the primary mechanical insult of TBI, secondary injuries have been shown to increase neurological damage and perpetuate the chronicity of TBI [3]. Increased blood-brain barrier (BBB) permeability [4], neuroinflammation [5], edema, neurodegeneration, oxidative stress and innate immune response are hallmarks of secondary injury, with onset as early as minutes after impact and some effects lasting for several years, supporting the idea that TBI develops as a chronic illness [6]. Moments after impact, an intense inflammatory response develops and BBB integrity is compromised, allowing peripheral leukocytes to infiltrate the brain parenchyma. Injured cells release damage-associated molecular pattern molecules (DAMPs) [7], which are recognized by infiltrating immune cells and resident microglia, astrocytes and neurons, stimulating the release of pro-inflammatory cytokines, anti-inflammatory cytokines, reactive oxygen and nitrogen species. The complex cascade of events following the primary injury is referred to as secondary injury. Modulation of this signaling process would provide a therapeutic target to interrupt or mitigate the exacerbation of secondary injury, ultimately sparing neighboring cells and lessening the likelihood of poor outcome.

Microglia activation following TBI is associated with neuroinflammation. Activated microglia are capable of exhibiting pro- and anti-inflammatory phenotypes, based upon polarization states. M1 phenotype microglia release pro-inflammatory cytokines and oxidative mediators, while M2 phenotype microglia release anti-inflammatory cytokines and neurotrophic factors [8]. Activation and polarization of microglia is transient, varying at many points after injury. The classical paradigm of M1 and M2 phenotypes may not accurately characterize microglial activation, which has been suggested in more recent publications [9]. Understanding temporal patterns in activation and polarization of microglia following TBI may provide information that allows more specific targets for modulation of neuroinflammation.

The interest in cell therapy as a potential therapeutic intervention for TBI has gained momentum over the past several years. Mesenchymal stem cells (MSC) are multipotent, fibroblast-like cells that can be isolated from various tissues, including bone, adipose, muscle, teeth, pancreas, lung, liver, amniotic fluid, cord blood and umbilical cord tissues [10–13]. MSCs are appealing for therapeutic intervention because they are easily isolated from virtually any adult tissues[10], have been shown to be safe and non-tumorigenic[14, 15] and exhibit potent immunomodulatory properties [16–18]. Administration of MSCs has been studied in many experimental models and clinical trials including, TBI [5, 19–25], stroke [26, 27], and spinal cord injury [28–35]. In regenerative medicine, some stem cell therapies aim to replace damaged cells, however the primary benefit of MSCs is derived from paracrine immunomodulation and alteration of the injury environment. Our group, as well as others, has demonstrated that the majority of MSCs do not cross the BBB to the injury site but rather, they perform immunomodulation from the periphery [36–40]. The release of various trophic factors contributes to attenuation of neuroinflammation, promoting angiogenesis, neurogenesis, and reducing apoptosis. Proinflammatory signals such as lipopolysaccharide (LPS), tumor necrosis factor-α (TNF-α), and nitric oxide (NO), stimulate MSCs to secrete anti-inflammatory factors [41]. Of the anti-inflammatory factors, prostaglandin E2 (PGE2) has been shown to be constitutively expressed by MSCs via the COX-2 pathway and acts as a potent immunomodulatory factor [18]. MSCs derived from bone marrow, umbilical cord and adipose tissue are most commonly studied, with some differences noted between tissues of origin [42]. In this study, we explored the potential for early and delayed administration of adipose-derived MSCs (adMSCs) as a treatment for TBI.

Adipose-derived stem cells, also referred to as adipose-derived mesenchymal stem cells, are appealing for therapeutic use because they are relatively easy to harvest and they are available in abundance. AdMSCs are harvested from liposuction waste tissue and undergo collagenase digestion and differential centrifugation to produce a stromal vascular fraction containing adipocyte progenitor cells [43, 44]. These cells differentiate into adipocytes, osteoblasts, myoblasts, chondroblasts and neural cells, and maintain their identifying characteristics through several cell passages (manuscript in review). AdMSCs have been used in animal models for hemorrhagic stroke [45], spinal cord injury [46] and cerebral ischemia [47], and have demonstrated decreased inflammation, decreased neurodegeneration, improvement in motor function and decreased immune response.

In this study, we investigated the effects of early and delayed initiation of treatment following TBI. Examination of delayed initiation of treatment following TBI is novel and can provide a clinically relevant timeline for potential autologous cell therapy. We used a controlled cortical impact (CCI) model to simulate moderate to severe TBI in adult rats, which were treated with human adMSCs at either 3 days or 14 days post-injury. Animals were routinely assessed for neurological and vestibulomotor function and, upon sacrifice at 32 days post-injury, brain tissues were analyzed for neuroinflammation via flow cytometry and immunohistochemistry.

## Materials and methods

### Animals

All protocols involving the use of animals were following the National Institutes of Health Guide for the Care and Use of Laboratory Animals. All work performed was approved by the University of Texas Health Sciences Center at Houston Animal Welfare Committee (HSC-AWC 16–0046) following the NIH Guide for the Care and Use of Laboratory Animals. The study utilized inhaled isoflurane for short-term anesthesia during surgical procedures followed by a lethal dose as a primary method of euthanasia followed by exsanguination and decapitation. Rats were purchased from Envigo (Indianapolis, IN, USA) for use in this study and housed on a 12h light/dark cycle with *ad libitum* access to food and water.

### Controlled cortical impact animal model

Moderate/severe cortical contusion injury (CCI) was performed in this study using an electromagnetically driven controlled cortical impact device (Pittsburgh Precision Instrument, Inc.), as previously described [48]. Briefly, male Sprague Dawley rats (Envigo Harlan, USA) weighing 250-275g were anesthetized with 4% isoflurane in oxygen and maintained using 1.5 L/min of 2–3% isoflurane throughout the procedure. The head of the rat was then immobilized on a stereotactic frame (Pittsburgh Precision Instrument, Inc.) and a 6–7 mm diameter craniectomy was performed on the right cranial vault. The center of the craniectomy was placed at the midpoint between bregma and lambda, 3 mm lateral to the midline, overlying the temporoparietal cortex. Animals received a single impact of 3.1 mm depth of deformation with an impact velocity of 5.6 m/s and a dwell time of 150ms using a 6mm diameter flat impactor tip to the parietal association cortex orthogonal to the surface at a 20° angle from the vertical plane.

### Isolation and expansion of HB-adMSCs

HB-adMSCs were derived from one single donor. Fat extraction (~ 10cc) was performed by a licensed plastic surgeon via mini liposuction from the abdominal adipose tissues. Fat was centrifuged at 3,000 rpm for 5 mins, which then separated into three layers: oil (top), tumescent (middle) and blood (bottom) layer. The middle layer was collected and mixed with collagenase type 1 solution (HB-102, Hope Biosciences, Sugar Land, TX, 1mg/ml), 1 ml of fat for every 4 ml of collagenase, which was incubated at 37°C with gentle shaking (150 ± 30 rpm) for 1–2 hours. Then the mixture was centrifuged at 1,800 rpm for 5 mins. There were two layers after centrifugation, the bottom layer was collected and re-suspended with 10 ml of HB-103 isolation media (Hope Biosciences, Sugar Land, TX). The solution was then filtered with a 100 μm cell strainer to remove debris and subsequently centrifuged at 1,700 rpm for 5 mins. The cell pellet, aka stromal vascular fraction (SVF), was collected and seeded in HB-103 at 37°C, 5% CO_2_ for 16–32 hrs. Cell adhesion was checked using the inverted microscope and the flask was washed with PBS/DPBS to remove non-adherent cells. The growth media, Hope Biosciences’ proprietary HB-101, was added to the flask to achieve the first expansion passage (P0) and incubated at 37°C, 5% CO_2_ until reaching 95% confluence. Media was changed every 2 days. Once cells reached 95% confluence, the P0 cells were sub-cultured and expanded in HB-101 until passage 4. P4 cells were harvested in PBS solution and transported to UT on the same day, which was also the injection day, at room temperature. HB-adMSC have characteristic traits consistent with conventional MSC, including a phenotype negative for CD31, CD34, CD45, and HLA-DR and positive for CD29, CD44, CD73, and CD90 (S1 Table).

### Treatment with human adipose-derived mesenchymal stem cells

**Fig 1:**
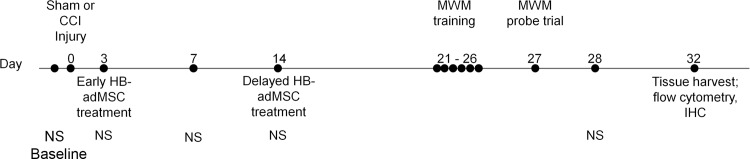
Experimental timeline. Animals are tested for baseline behavior measurements prior to injury at Day 0. HB-adMSCs treatment occurs either at Day 3 or Day 14, depending on group. Behavior testing continues on Day 3, 7, 14, 21–26, 27, and 28. Animals are sacrificed and tissues are harvested on Day 32. Neuroscore (NS), Hope Biosciences’ adipose derived mesenchymal stem cells (HB-adMSCs), Morris water maze (MWM), immunohistochemistry (IHC).

Human adipose-derived mesenchymal stem cells (HB-adMSCs; from Hope Biosciences) treatments were delivered at 3 days (early) and 14 days (delayed) post-CCI to determine the effects on neurocognitive function, neuroinflammation and neurogenesis at 32 days post-CCI. Treatment consists of a single administration of 3X10^6^ cells/kg via tail vein injection at either 3 days post-CCI or 14 days post-CCI (Fig 1).

### Morris water maze

We assessed cognitive function at 21 days post injury to assess spatial memory and spatial learning in rats as previously described [49]. Standard protocol was followed where a blinded investigator tested all the groups in a hidden platform MWM set-up by performing 4 trials per day with 4 minutes between each trial for 6 days for each rat. The latency to platform was measured as the time to find the platform (Adanac 3000 Digital, Marathon Watch Company Ltd., Ontario, Canada). Probe Trials (removal of platform) were done on day 7, 24 hours after the conclusion of platform testing. The EthoVision® XT 8.0 tool from Noldus Information Technology was used to record and analyze the probe trials.

### Flow cytometry

Microglial isolation and analysis was performed as previously published [50]. In brief, microglia were isolated by first extracting the the brain and then mechanical and enzymatic digestion was used to obtain a single-cell suspension using a Neural Cell Dissociation kit (Miltenyi Biotec). We utilized density centrifugation to remove a large amount of myelin, followed by microglial enrichment using CD11b/c microbeads in magnet activated sorting (MACS) kit (Miltenyi Biotec). The resulting CD11-enriched cells were then stained for CD45, CD11, P2Y12, CD32, CD86, CD200R, RT1B, CD163, and a Ghost™ viability dye (TONBO) with the addition of Cyto-Cal™counting beads (Thermo Fisher Scientific). This optimized multicolor immunofluorescence panel (OMIP) was designed to phenotype rat-derived microglia from the CNS, and allows for differentiation between macrophages and microglia, as well as phenotypic changes in microglia. Time points were selected as comparable to our previous work in MSCs to treat TBI.

### Immunohistochemistry

Rats were anesthetized and brain was harvested following perfusion with PBS and 4% PFA 32 days after the injury. Coronal sections at 30 μm were cut in a vibrating blade microtome (Leica VT1000 S) and stained in suspension using standard staining protocol. Briefly, the sections were first rinsed with PBS with 0.01% Triton X-100 (PBST; T-9284, Sigma Aldrich) twice for a minute each followed by permeabilizing step for 20 minutes with PBS with 0.2% Triton X-100. Then they were blocked for 30 minutes in PBST containing 3% goat serum (005-000-121, Jackson Immuno Research, PA). The primary and secondary antibodies were both prepared in PBST with 2% Bovine Serum Albumin (A2153, Sigma Aldrich) and 1% goat serum incubated for overnight at 4°C and 2 hours at room temperature, respectively. The sections were washed thrice with PBST for 10 minutes each prior to and after incubation with secondary antibodies. Lastly, the sections were mounted onto slides and let dry at room temperature before they were cover-slipped with DAPI Fluoromount-G (0100, Southern Biotech, Birmingham, AL). Primary antibodies: regenerating neurons (Doublecortin, 1:1000, AB2253, Temecula, CA), mature neurons (NeuN, 1:500, Millipore Sigma, Billerica, MA), astrocytes (GFAP, 1:500, Millipore Sigma, Billerica, MA), activated microglia (IBA-1, 1:500, Wako-chem, Richmond, VA). Secondary antibodies: 1:1000, Alexa Fluor 488/green: A11073, 1:1000, Alexa Fluor 568/red: A11011, Invitrogen.

### Neurogenesis

Neurogenesis was determined using the same brain slices used for microglia/macrophage characterization, but assessing the number of doublecortin positive cells (DCX+) in the subgranular zone of the dentate gyrus [51]. Cell quantification was performed using fluorescent microscopy with a Leica BDX5100 20x objective. Two slices per brain, at least 30 μm apart, were analyzed for the total number of DCX+ cells present in the subgranular zone of the dentate gyrus. The number of DCX+ cells were normalized by the total length of the subgranular zone of the dentate gyrus on each brain slice analyzed.

### Brief neurological assessment

Each day, starting 2 days prior to injury, neurological outcomes were assessed using a neuroscore system [52]. The short functional neuroscore consists of five tests: (1) forelimb flexion test, (2) hind limb flexion test, (3) visually triggered placing test, (4) contact triggered placing test, and (5) hind paw grasping reflex test. Forelimb flexion was tested by lifting the rat by the tail and holding approximately 12 inches above the table surface, observing for flexion or extension of forelimbs. Flexion is abnormal and receives a score of 1. No flexion has a score of 0. The hind limb flexion test is done the same as the flexion test, scoring 1 for hind limb flexion or 0 for no flexion. Visual triggered placing tests were performed by lifting the rat by the tail and slowly lowering toward the table edge, up to approximately 10 cm from nose to table edge. Moving the rat towards the edge, observation of the presence or absence of extending forepaws was scored. Extension is a normal behavior with a score of 0, no extension has a score of 1. Contact triggered placing tests were performed by holding the rat with body in hand, parallel to table edge, with forelegs free. Slowly the rat was lowered to the table until the whiskers on one side touch the edge of the table. Forelimb extension on the same side as the touching whiskers is normal, scoring 0, while the absence of extension in response to tactile stimulation is abnormal and given a score of 1. The test was repeated for opposite side. Hindpaw grasping reflex was tested by holding the rat in hand, thumb and index finger around the chest, under the forelimbs. Gently touching the palm of each hind paw with right forefinger and observing whether the rat grasps the finger. The presence of grasp is normal and receives a score of 0, no grasp has a score of 1. Each test was performed on both left and right sides. All scores were tallied for a possible total of 21. A score of 0 indicated normal reflex function.

### Beam balance

The balance beam apparatus consists of a beam 22.5” L x 1.5” H x .75” W and a barrier 10.5” H x 13” W. The beam was secured to a table and the barrier was attached to the beam so that 10.5” of the beam protrudes from the barrier away from the table over a padded safety box. Animals underwent two training sessions and one pre-assessment prior to injury (day 0) beginning on day –2. On day –2, the animal was placed on the balance beam for 60 second trials. Then the animal was removed from the beam for a 15 second resting period between each trial in order to disorient him from the beam. If the animal could not balance, it was allowed to fall from the beam into a padded box. Animals were trained until able to remain on the beam for three consecutive 60-second trials. Trials were scored numerically, 1–6. Each trial was scored as follows: (1) Balances with steady posture (grooms, climbs barrier), (2) Grasps sides of beam and/or has shaky movements, (3) Hugs the beam or slips or spins on beam, (4) Attempts to balance, but falls off after ten seconds, (5) Drapes over beam or hangs from beam and falls off in less than ten seconds, and (6) Falls off, makes no attempt to balance or hang from beam. On the day of the injury (day 0), the animal underwent a pre-assessment consisting of three trials. The animals were assessed on various days post injury, 3, 14 and 28 days–three trials each day [53–56].

### Beam walking

The beam walk apparatus consists of a beam that is measured 40” L x 1” W. One end of the beam is stabilized by a stand (starting end) and the opposite end is attached to the goal box that is on a table. The goal box is a black box with a hinged lid, for accessing the animal. The box is 11” L x 7.25” H x 7.25” W. The beam leads to a doorway (4.25” square) in the goal box. Four pegs (0.75” H) are inserted at 9.5”, 18.5”, 28.5”, and 38.25” from the starting end. Peg placement alternates along the outer edges of the beam beginning on the right edge. A light source and white noise source are positioned on a cart at the starting end of the beam walk. Training on the beam walk begins on day –2. The animal was placed in the goal box for two minutes at the start of the training session and the pegs are removed from the beam. At the end of two minutes, the handler turned on the white noise and light and removed the animal from the goal box via the hinged lid. The animal was placed on the beam at the location of the peg closest to the goal box and allowed to walk to the goal box. As soon as the animal’s front feet crossed the threshold of the goal box, the light and noise were turned off. The animal was allowed to rest in the goal box for 30 seconds between each run. The animal was not allowed to fall from the beam; the handler assisted him if he started to fall off. This procedure was repeated twice at each peg location and from the starting position. After two training runs from each position, the pegs were inserted and one complete beam walk was done for practice. Three timed beam walk trials were then recorded to conclude training. The timer started once the animal was securely positioned at the starting point and the first step was taken in the direction of the goal box. On the day of the injury (day 0), the animal underwent a pre-assessment consisting of three timed trials. The animals were assessed on various days post-injury days 1, 2 and 3 with three trials each day [53, 54, 56].

### In vitro immunoactivity assays

The ability of HB-adMSC to change immune activity was characterized using a set of previously published *in vitro* assays [18, 41, 48]. In brief, a dilution of HB-adMSC was co-cultured with either lipopolysaccharide (LPS) (Sigma) or concanavalin A (conA) (Sigma) and incubated for 24 or 48 hrs with the resulting supernatant analyzed for TNF-α or IFN-γ by ELISA (BD), respectively. Additionally, the LPS-stimulated co-culture’s media was analyzed for PGE2 by ELISA (Cayman). Separately, MSC were stimulated with either TNF-α (50 ng/ml) or IFN-γ (50 ng/ml) and quantitative reverse-transcriptase PCR was used to evaluate expression of COX-2 (PTGS2), IDO1, TSG-6, and IL-1ra using commercial Taqman probes (Applied Biosystems).

### Statistical analysis

All data are represented as mean ± SEM. Comparisons between means of each group were made with the use of Unpaired t test, One-way ordinary ANOVA followed by Tukey’s post hoc test, and Two-way ANOVA. p < 0.05 was considered significant.

## Results

### Treatment strategy

We selected two different treatment strategies for HB-adMSC infusion to approximate two common cell therapy approaches. HB-adMSC were infused 3d post-injury to target sub-acute secondary injury mechanisms similar to our previous studies [48, 57], and consistent with the application of a previously manufactured allogeneic cell product. Alternatively, a separate group was treated with HB-adMSC 14d post-injury to approximate the time required to isolate, expand, characterize, and deliver an autologous cell product in the absence of previously banked cells.

### Spatial learning and memory

**Fig 2:**
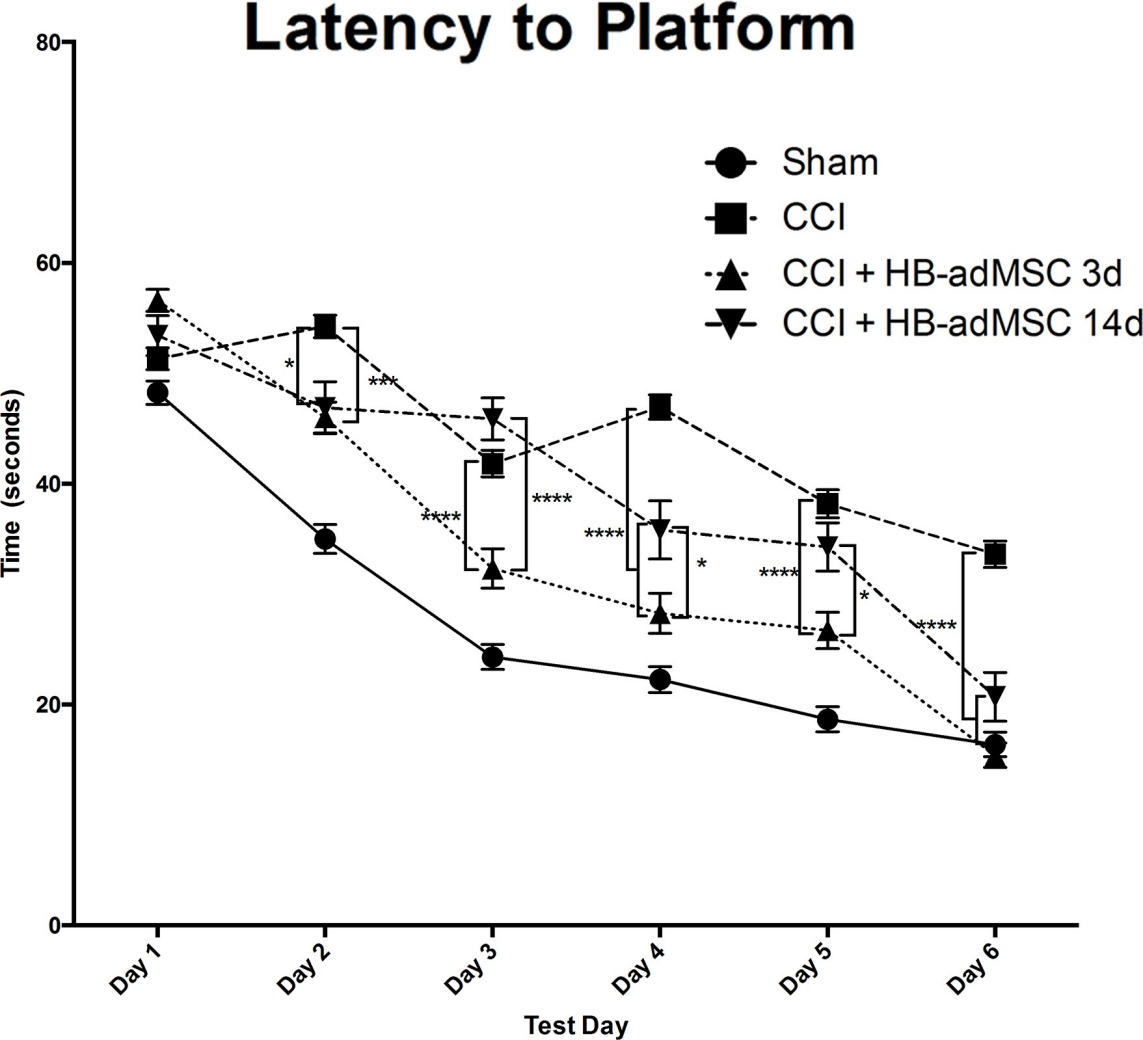
Spatial learning and memory. Animals treated at 3d have significantly shorter latencies compared to CCI on days 2–6. Animals treated at 14d have significantly shorter latencies compared to CCI on days 2, 4, 6. On days 3,4,5 3d treated animals are significantly shorter latency than those treated at 14d. Sham, n = 10, CCI + PBS, n = 13, CCI + HB-adMSCs 3d, n = 7, CCI + HB-adMSCs 14d, n = 3.

The latency to platform was measured each day, for 6 days, beginning day 21 post-CCI (Fig 2). Animals are tested in two consecutive trials each day; the first trial is to learn the location of the platform (location) and the second trial is to remember the location (match). The amount of time required for the animal to locate the platform on the second trial is indicative of ability to remember location based upon the spatial cues surrounding the maze. Animals treated with HB-adMSCs at 3 days post-CCI displayed significantly shorter latencies on test days 2, 3, 4, 5 and 6, when compared to injured controls. Delayed treatment resulted in significantly shorter latencies on test days 2 and 6. There was also significant difference between treatment groups on test days 3, 4 and 5. At the end of the test period, both treatment groups exhibited significantly shorter latencies (****, p<0.0001) when compared to injured controls, although there was not a statistical difference between treatments. Data represents means ± SEM and statistical analysis by Two-Way ANOVA. Sham, n = 10, CCI + PBS, n = 13, CCI + HB-adMSCs 3d, n = 7, CCI + HB-adMSCs 14d, n = 3.

**Fig 3:**
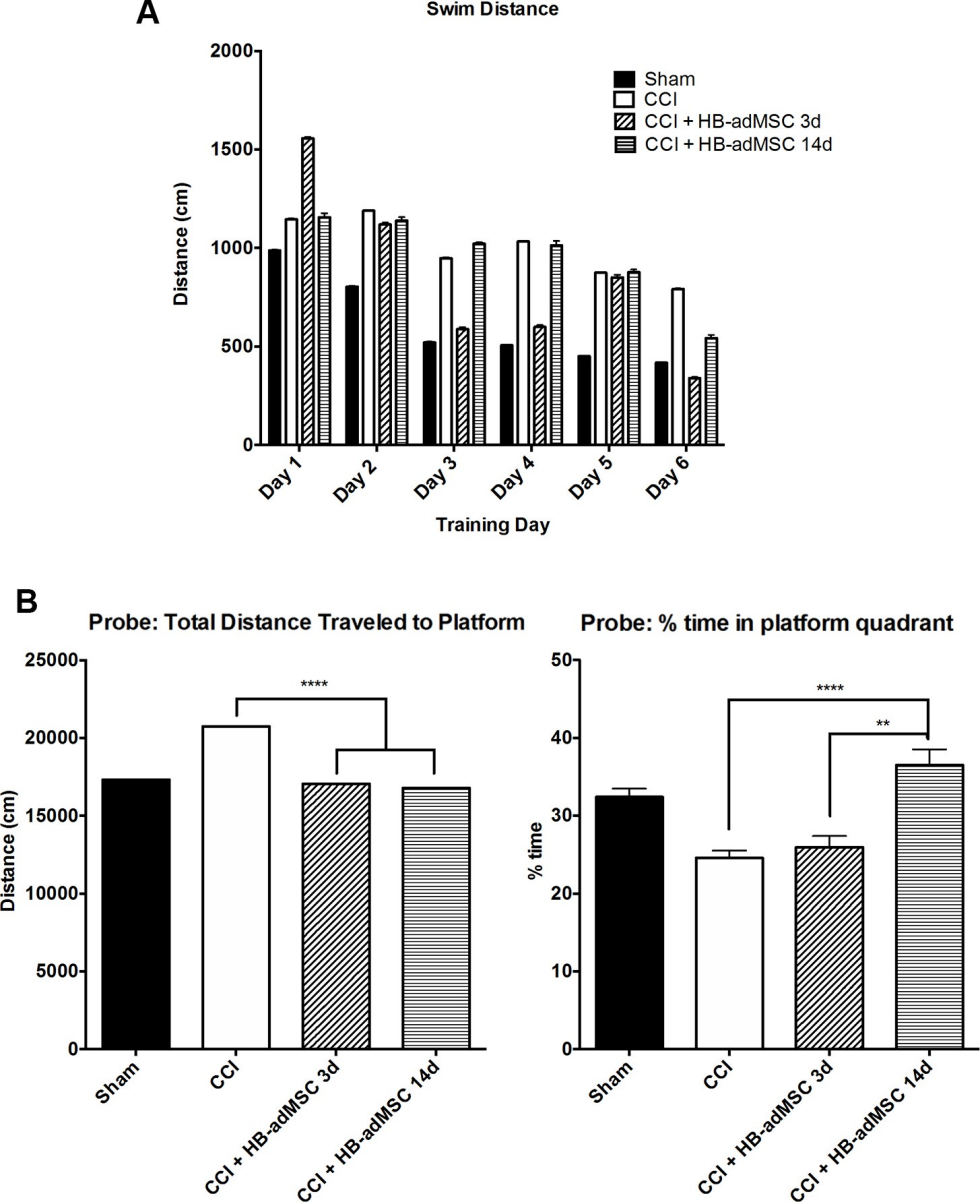
Swim distance and probe trials. Treatment significantly decreased the length of swim on days 2–6 in 3d treated animals. **A.** Length of swim was significantly decreased on days 2, 3, 5 in 14d treated animals. On days 3, 4, 5 3d treated animals swam significantly shorter distances than 14d treated group. Animals treated at 3d and 14d exhibited decreased distances traveled prior to reaching the location. Treated animals demonstrated significantly shorter swim distances. **B.** The percent of time spent in the platform quadrant was significantly higher for CCI + HB-adMSCs 14d animals compared to controls. Animals treated at 14d also spent significantly more time in the platform quadrant than those treated at 3d(*, p value<0.05; **, p value<0.01; ****, p value<0.0001). Sham, n = 10, CCI + PBS, n = 13, CCI + HB-adMSCs 3d, n = 7, CCI + HB-adMSCs 14d, n = 3.

Treatment with HB-adMSCs at 3 days post-CCI significantly decreased swim distance to the submerged platform in the Morris water maze (Fig 3A). Animals exhibited significantly shorter swim distances when compared to injured controls on test days 2, 3, 4, 5 and 6. Animals treated with HB-adMSCs at 14 days post-CCI demonstrated significantly shorter swim distances compared to injured controls on test days 2, 3 and 6. Decreased swim distance suggests improvements in spatial memory, as the animal is able to remember the platform location and exhibits less searching activity than animals with memory deficits. On test day 7, animals perform a probe trial in the Morris water maze. Both early and delayed treatment resulted in significantly shorter swim distances when compared to injured controls (Fig 3A). While both treatment groups exhibit sham-like swim distances, there is no significant difference between treatment groups. Data represents means ± SEM and statistical analysis by Two-Way ANOVA. Sham, n = 10, CCI + PBS, n = 13, CCI + HB-adMSCs 3d, n = 7, CCI + HB-adMSCs 14d, n = 3.

Interestingly, the results of the probe trial show a significant difference in the percent of time spent swimming in the quadrant previously occupied by the platform (Fig 3B). Animals receiving delayed treatment spent significantly more time in the platform quadrant than both injured controls (****, p<0.0001) and early treatment animals (**, p<0.01). Extended time in the platform quadrant during the probe trial supports the recovery of spatial memory observed in the previous testing trials.

### Microglial activation and polarization

**Fig 4:**
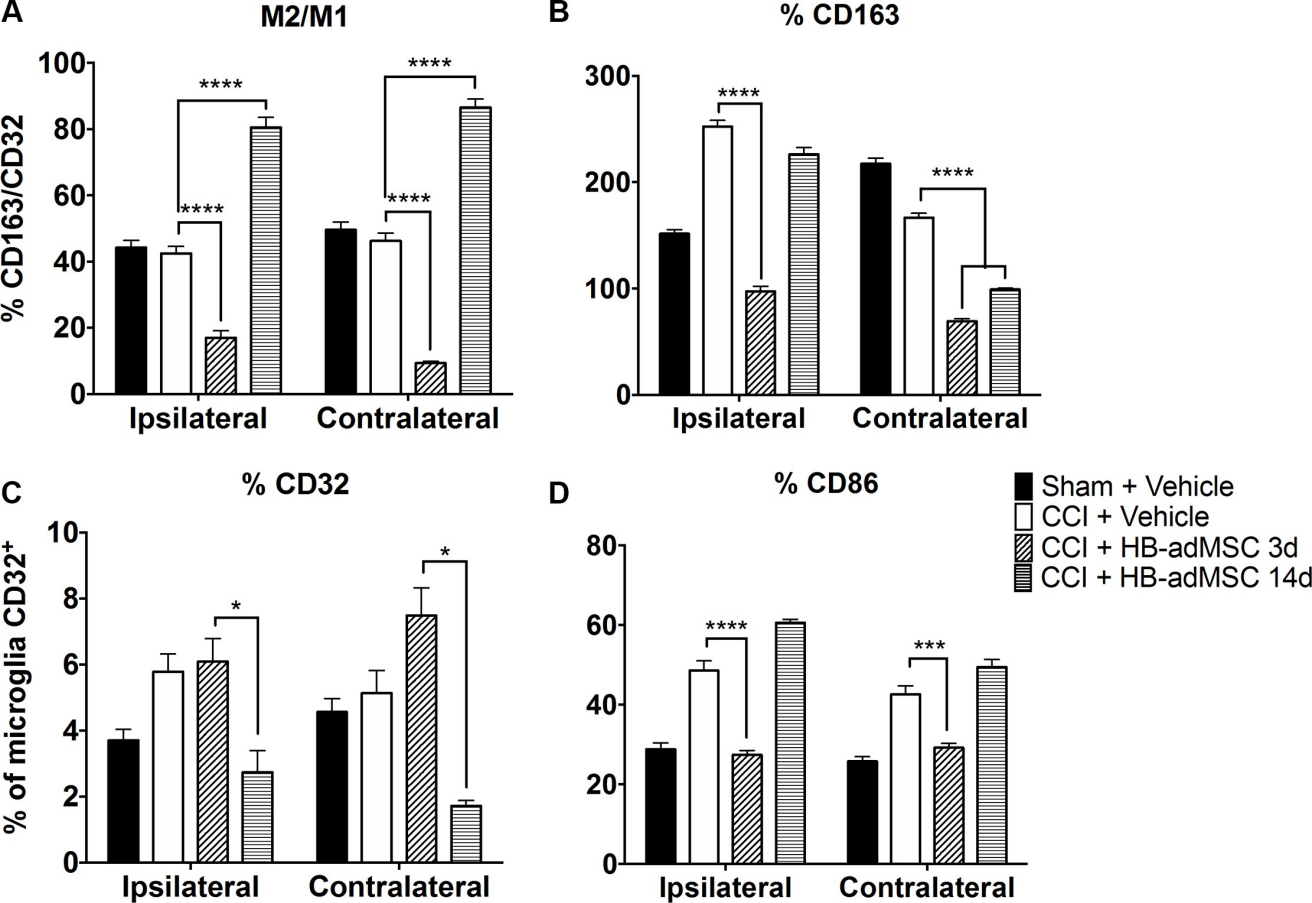
Rat brain microglia flow analysis. The microglia were gated from CD11b/c enriched myeloid cells and defined as CD45+CD11b/c+p2y12+. Microglia were then analyzed for M1 (CD32, CD86) and M2 (CD163) phenotype markers. **A.** The ratio of CD163+/CD32+ microglia reveals a significant shift toward anti-inflammatory M2 microglia in animals treated at 14 days post-injury, whereas, the 3d treatment group displays a preference toward M1. **B.** M2 marker CD163 is significantly lower than injured controls for both treatment groups. **C.** There is a significantly smaller percentage of cells that are positive for M1 marker CD32 in 14d treatment group. **D.** The same is true for M1 marker CD86 in 3d treatment group. Data represent means ± SEM. Statistical analysis performed by Two-way ANOVA with Tukey’s post hoc test. **, p value<0.01; ****, p value <0.0001. Sham, n = 8, CCI + PBS, n = 8, CCI + HB-adMSCs 3d, n = 4, CCI + HB-adMSCs 14d, n = 2.

Microglia activation and polarization was analyzed by flow cytometry as a measure of neuroinflammation following TBI and adMSCs treatment (Fig 4). The overall percentage of microglia relative to CD11b/c enriched cells was found to not change significantly between treatment groups. Overall, activation markers seem to decrease relative to injured rats not treated with HB-adMSCs. Ipsilateral hemispheres from CCI + HB-adMSCs 3d animals had 6.09% (CD32), 27.42% (CD86), and 97.45% (CD163) positive microglia. Similar tissue from CCI + HB-adMSCs 14d animals had 2.74% (CD32), 60.63% (CD86), and 226.41% (CD163) positive microglia. Compared to injured controls, there was a significant decrease in the %CD32 microglia (*, p value<0.05) in CCI + HB-adMSCs 14d. In comparison to injured controls, there was a significant decrease in %CD86 microglia (****, p value<0.0001), as well as in %CD163 microglia (****, p value<0.0001) in CCI + HB-adMSCs 3d animals. An inhibition of microglial activation is evident in both ipsilateral and contralateral hemispheres of animals treated early and at the delayed time point compared to those of untreated animals. However, there is no clear distinction of preference of polarization towards anti- or pro-inflammatory.

The ratio of microglia: macrophages was calculated as the percent of CD45+CD11b/c+p2y12+ cells over the percent of CD45+CD11b/c+p2y12- cells. In the ipsilateral hemispheres, the ratio for CCI + HB-adMSCs 3d animals was significantly lower than injured controls (17.02% vs 42.47%). In CCI + HB-adMSCs 14d tissue, the ratio was significantly higher than injured controls (80.56% vs 42.47%). The ratio of the % CD163/CD32 reveals a significant shift toward anti-inflammatory M2 microglia in animals treated at 14 days post-injury, whereas, the 3d treatment group displays a preference toward M1. There is a significantly smaller percentage of cells that are positive for M1 marker CD32 in 14d treatment group. However, the same is true for M1 marker CD86 in 3d treatment group. M2 marker CD163 is lower than injured controls for both treatment groups but did not reach statistical significance with our limited power for the 14d treatment group in the ipsilateral hemisphere. Data represent means ± SEM. Statistical analysis performed by Two-way ANOVA with Tukey’s post hoc test. ****, p value <0.0001. Sham, n = 8, CCI + PBS, n = 8, CCI + HB-adMSCs 3d, n = 4, CCI + HB-adMSCs 14d, n = 2.

### Representative localization of neurogenesis and neuroinflammation

**Fig 5:**
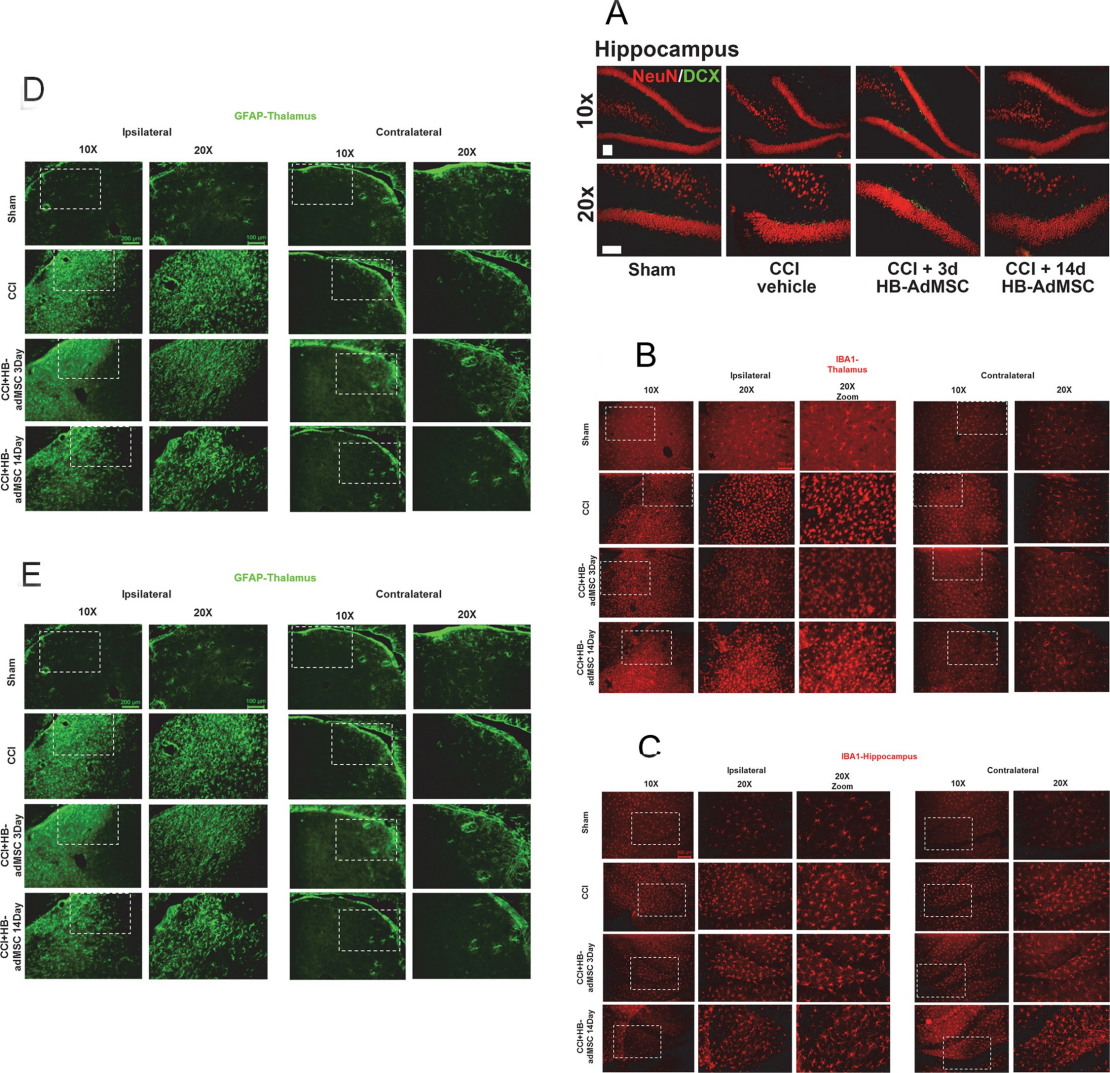
Representative localization of neuroinflammation and neurogenesis. Thin sections from ipsilateral and contralateral hemispheres were immunostained at Day 32. Presented here are portions of the thalamus and hippocampus, specifically the subgranular zone (SGZ). **A.** Antibodies for NeuN and Doublecortin (DCX) were used to stain for neurogenesis, **B, C.** IBA-1 for microglial activation and **D, E.** GFAP for reactive astrocytes. Images are representative of sham, CCI + vehicle, CCI + HB-adMSCs 3d and CCI + HB-adMSCs 14d, at 20x magnification with a 10x inset showing a larger field. Scale bars indicate 200 μm.

Thin tissue sections of ipsilateral and contralateral brain hemispheres were harvested from sham and CCI animals at Day 32 and were prepared for immunohistochemistry. Tissues were stained with antibodies and analyzed with fluorescent microscopy. To measure neurogenesis, tissues were stained with doublecortin (DCX) and NeuN (Fig 5A). In the subgranular zone (SGZ) of the hippocampus, there was an increase in positively stained neurons in early treated animals compared to injured controls. DCX is expressed during development, as well as in adult structures during migration of neuroblasts. Neuronal precursor cells and immature neurons stain positive for DCX, possibly indicating neurogenesis [58]. Sections of thalamus were stained with IBA-1 and analyzed. There is an increased appearance of IBA-1^+^ microglia associated with CCI, which appears to be reduced when treated with HB-adMSCs at 3d (Fig 5B). In the SGZ of the hippocampus, we observed an increase in IBA-1 positive microglia associated with CCI. Treatment with HB-adMSCs at 14d reduced the appearance of visible IBA-1^+^ microglia, as did treatment with HB-adMSCs at 3d, however to a lesser extent (Fig 5B). In the thalamus, GFAP^+^ astrocytes appear to be more prominent after CCI, however there is no visible reduction following HB-adMSC at 3d or at 14d (Fig 5C). Alternatively, sections of hippocampus stained with GFAP appear to have increased GFAP^+^ astrocytes in the SGZ (Fig 5D). This perceived increase of activated astrocytes is associated with CCI and is reduced with HB-adMSCs at 3d, and slightly less so with HB-adMSCs at 14d. The images in Fig 5 are representative of the injury and treatment groups in this study. Sham, n = 3, CCI + PBS, n = 3, CCI + HB-adMSCs 3d, n = 2, CCI + HB-adMSCs 14d, n = 2.

## Discussion

In this study, we demonstrate the efficacy of early and delayed treatment of TBI with HB-adMSCs. To our knowledge, this is the first report to compare human adipose-derived mesenchymal stem cells delivered intravenously 14 days post-injury in a rat CCI model as compared to a sub-acute delivery 3d after injury. HB-adMSCs administered at 3 days post-injury effectively diminished M1 microglia, evidenced by flow cytometry, and may increase neurogenesis in the subgranular zone of the hippocampus. When HB-adMSCs were administered at 14 days post-injury, the percentage of microglia exhibiting M2 markers and the M2/M1 ratio were significantly increased, signaling an anti-inflammatory shift. Treatment at either timepoint resulted in significantly improved spatial memory performance in the MWM. HB-adMSCs are shown to be effective in modulating the inflammatory response to facilitate a more restorative, anti-inflammatory environment during the early stages of TBI. Our study has several notable limitations and is at times underpowered as we did not expect to see any notable effects from such a delayed treatment based upon existing literature. Future studies are required to better quantify the differences between sub-acute and delayed treatments and long-term effects of HB-adMSCs to treat TBI.

Deficits in neurocognitive function are commonly associated with TBI. In animal models, the use of MWM is standard for assessment of injury-induced cognitive impairment. Using a spatial memory paradigm, we examined the effects of early and delayed treatment with HB-adMSCs. The delayed treatment group displayed unique behavior with a significant increase in the amount of time spent in the platform quadrant when compared to control and early treatment groups. This result suggests additional recovery or preservation of spatial learning and memory in animals treated at 14 days post-TBI. Neurological reflexes were tested with a brief neuroscore tested 7 days post-TBI and revealed a significant decrease in neurological deficits in the early treatment animals. Potentially in coordination with the improved neurological function, qualitative immunohistochemical analysis for neurogenesis indicated an increase in DCX^+^ neurons in the subgranular zone of early treatment animals compared to control tissues. Flow cytometry analysis of brain tissues revealed a significant decrease in activation of microglia in both treatment groups when compared to injured controls. Calculated ratios suggest a significant shift toward pro-inflammatory M1 phenotype for animals treated at 3d post-TBI, while those treated at 14 days post-TBI show a shift towards anti-inflammatory M2 phenotype. This novel data set shows that delayed treatment of TBI with HB-adMSCs is effective in decreasing cognitive and neurological deficits and supports anti-inflammatory pathways.

There is an abundance of literature demonstrating that MSCs are effective in the treatment of TBI [5, 19, 24, 59–67], yet the mechanisms responsible are not well understood. Among the proposed mechanisms of action for MSCs, paracrine signaling and its effects on immunomodulation and inflammation are among the most important functions. In a previous report, we demonstrated that by either knocking down or over-expressing COX2, we are able to block the therapeutic effects of MSCs, or enhance them, respectively [18]. Similar studies have indicated that TSG-6 can be critical for efficacy of MSCs [68], as well as a number of other immunomodulatory cytokines or paracrine growth factors, including IDO and IL-1ra.

We performed a set of assays on a representative batch of HB-adMSCs to evaluate their immunomodulatory potential (S3 Fig). Our studies indicate that HB-adMSCs are effective in inhibiting TNF-α and IFN-γ secretion from LPS or concanavlin A activated primary splenocytes in a dose dependent manner. We have previously shown that PGE2 secretion by hBM-MSCs is a key mediator of MSC efficacy to treat TBI and can be used as an indicator for *in vivo* therapeutic potential [18], and we found that HB-adMSC significantly increase the accumulation of PGE2 in the LPS-activated co-cultures (S3 Fig). We also found that treatment of HB-adMSCs with TNF-α significantly induced the expression of TSG-6 and IL-1ra and treatment with IFN-γ resulted in a large increase in the expression of IDO. These immune modulating molecules are all known to contribute to the efficacy of MSC therapies [68–70].

Neuroinflammation is a hallmark of TBI and is considered a major component in the early and chronic deficiencies of TBI [71–74]. Neuroinflammation develops through a complex process which includes glial activation, leukocyte recruitment and production and secretion of cytokines and chemokines. Microglia play a significant role in neuroinflammation as they are the resident immune cells in the brain and undergo phenotypic transformation and activation as a result of injury [75–78]. Microglia have been reported to exhibit both neurotoxic and neuroprotective effects after CNS injury [79]. Injury-activated microglia secrete IL-1a, TNF-a and C1q, inducing neurotoxic reactive astrocytes, which limits the promotion of neuron survival, outgrowth, synaptogenesis and phagocytosis [80]. In a classical paradigm based upon polarization, similar to macrophage, microglia exhibiting an M1 phenotype release proinflammatory cytokines, chemokines and oxidative mediators, while microglia with M2 phenotype release anti-inflammatory and neurotrophic factors that are more conducive to tissue repair [8].

In this study, we employed a highly sensitive flow cytometric analysis to isolate and quantify microglia in both hemispheres of control, CCI and treated animals. This technique provides information regarding the microglial population and their polarization to either M1-like or M2-like phenotypes. Brain tissue was harvested and analyzed by flow cytometry using a panel of myeloid markers. Microglia were identified using logic gating as CD45+, CD11b/c+, and P2Y12 and analyzed separate from other infiltrating myeloid populations. The resolution of microglia from macrophage is notable in a rat model, as the lack of suitable reagents has forced most previous studies to analyze the two different cell populations together. We calculated the ratio of microglia: macrophages as the percent of cells CD45+CD11b/c+p2y12+: CD45+CD11b/c+p2y12^-^ demonstrating the relatively small number of microglia in comparison to macrophage even 32 d after injury.

Microglia were further analyzed using M1-associated markers (CD32 and CD86) and an M2-associated marker (CD163). Following tissue harvest and flow cytometry analysis, the activation markers seem to decrease relative to injured rats not treated with cells. CD163, an anti-inflammatory M2-associated marker, was significantly decreased when compared to injured controls in both the ipsilateral and contralateral hemispheres of animals treated at early or delayed timepoint. However, the percentage of microglia expressing CD163 observed in the ipsilateral hemisphere of early treated animals and the contralateral hemisphere of both treatment groups were similar (****, p <0.0001). The ipsilateral hemisphere of delayed treatment group animals also showed a significant decrease in CD163^+^ microglia (**, p <0.01). The ratio of M2/M1 (CD163/CD32) reveals a significant shift toward anti-inflammatory M2 microglia in animals treated at 14 days post-injury, whereas, the 3d treatment group displays a preference toward M1. There is a significantly smaller portion of the microglia population that are positive for M1 markers (CD86, CD32) in 3d treatment and 14d treatment groups, respectively. In mice, M1 markers are increased and remain high, while M2 markers rise and then fall around 5 days post-injury [81, 82]. In some cases, it has been shown that there is a secondary peak in M2 activation around 28 days post-injury [83]. In this study, the delayed treatment with HB-adMSCs appears to result in increased M2 markers that remain elevated at 32 days post-injury. This extended period or secondary increase of anti-inflammatory activity may be reflected in some of the behavioral improvements associated with the delayed treatment group.

Lesions to the hippocampus are associated with spatial learning and memory deficits which is routinely measured with the Morris water maze [84, 85]. A thorough review by Hall *et al*.,[86] describes the characteristics of CCI and its ability to generate enduring cognitive deficits and widespread hippocampal degeneration, injuries that are analogous to those seen in human TBI patients [87]. It is reasonable to use a sensitive MWM paradigm to evaluate hippocampal damage and effects of treatment and to extrapolate observations for translation to human studies. The restoration of spatial memory and learning in HB-adMSCs-treated animals is of particular interest in the context of increased neurogenesis in the ipsilateral SGZ. Therefore, the observed neurogenesis and increased performance in spatial learning and memory tasks in HB-adMSCs-treated animals in our study are very interesting and are in accordance with the literature [88], even in the absence of motor or vestibular deficits (S1, S2 Figs).

The dentate gyrus (DG) of the hippocampus contains precursor cells in the subgranular zone (SGZ) which develop into hippocampal neurons playing a critical role in learning and memory [89–94]. In this study, we observed a significant increase in DCX^+^ cells in the ipsilateral SGZ region of hippocampus of animals treated with HB-adMSCs at 3 days post-injury. This is in accordance with data we have previously published reporting that BM-MSCs increased the number of DCX^+^ cells associated with neurogenesis [48]. In newly generated cells of the adult dentate gyrus, DCX displays a transient expression profile, peaking between 4–7 days, then decays rapidly, becoming undetectable by 30 days. This is inversely proportional to the expression of NeuN which begins to rise between 10–14 days and remains elevated in mature adult neurons [51]. In this study, animals received HB-adMSCs at either 3 days or 14 days post-CCI and tissues were harvested at 32 days post-CCI and analyzed for DCX staining. Our results revealed an increase in DCX^+^ cells in the 3d treatment group, but not the 14d treatment group in the SGZ. Testing occurs between weeks 3 and 4 after early treatment or between weeks 1 and 2 in delayed treated animals, possibly indicating that the cell therapy may require a minimum amount of time before neurogenesis is detected.

In this region, we also observed increased evidence of neuroinflammation (activated microglia) which was reduced when treated with HB-adMSCs at 14d, as well as activated astrocytes which were reduced with HB-adMSCs at 3d. In addition to the hippocampus, sections of the thalamus were also stained and analyzed for microglial activation and reactive astrocytes. Activated thalamic microglia associated with CCI appear to be reduced when treated with HB-adMSCs at 3d. However, treatment with HB-adMSCs at either 3d or 14d did not seem to have an effect on activated astrocytes. These results support the idea that the relationship between glia and neuroinflammation is a complex one, with a spectrum of activation. In this study, the immunohistochemical analysis is representative of the effect of adMSCs delivered at early and delayed time points after injury has on common indicators of neuroinflammation and potential association with cognitive function. We utilize flow cytometric analysis of multiple surface markers for inflammation to define and characterize microglia polarization associated with TBI and subsequent treatment with HB-adMSCs. Using our alternative method has given us significant quantitative data previously [50] and can be more useful, in some cases, than unbiased stereological analysis of immunohistochemical staining of tissue sections. With the systemic infusion of cells, a systemic quantification method seems more reasonable than isolated examination of positively stained antibodies in tissue sections.

Previous studies have demonstrated the use of adMSC to treat TBI, however not much is currently known about the optimal time for therapy administration. Commonly, studies focus on very early delivery time (<24hrs) due to the belief that treatment is most effective when delivered soon after injury, targeting the early stages of secondary injury. Our study adds novel data to the field by directly comparing this sub-acute treatment with a “late” 14d treatment, creating a window for the isolation, expansion, and delivery of autologous cells post-injury. Our results, when considered in composite, can be interpreted several ways. In a simple model, both treatment strategies may have had the same therapeutic effect on neuroinflammation through a series of steps that require sequential changes in microglia. Since the two treatment groups were analyzed at the same time after injury, but not the same time after treatment, we may have captured different snapshots of a progressive response. Alternatively, there may be multiple therapeutic windows that present themselves following TBI. Many groups target a very acute delivery with therapeutic results. We have repeatedly found that the delivery of MSCs 3d after injury results in a reliable improvement in outcome in previous studies of bone marrow-derived MSCs [48]. It is not inconceivable that there are additional windows where a cell therapy could yield significant improvements in neuroinflammation and cognitive outcome. The identification and optimization of these targets could inform future therapies involving multiple treatments and provide some benefit to those patients that are unable to receive a cell therapy during their acute or sub-acute phases of injury.

## Supporting information

S1 Fig Short neurological assessment. There is a significant decrease in neuroscore on day 7 in animals treated at 3d when compared to injured controls. Data represent mean scores ± SEM. Statistical analysis performed by Two‐way ANOVA with Tukey’s post hoc test. (*, p<0.05). Sham, n = 10, CCI + PBS, n = 13, CCI + HB‐adMSCs 3d,n = 7, CCI + HB‐adMSCs 14d, n = 3.
(PDF)

S2 Fig Beam balance and walking. Beam balance results indicate a significant improvement in CCI + HB‐adMSCs 3d animals at Day 14 compared to injured controls (****, p value<0.0001). No significant difference was seen in beam walking measurements. Values represent means ± SEM. Statistical analysis performed by Two‐way ANOVA. Sham, n = 10, CCI + PBS, n = 13, CCI + HB‐adMSCs 3d, n = 7, CCI + HB‐adMSCs 14d, n = 3.
(PDF)

S3 Fig *In Vitro* HB‐adMSC analysis. HB‐adMSCs reduce the secretion of TNF‐α and IFN‐γ from LPS or concanavalin A stimulated rat splenocyte co‐cultures in a dose‐dependent fashion (top left and top right, respectively). This reduction in TNF‐α was accompanied by an inverse relationship between the number of HB‐adMSC in the co‐cultures and PGE2 (bottom left). Quantitative PCR detected an increase in several possible mechanisms of immune modulation when HB‐adMSC were stimulated with either TNF‐α or IFN‐γ (50ng/ml each, bottom right). Values represent means ± SD. *, p value <0.05; ***, p value <0.001; ****, p value <0.0001. Statistical analysis performed by One‐way ANOVA with multiple comparison Sidak’s test or Fisher’s LSD, as noted.
(PDF)

S1 Table *In Vitro* HB‐adMSC analysis. HB‐adMSC have characteristic traits consistent with conventional MSC, including a phenotype negative for CD31, CD34, CD45, and HLA‐DR and positive for CD29, CD44, CD73, and CD90. Value is expressed as a percentage.
(PDF)

S2 Table Full statistical comparisons between treatment groups. Complete statistical comparisons between all the groups presented in Fig 4.
(PDF)

S1 File (ZIP)
