# Correction: Human adipose-derived mesenchymal stem cells for acute and sub-acute TBI

**DOI:** 10.1371/journal.pone.0261599

**Published:** 2021-12-14

**Authors:** Katherine A. Ruppert, Karthik S. Prabhakara, Naama E. Toledano-Furman, Sanjna Udtha, Austin Q. Arceneaux, Hyeonggeun Park, An Dao, Charles S. Cox, Scott D. Olson

Fig 5 is incorrect. The authors have provided a corrected version here.

**Fig 5:**
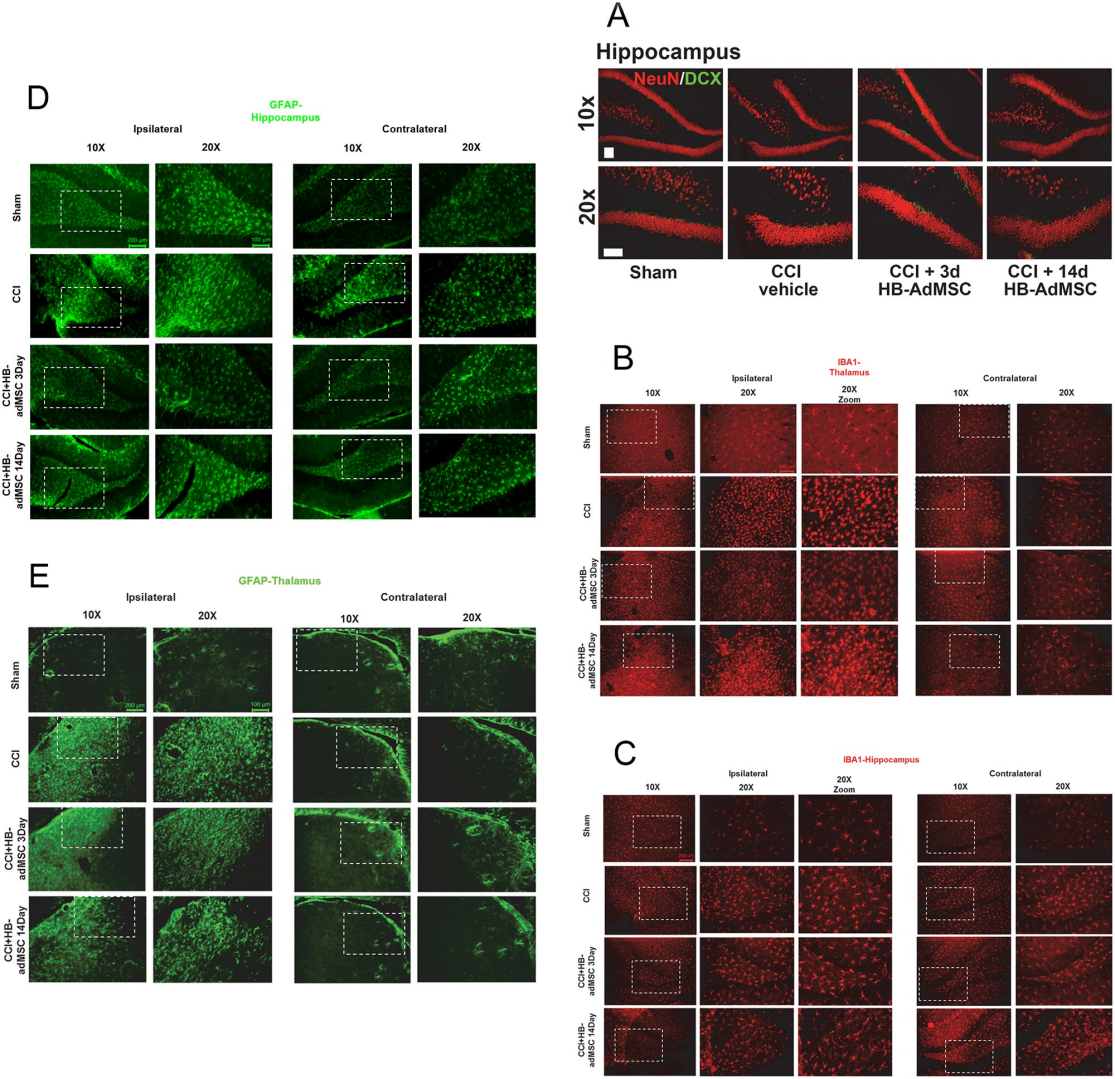
Representative localization of neuroinflammation and neurogenesis. Thin sections from ipsilateral and contralateral hemispheres were immunostained at Day 32. Presented here are portions of the thalamus and hippocampus, specifically the subgranular zone (SGZ). **A.** Antibodies for NeuN and Doublecortin (DCX) were used to stain for neurogenesis, **B, C.** IBA-1 for microglial activation and **D, E.** GFAP for reactive astrocytes. Images are representative of sham, CCI + vehicle, CCI + HB-adMSCs 3d and CCI + HB-adMSCs 14d, at 20x magnification with a 10x inset showing a larger field. Scale bars indicate 200 μm.

## References

[1] Ruppert KA, Prabhakara KS, Toledano-Furman NE, Udtha S, Arceneaux AQ, Park H, et al. (2020) Human adipose-derived mesenchymal stem cells for acute and sub-acute TBI. PLoS ONE 15(5): e0233263. 10.1371/journal.pone.0233263 32453741 PMC7250455

