# Supplementary material for: Human adipose-derived mesenchymal stem cells for acute and sub-acute TBI
Source: PLoS One. 2020 May 26;15(5):e0233263. doi: 10.1371/journal.pone.0233263 (PMC7250455; doi:10.1371/journal.pone.0233263)
Supplement: S1 Fig — There is a significant decrease in neuroscore on day 7 in animals treated at 3d when compared to injured controls. Data represent mean scores ± SEM. Statistical analysis performed by Two‐way ANOVA with Tukey’s post hoc test. (*, p<0.05). Sham, n = 10, CCI + PBS, n = 13, CCI + HB‐adMSCs 3d,n = 7, CCI + HB‐adMSCs 14d, n = 3. (PDF) [file pone.0233263.s001.pdf]

Supplemental Data

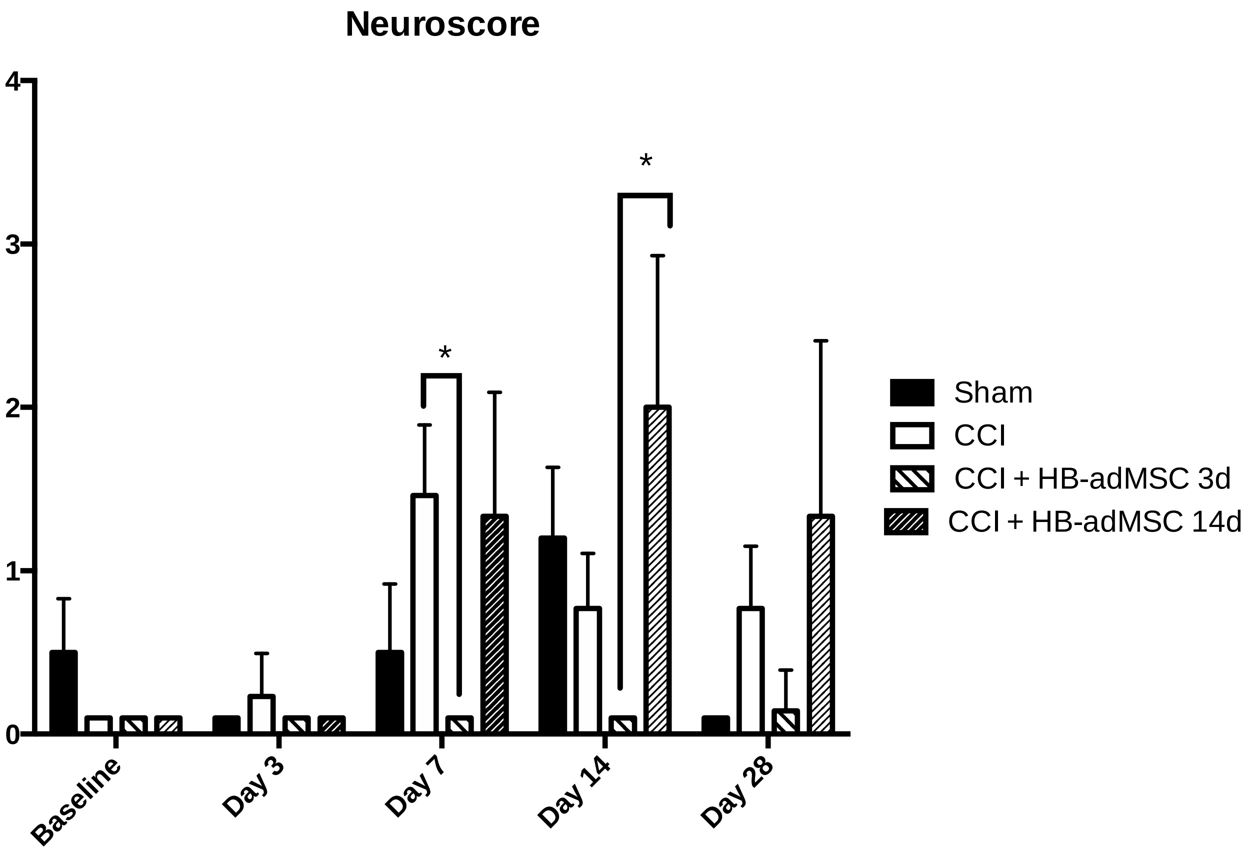

Supplemental Fig 1. Short Neurological Assessment There is a significant decrease in neuroscore on day 7 in animals treated at 3d when compared to injured controls. Data represent mean scores ± SEM. Statistical analysis performed by Two-way ANOVA with Tukey's post hoc test. (*, $p<0.05$). Sham, n= 10, CCI + PBS, n=13, CCI + HB-adMSCs 3d, n=7, CCI + HB-adMSCs 14d, n=3.
