# Supplementary material for: Human adipose-derived mesenchymal stem cells for acute and sub-acute TBI
Source: PLoS One. 2020 May 26;15(5):e0233263. doi: 10.1371/journal.pone.0233263 (PMC7250455; doi:10.1371/journal.pone.0233263)
Supplement: S2 Fig — Beam balance results indicate a significant improvement in CCI + HB‐adMSCs 3d animals at Day 14 compared to injured controls (****, p value<0.0001). No significant difference was seen in beam walking measurements. Values represent means ± SEM. Statistical analysis performed by Two‐way ANOVA. Sham, n = 10, CCI + PBS, n = 13, CCI + HB‐adMSCs 3d, n = 7, CCI + HB‐adMSCs 14d, n = 3. (PDF) [file pone.0233263.s002.pdf]

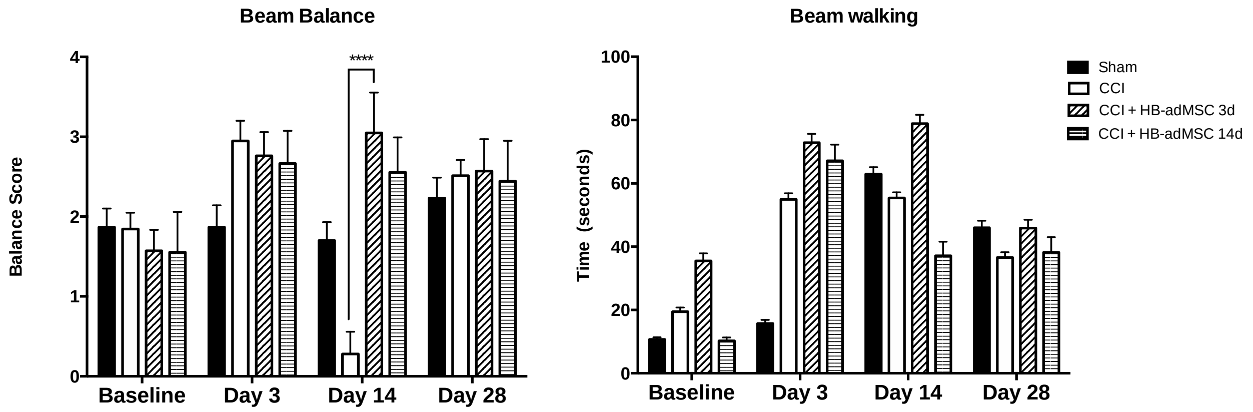

Supplemental Fig 2. Beam Balance and Walking

Beam balance results indicate a significant improvement in CCI + HB-adMSCs 3d animals at Day 14 compared to injured controls (****, p value<0.0001). No significant difference was seen in beam walking measurements. Values represent means ± SEM. Statistical analysis performed by Two-way ANOVA. Sham, n= 10, CCI + PBS, n=13, CCI + HB-adMSCs 3d, n=7, CCI + HB-adMSCs 14d, n=3.
