# Supplementary material for: Human adipose-derived mesenchymal stem cells for acute and sub-acute TBI
Source: PLoS One. 2020 May 26;15(5):e0233263. doi: 10.1371/journal.pone.0233263 (PMC7250455; doi:10.1371/journal.pone.0233263)
Supplement: S3 Fig — HB‐adMSCs reduce the secretion of TNF‐α and IFN‐γ from LPS or concanavalin A stimulated rat splenocyte co‐cultures in a dose‐dependent fashion (top left and top right, respectively). This reduction in TNF‐α was accompanied by an inverse relationship between the number of HB‐adMSC in the co‐cultures and PGE2 (bottom left). Quantitative PCR detected an increase in several possible mechanisms of immune modulation when HB‐adMSC were stimulated with either TNF‐α or IFN‐γ (50ng/ml each, bottom right). Values represent means ± SD. *, p value <0.05; ***, p value <0.001; ****, p value <0.0001. Statistical analysis performed by One‐way ANOVA with multiple comparison Sidak’s test or Fisher’s LSD, as noted. (PDF) [file pone.0233263.s003.pdf]

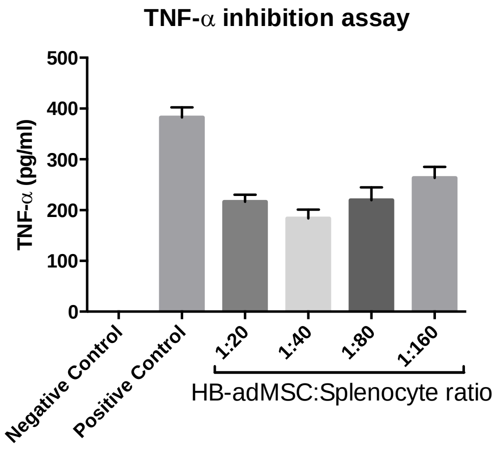

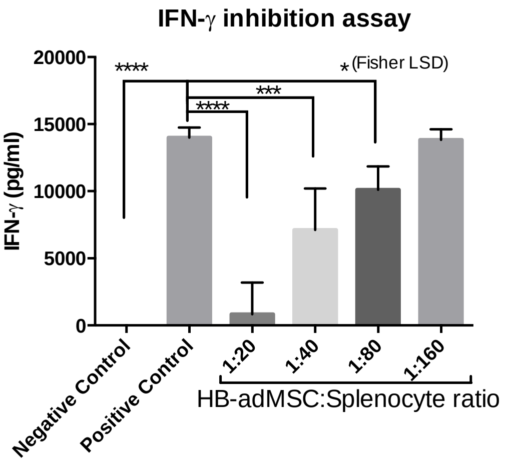

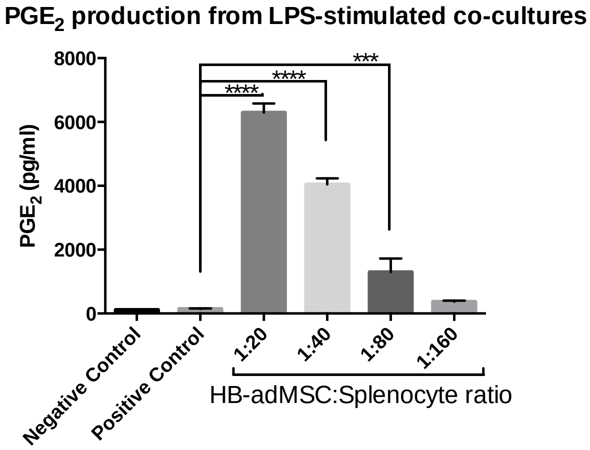

Fold Change vs. Resting HB-adMSC ΔΔCt (stdev)

	COX-2	IDO	TSG-6	IL-1ra
TNF-α	2.58 (0.16)	2.53 (0.17)	4.19 (0.10)	27.97 (0.11)
IFN-γ	1.41 (0.24)	3110.34 (0.15)	1.81 (0.08)	0.50 (0.07)

Supplemental Fig 3. *In Vitro* HB-adMSC Analysis

HB-adMSCs reduce the secretion of TNF-α and IFN-γ from LPS or concanavalin A stimulated rat splenocyte co-cultures in a dose-dependent fashion (top left and top right, respectively). This reduction in TNF-α was accompanied by an inverse relationship between the number of HB-adMSC in the co-cultures and PGE_2 (bottom left). Quantitative PCR detected an increase in several possible mechanisms of immune modulation when HB-adMSC were stimulated with either TNF-α or IFN-γ (50ng/ml each, bottom right). Values represent means ± SD. *, p value <0.05; ***, p value <0.001; ****, p value <0.0001. Statistical analysis performed by One-way ANOVA with multiple comparison Sidak's test or Fisher's LSD, as noted.
