# Supplementary material for: Human adipose-derived mesenchymal stem cells for acute and sub-acute TBI
Source: PLoS One. 2020 May 26;15(5):e0233263. doi: 10.1371/journal.pone.0233263 (PMC7250455; doi:10.1371/journal.pone.0233263)
Supplement: S1 Table — HB‐adMSC have characteristic traits consistent with conventional MSC, including a phenotype negative for CD31, CD34, CD45, and HLA‐DR and positive for CD29, CD44, CD73, and CD90. Value is expressed as a percentage. (PDF) [file pone.0233263.s004.pdf]

		Positive				Negative			
Donor	passage	**CD73+**	**CD90+**	**CD44**	**CD29**	**CD34+**	**CD45+**	**HLA DR+**	**CD31**
**A**	2	97.984	96.938	80.088	99.354	0	0	0.037	0
	4	98.644	76.441	98.552	99.749	0.017	0	0	0.023
	8	96.692	71.554	83.539	95.91	0	0.031	0.031	0.112
	12	84.944	71.19	87.349	95.783	0	0	0.186	0.151
**B**	2	98.175	94.681	82.497	99.278	0	0	0.024	0.01
	4	98.703	82.248	98.087	99.854	0.027	0.027	0.027	0
	8	99.21	97.315	90.492	99.48	0	0	0.053	0.062
	12	89.242	88.143	86.945	99.104	0.058	0	0.058	0.195
**C**	2	96.225	88.88	74.748	99.711	0.011	0.023	0.046	0
	4	99.404	74.348	97.946	99.893	0.012	0.012	0	0
	8	98.989	80.699	89.333	99.625	0.013	0	0.026	0.08
	12	92.078	68.909	77.62	96.601	0	0.149	0	0.85
**D**	2	98.451	94.218	85.797	99.856	0.018	0.018	0.036	0
	4	99.68	71.383	98.499	99.83	0	0	0	0.032
	8	98.932	87.315	95.554	99.431	0.013	0	0.027	0.032
	12	78.415	75.137	87.94	98.151	0	0.546	0	0.616
**Control**	8	96.855	93.711	82.535	98.642	0	0	0.21	0.197
	12	99.146	98.719	79.906	98.823	0.107	0.534	1.387	0.157

Supplemental Table S1. ***In Vitro*** **HB-adMSC Analysis**
HB-adMSC have characteristic traits consistent with conventional MSC, including a phenotype negative for CD31, CD34, CD45, and HLA-DR and positive for CD29, CD44, CD73, and CD90. Value is expressed as a percentage.
