# Supplementary material for: Human adipose-derived mesenchymal stem cells for acute and sub-acute TBI
Source: PLoS One. 2020 May 26;15(5):e0233263. doi: 10.1371/journal.pone.0233263 (PMC7250455; doi:10.1371/journal.pone.0233263)
Supplement: S2 Table — Complete statistical comparisons between all the groups presented in Fig 4. (PDF) [file pone.0233263.s005.pdf]

Supplemental Table S2. Full statistical comparisons between treatment groups. Complete statistical comparisons between all the groups presented in **Figure 4**.

%CD32				
Number of families	2			
Number of comparisons per family	6			
Alpha	0.05			
Tukey's multiple comparisons test	Mean Diff.	95% CI of diff.	Significant?	Summary
Ipsilateral				
Sham + Vehicle vs. CCI + Vehicle	-2.072	-3.995 to -0.1495	Yes	*
Sham + Vehicle vs. CCI + HB-adMSC 3d	-2.386	-4.741 to -0.03122	Yes	*
Sham + Vehicle vs. CCI + HB-adMSC 14d	0.9693	-2.071 to 4.009	No	ns
CCI + Vehicle vs. CCI + HB-adMSC 3d	-0.3138	-2.669 to 2.041	No	ns
CCI + Vehicle vs. CCI + HB-adMSC 14d	3.042	0.001430 to 6.082	Yes	*
CCI + HB-adMSC 3d vs. CCI + HB-adMSC 14d	3.355	0.02509 to 6.686	Yes	*
Contralateral				
Sham + Vehicle vs. CCI + Vehicle	-0.5685	-2.491 to 1.354	No	ns
Sham + Vehicle vs. CCI + HB-adMSC 3d	-2.918	-5.273 to -0.5628	Yes	*
Sham + Vehicle vs. CCI + HB-adMSC 14d	2.846	-0.1944 to 5.886	No	ns
CCI + Vehicle vs. CCI + HB-adMSC 3d	-2.349	-4.704 to 0.005672	No	ns
CCI + Vehicle vs. CCI + HB-adMSC 14d	3.414	0.3741 to 6.454	Yes	*
CCI + HB-adMSC 3d vs. CCI + HB-adMSC 14d	5.763	2.433 to 9.094	Yes	***

%CD86				
Number of families	2			
Number of comparisons per family	6			
Alpha	0.05			
Tukey's multiple comparisons test	Mean Diff.	95% CI of diff.	Significant?	Summary
Ipsilateral				
Sham + Vehicle vs. CCI + Vehicle	-19.71	-26.23 to -13.18	Yes	****
Sham + Vehicle vs. CCI + HB-adMSC 3d	1.434	-6.556 to 9.423	No	ns
Sham + Vehicle vs. CCI + HB-adMSC 14d	-31.78	-42.09 to -21.47	Yes	****
CCI + Vehicle vs. CCI + HB-adMSC 3d	21.14	13.15 to 29.13	Yes	****
CCI + Vehicle vs. CCI + HB-adMSC 14d	-12.07	-22.39 to -1.758	Yes	*
CCI + HB-adMSC 3d vs. CCI + HB-adMSC 14d	-33.21	-44.51 to -21.91	Yes	****
Contralateral				
Sham + Vehicle vs. CCI + Vehicle	-16.86	-23.38 to -10.34	Yes	****

Sham + Vehicle vs. CCI + HB-adMSC 3d	-3.405	-11.39 to 4.585	No	ns
Sham + Vehicle vs. CCI + HB-adMSC 14d	-23.7	-34.02 to -13.39	Yes	****
CCI + Vehicle vs. CCI + HB-adMSC 3d	13.45	5.464 to 21.44	Yes	***
CCI + Vehicle vs. CCI + HB-adMSC 14d	-6.843	-17.16 to 3.472	No	ns
CCI + HB-adMSC 3d vs. CCI + HB-adMSC 14d	-20.3	-31.60 to -8.997	Yes	***

%CD163				
Number of families	2			
Number of comparisons per family	6			
Alpha	0.05			
Tukey's multiple comparisons test	Mean Diff.	95% CI of diff.	Significant?	Summary
Ipsilateral				
Sham + Vehicle vs. CCI + Vehicle	-100.8	-117.8 to -83.91	Yes	****
Sham + Vehicle vs. CCI + HB-adMSC 3d	54.17	33.45 to 74.90	Yes	****
Sham + Vehicle vs. CCI + HB-adMSC 14d	-74.79	-101.5 to -48.03	Yes	****
CCI + Vehicle vs. CCI + HB-adMSC 3d	155	134.3 to 175.7	Yes	****
CCI + Vehicle vs. CCI + HB-adMSC 14d	26.05	-0.7092 to 52.80	No	ns
CCI + HB-adMSC 3d vs. CCI + HB-adMSC 14d	-129	-158.3 to -99.65	Yes	****
Contralateral				
Sham + Vehicle vs. CCI + Vehicle	50.69	33.77 to 67.61	Yes	****
Sham + Vehicle vs. CCI + HB-adMSC 3d	148.2	127.4 to 168.9	Yes	****
Sham + Vehicle vs. CCI + HB-adMSC 14d	118.2	91.49 to 145.0	Yes	****
CCI + Vehicle vs. CCI + HB-adMSC 3d	97.48	76.75 to 118.2	Yes	****
CCI + Vehicle vs. CCI + HB-adMSC 14d	67.55	40.80 to 94.30	Yes	****
CCI + HB-adMSC 3d vs. CCI + HB-adMSC 14d	-29.93	-59.24 to -0.6194	Yes	*

CD163/CD32				
Number of families	2			
Number of comparisons per family	6			
Alpha	0.05			
Tukey's multiple comparisons test	Mean Diff.	95% CI of diff.	Significant?	Summary
Ipsilateral				
Sham + Vehicle vs. CCI + Vehicle	1.742	-6.080 to 9.565	No	ns
Sham + Vehicle vs. CCI + HB-adMSC 3d	27.2	17.62 to 36.78	Yes	****
Sham + Vehicle vs. CCI + HB-adMSC 14d	-36.34	-48.71 to -23.97	Yes	****
CCI + Vehicle vs. CCI + HB-adMSC 3d	25.46	15.88 to 35.04	Yes	****
CCI + Vehicle vs. CCI + HB-adMSC 14d	-38.08	-50.45 to -25.72	Yes	****
CCI + HB-adMSC 3d vs. CCI + HB-adMSC 14d	-63.54	-77.09 to -49.99	Yes	****
Contralateral				

Sham + Vehicle vs. CCI + Vehicle	3.33	-4.492 to 11.15	No	ns
Sham + Vehicle vs. CCI + HB-adMSC 3d	40.29	30.71 to 49.87	Yes	****
Sham + Vehicle vs. CCI + HB-adMSC 14d	-37	-49.37 to -24.63	Yes	****
CCI + Vehicle vs. CCI + HB-adMSC 3d	36.96	27.38 to 46.54	Yes	****
CCI + Vehicle vs. CCI + HB-adMSC 14d	-40.33	-52.70 to -27.96	Yes	****
CCI + HB-adMSC 3d vs. CCI + HB-adMSC 14d	-77.29	-90.84 to -63.74	Yes	****
