# Supplementary material for: Human adipose-derived mesenchymal stem cells for acute and sub-acute TBI
Source: PLoS One. 2020 May 26;15(5):e0233263. doi: 10.1371/journal.pone.0233263 (PMC7250455; doi:10.1371/journal.pone.0233263)
Supplement: S1 File — (ZIP) [file pone.0233263.s006.zip › Data/hbadmsc brains FC plots.pdf]

1 ipsi-2017-03-14-155101-1
Rat microglia M1M2 protocol - Imported

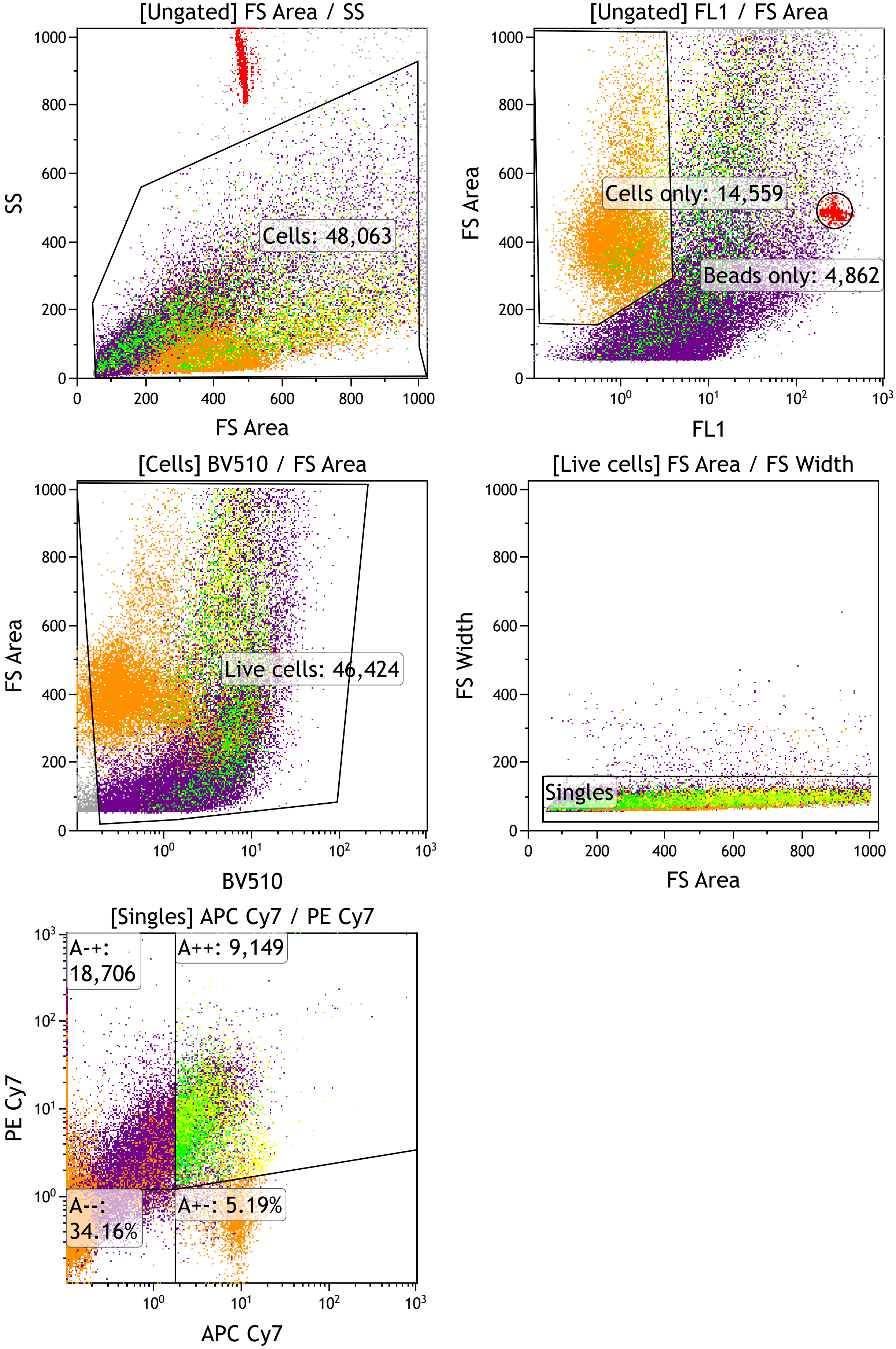

1 ipsi-2017-03-14-155101-1

Rat microglia M1M2 protocol - Imported

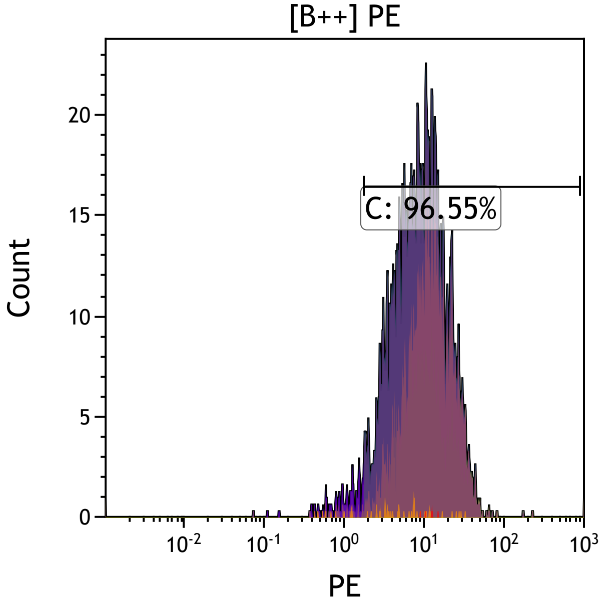

Gate	X-Med
All	8.88
C	9.23

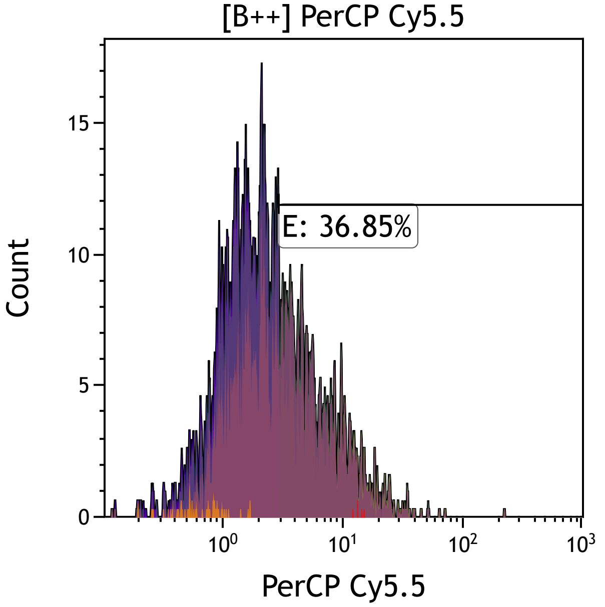

Gate	X-Med
All	2.19
E	5.41

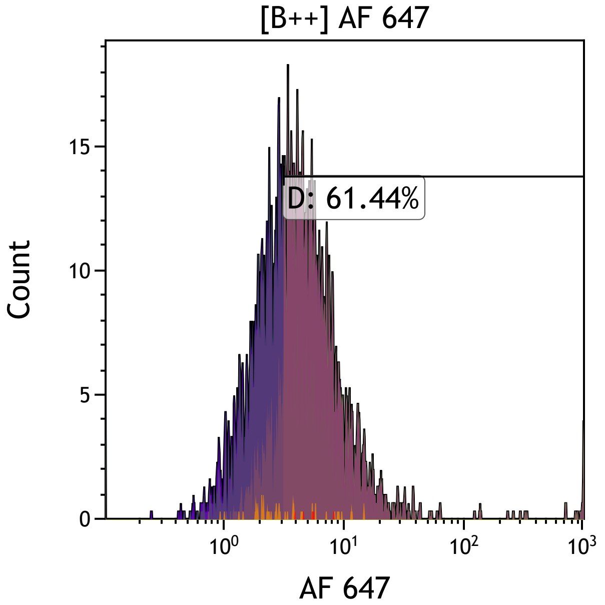

Gate	X-Med
All	3.87
D	5.50

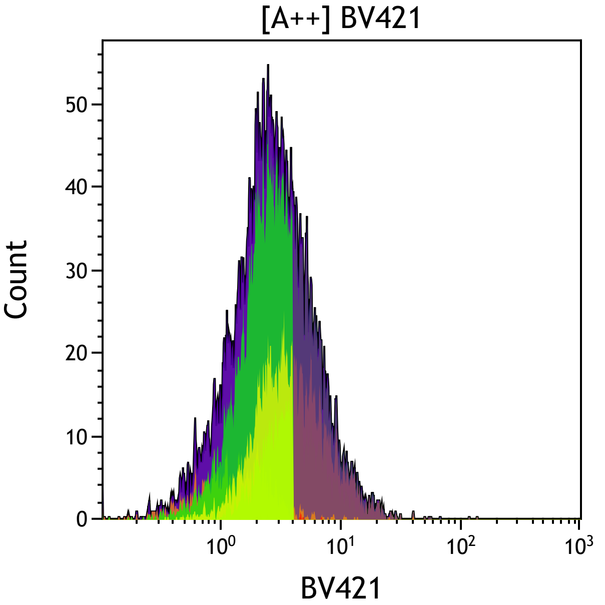

1 ipsi-2017-03-14-155101-1
Rat microglia M1M2 protocol - Imported

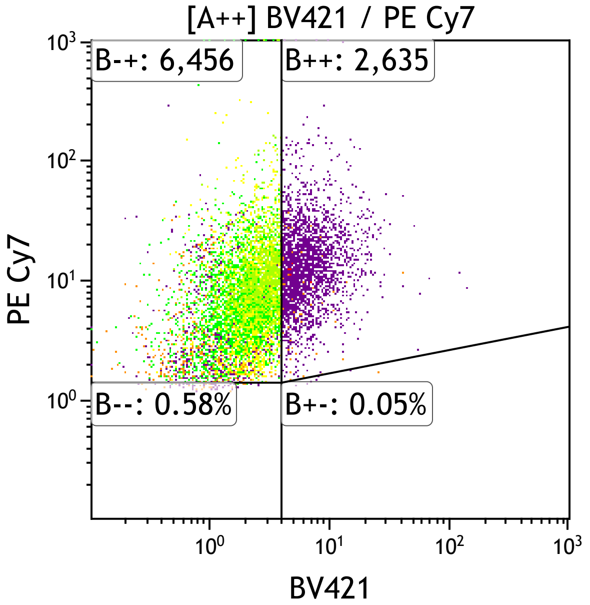

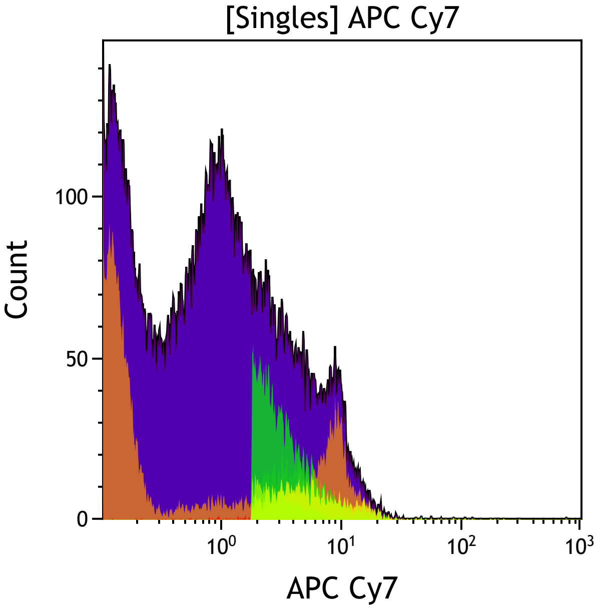

Gate	X-Med	Y-Med
All	2.68	8.04
B--	0.92	1.32
B-+	2.10	6.45
B+-	6.99	1.43
B++	5.89	12.68

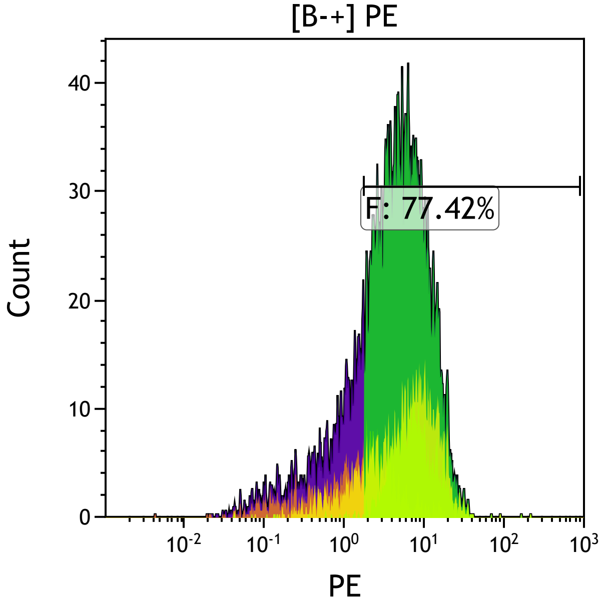

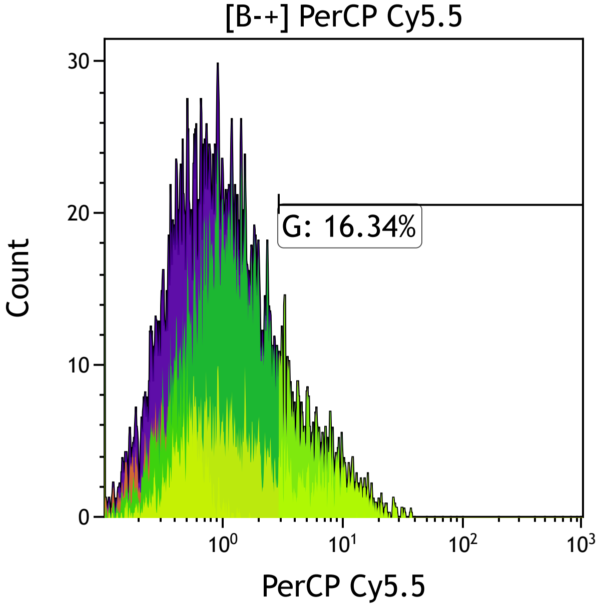

Gate	X-Med
All	4.27
F	5.66

Gate	X-Med
All	0.93
G	5.26

[B-+] AF 647

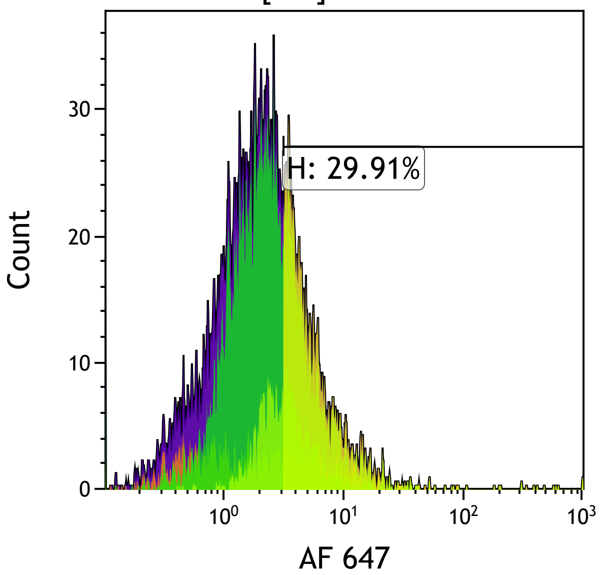

Gate	X-Med
All	2.07
H	4.82

2 ipsi-2017-03-14-155353-2
Rat microglia M1M2 protocol - Imported

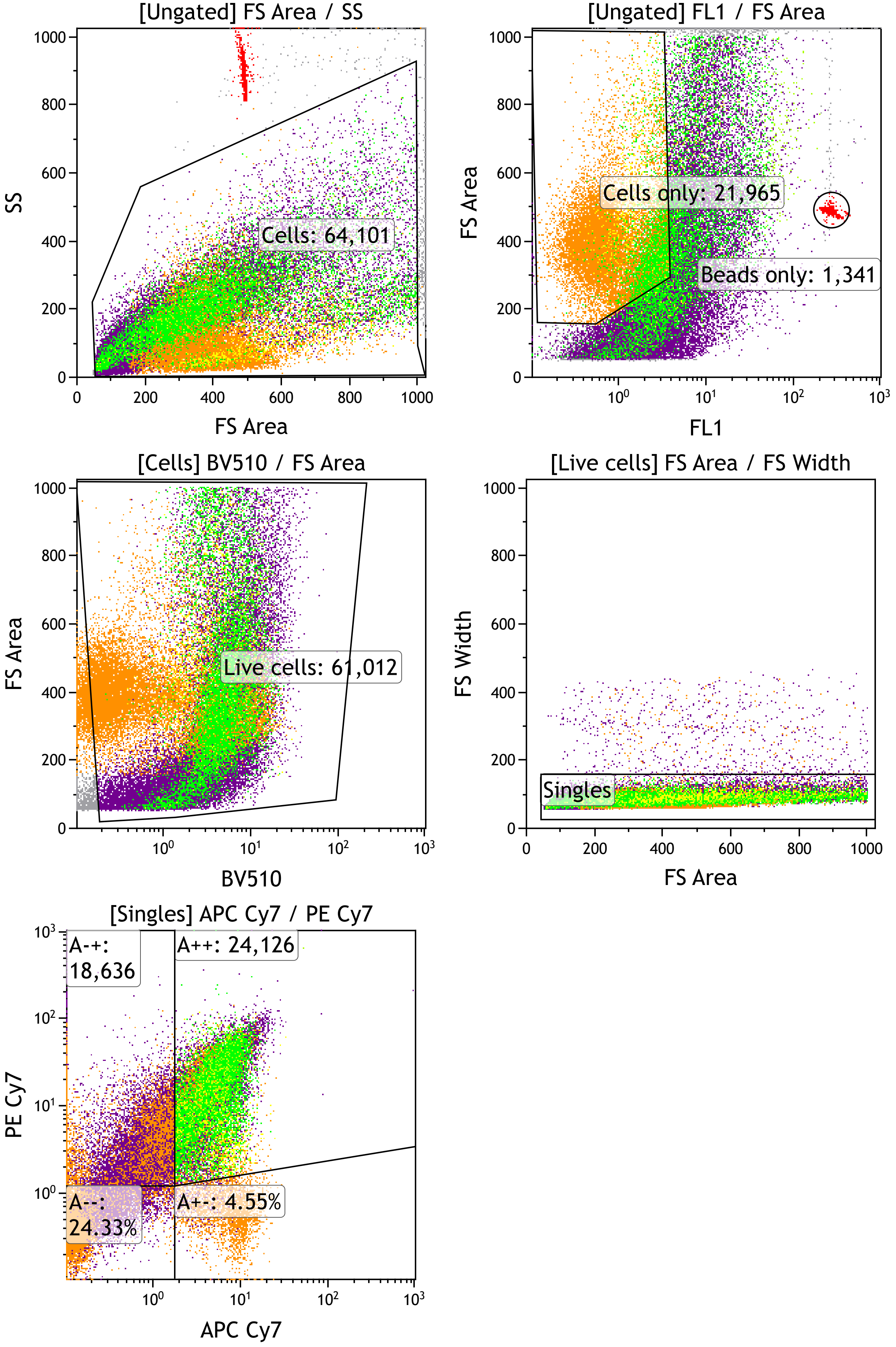

[B++] PE

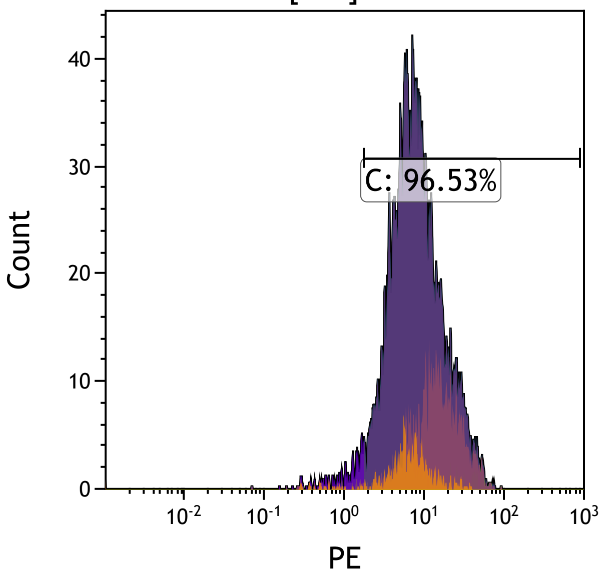

Gate	X-Med
All	7.55
C	7.79

[B++] PerCP Cy5.5

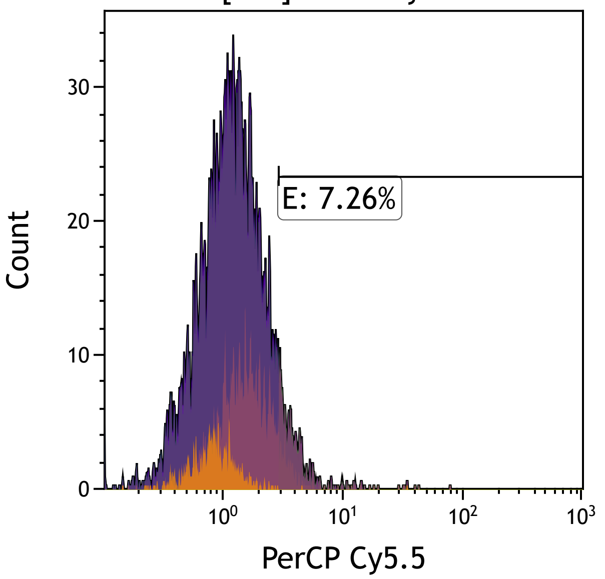

Gate	X-Med
All	1.20
E	3.70

[B++] AF 647

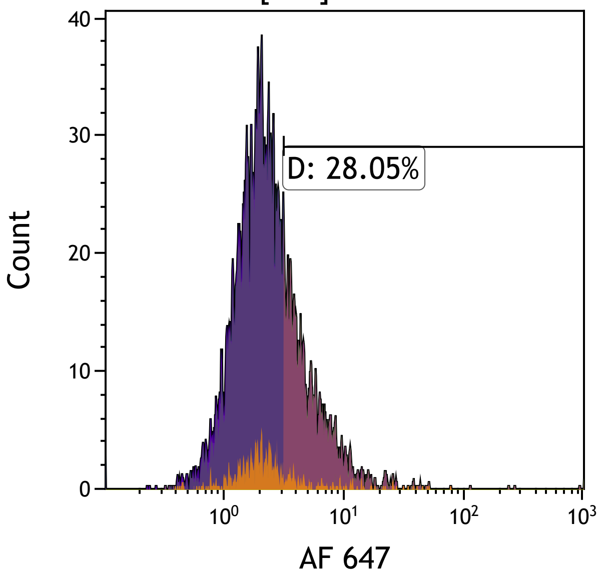

Gate	X-Med
All	2.20
D	4.71

[A++] BV421

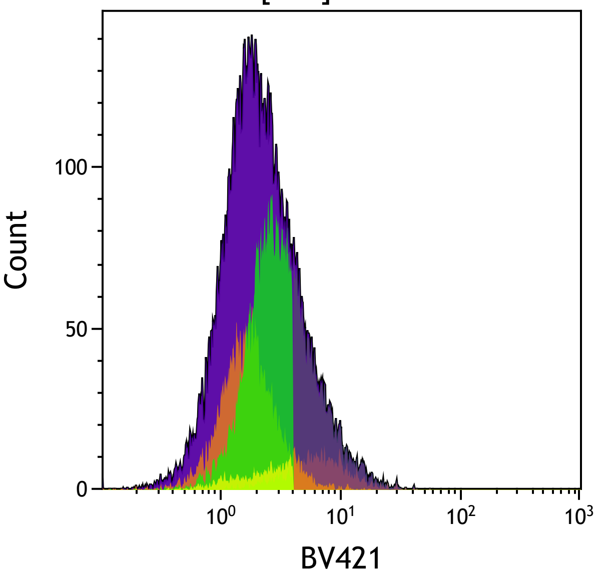

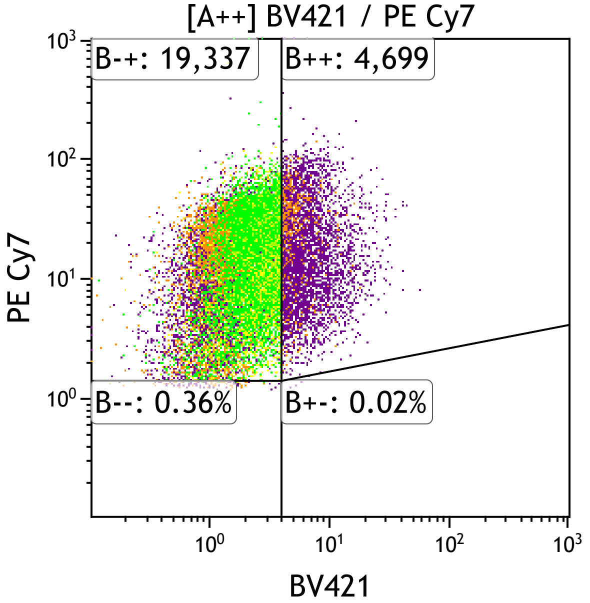

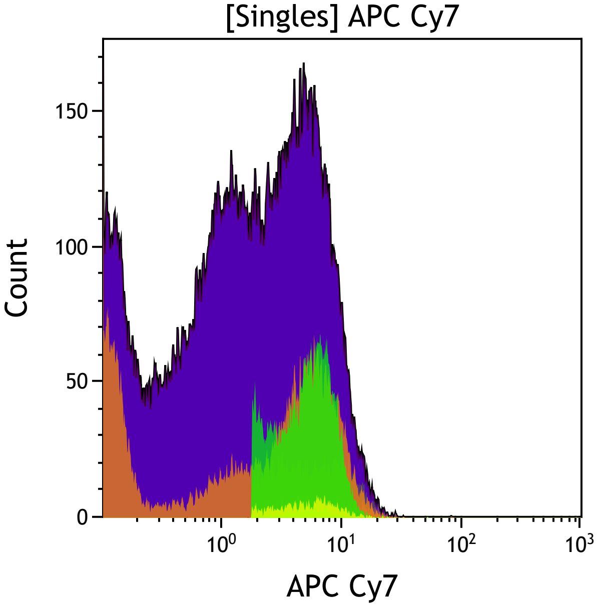

Gate	X-Med	Y-Med
All	2.07	14.35
B--	0.95	1.33
B-+	1.77	14.04
B+-	5.86	1.37
B++	5.80	15.99

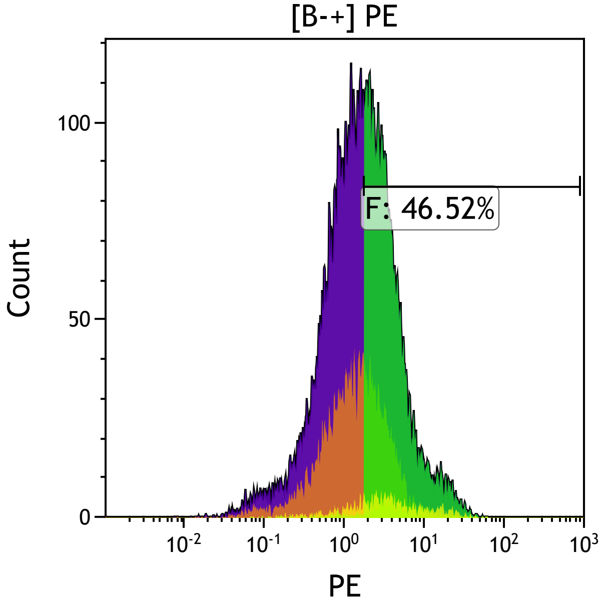

Gate	X-Med
All	1.62
F	3.22

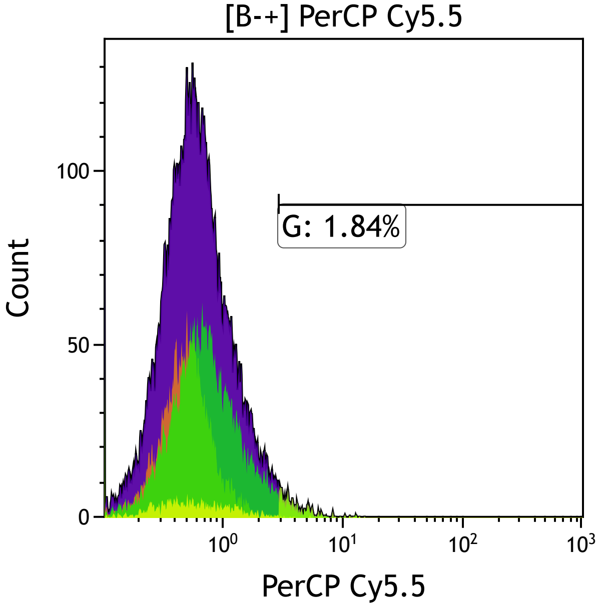

Gate	X-Med
All	0.58
G	3.77

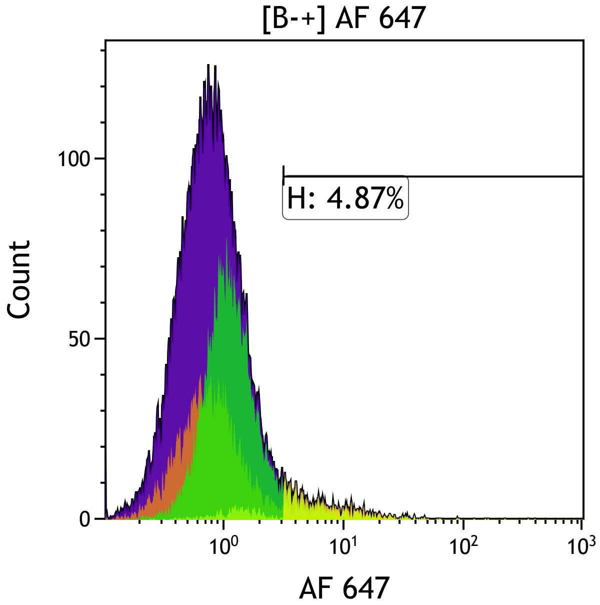

Gate	X-Med
All	0.79
H	5.91

1 ipsi-2017-03-14-155101-1
Rat microglia M1M2 protocol - Imported

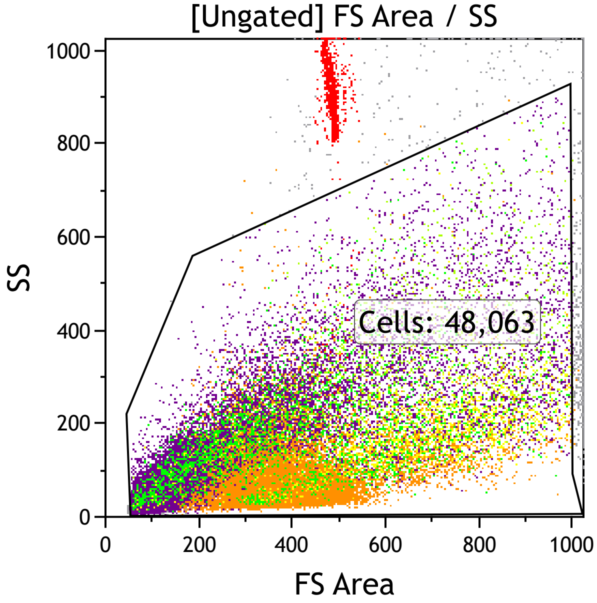

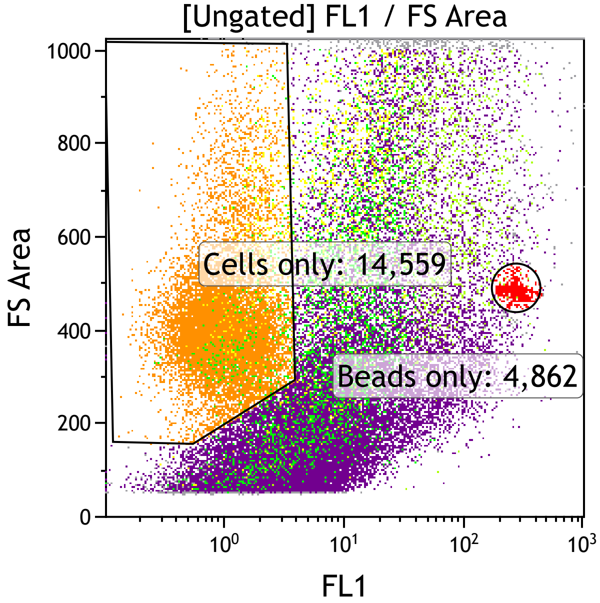

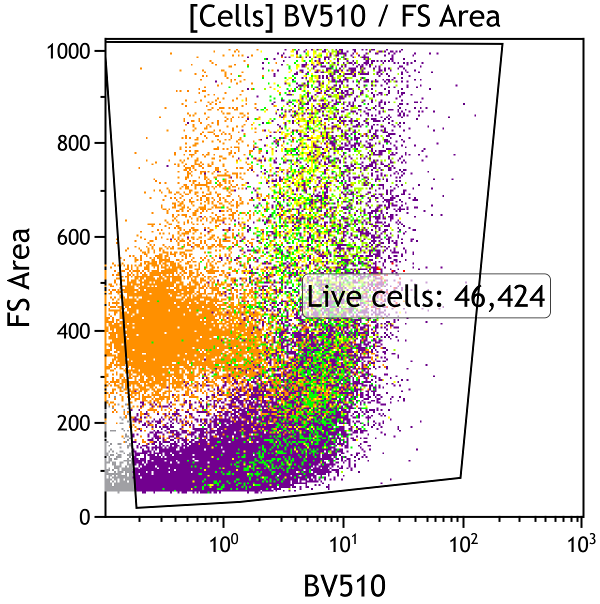

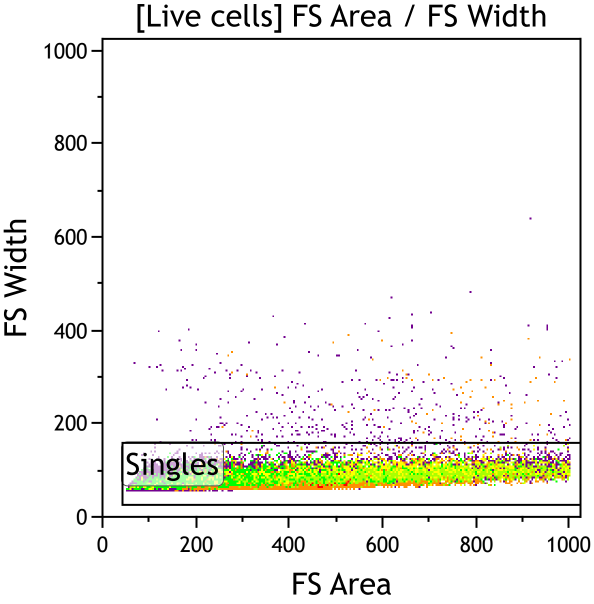

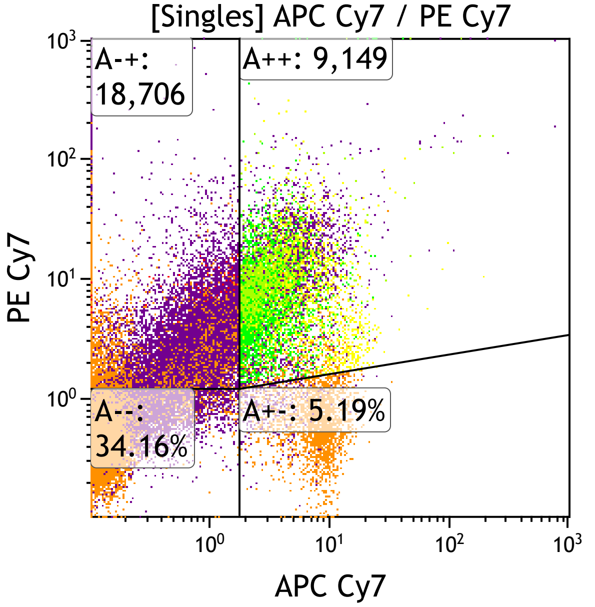

[B++] PE

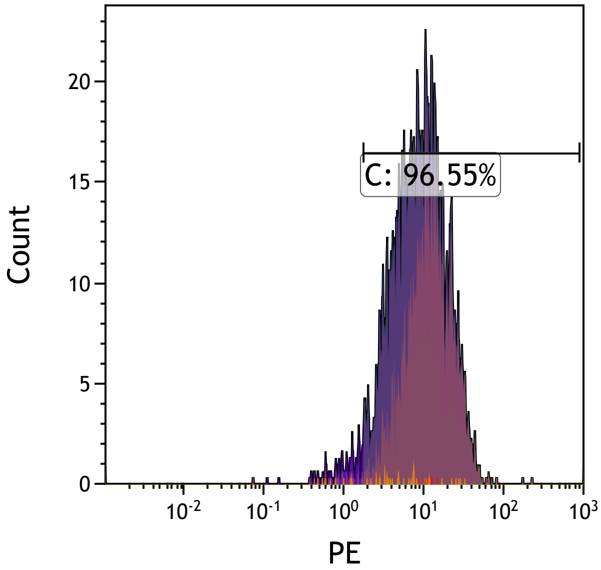

Gate	X-Med
All	8.88
C	9.23

[B++] PerCP Cy5.5

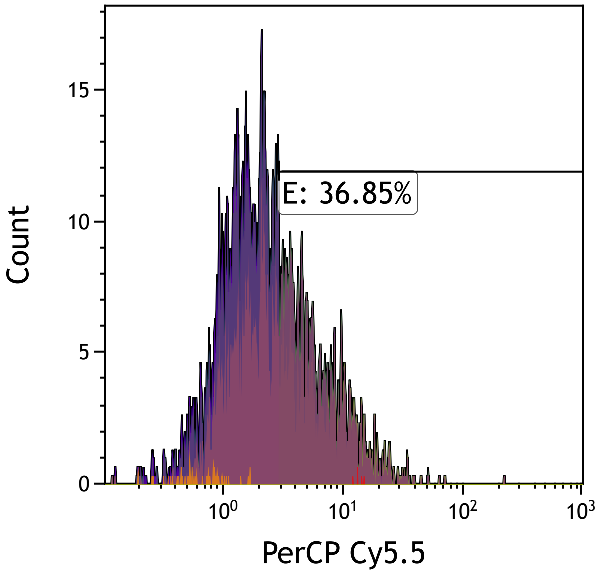

Gate	X-Med
All	2.19
E	5.41

[B++] AF 647

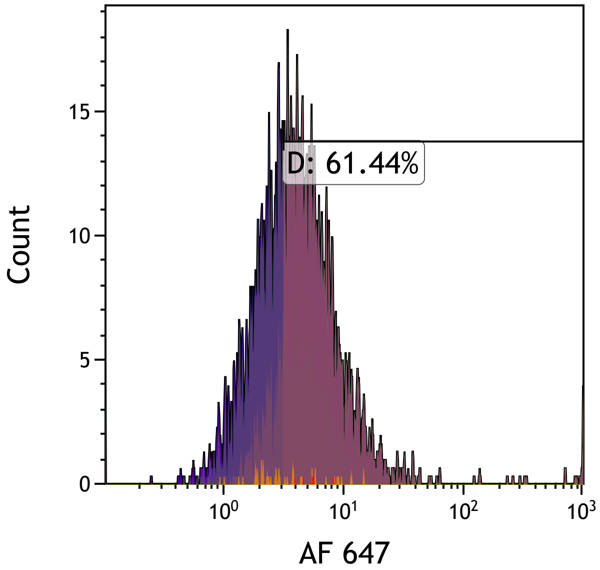

Gate	X-Med
All	3.87
D	5.50

[A++] BV421

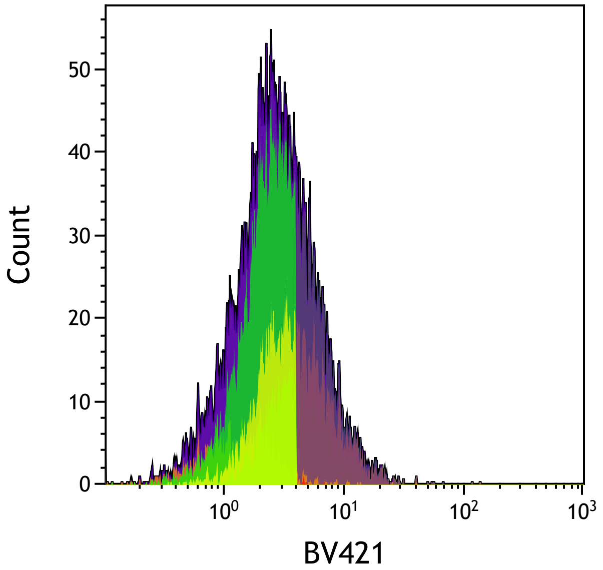

[A++] BV421 / PE Cy7

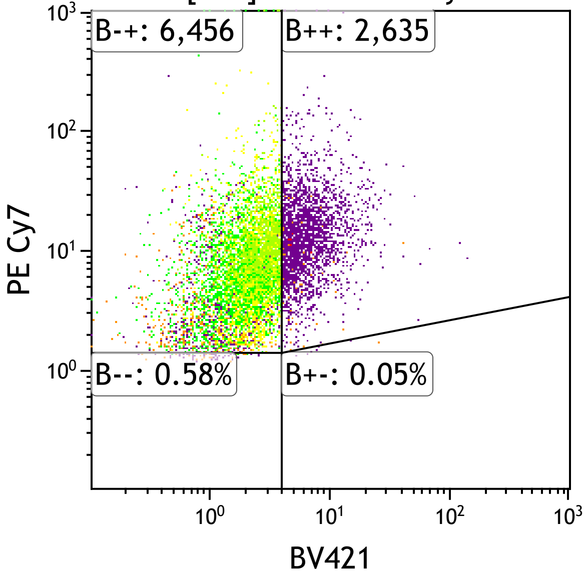

[Singles] APC Cy7

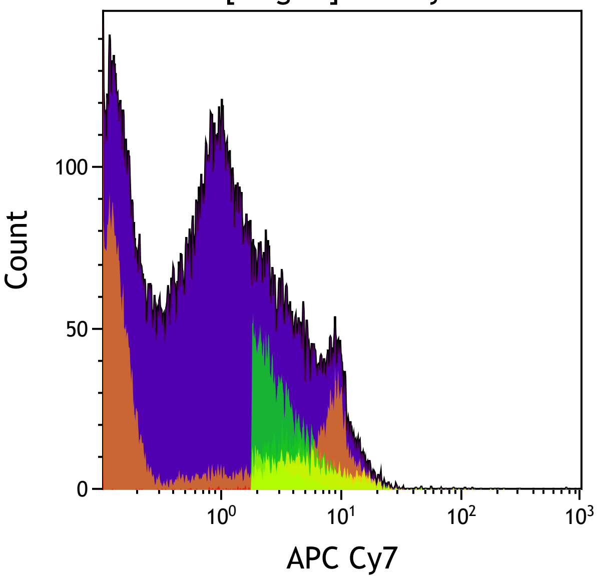

Gate	X-Med	Y-Med
All	2.68	8.04
B--	0.92	1.32
B-+	2.10	6.45
B+-	6.99	1.43
B++	5.89	12.68

[B-+] PE

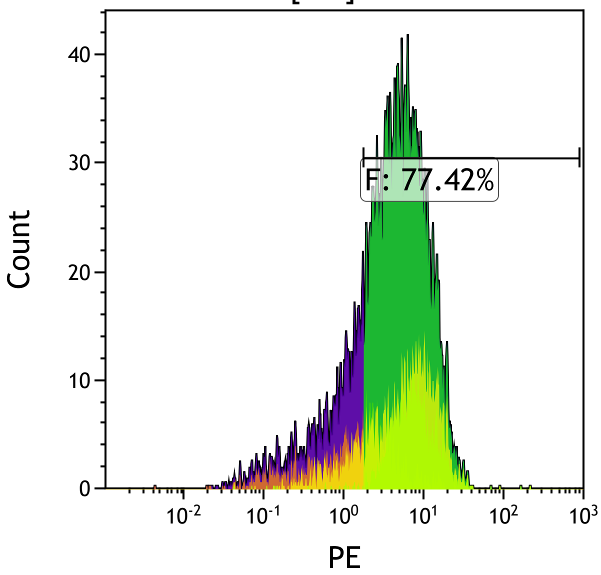

[B-+] PerCP Cy5.5

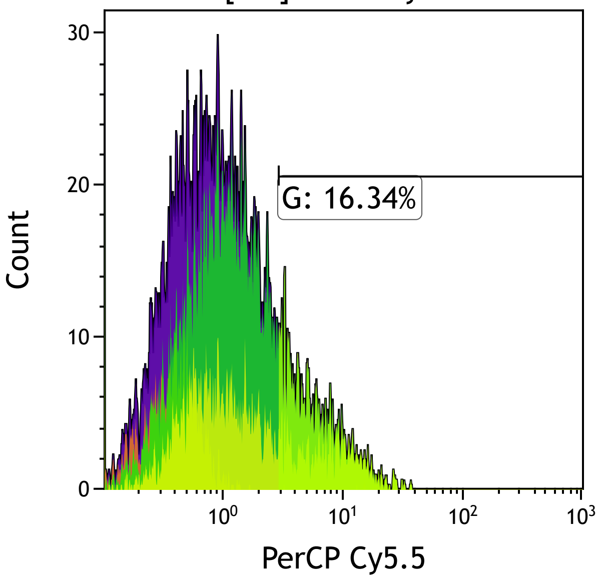

Gate	X-Med
All	4.27
F	5.66

Gate	X-Med
All	0.93
G	5.26

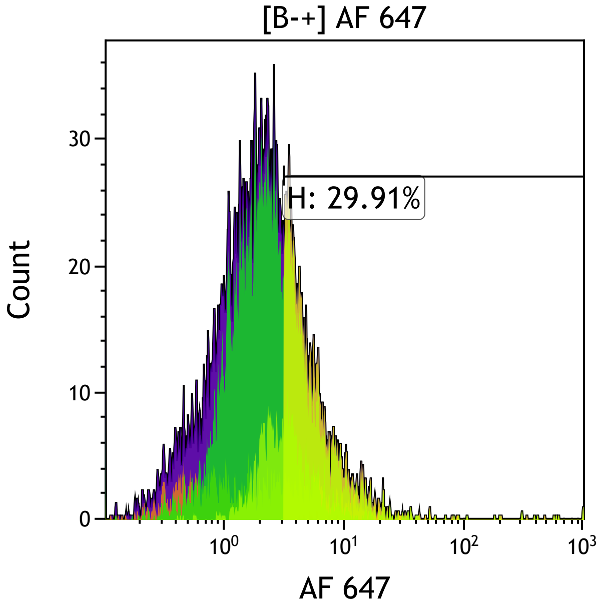

Gate	X-Med
All	2.07
H	4.82

3 ipsi-2017-03-14-155459-3
Rat microglia M1M2 protocol - Imported

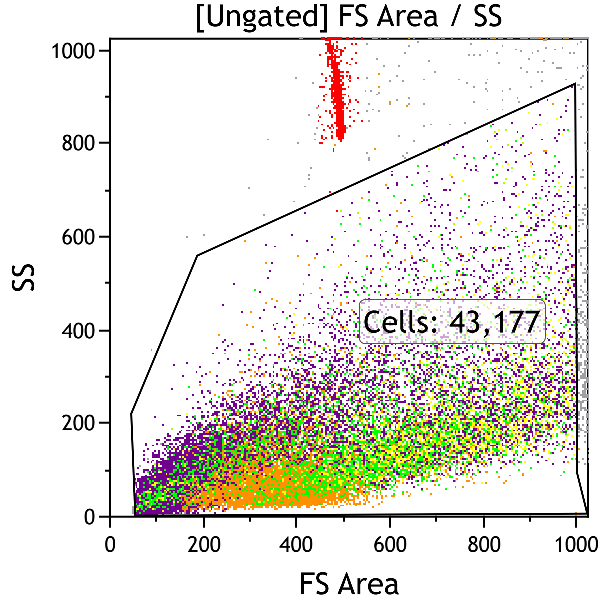

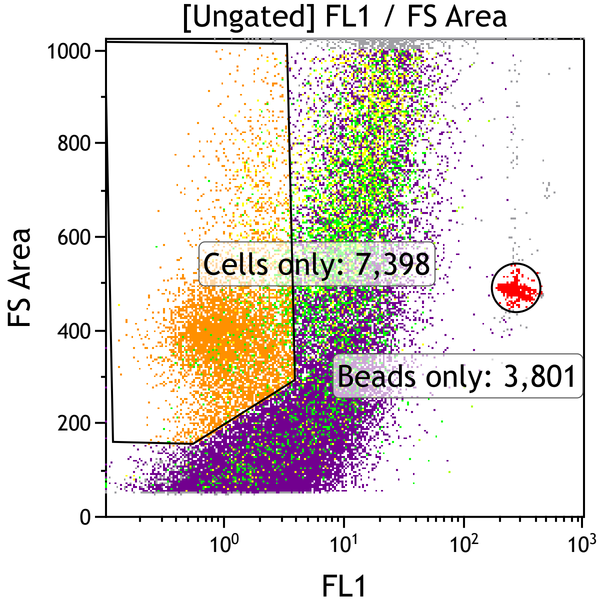

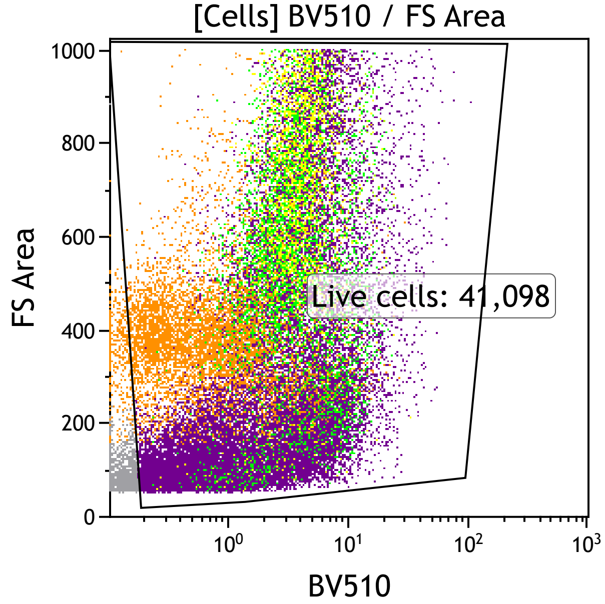

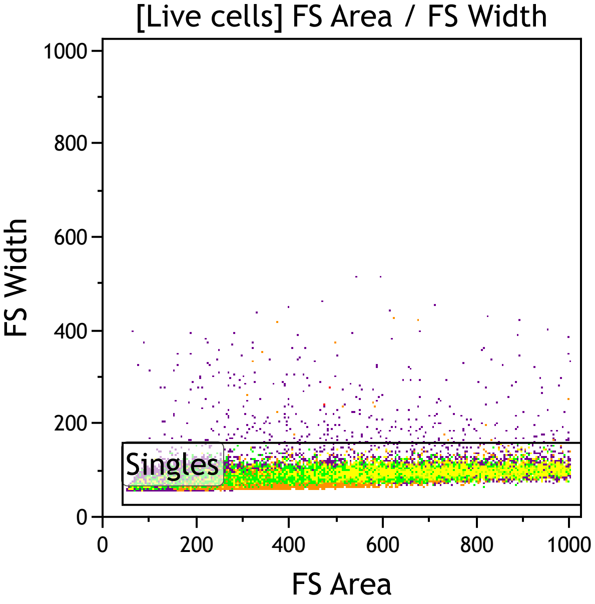

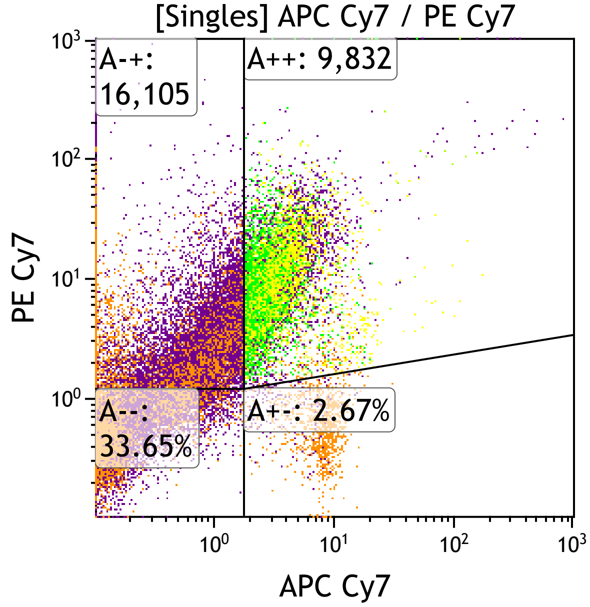

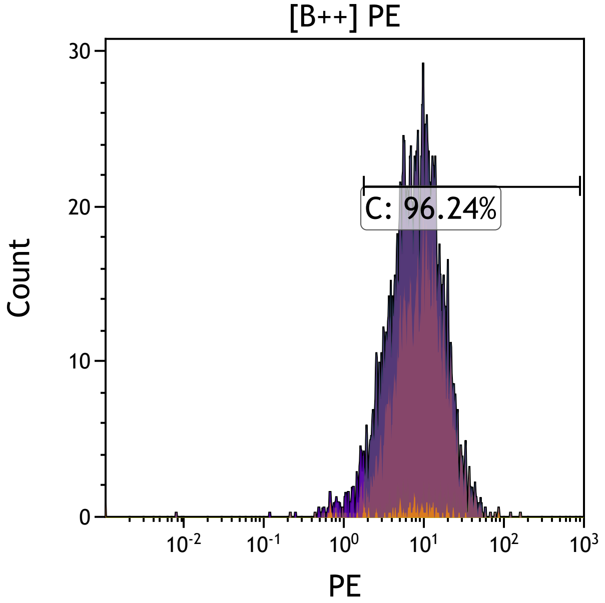

Gate	X-Med
All	8.09
C	8.39

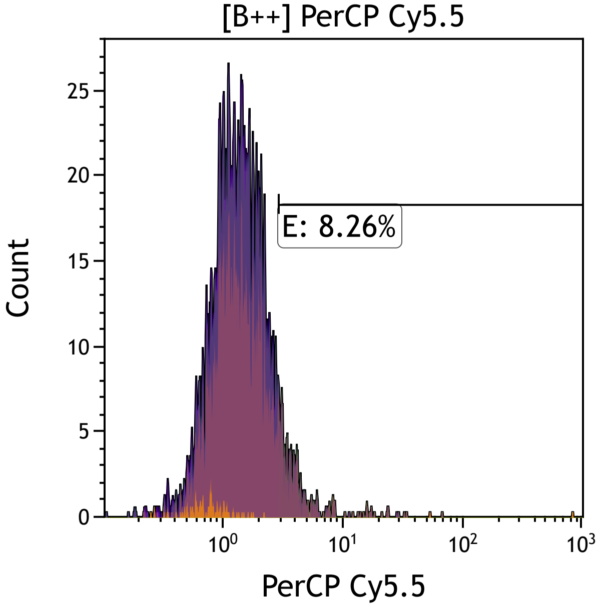

Gate	X-Med
All	1.37
E	3.77

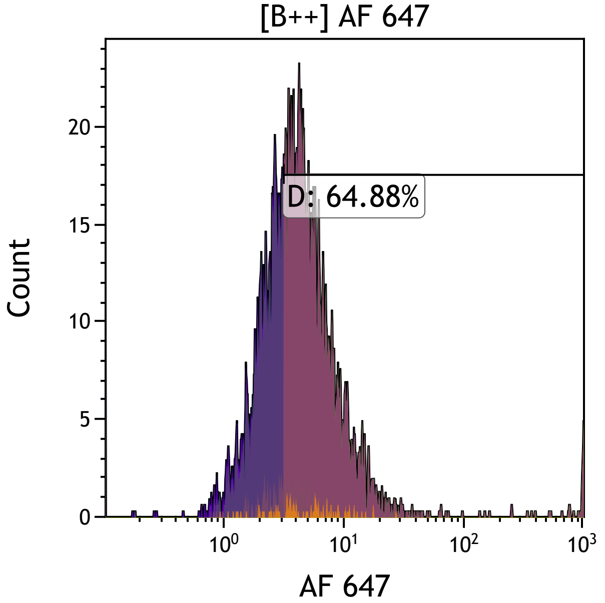

Gate	X-Med
All	3.96
D	5.22

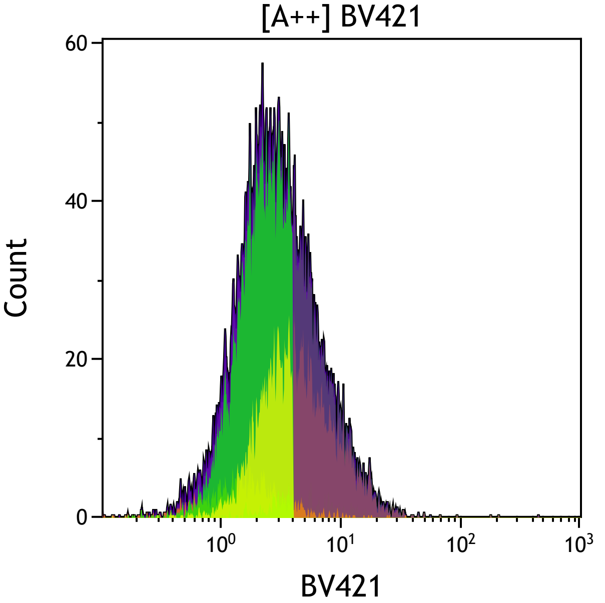

3 ipsi-2017-03-14-155459-3
Rat microglia M1M2 protocol - Imported

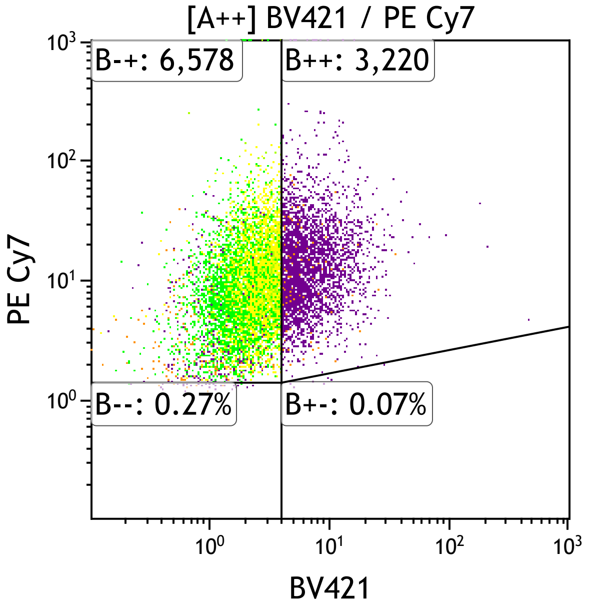

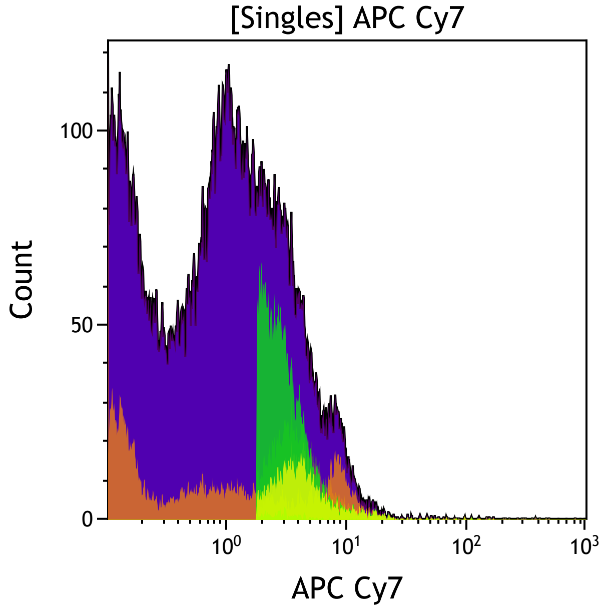

Gate	X-Med	Y-Med
All	2.85	9.31
B--	0.97	1.33
B-+	2.12	7.99
B+-	8.91	1.42
B++	6.22	12.46

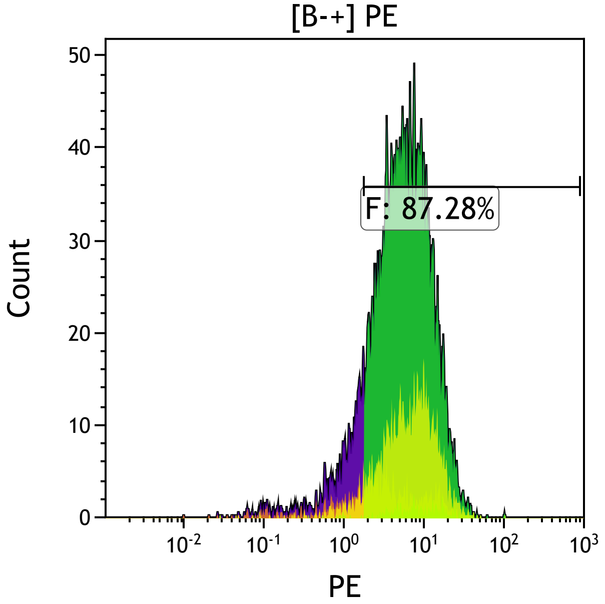

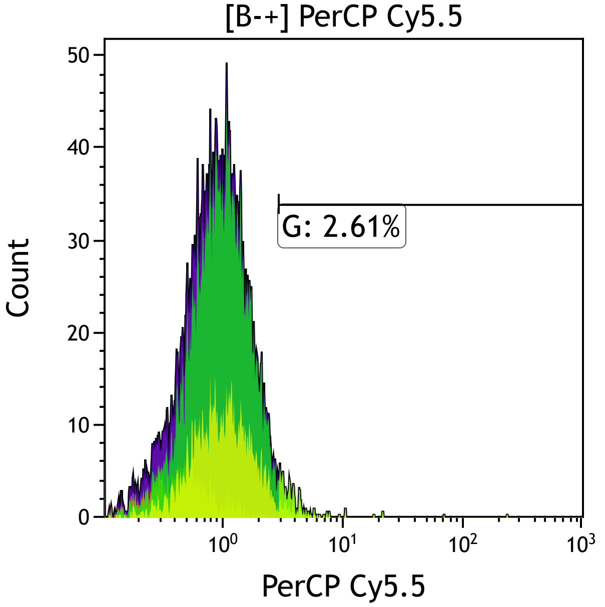

Gate	X-Med
All	5.33
F	6.12

Gate	X-Med
All	0.90
G	3.61

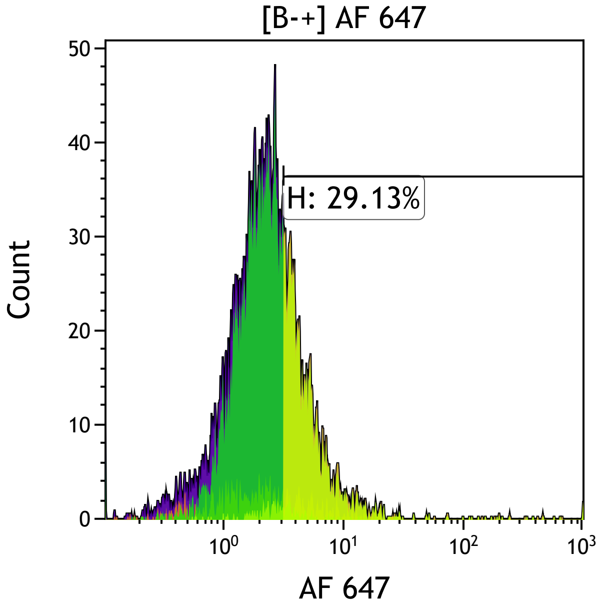

Gate	X-Med
All	2.25
H	4.49

4 ipsi-2017-03-14-155734-4

Rat microglia M1M2 protocol - Imported

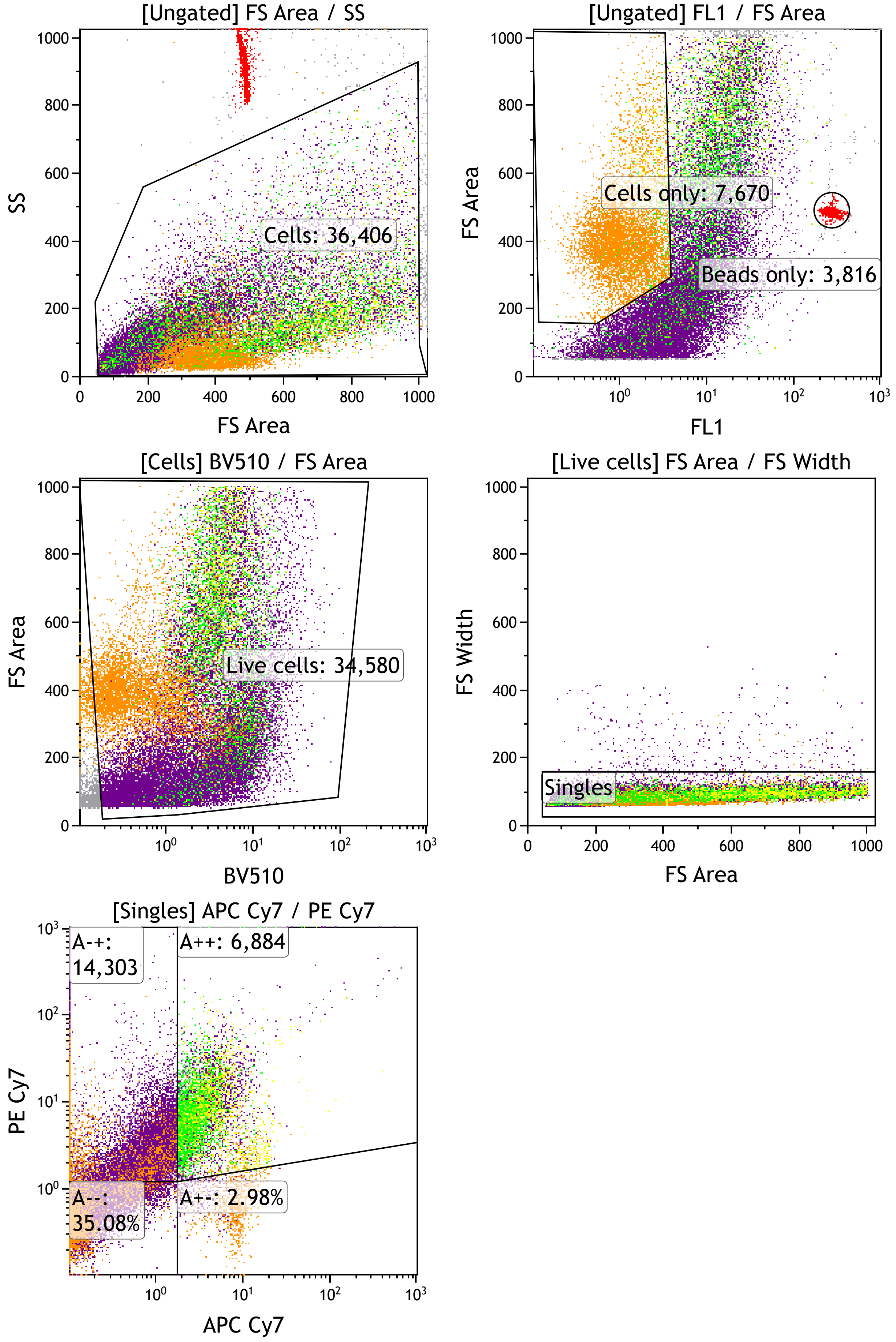

[B++] PE

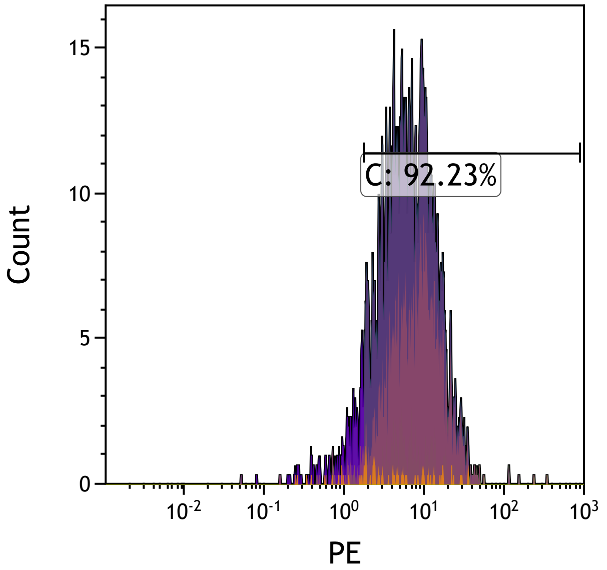

Gate	X-Med
All	6.15
C	6.70

[B++] PerCP Cy5.5

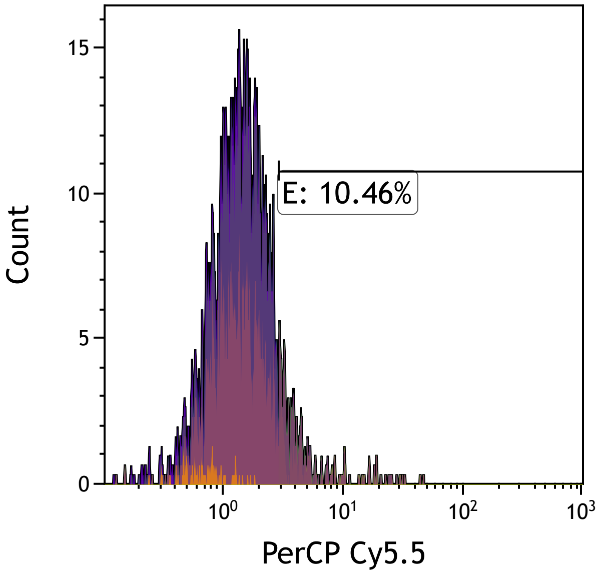

Gate	X-Med
All	1.43
E	3.91

[B++] AF 647

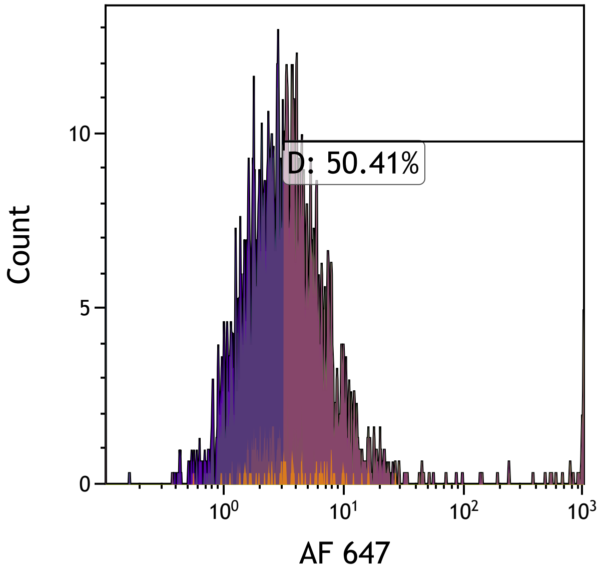

Gate	X-Med
All	3.19
D	5.29

[A++] BV421

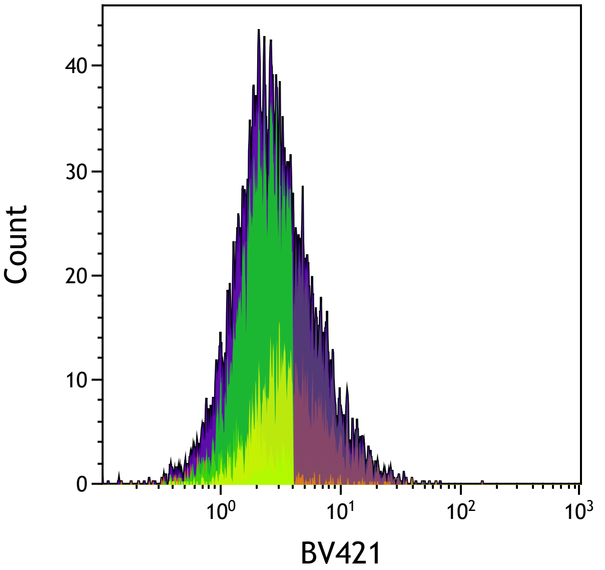

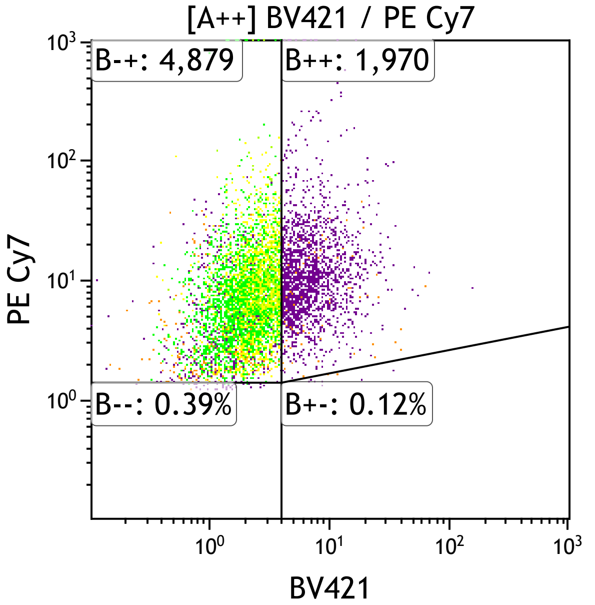

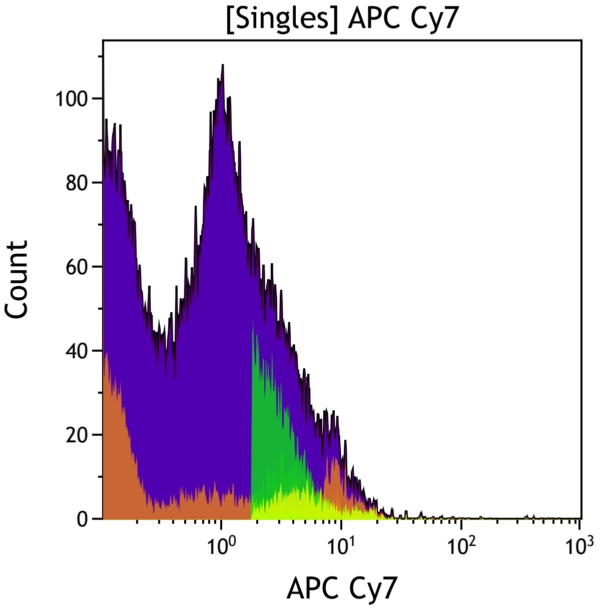

Gate	X-Med	Y-Med
All	2.65	7.40
B--	1.31	1.31
B-+	2.08	6.41
B+-	8.71	1.47
B++	6.22	10.45

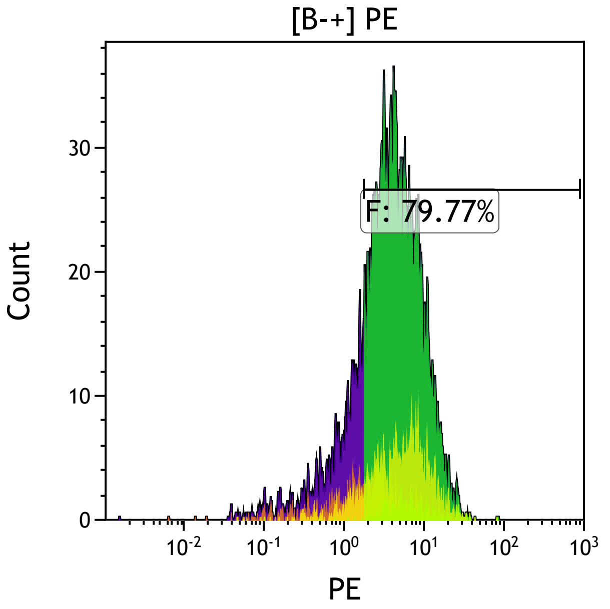

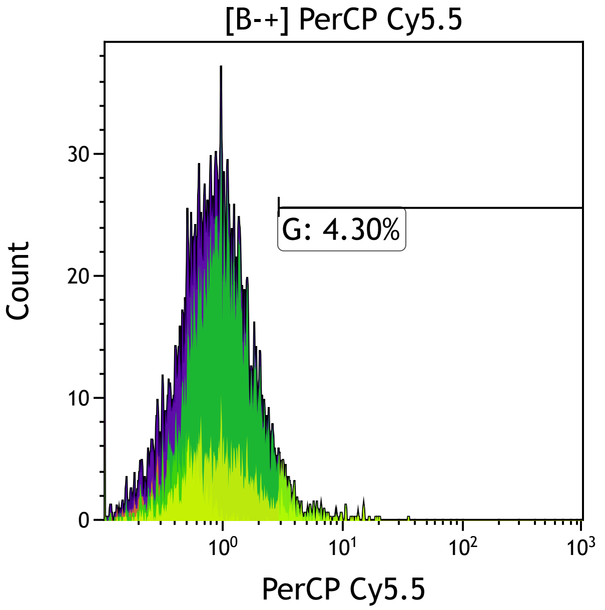

Gate	X-Med
All	3.84
F	4.71

Gate	X-Med
All	0.86
G	3.72

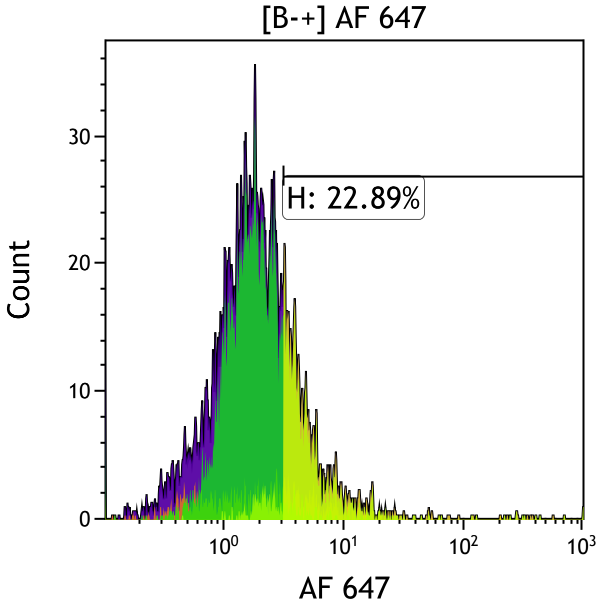

Gate	X-Med
All	1.83
H	4.53

5 ipsi-2017-03-14-160007-5
Rat microglia M1M2 protocol - Imported

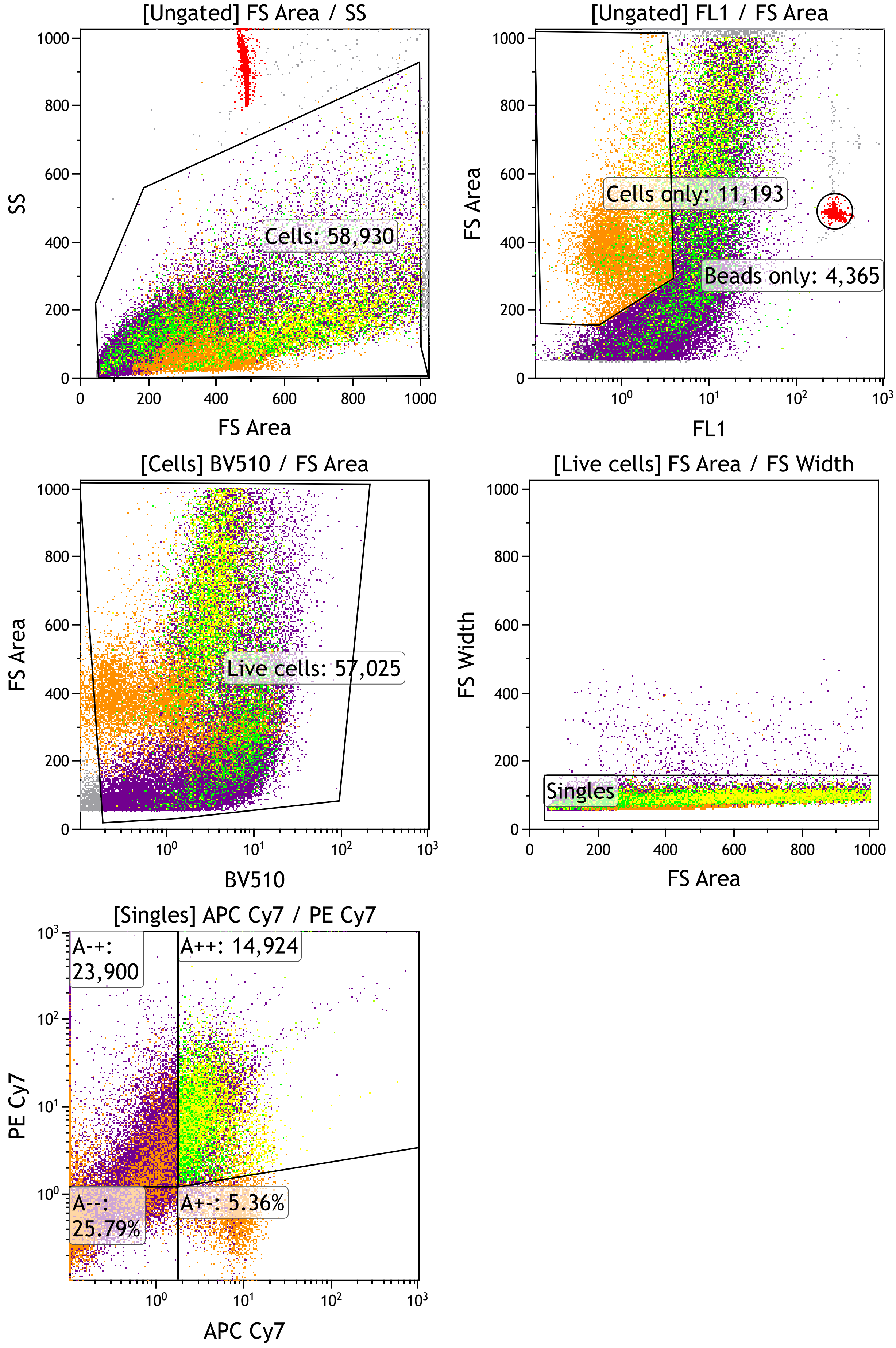

5 ipsi-2017-03-14-160007-5

Rat microglia M1M2 protocol - Imported

[B++] PE

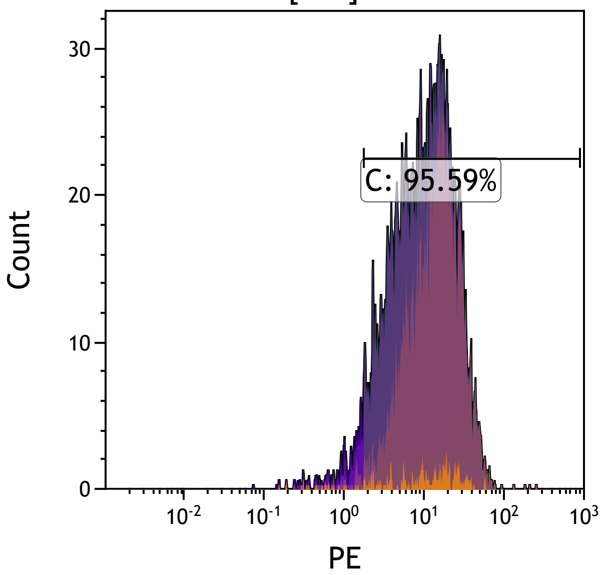

Gate	X-Med
All	10.20
C	10.87

[B++] PerCP Cy5.5

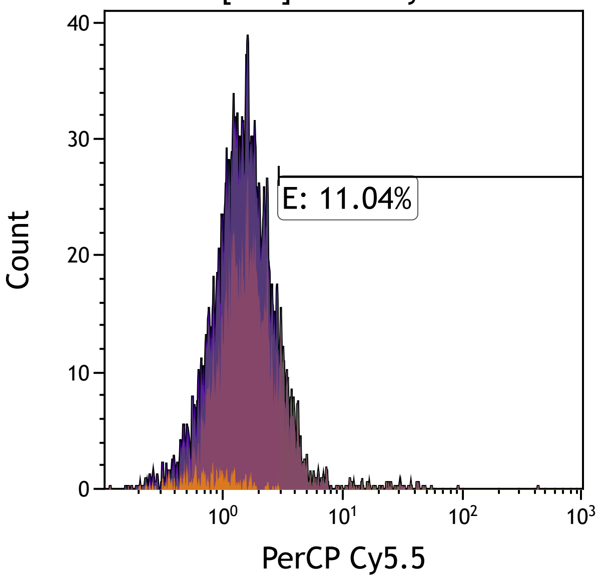

Gate	X-Med
All	1.49
E	3.68

[B++] AF 647

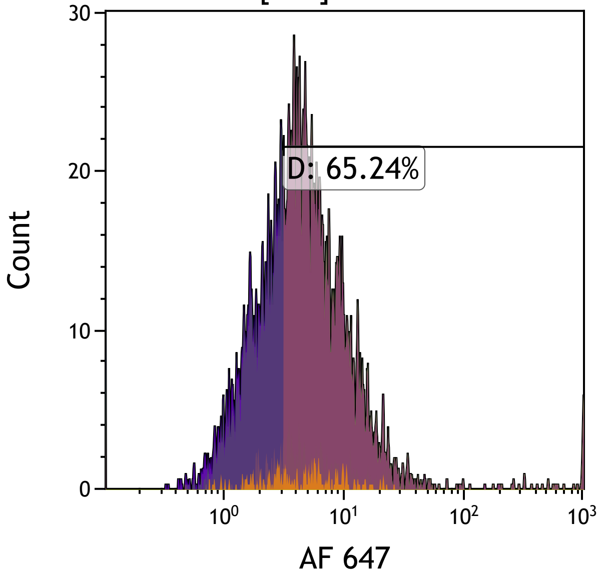

Gate	X-Med
All	4.22
D	5.96

[A++] BV421

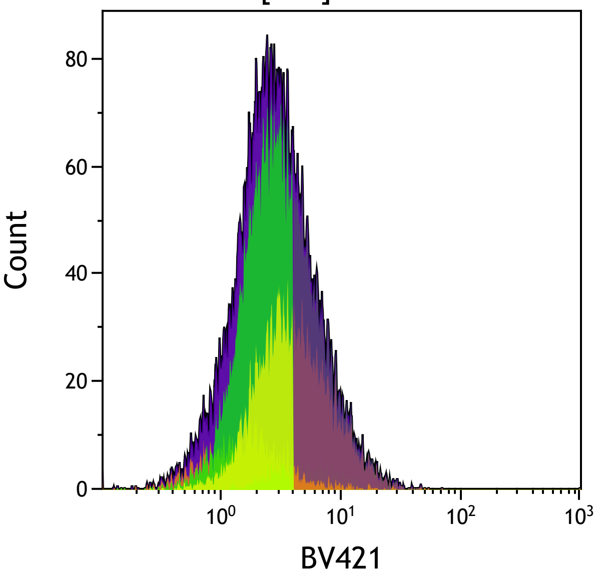

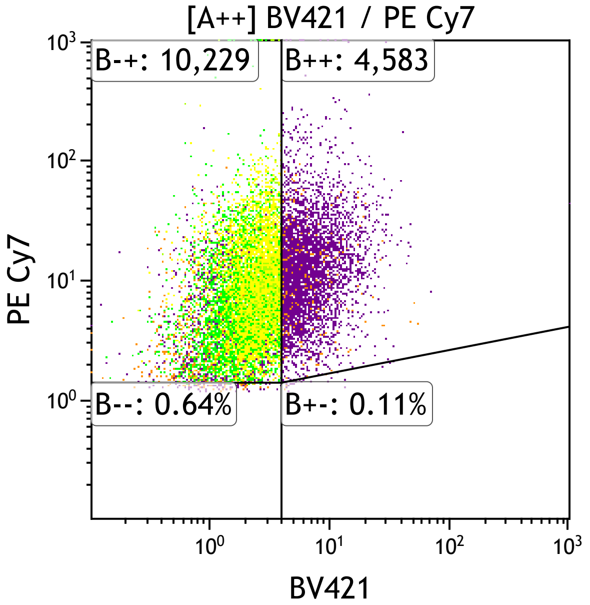

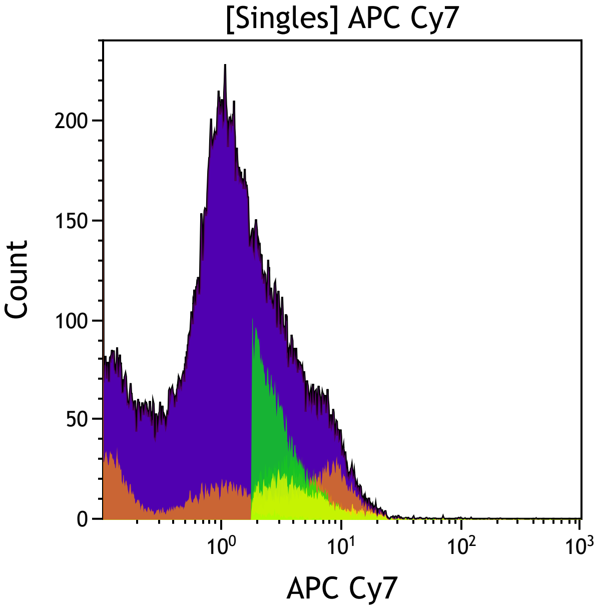

Gate	X-Med	Y-Med
All	2.78	7.67
B--	1.20	1.32
B-+	2.12	6.32
B+-	5.01	1.41
B++	6.10	11.86

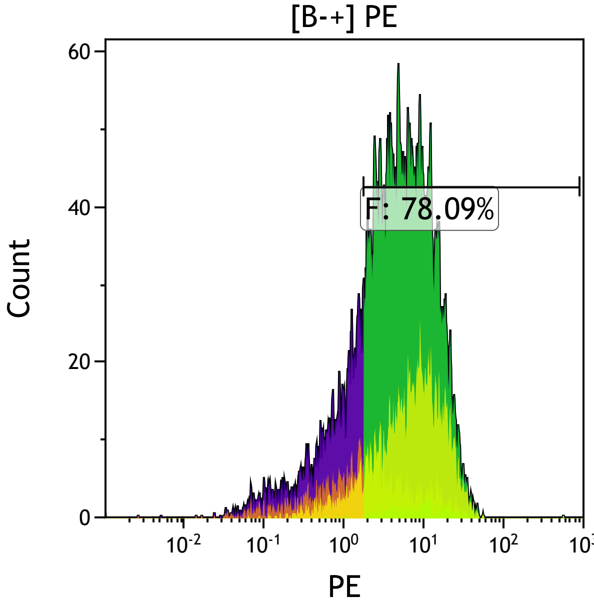

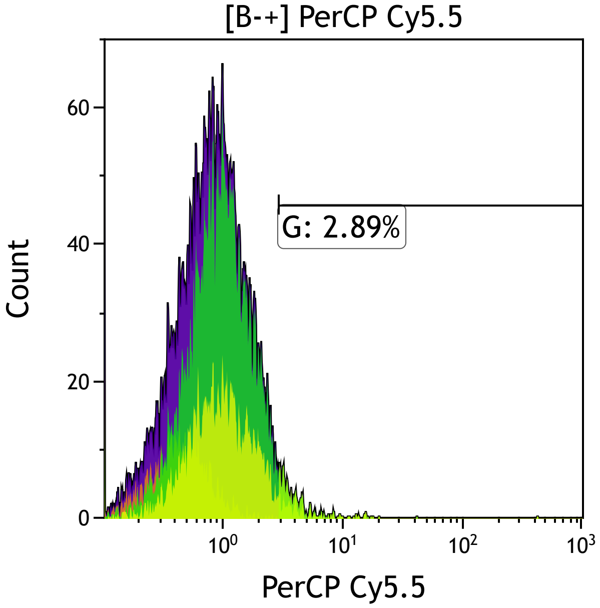

Gate	X-Med
All	4.54
F	6.26

Gate	X-Med
All	0.83
G	3.71

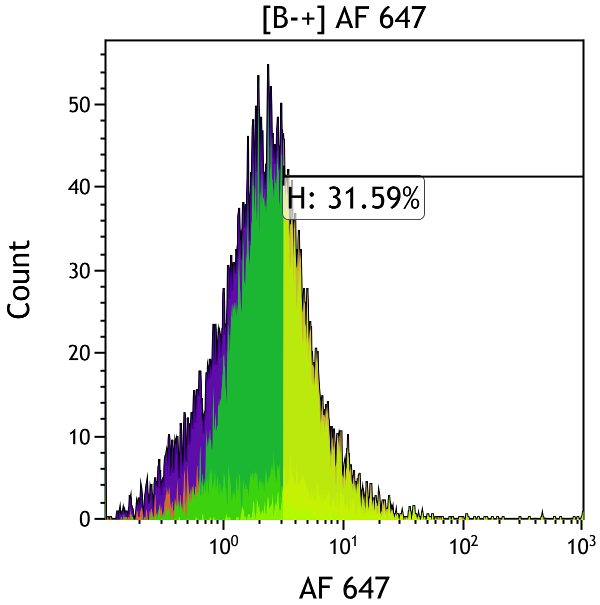

Gate	X-Med
All	2.18
H	4.79

6 ipsi-2017-03-14-160240-6

Rat microglia M1M2 protocol - Imported

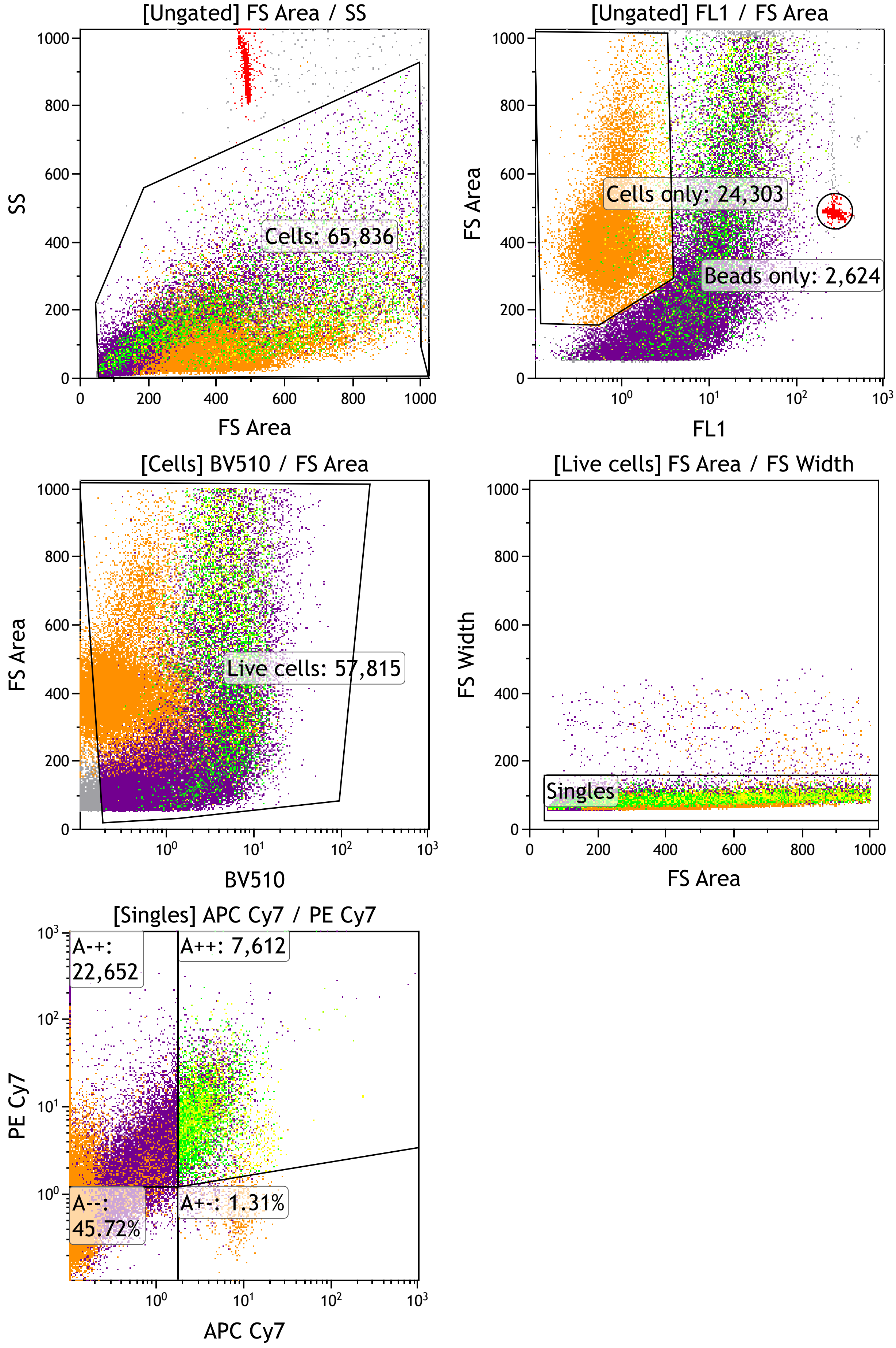

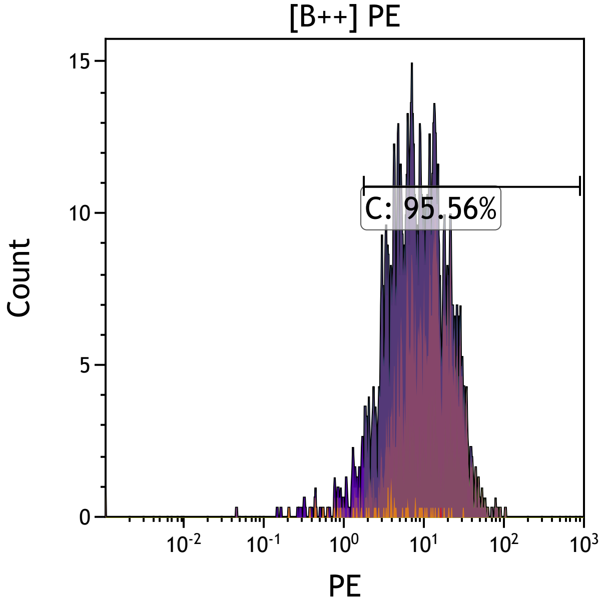

Gate	X-Med
All	8.26
C	8.79

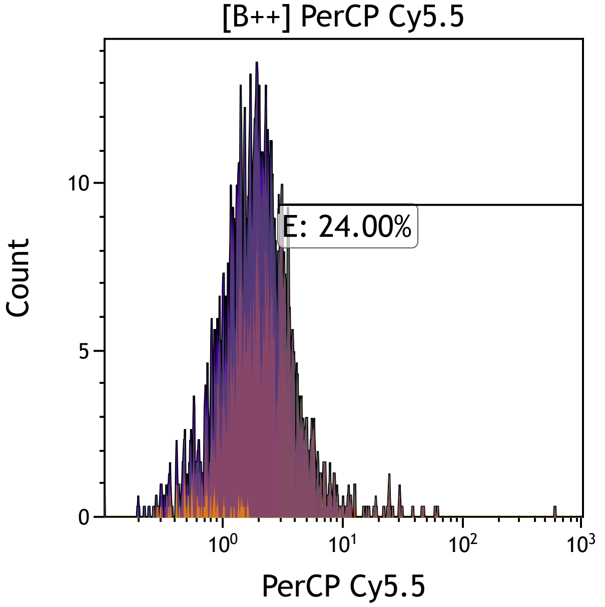

Gate	X-Med
All	1.90
E	3.86

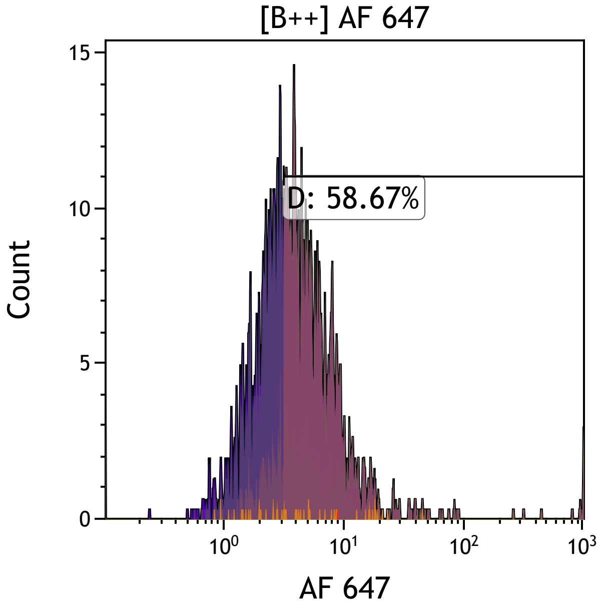

Gate	X-Med
All	3.63
D	5.29

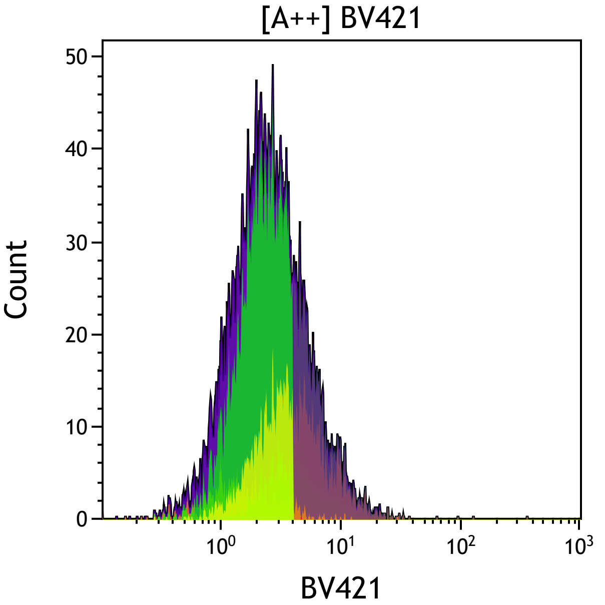

6 ipsi-2017-03-14-160240-6
Rat microglia M1M2 protocol - Imported

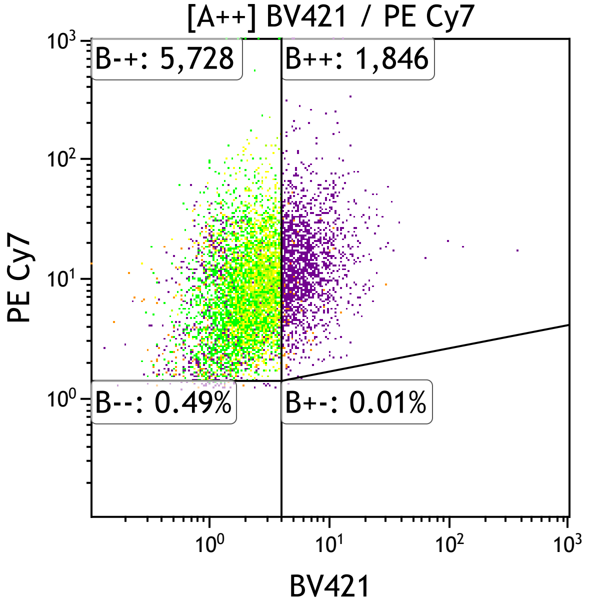

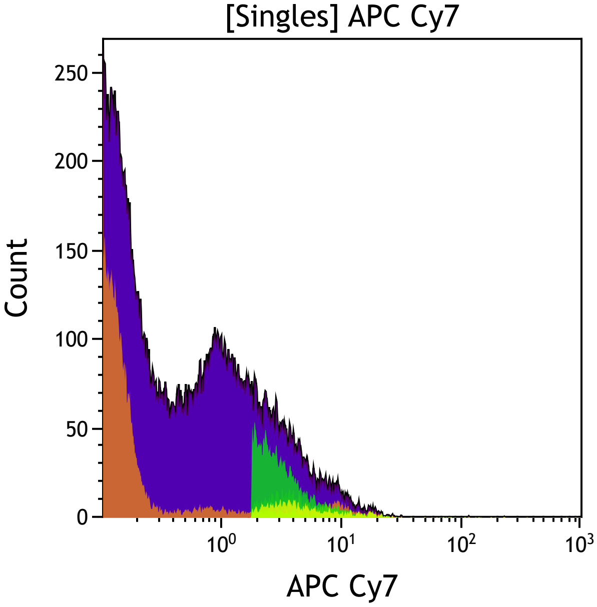

Gate	X-Med	Y-Med
All	2.48	8.06
B--	1.15	1.31
B-+	2.02	6.95
B+-	4.58	1.34
B++	5.74	12.45

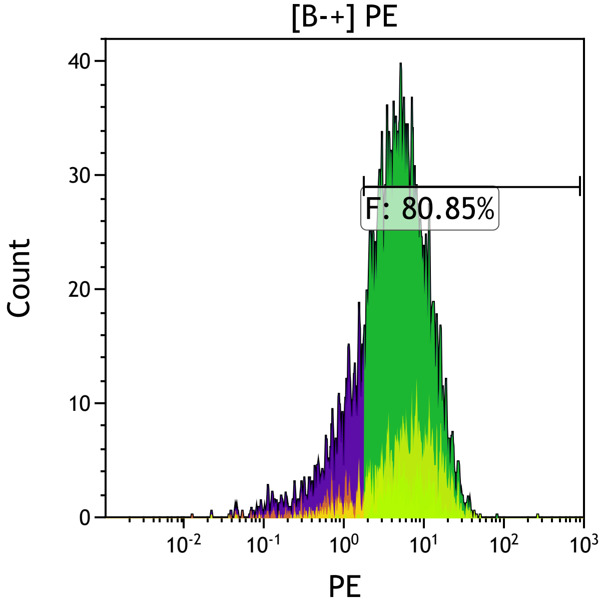

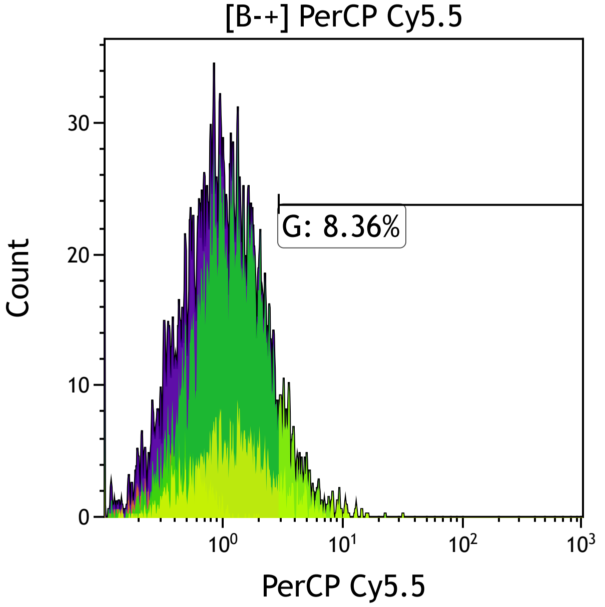

Gate	X-Med
All	4.37
F	5.42

Gate	X-Med
All	0.98
G	3.87

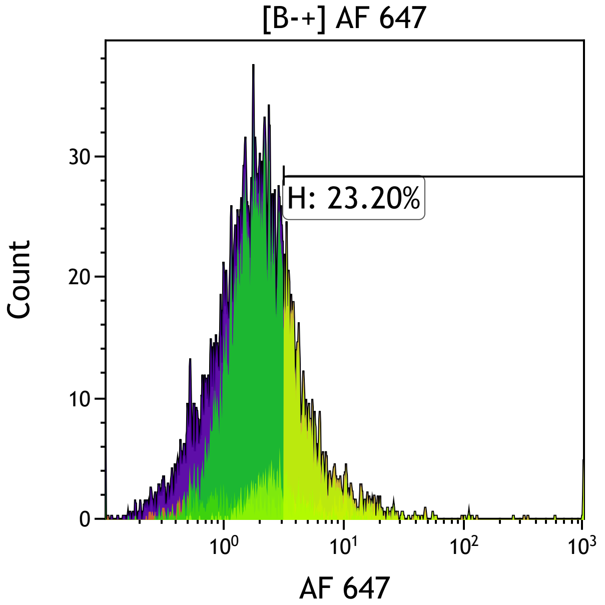

Gate	X-Med
All	1.86
H	4.63

7 ipsi-2017-03-14-160411-7

Rat microglia M1M2 protocol - Imported

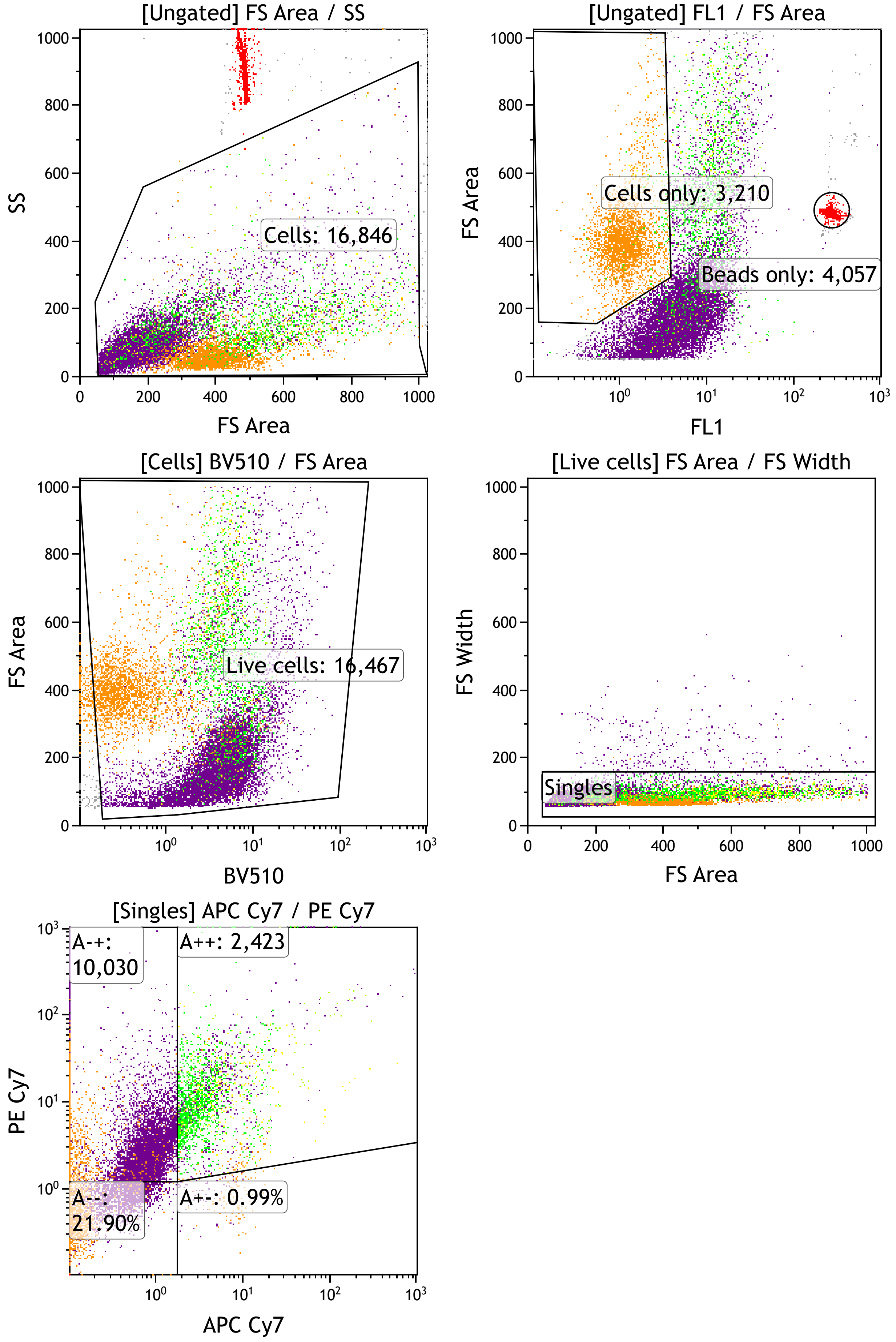

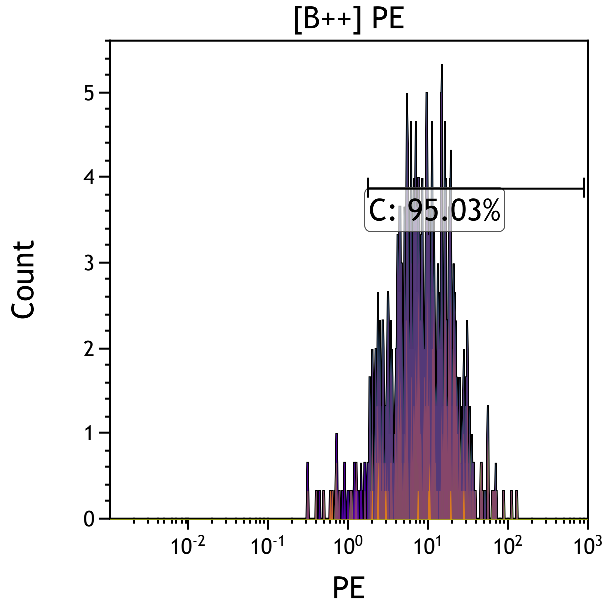

Gate	X-Med
All	7.99
C	8.51

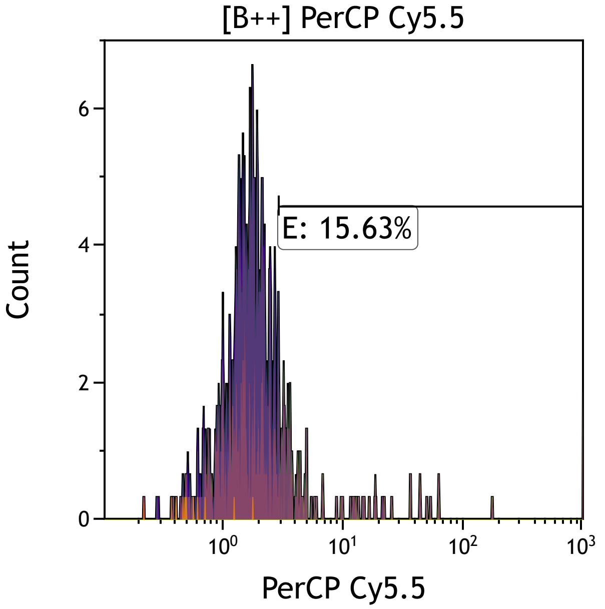

Gate	X-Med
All	1.75
E	4.24

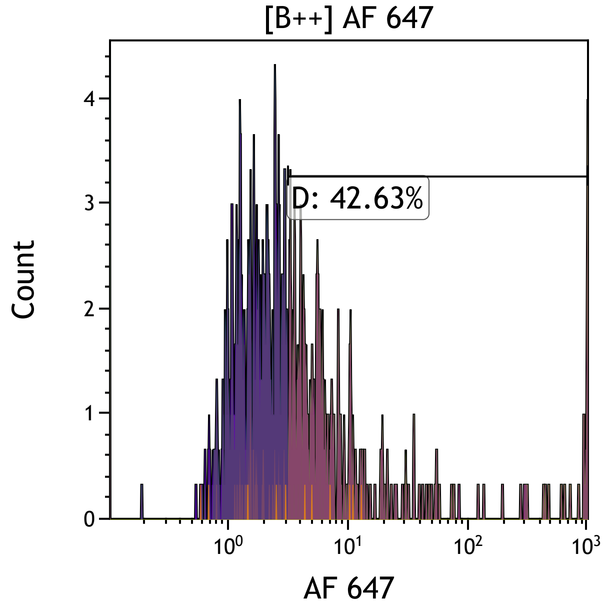

Gate	X-Med
All	2.67
D	6.24

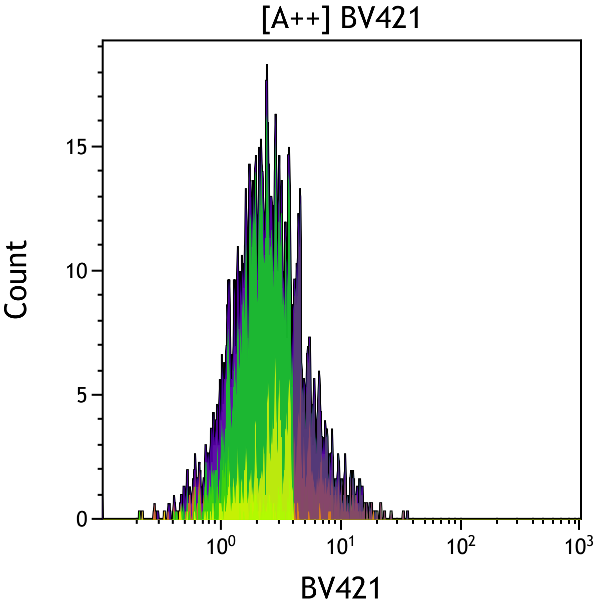

7 ipsi-2017-03-14-160411-7

Rat microglia M1M2 protocol - Imported

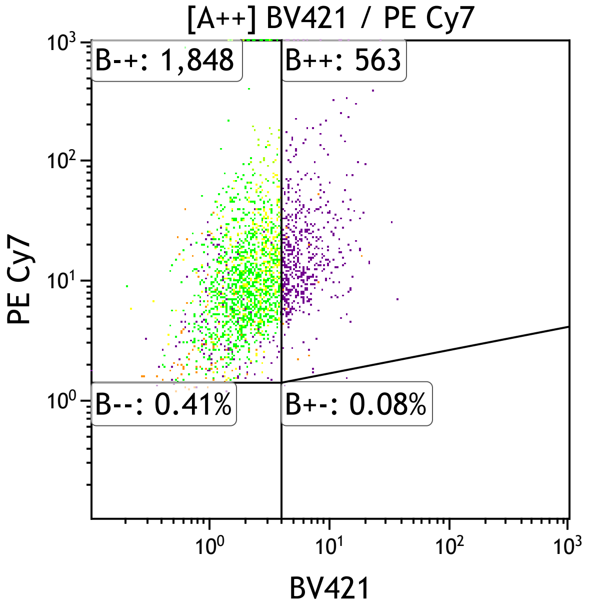

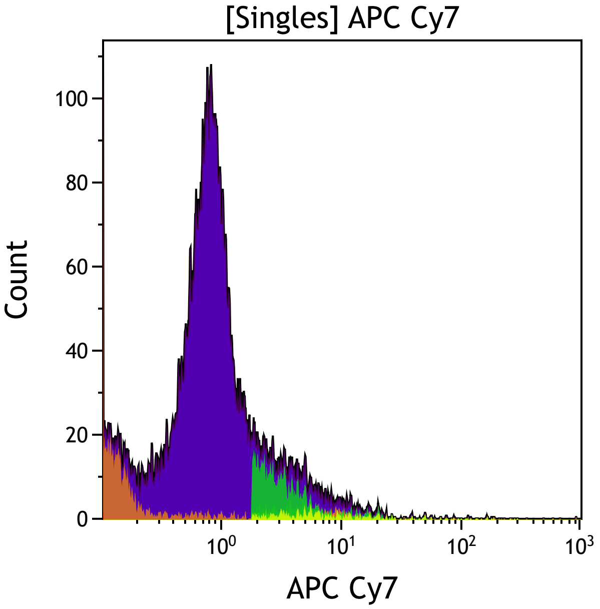

Gate	X-Med	Y-Med
All	2.45	10.42
B--	0.68	1.28
B-+	2.04	9.13
B+-	9.05	1.44
B++	5.54	15.11

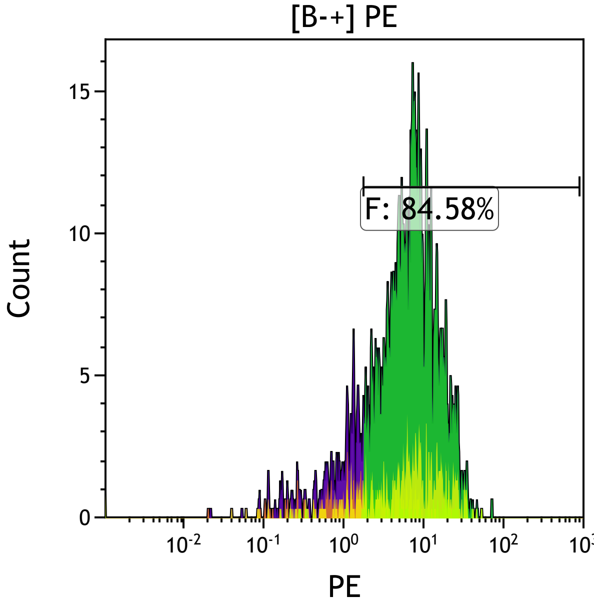

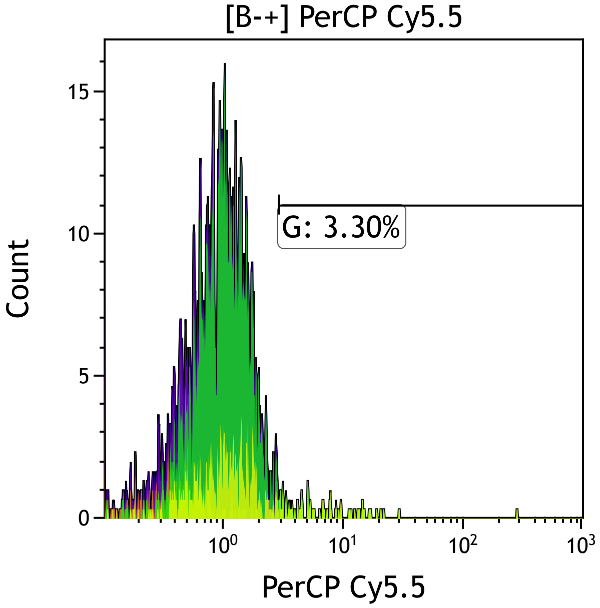

Gate	X-Med
All	6.33
F	7.44

Gate	X-Med
All	0.95
G	5.38

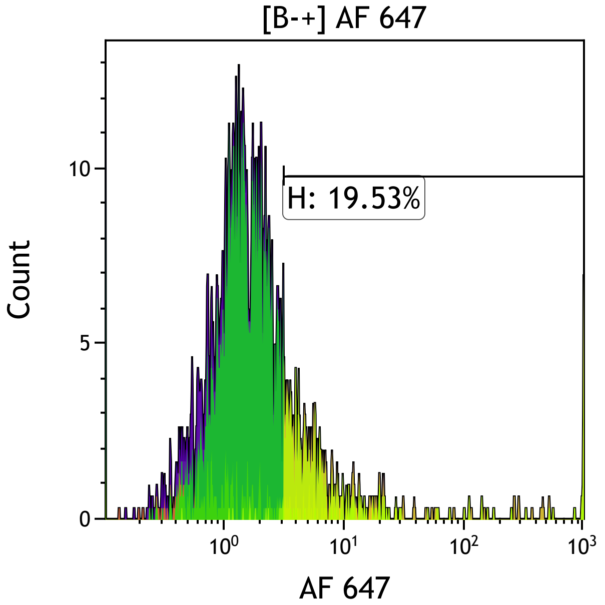

Gate	X-Med
All	1.57
H	5.73

8 ipsi-2017-03-14-160647-8

Rat microglia M1M2 protocol - Imported

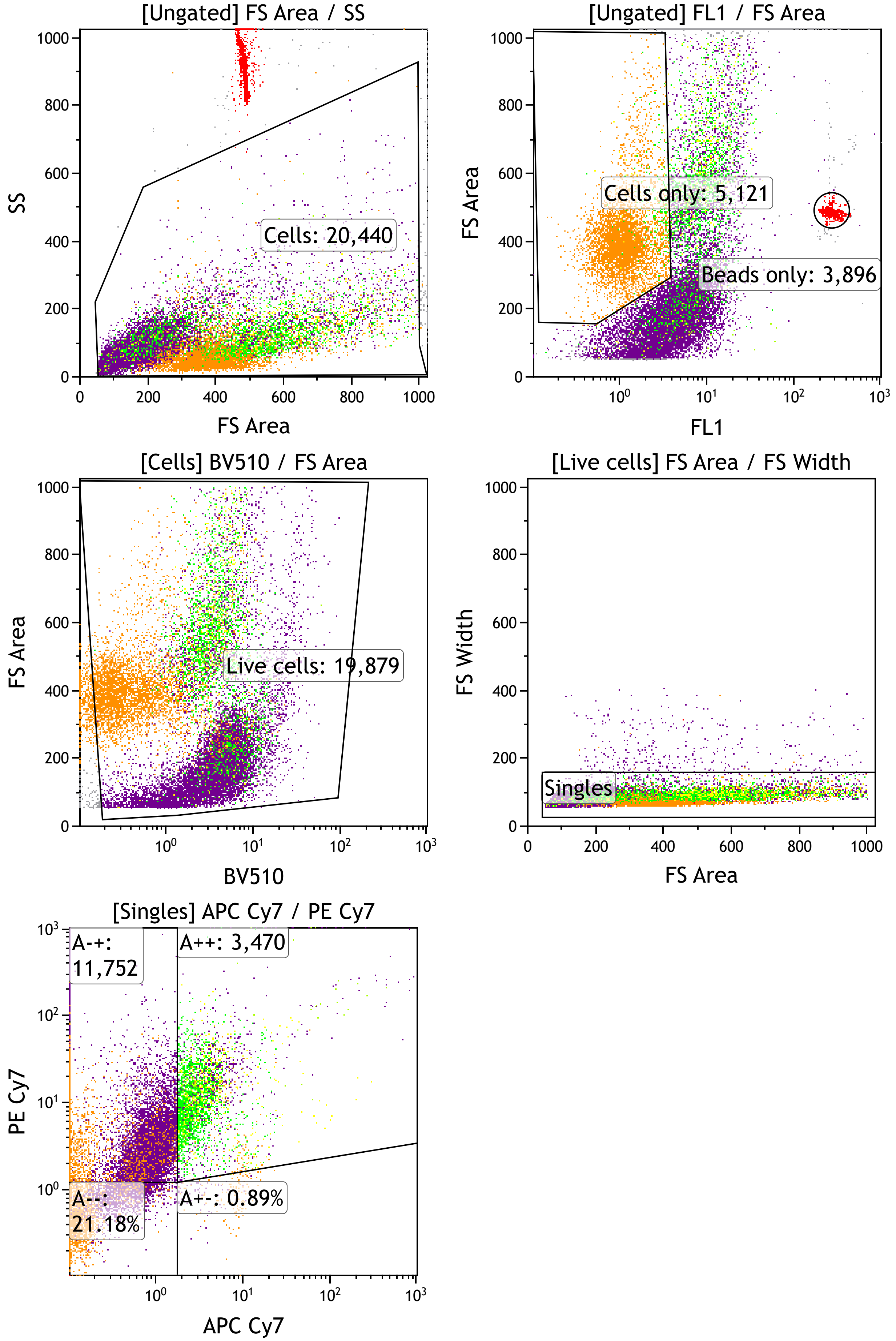

8 ipsi-2017-03-14-160647-8
Rat microglia M1M2 protocol - Imported

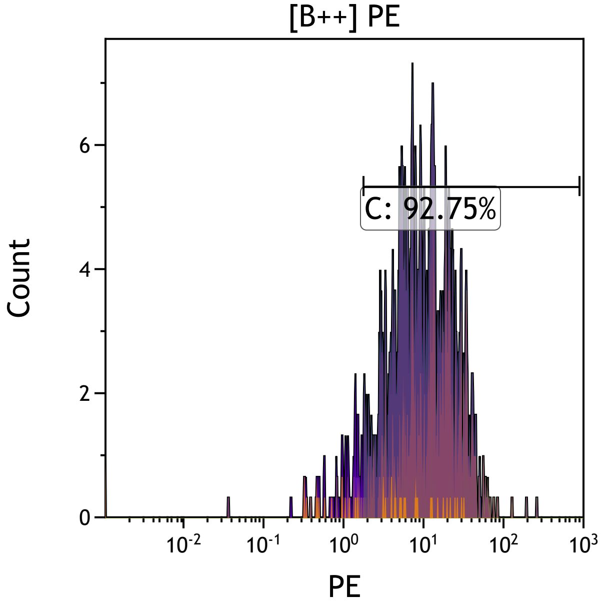

Gate	X-Med
All	8.85
C	9.62

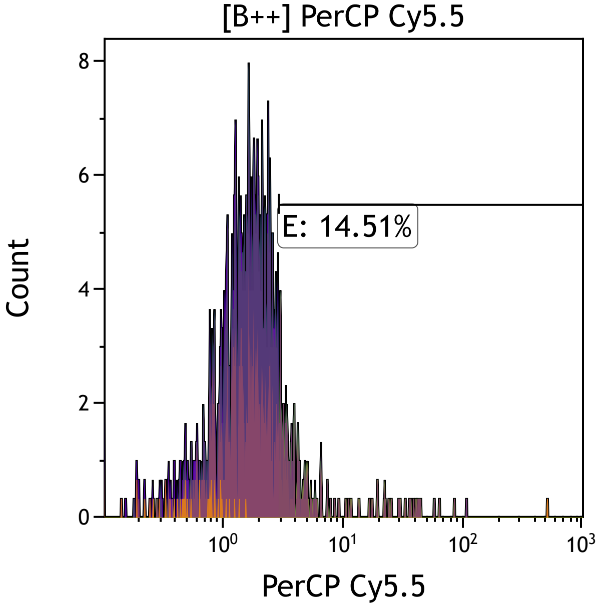

Gate	X-Med
All	1.66
E	4.17

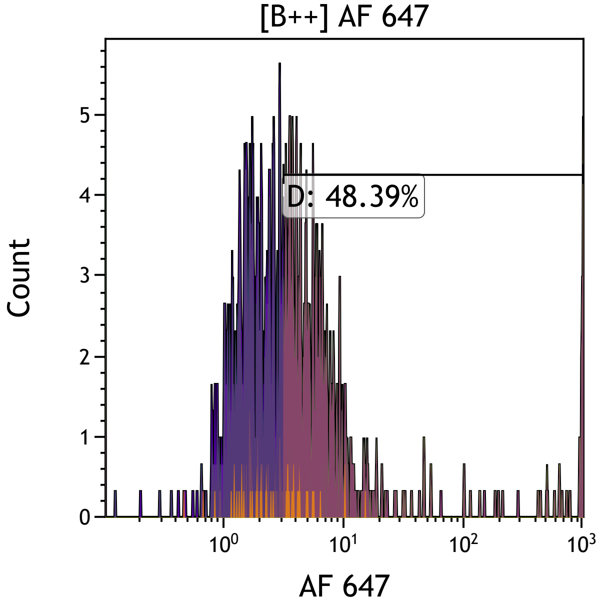

Gate	X-Med
All	3.04
D	5.85

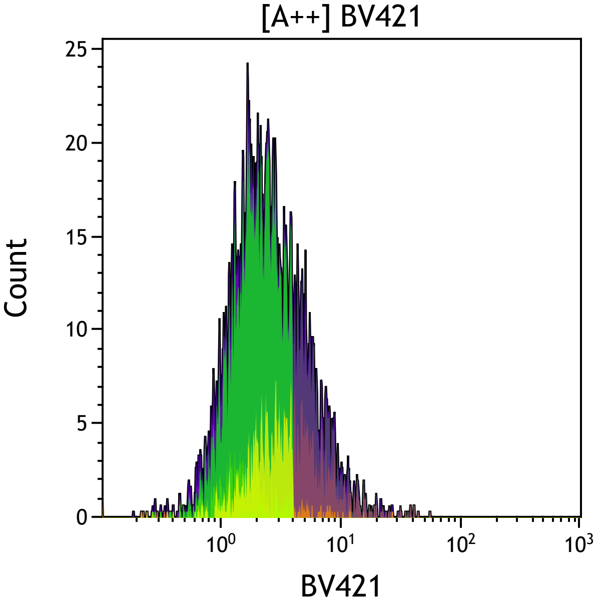

8 ipsi-2017-03-14-160647-8
Rat microglia M1M2 protocol - Imported

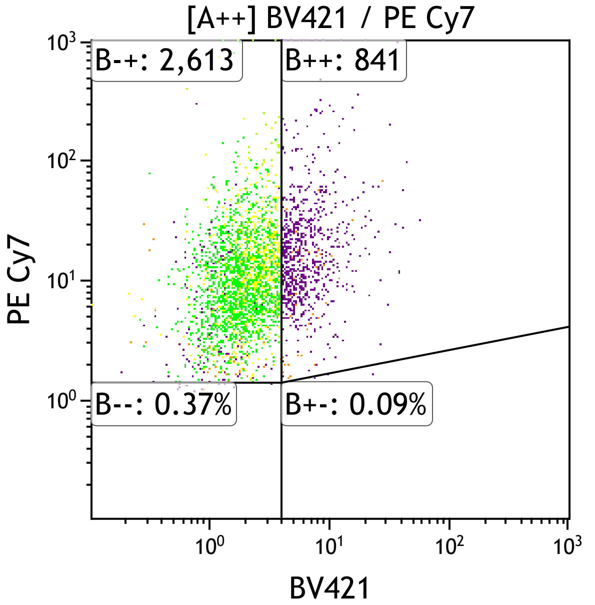

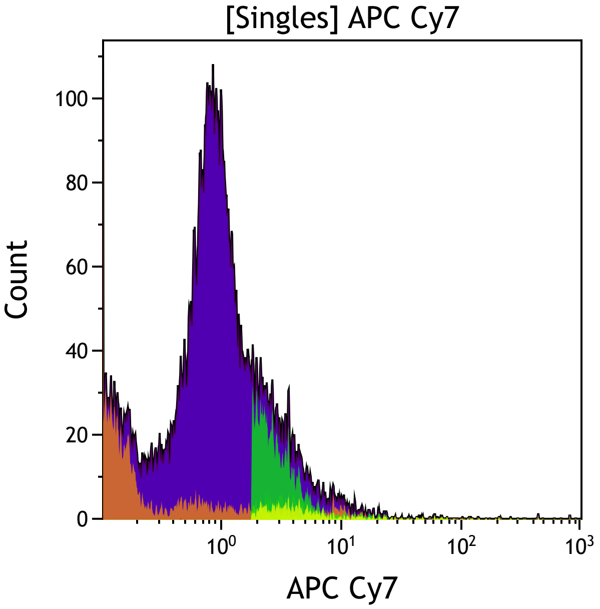

Gate	X-Med	Y-Med
All	2.38	10.82
B--	0.75	1.34
B-+	1.92	9.80
B+-	9.83	1.64
B++	5.81	15.79

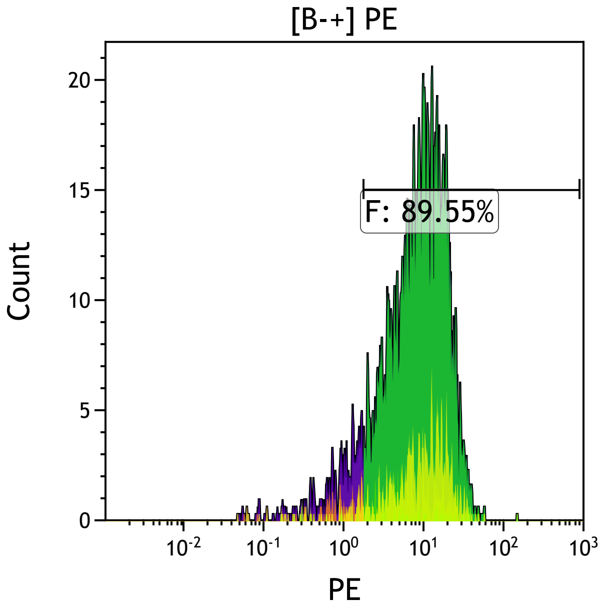

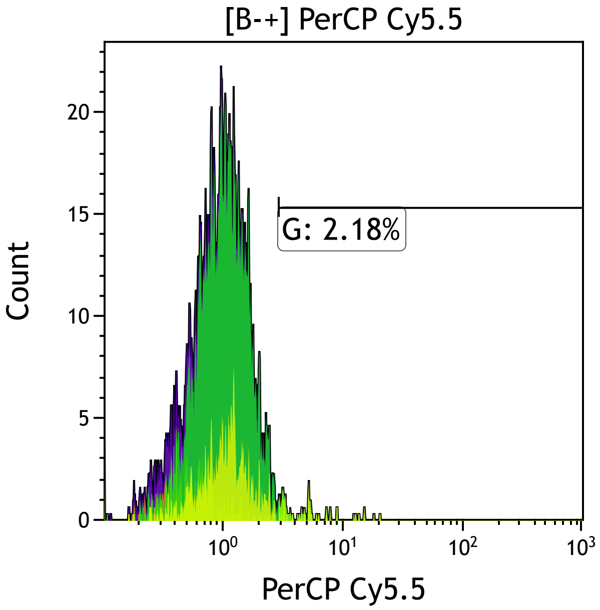

Gate	X-Med
All	8.67
F	9.76

Gate	X-Med
All	0.97
G	4.58

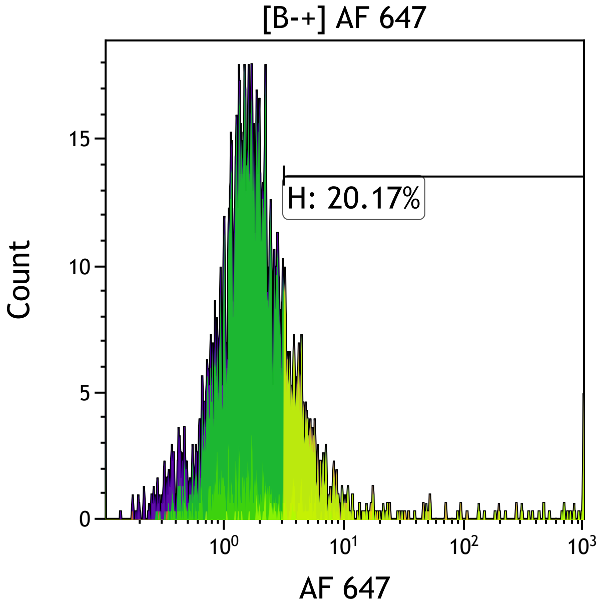

Gate	X-Med
All	1.72
H	4.66

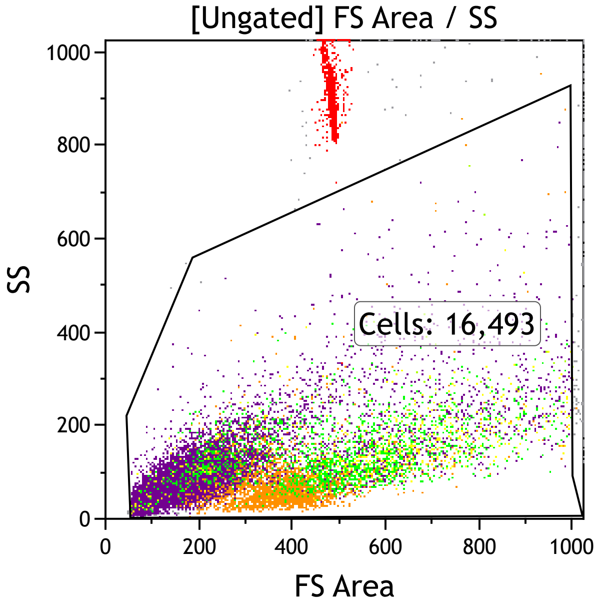
[Ungated] FS Area / SS
Cells: 16,493
SS
FS Area

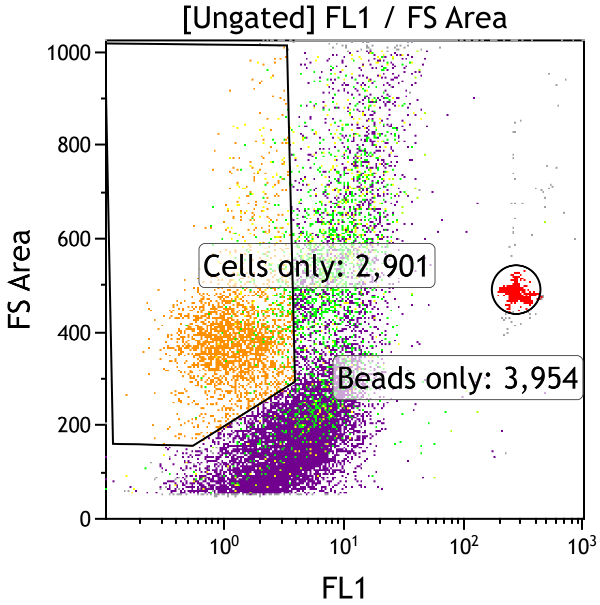
[Ungated] FL1 / FS Area
Cells only: 2,901
Beads only: 3,954
FS Area
FL1

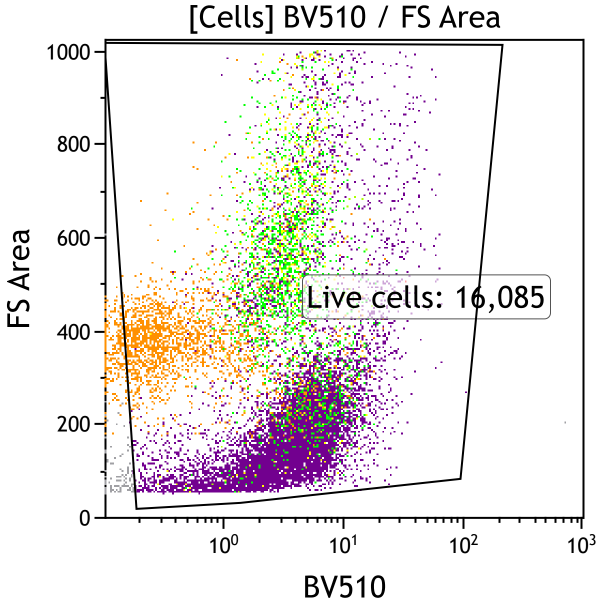
[Cells] BV510 / FS Area
Live cells: 16,085
FS Area
BV510

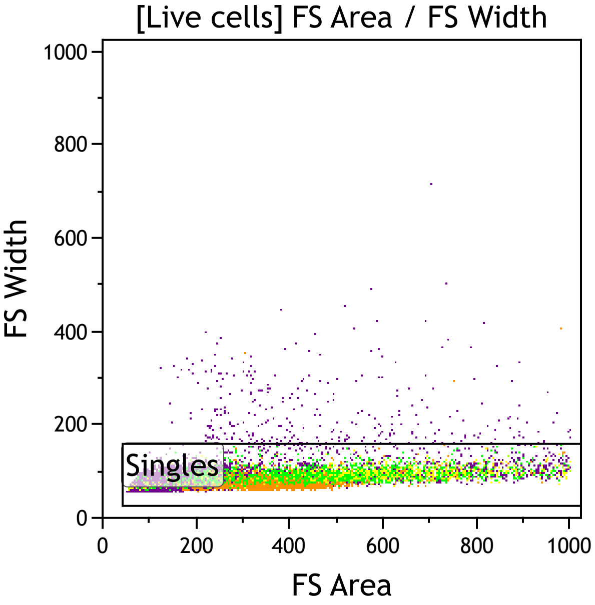
[Live cells] FS Area / FS Width
Singles
FS Width
FS Area

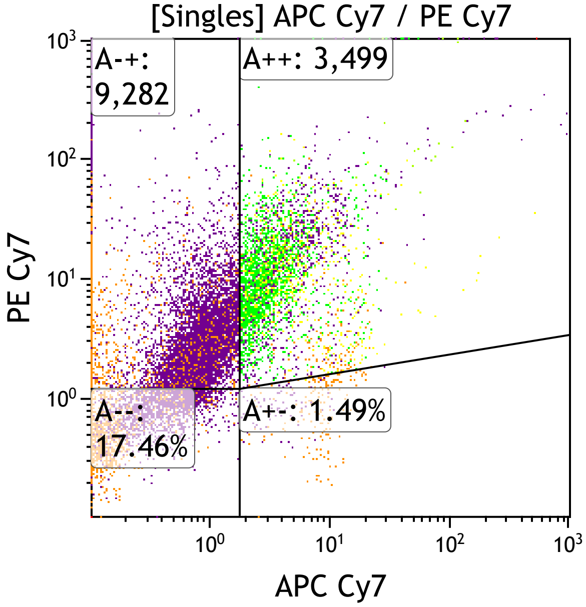
[Singles] APC Cy7 / PE Cy7
A-+: 9,282
A++: 3,499
A--: 17.46%
A+-: 1.49%
PE Cy7
APC Cy7

9 ipsi-2017-03-14-160919-9
Rat microglia M1M2 protocol - Imported

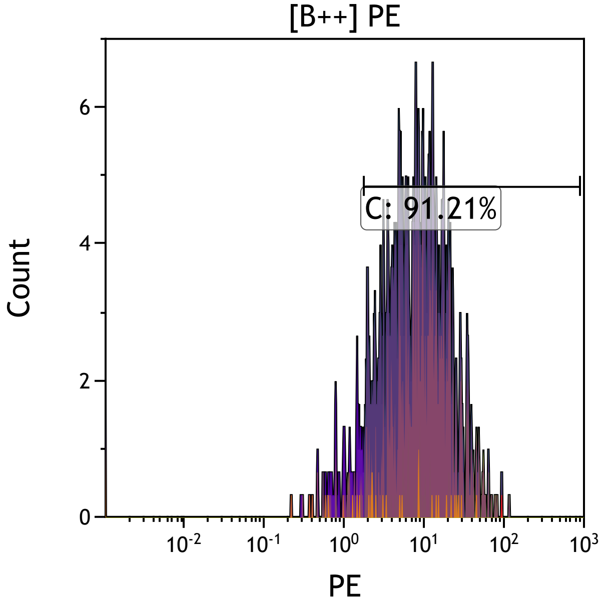

Gate	X-Med
All	7.78
C	8.53

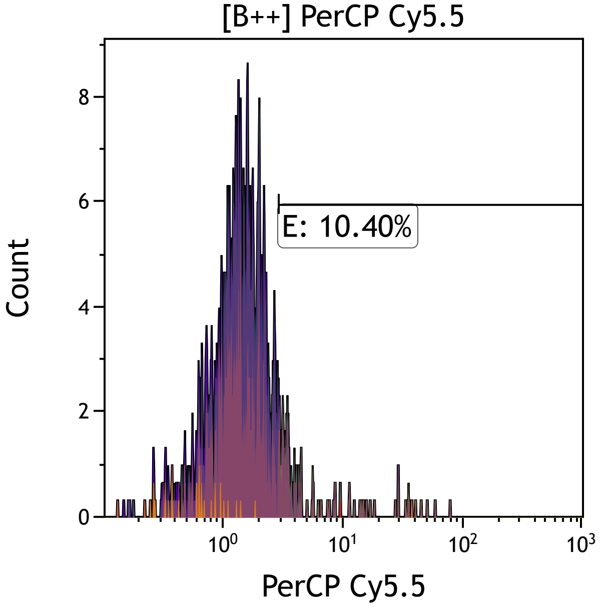

Gate	X-Med
All	1.44
E	4.09

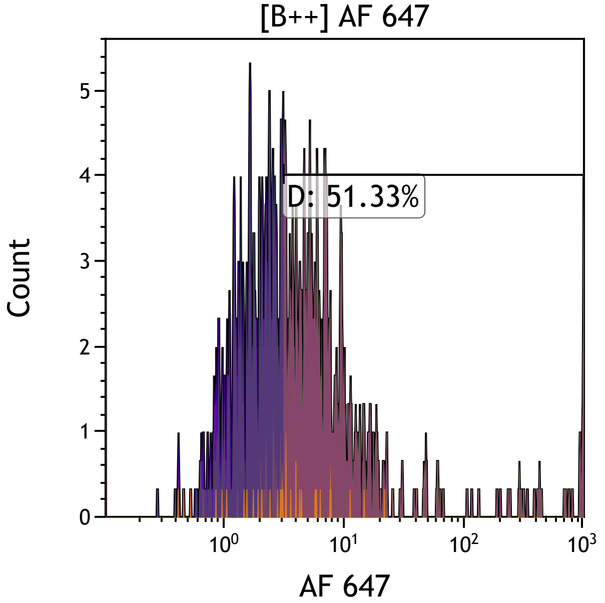

Gate	X-Med
All	3.23
D	6.56

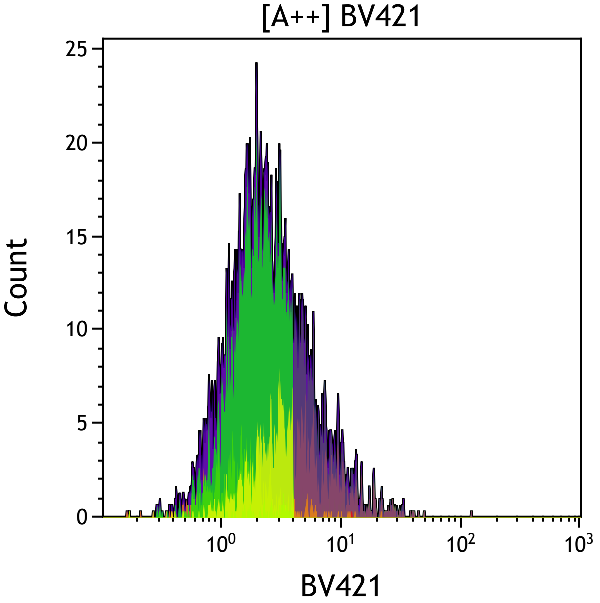

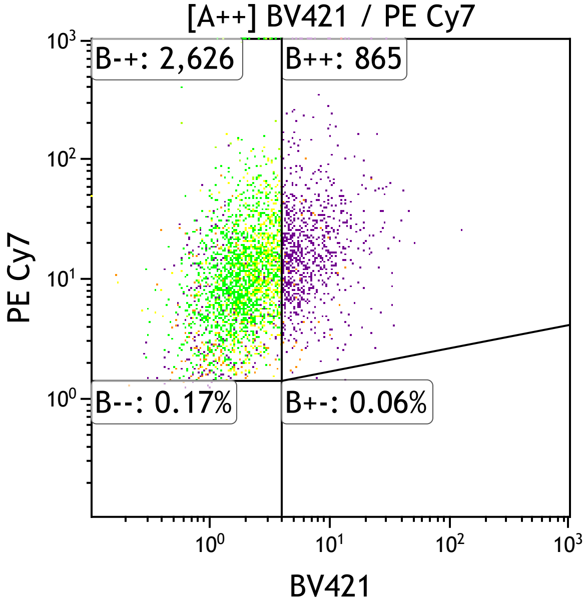

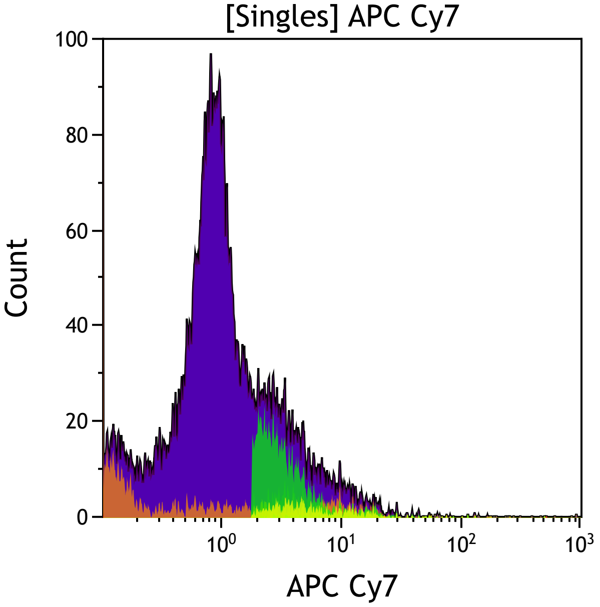

Gate	X-Med	Y-Med
All	2.35	11.07
B--	0.98	1.31
B-+	1.90	9.55
B+-	10.62	1.47
B++	5.98	16.91

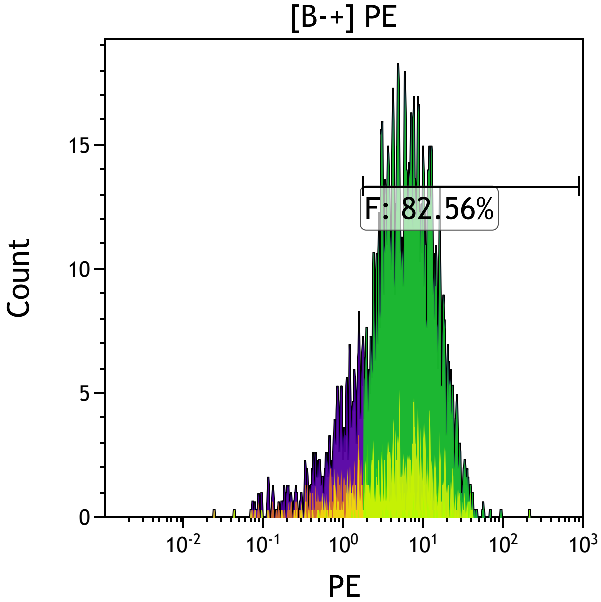

Gate	X-Med
All	5.25
F	6.66

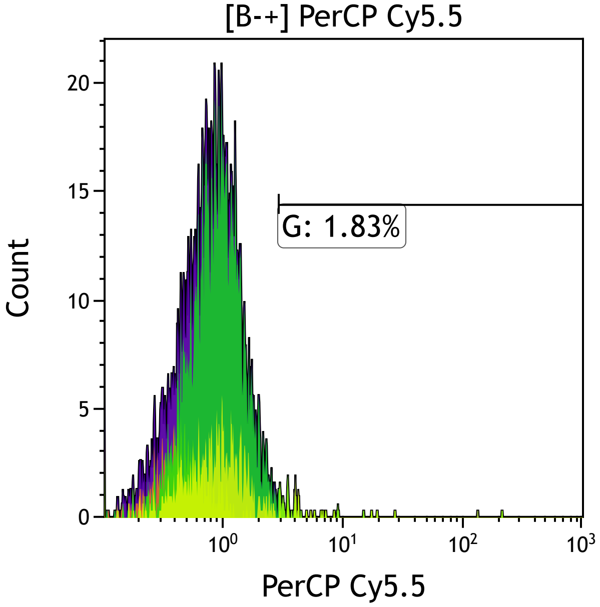

Gate	X-Med
All	0.81
G	4.01

[B-+] AF 647

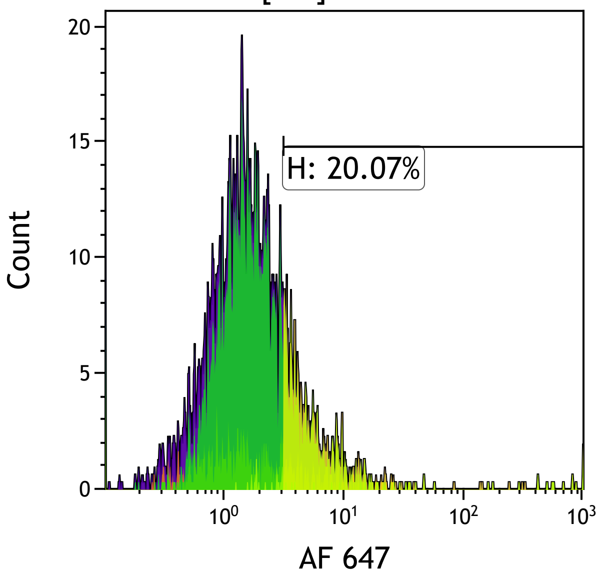

Gate	X-Med
All	1.60
H	4.93

10 ipsi-2017-03-14-161153-10
Rat microglia M1M2 protocol - Imported

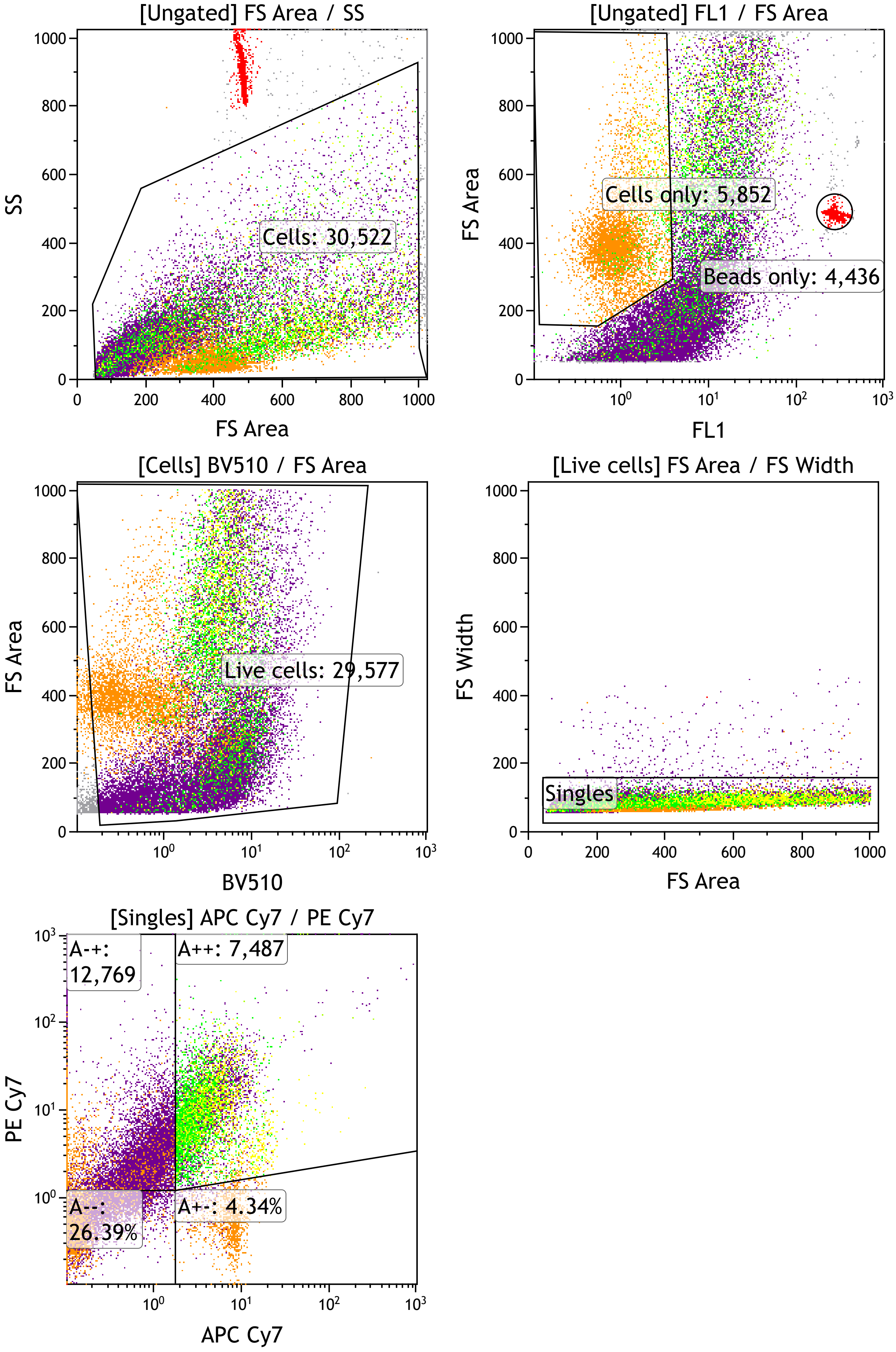

10 ipsi-2017-03-14-161153-10

Rat microglia M1M2 protocol - Imported

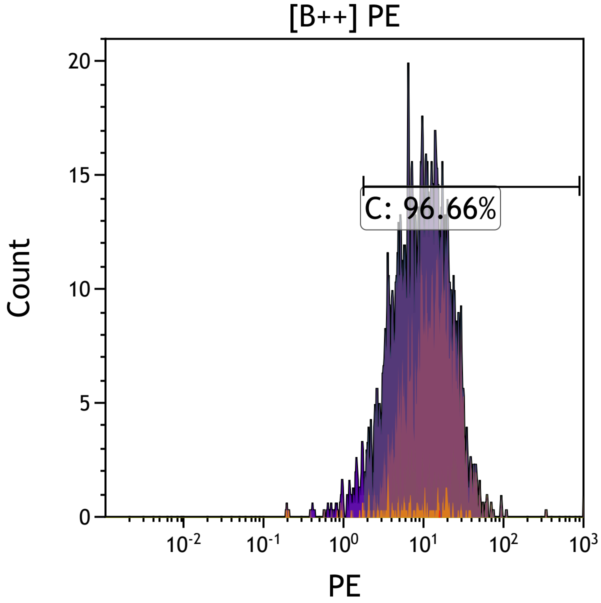

Gate	X-Med
All	9.63
C	9.86

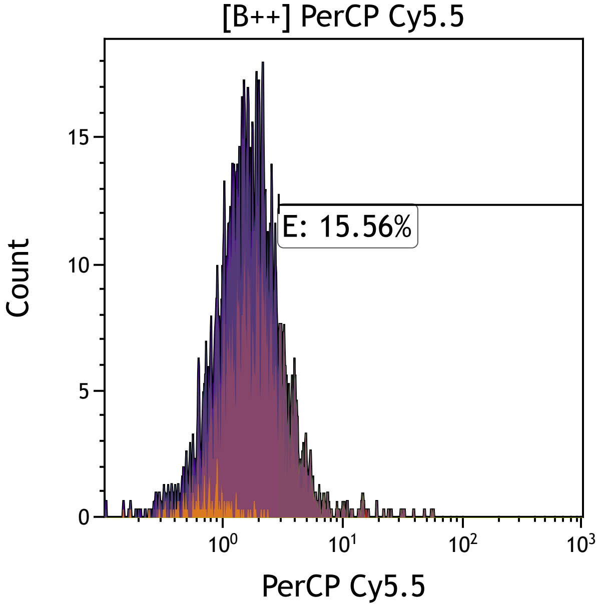

Gate	X-Med
All	1.63
E	3.89

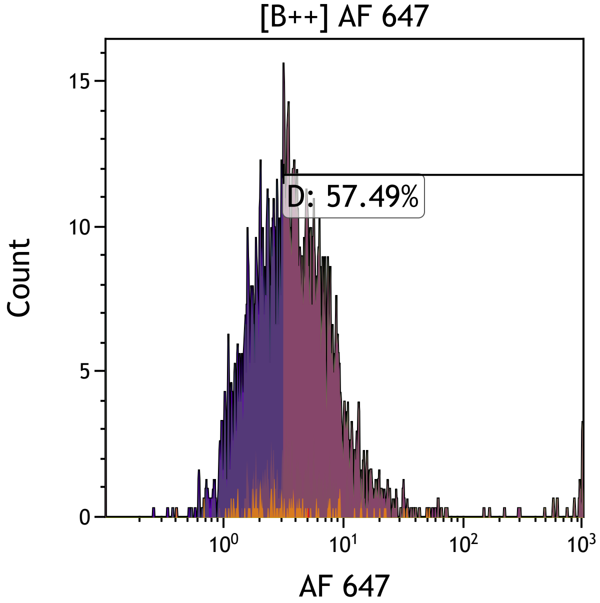

Gate	X-Med
All	3.55
D	5.59

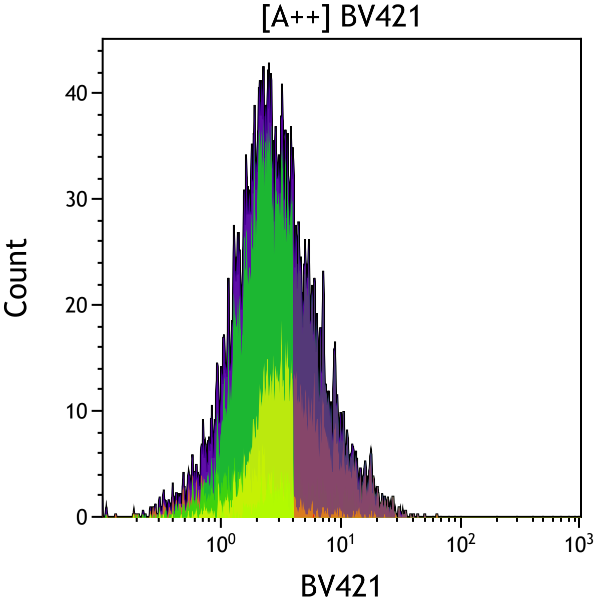

10 ipsi-2017-03-14-161153-10

Rat microglia M1M2 protocol - Imported

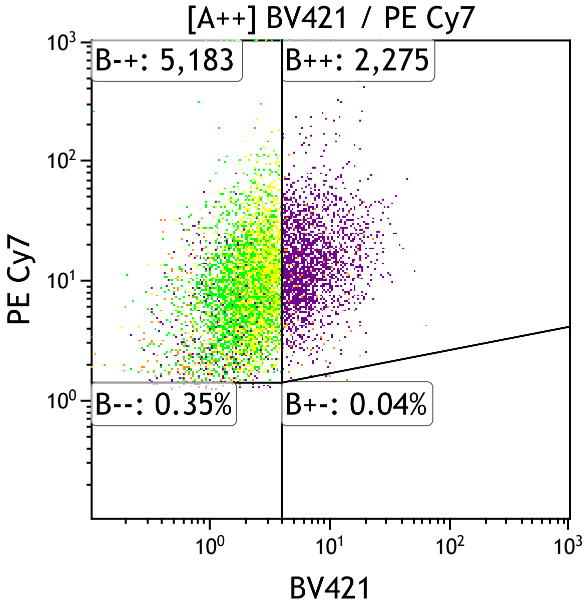

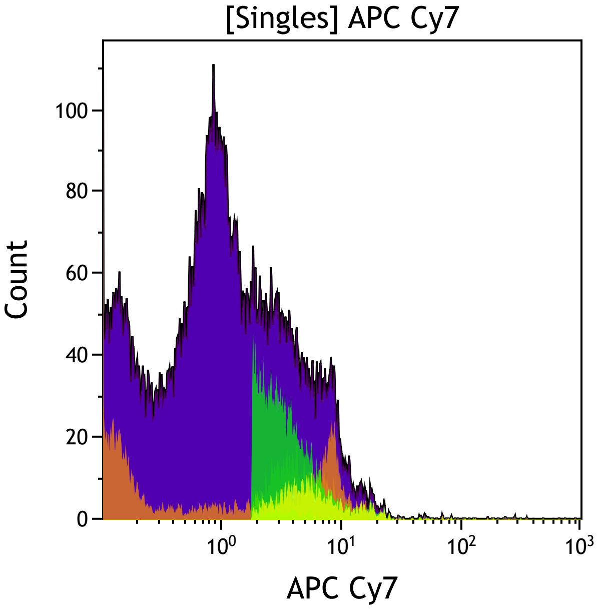

Gate	X-Med	Y-Med
All	2.70	9.45
B--	0.96	1.33
B-+	2.08	7.84
B+-	9.92	1.58
B++	6.28	13.68

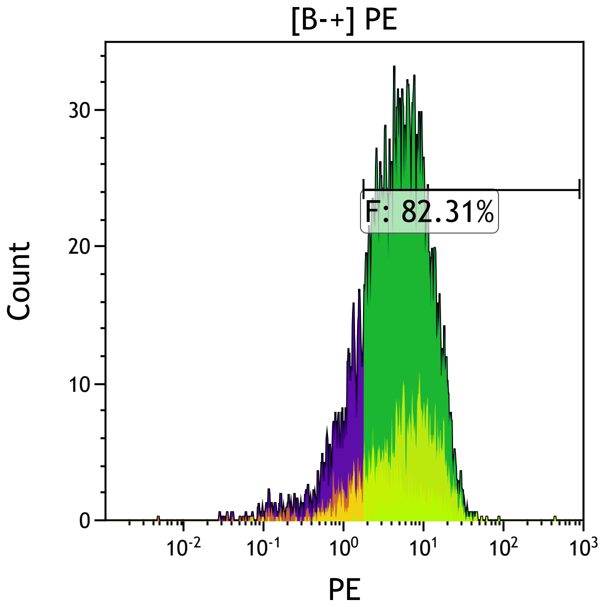

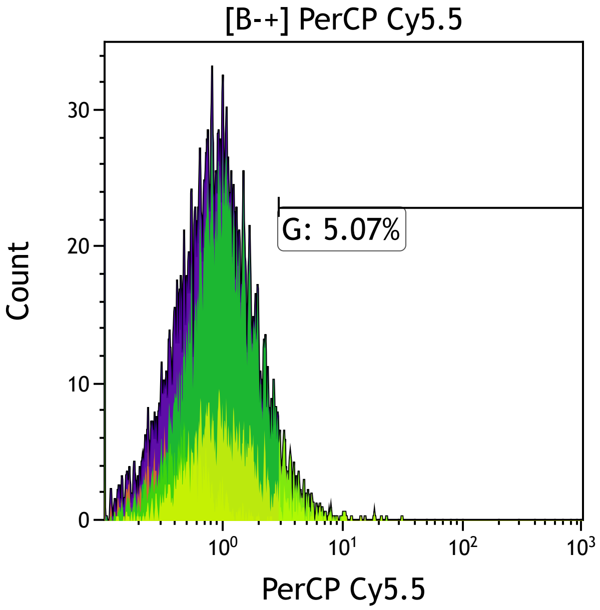

Gate	X-Med
All	4.72
F	5.87

Gate	X-Med
All	0.87
G	3.86

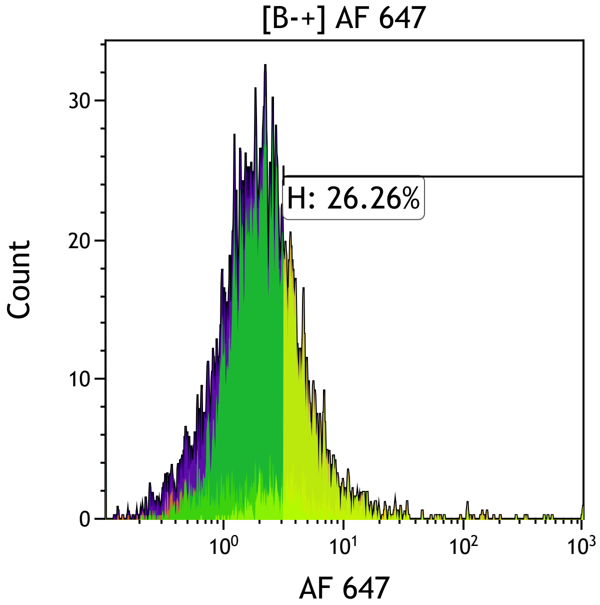

Gate	X-Med
All	2.01
H	4.63

11 ipsi-2017-03-14-161425-11
Rat microglia M1M2 protocol - Imported

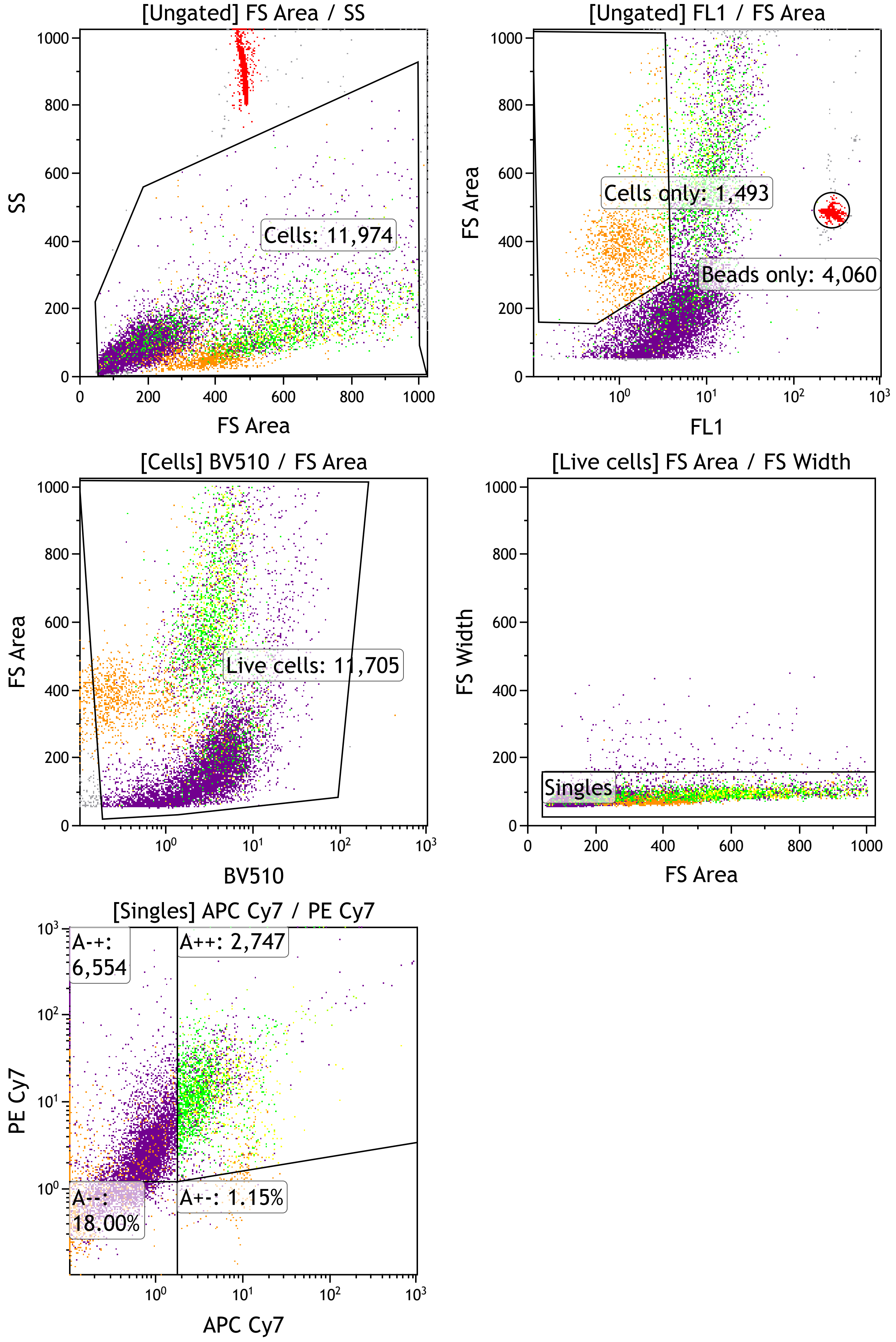

[B++] PE

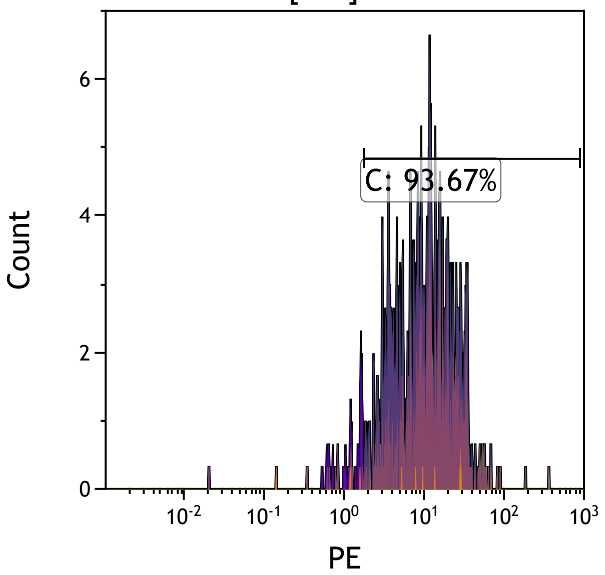

Gate	X-Med
All	9.70
C	10.78

[B++] PerCP Cy5.5

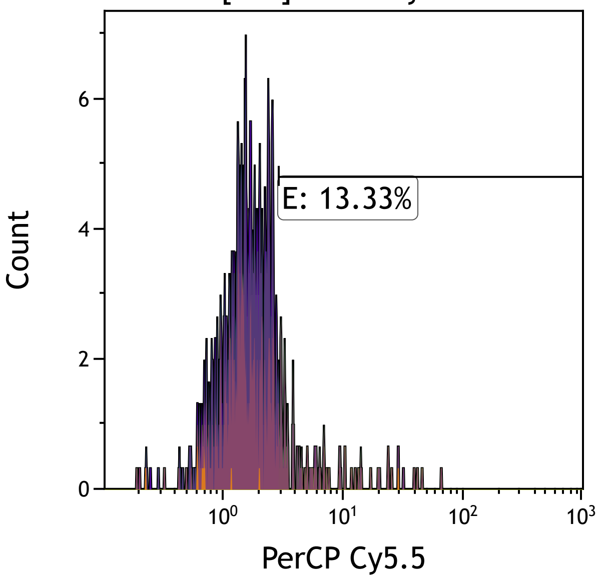

Gate	X-Med
All	1.70
E	4.85

[B++] AF 647

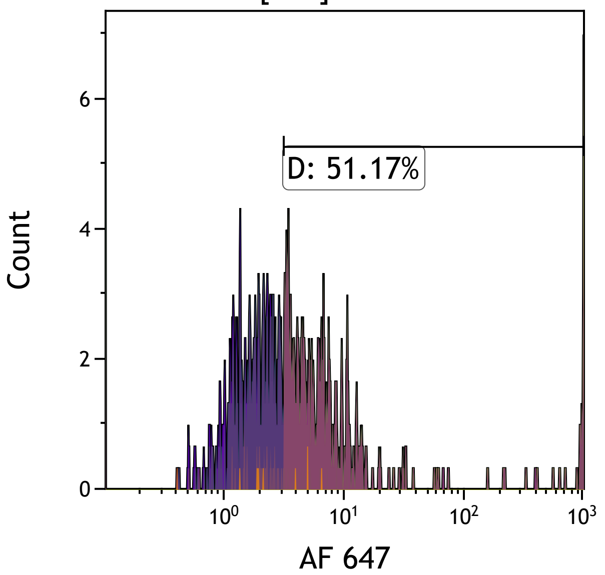

Gate	X-Med
All	3.23
D	6.20

[A++] BV421

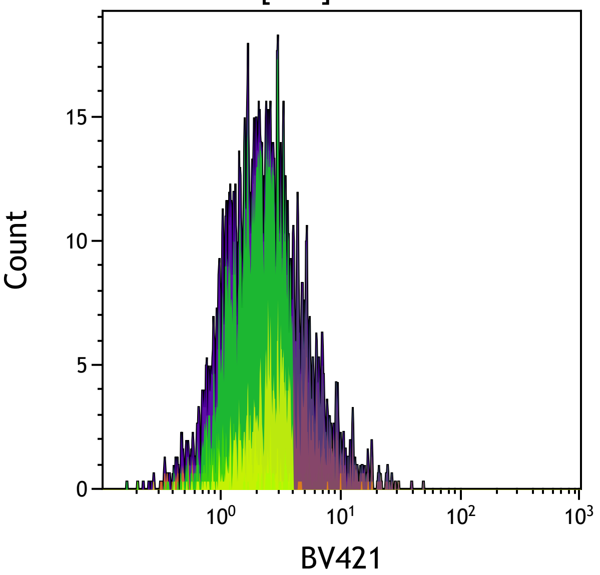

11 ipsi-2017-03-14-161425-11

Rat microglia M1M2 protocol - Imported

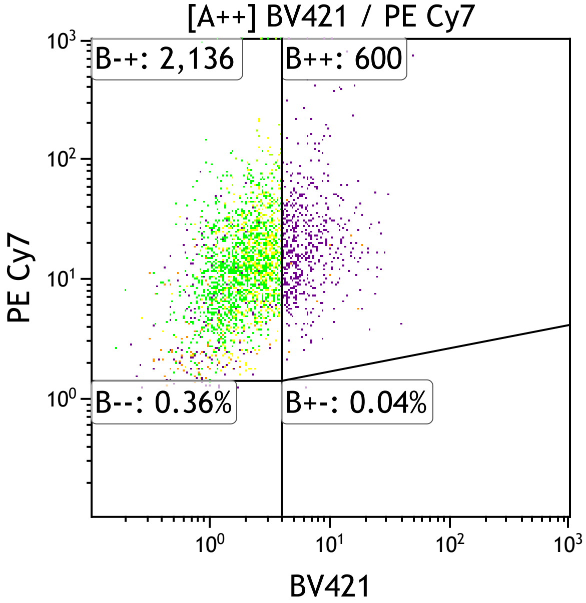

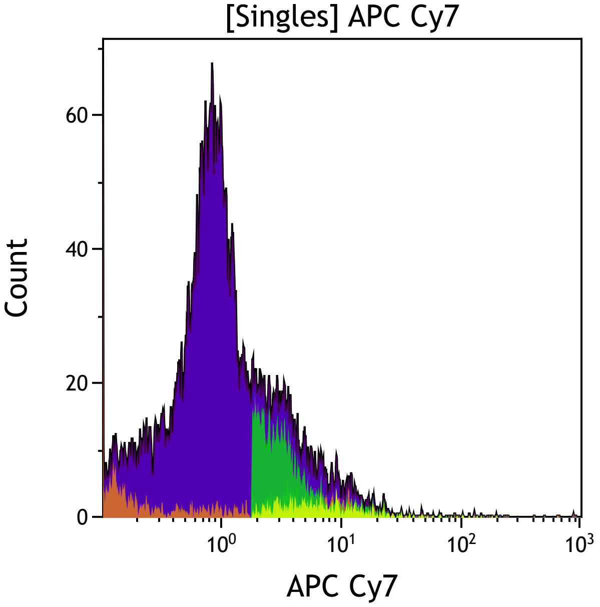

Gate	X-Med	Y-Med
All	2.25	12.64
B--	0.94	1.29
B-+	1.86	11.49
B+-	6.20	1.25
B++	5.87	18.37

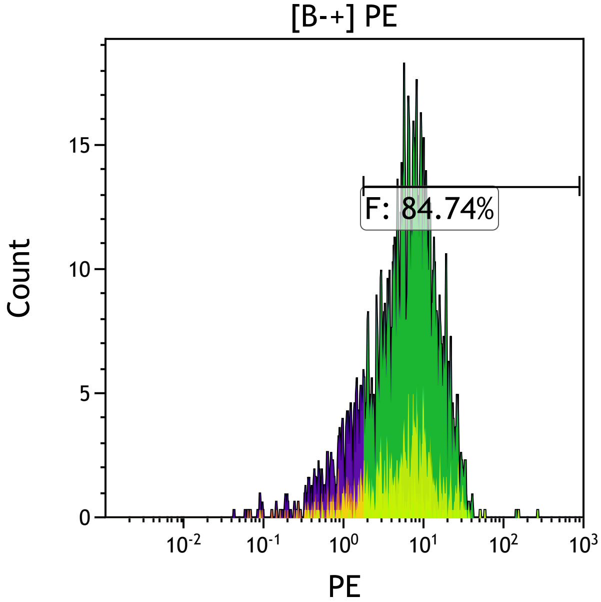

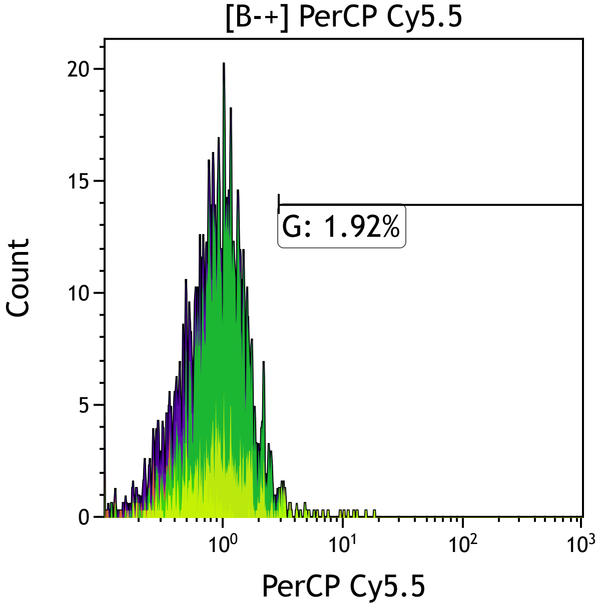

Gate	X-Med
All	6.37
F	7.51

Gate	X-Med
All	0.89
G	3.62

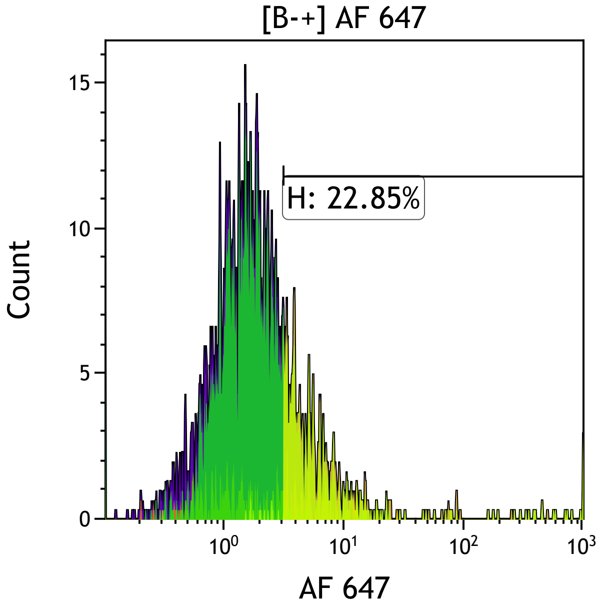

Gate	X-Med
All	1.73
H	5.23

12 ipsi-2017-03-14-161658-12

Rat microglia M1M2 protocol - Imported

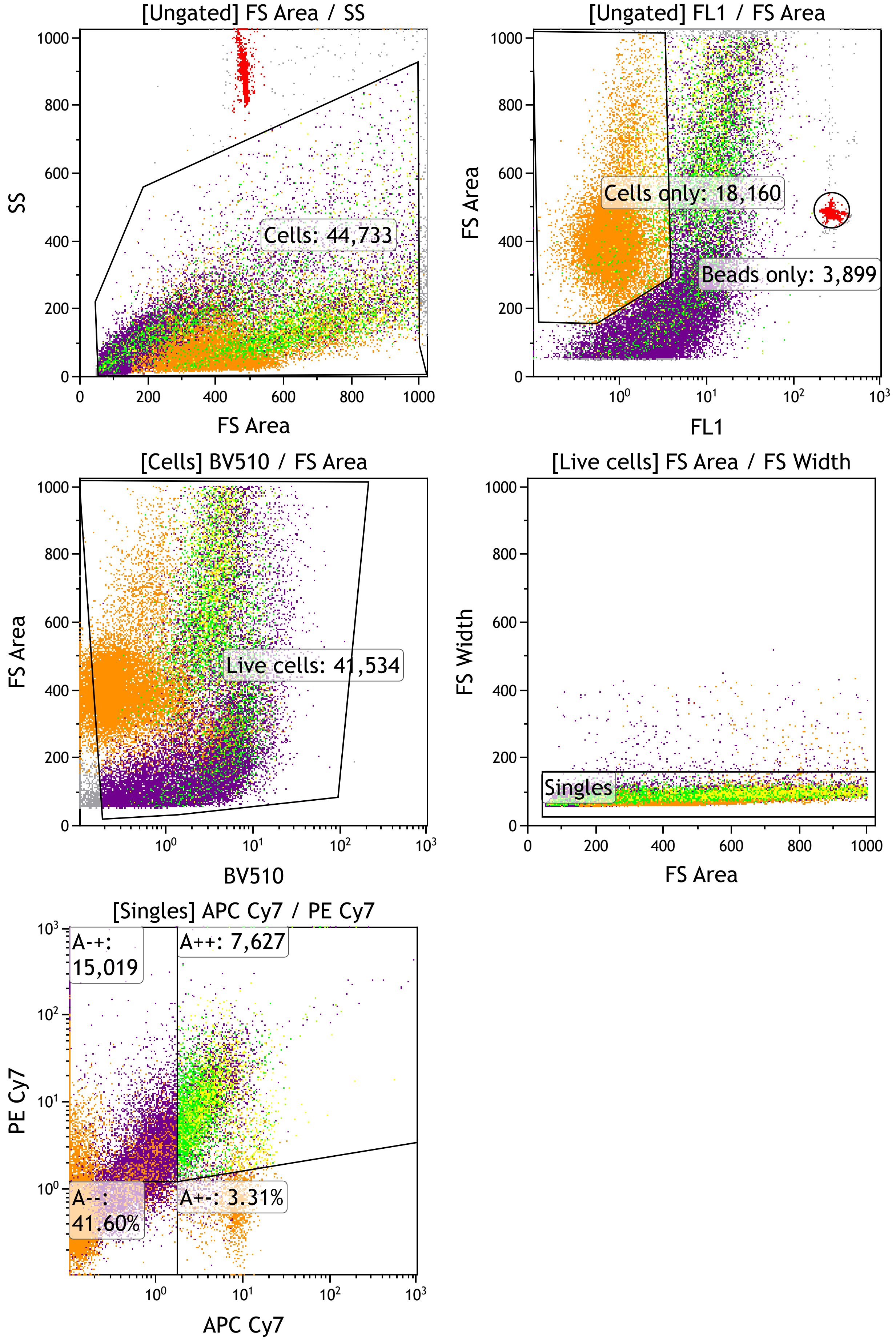

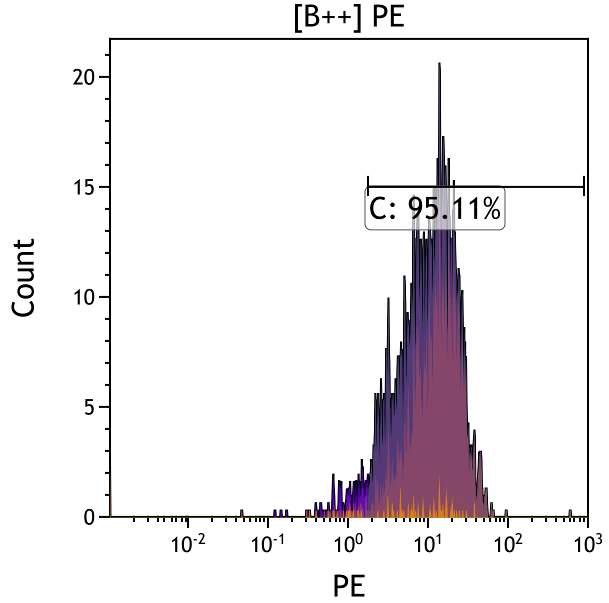

Gate	X-Med
All	10.56
C	11.17

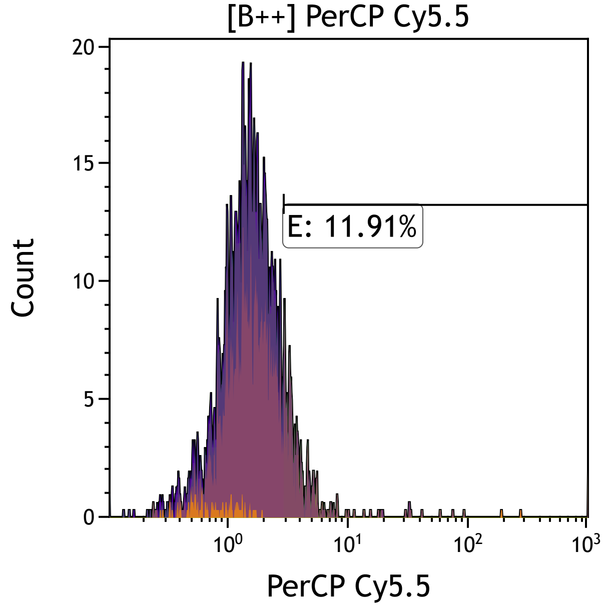

Gate	X-Med
All	1.55
E	3.62

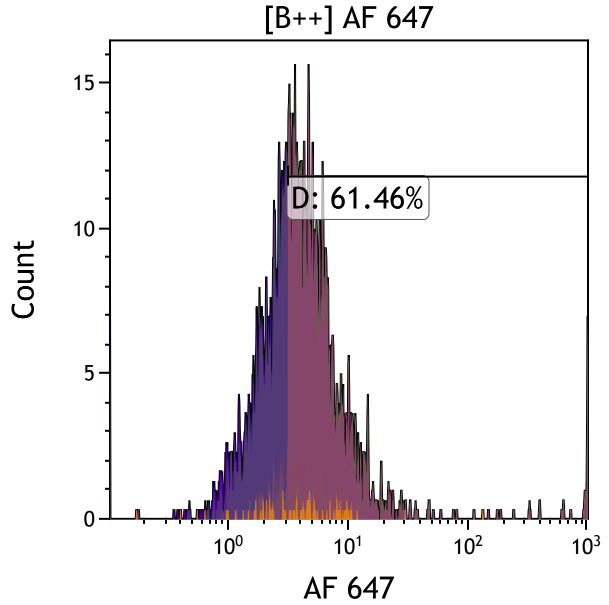

Gate	X-Med
All	3.74
D	5.22

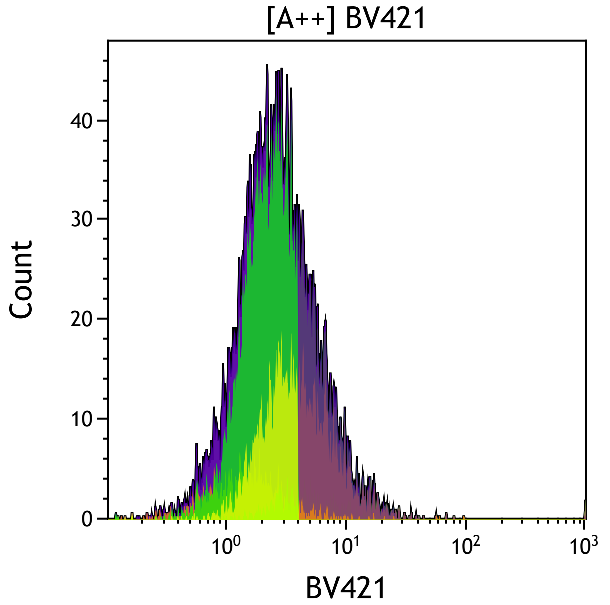

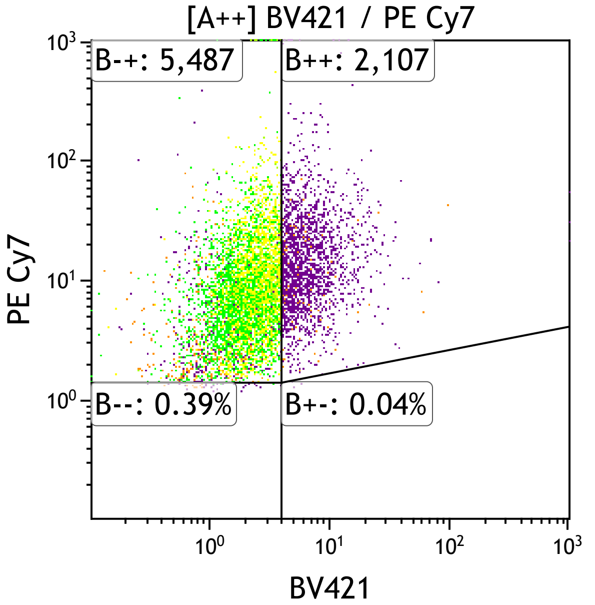

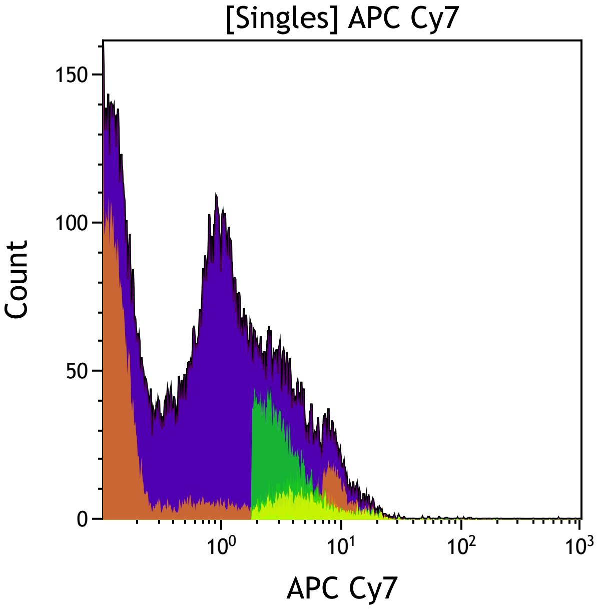

Gate	X-Med	Y-Med
All	2.62	8.49
B--	0.92	1.34
B-+	2.06	7.22
B+-	5.15	1.40
B++	5.95	13.13

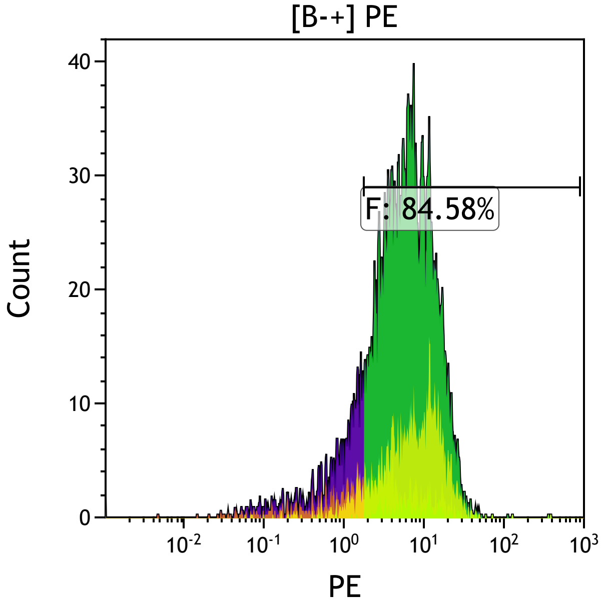

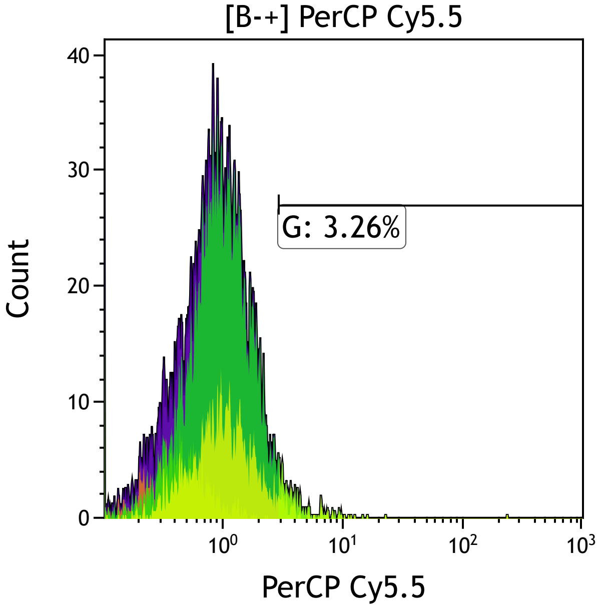

Gate	X-Med
All	5.52
F	6.58

Gate	X-Med
All	0.88
G	3.77

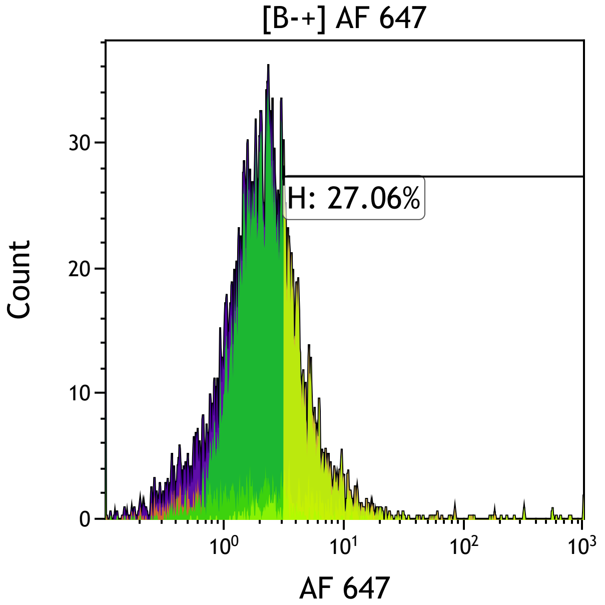

Gate	X-Med
All	2.11
H	4.46

1 contra-2017-03-14-161931-13
Rat microglia M1M2 protocol - Imported

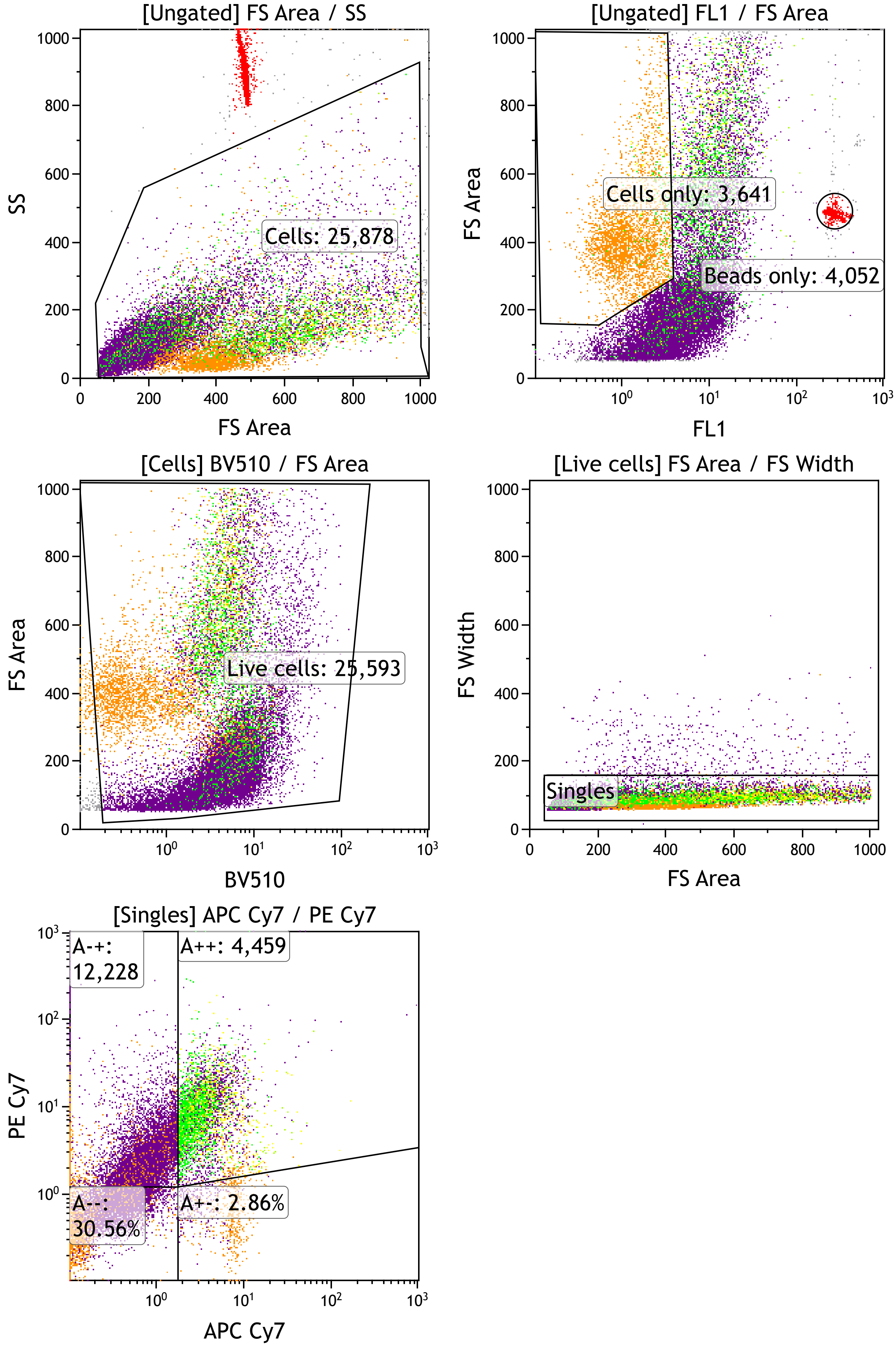

1 contra-2017-03-14-161931-13
Rat microglia M1M2 protocol - Imported

[B++] PE

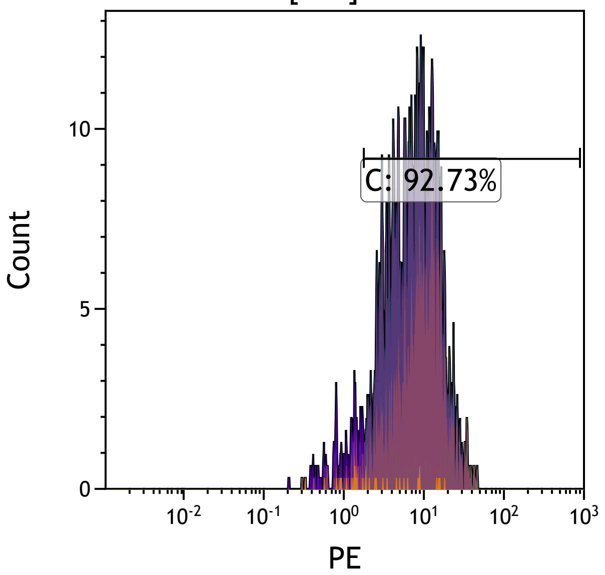

Gate	X-Med
All	7.28
C	7.89

[B++] PerCP Cy5.5

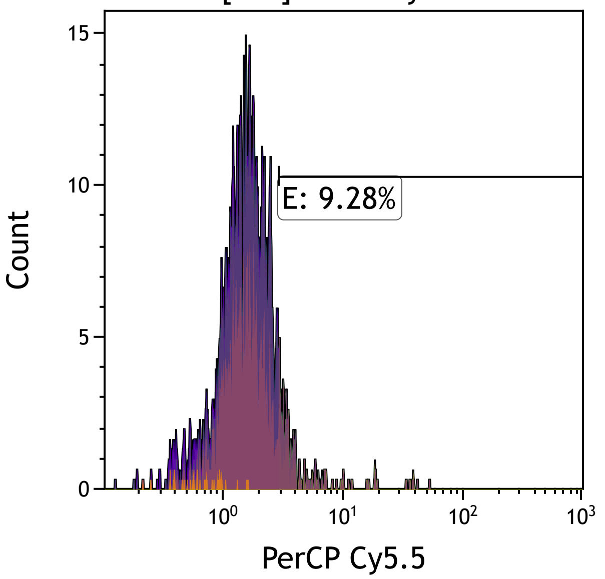

Gate	X-Med
All	1.59
E	3.56

[B++] AF 647

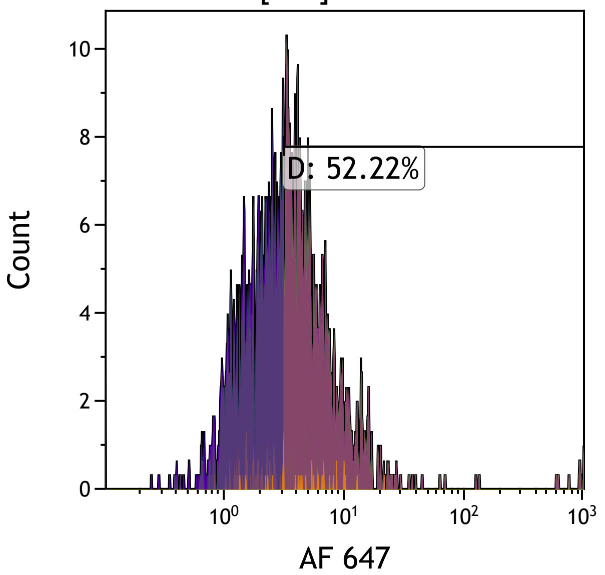

Gate	X-Med
All	3.31
D	5.17

[A++] BV421

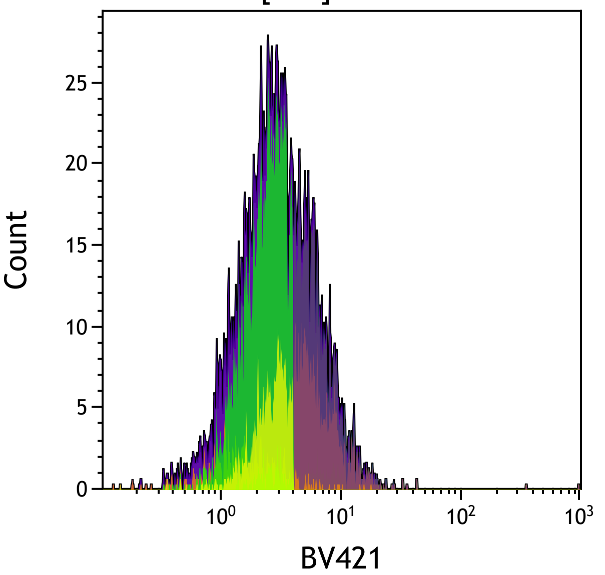

1 contra-2017-03-14-161931-13
Rat microglia M1M2 protocol - Imported

[A++] BV421 / PE Cy7

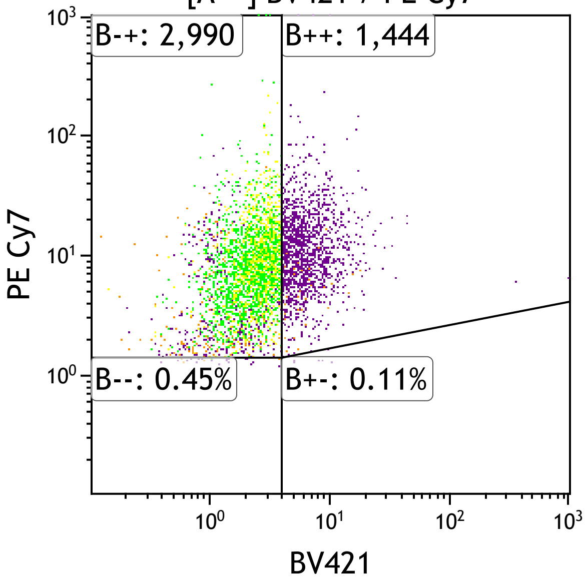

[Singles] APC Cy7

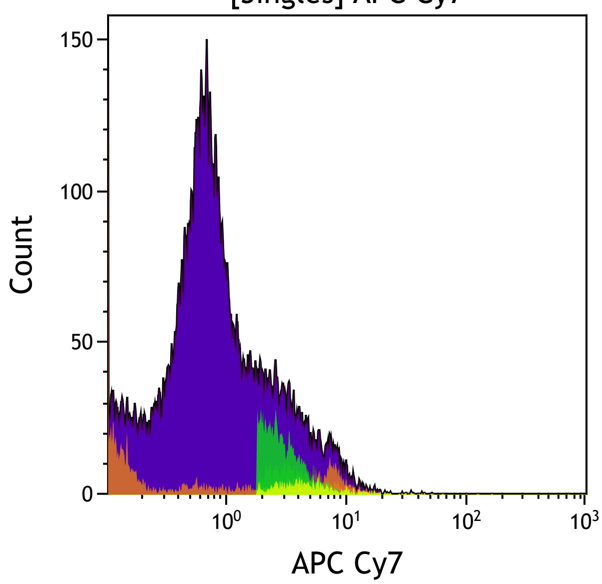

Gate	X-Med	Y-Med
All	2.92	8.24
B--	1.04	1.34
B-+	2.23	7.25
B+-	8.14	1.32
B++	5.96	10.50

[B-+] PE

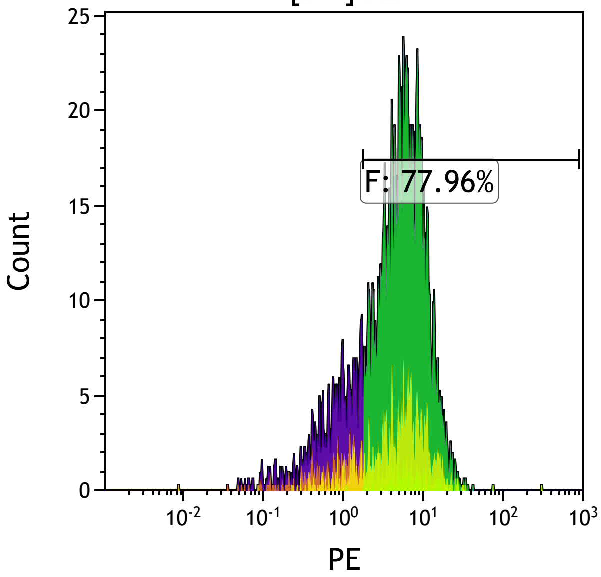

[B-+] PerCP Cy5.5

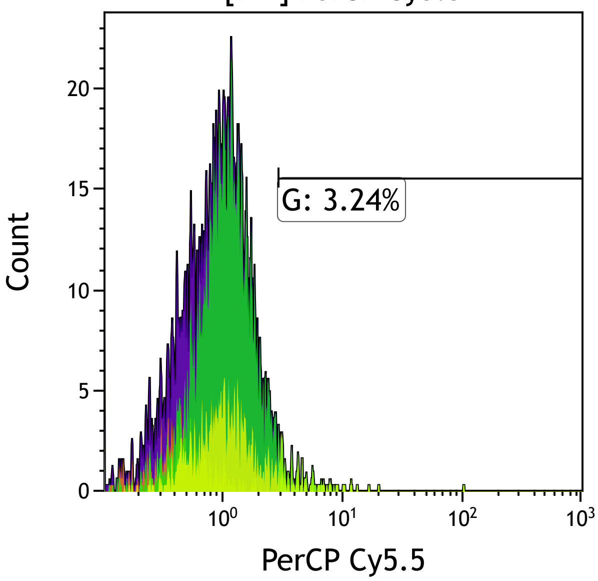

Gate	X-Med
All	4.72
F	5.94

Gate	X-Med
All	0.93
G	4.11

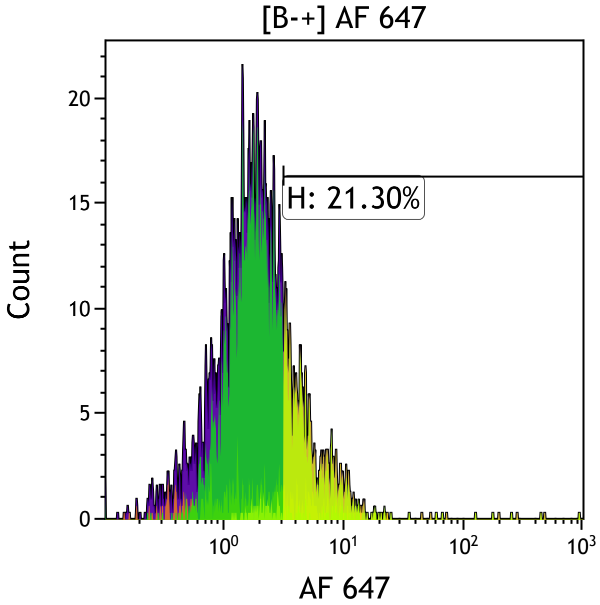

Gate	X-Med
All	1.84
H	4.76

2 contra-2017-03-14-162204-14

Rat microglia M1M2 protocol - Imported

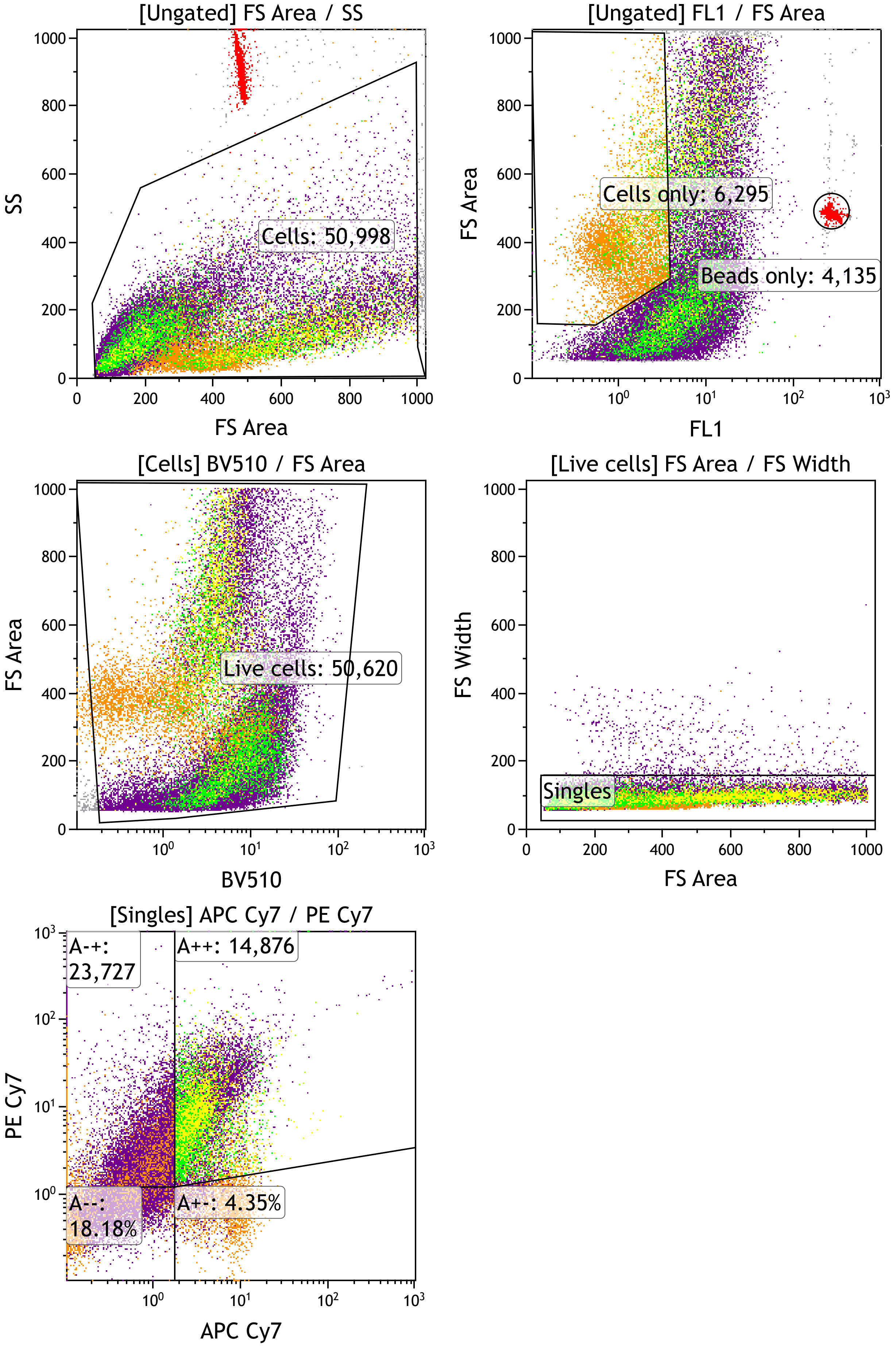

2 contra-2017-03-14-162204-14
Rat microglia M1M2 protocol - Imported

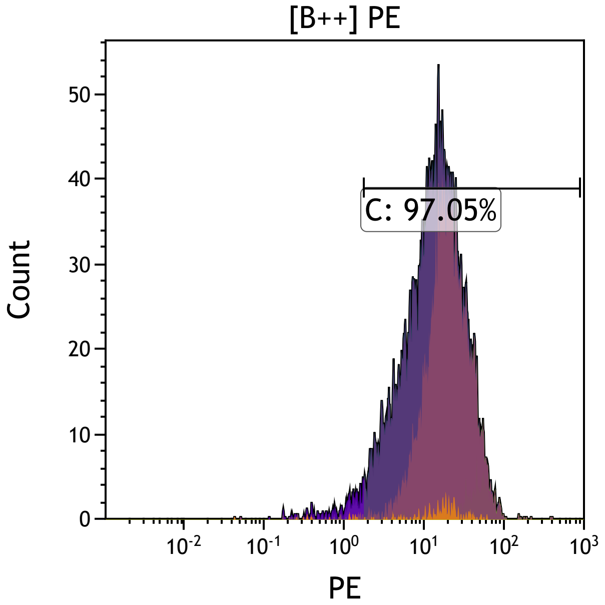

Gate	X-Med
All	14.03
C	14.39

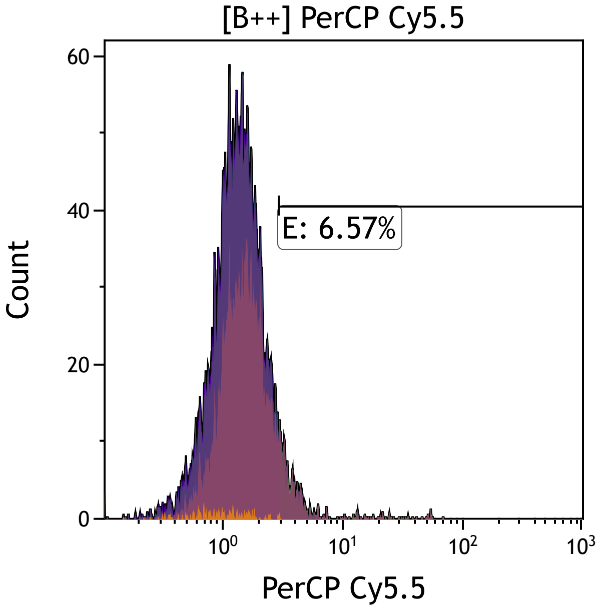

Gate	X-Med
All	1.37
E	3.66

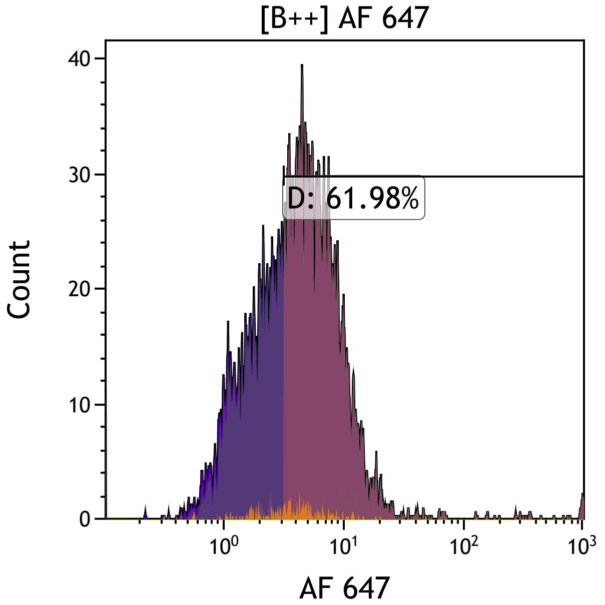

Gate	X-Med
All	4.07
D	5.90

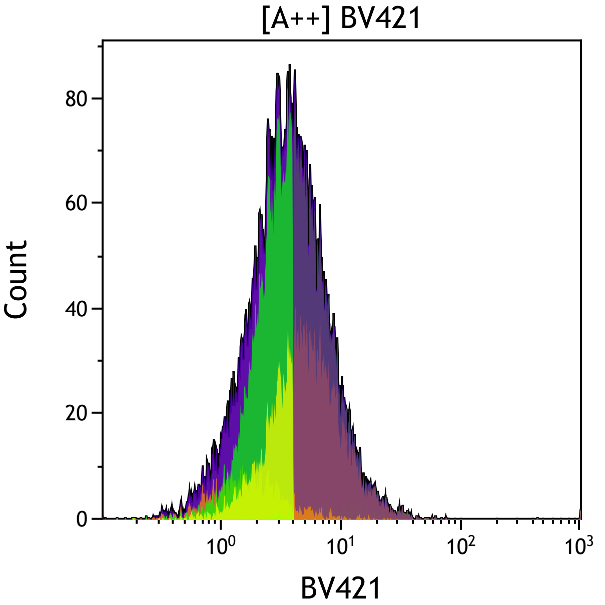

2 contra-2017-03-14-162204-14
Rat microglia M1M2 protocol - Imported

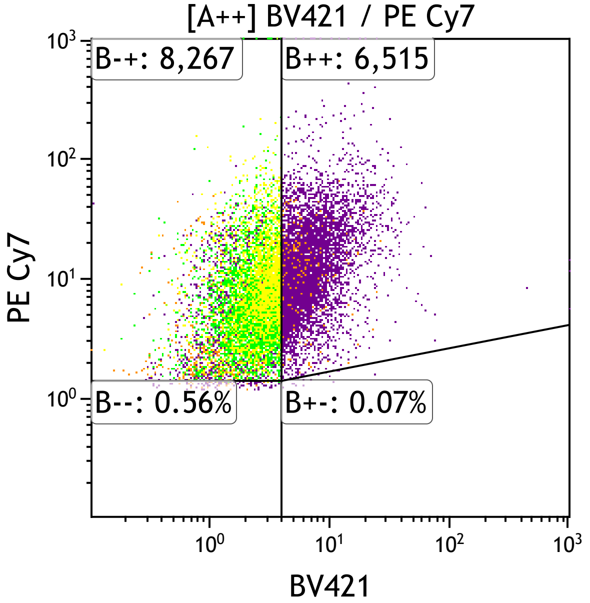

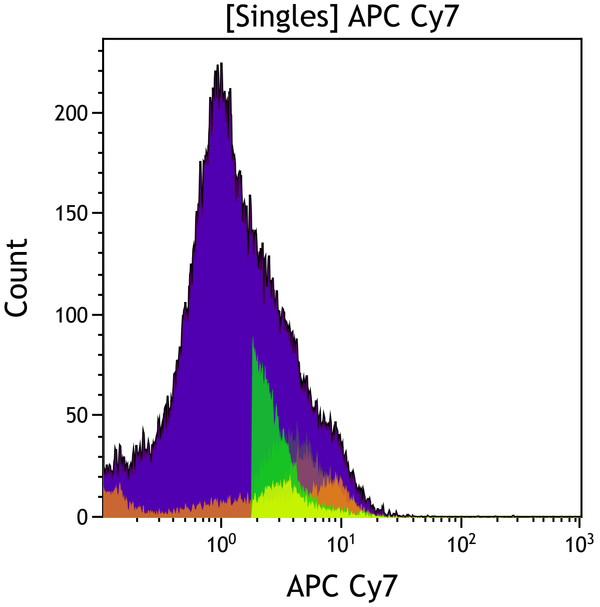

Gate	X-Med	Y-Med
All	3.62	7.86
B--	1.38	1.32
B-+	2.44	5.73
B+-	6.11	1.37
B++	6.23	11.74

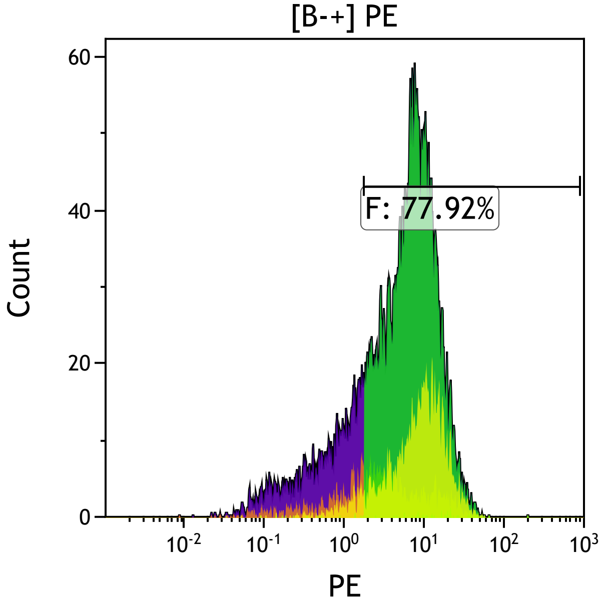

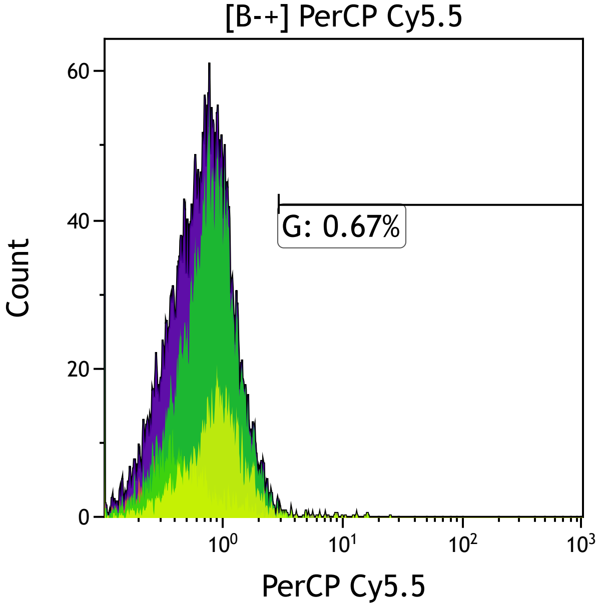

Gate	X-Med
All	5.79
F	7.49

Gate	X-Med
All	0.69
G	4.93

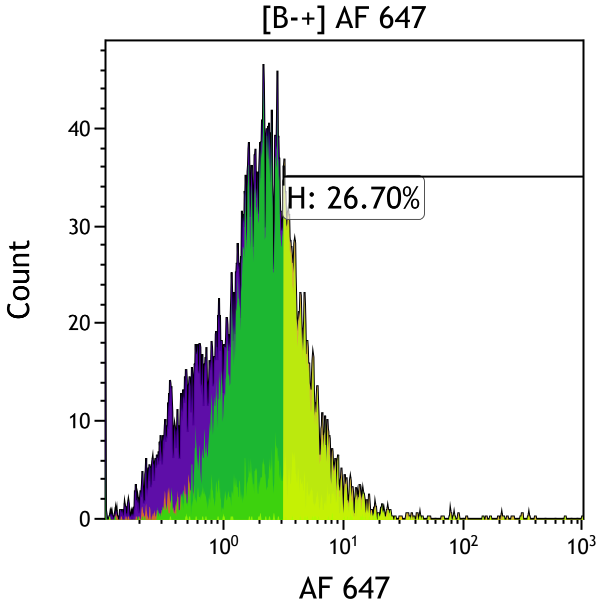

Gate	X-Med
All	1.95
H	4.55

3 contra-2017-03-14-162438-15
Rat microglia M1M2 protocol - Imported

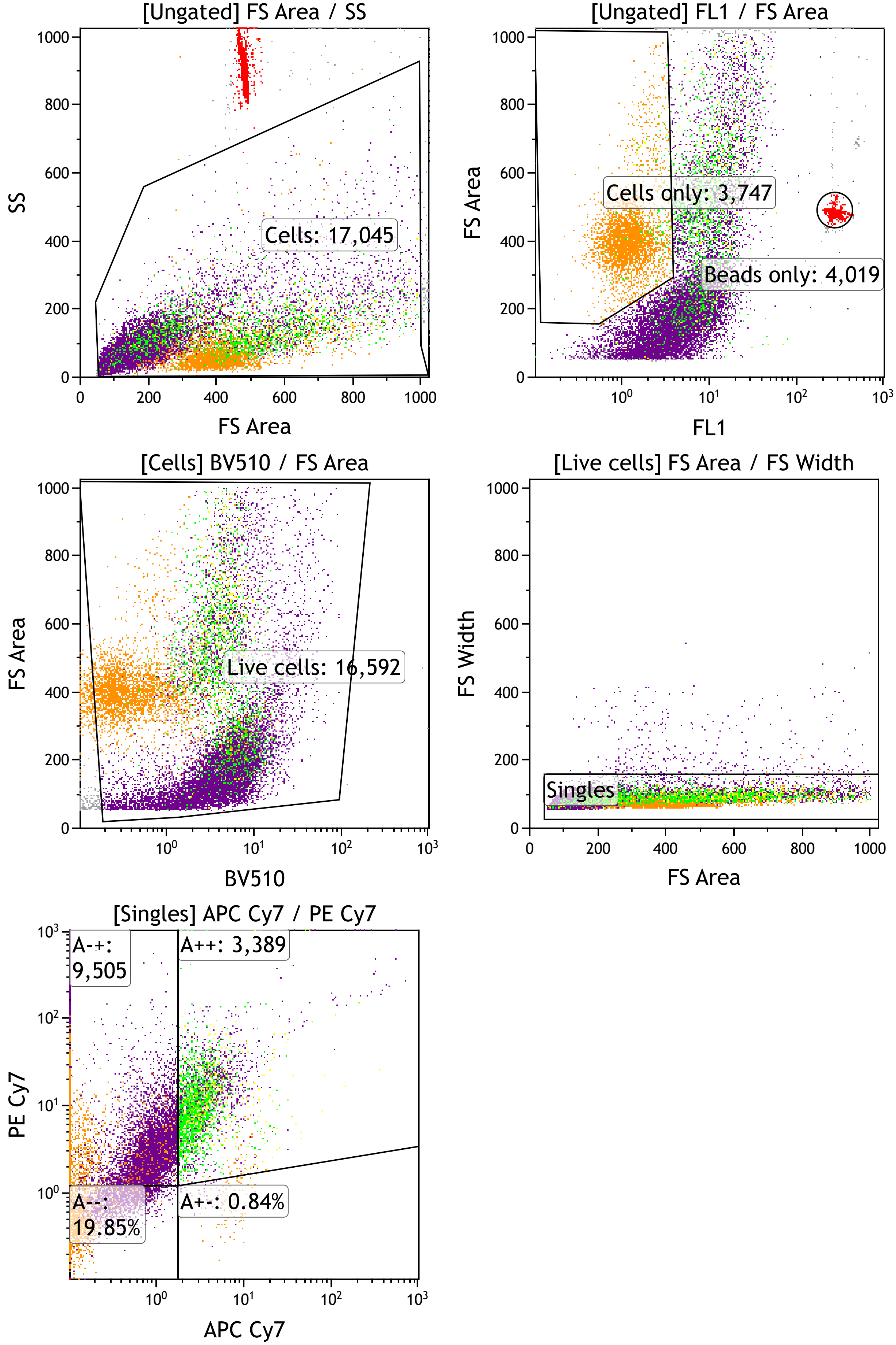

[B++] PE

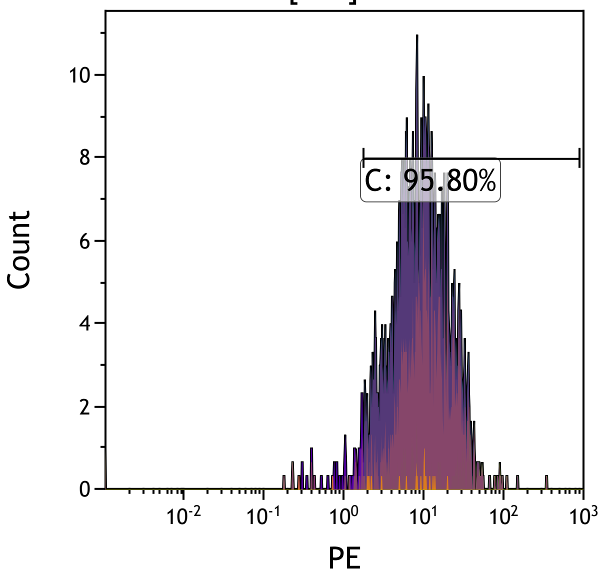

Gate	X-Med
All	8.96
C	9.37

[B++] PerCP Cy5.5

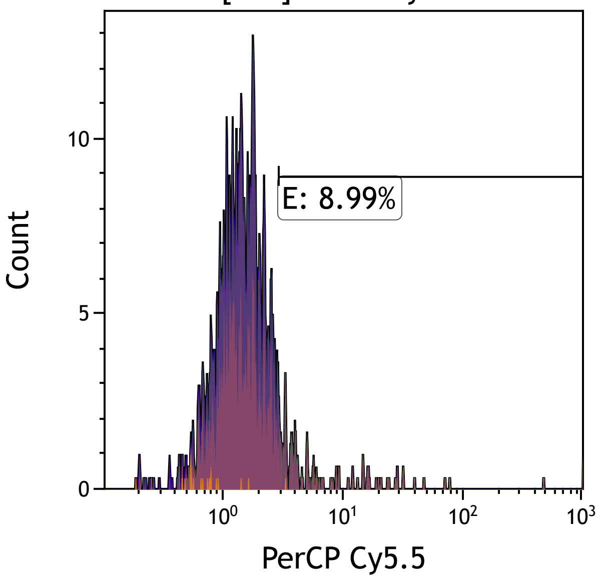

Gate	X-Med
All	1.46
E	4.18

[B++] AF 647

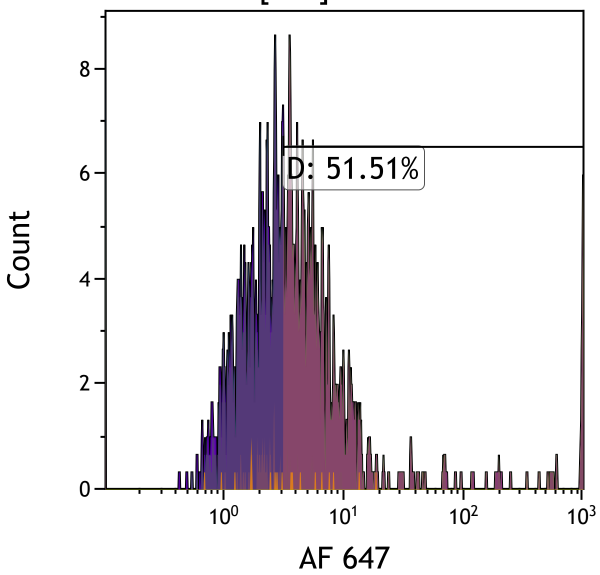

Gate	X-Med
All	3.27
D	5.57

[A++] BV421

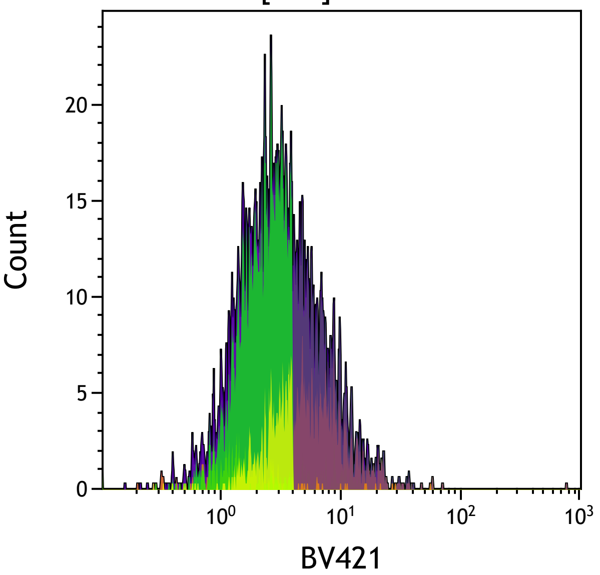

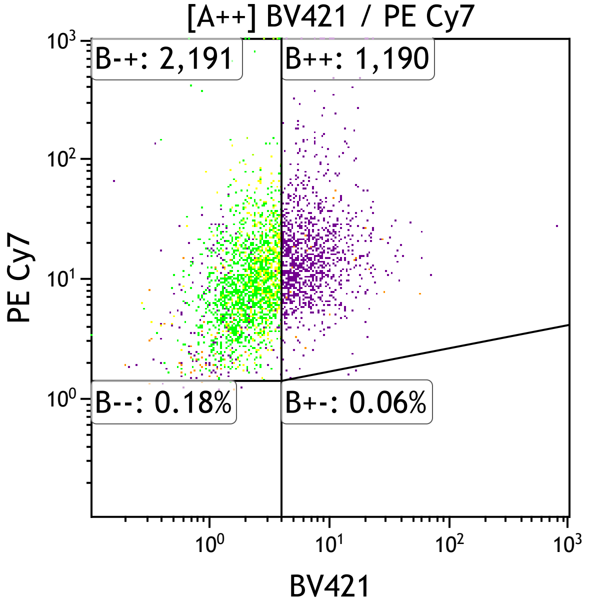

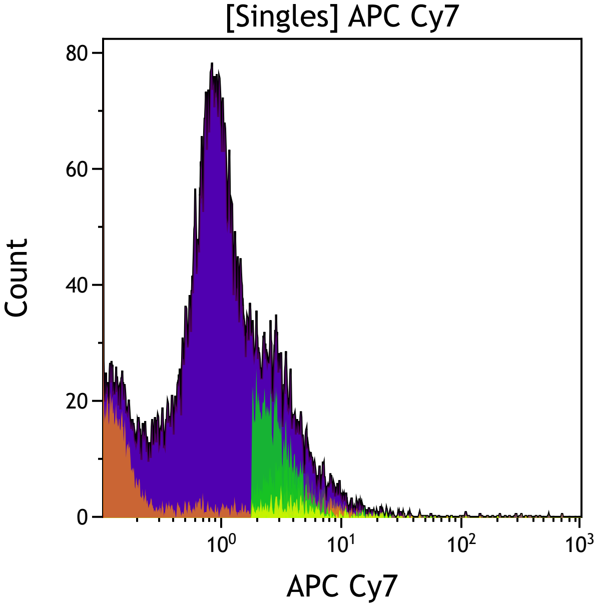

Gate	X-Med	Y-Med
All	3.01	10.56
B--	1.18	1.26
B-+	2.18	8.51
B+-	15.70	1.67
B++	6.52	14.81

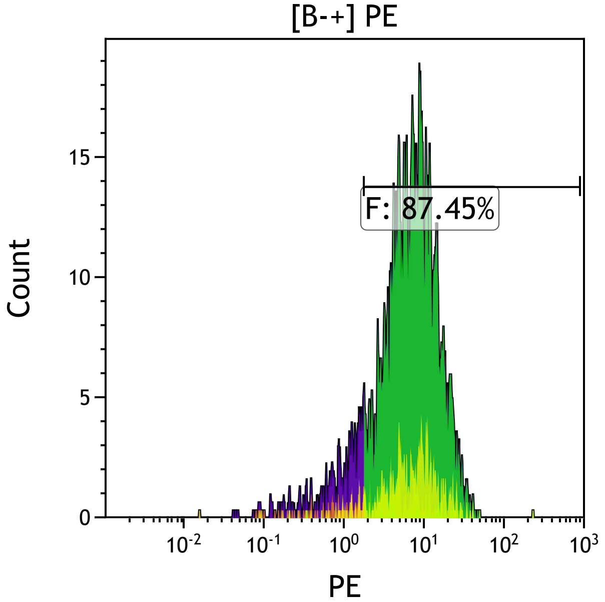

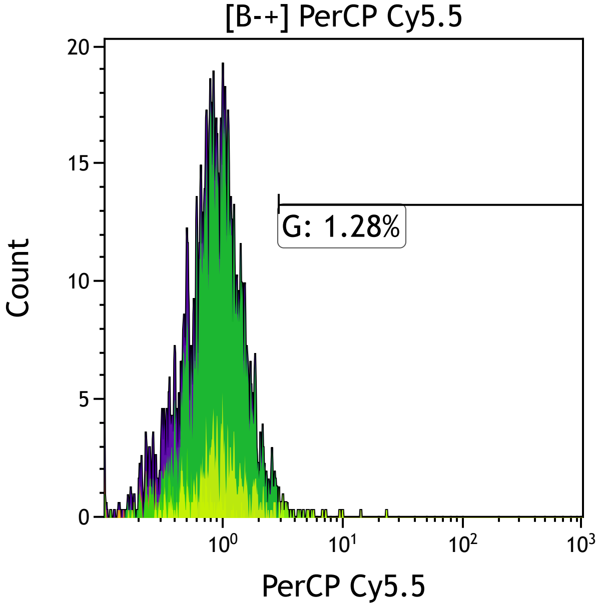

Gate	X-Med
All	6.58
F	7.48

Gate	X-Med
All	0.85
G	3.88

[B-+] AF 647

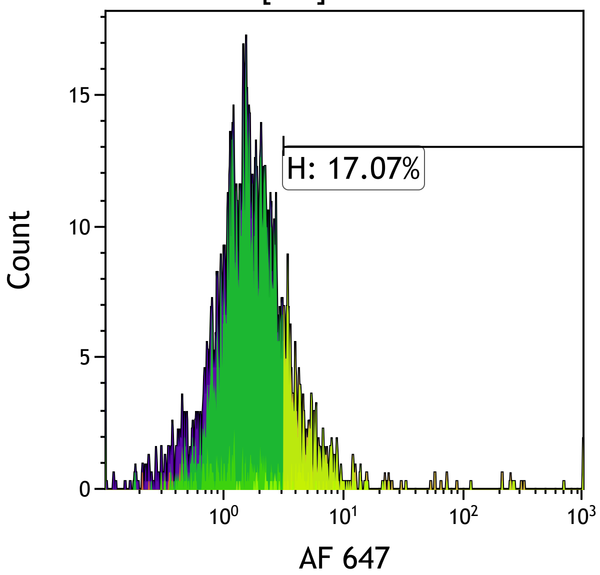

Gate	X-Med
All	1.64
H	4.56

4 contra-2017-03-14-162710-16
Rat microglia M1M2 protocol - Imported

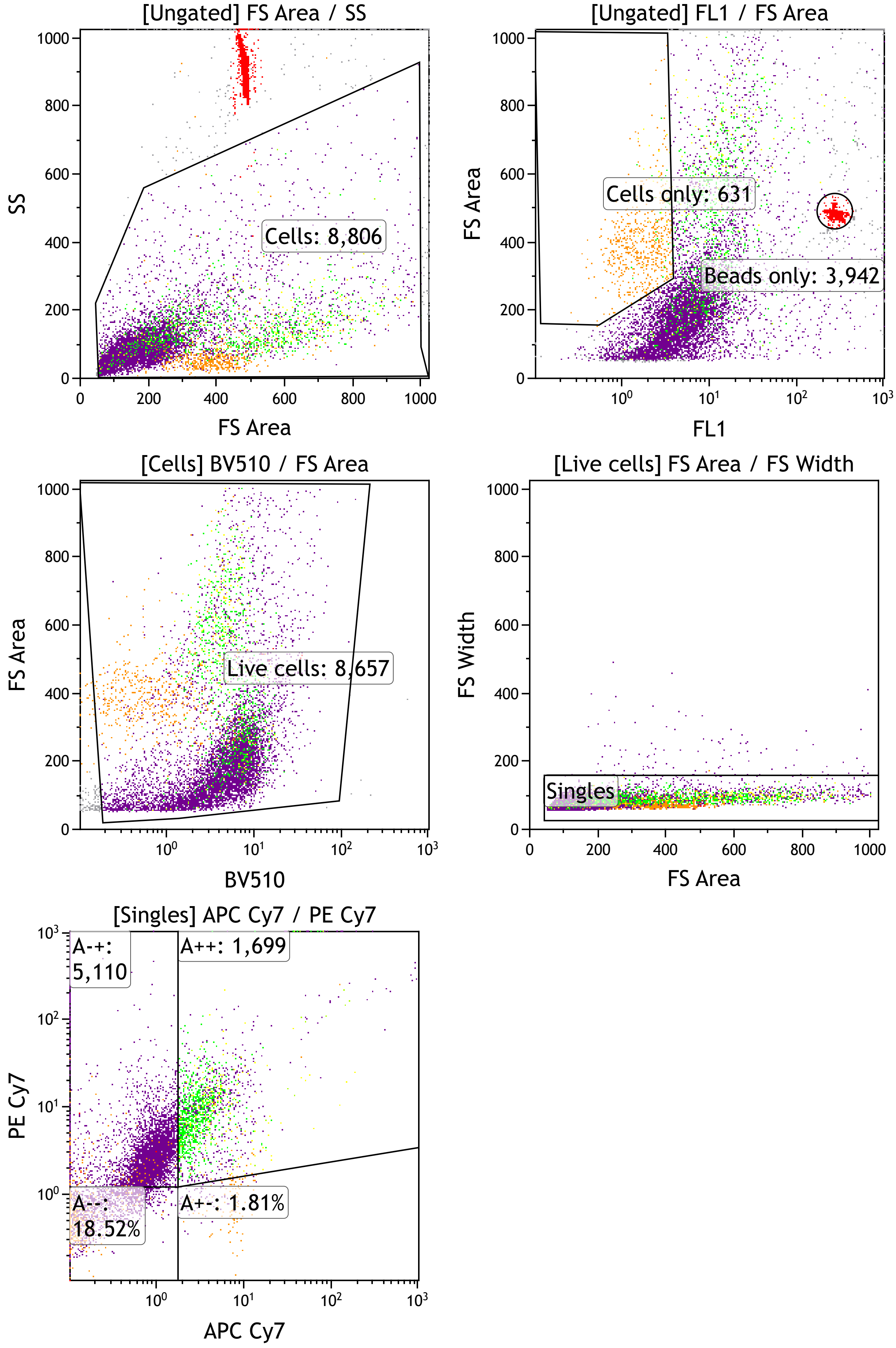

4 contra-2017-03-14-162710-16
Rat microglia M1M2 protocol - Imported

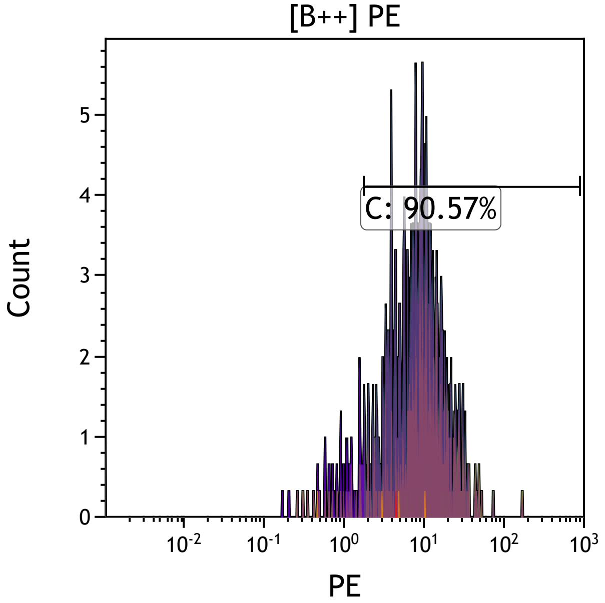

Gate	X-Med
All	7.90
C	8.65

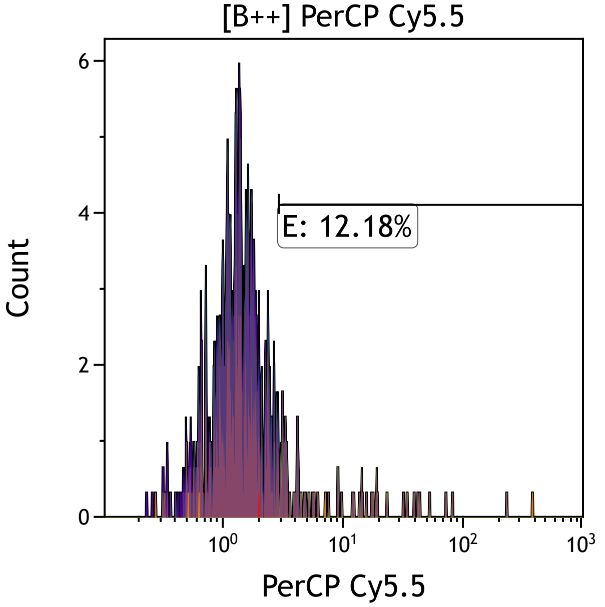

Gate	X-Med
All	1.39
E	4.90

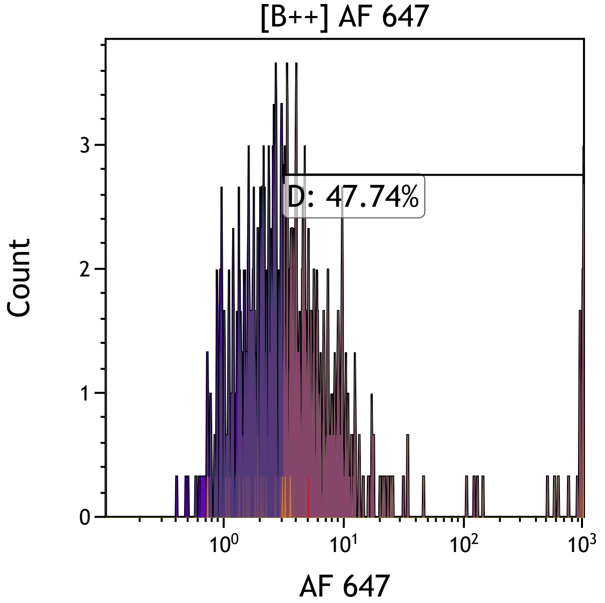

Gate	X-Med
All	3.02
D	5.89

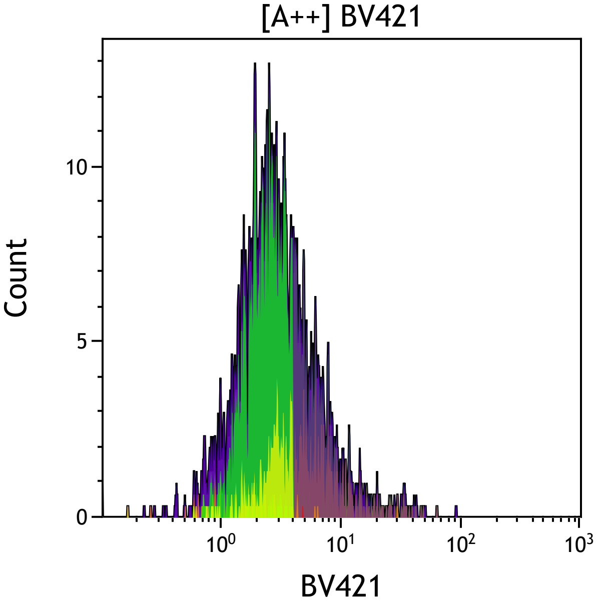

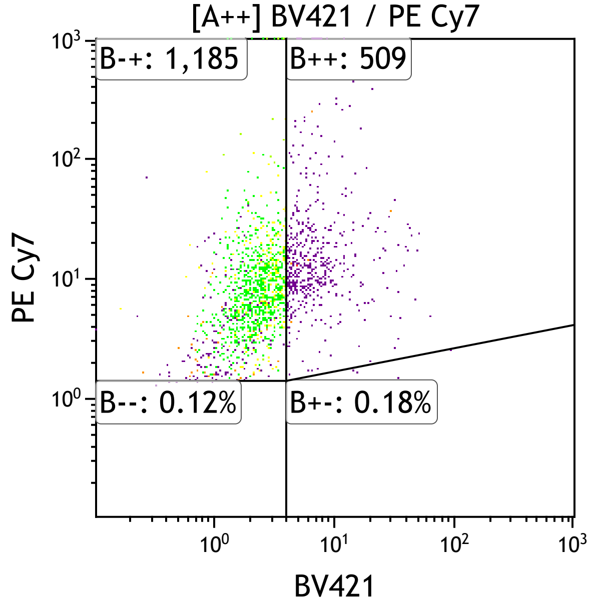

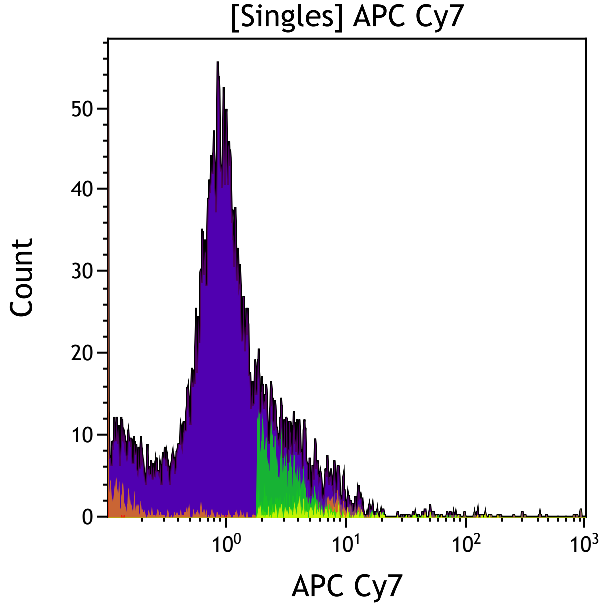

Gate	X-Med	Y-Med
All	2.78	8.63
B--	0.41	1.33
B-+	2.20	6.96
B+-	15.42	1.67
B++	6.15	12.95

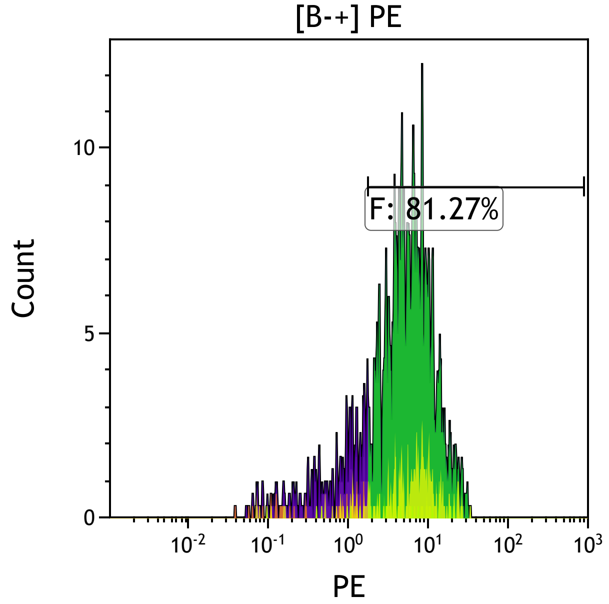

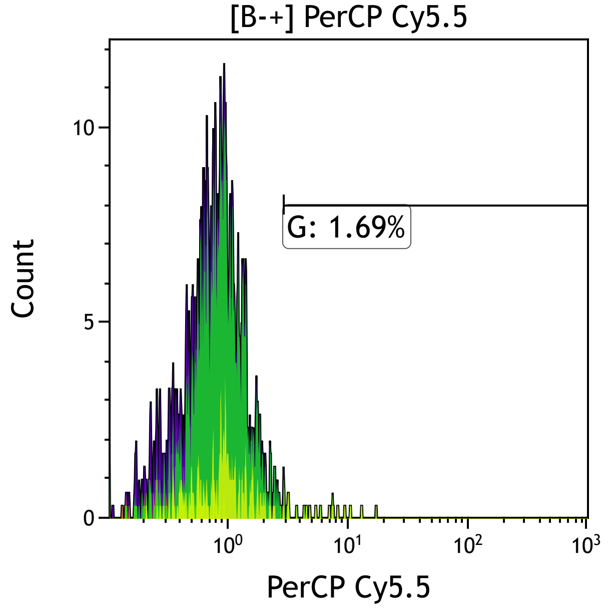

Gate	X-Med
All	4.89
F	6.05

Gate	X-Med
All	0.79
G	5.41

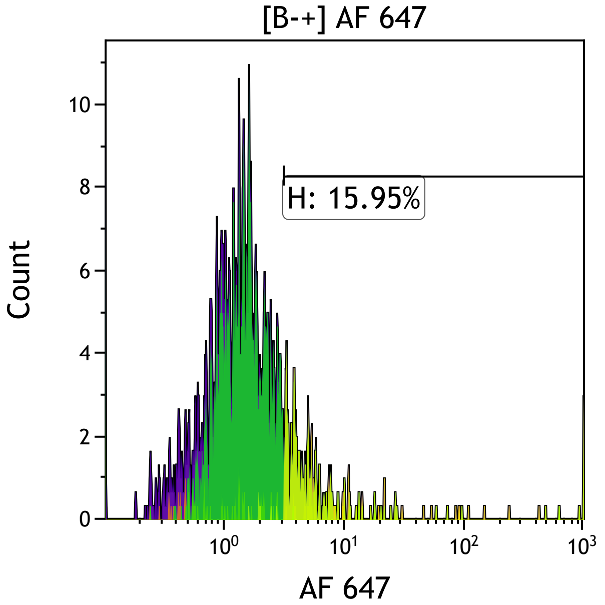

Gate	X-Med
All	1.46
H	5.08

5 contra-2017-03-14-162944-17

Rat microglia M1M2 protocol - Imported

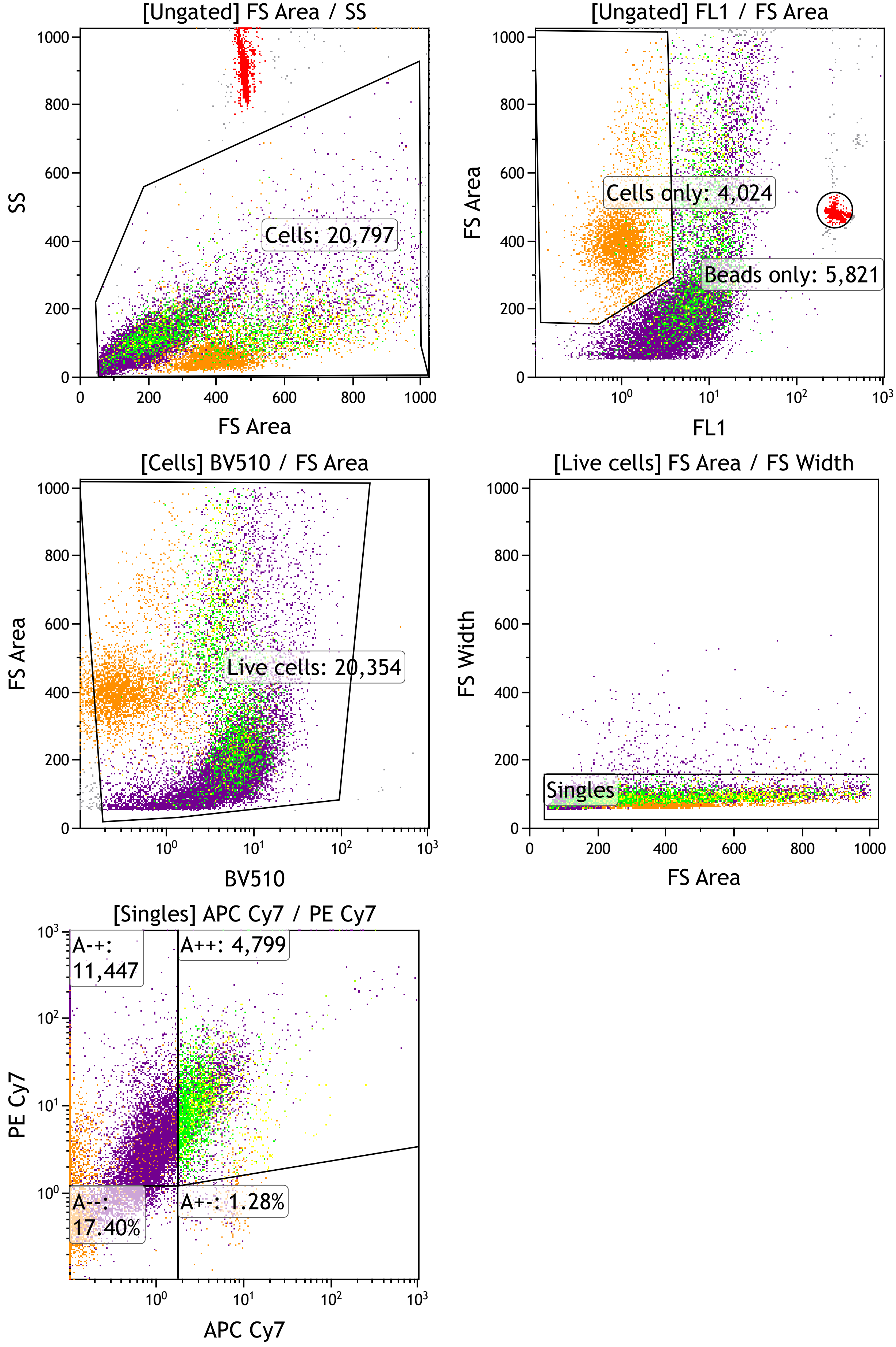

5 contra-2017-03-14-162944-17

Rat microglia M1M2 protocol - Imported

[B++] PE

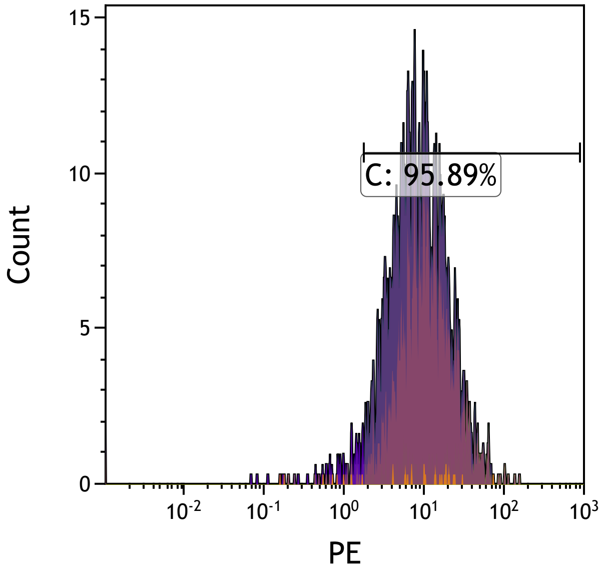

Gate	X-Med
All	8.33
C	8.79

[B++] PerCP Cy5.5

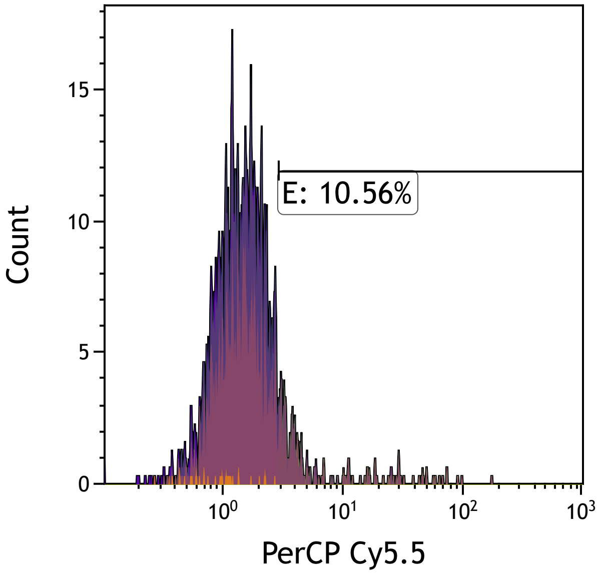

Gate	X-Med
All	1.49
E	4.02

[B++] AF 647

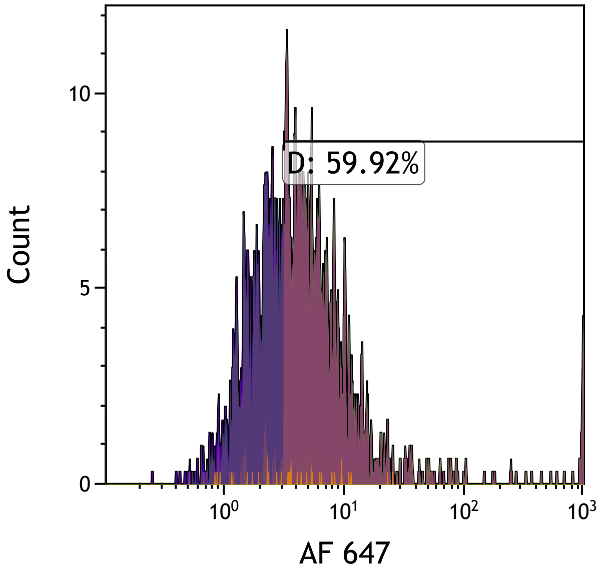

Gate	X-Med
All	3.86
D	5.94

[A++] BV421

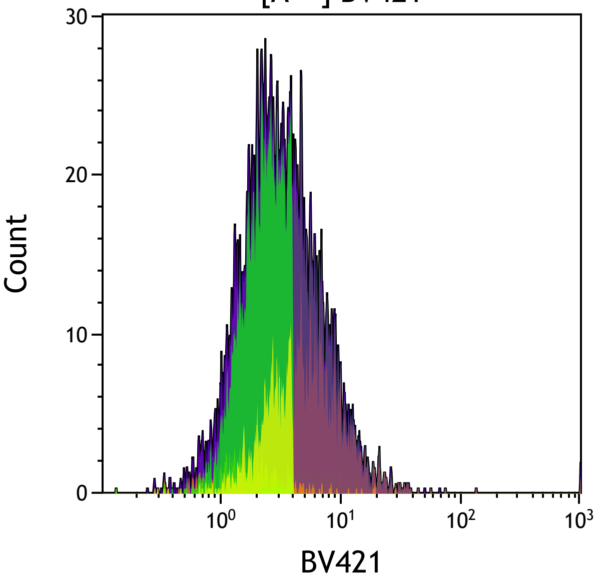

5 contra-2017-03-14-162944-17
Rat microglia M1M2 protocol - Imported

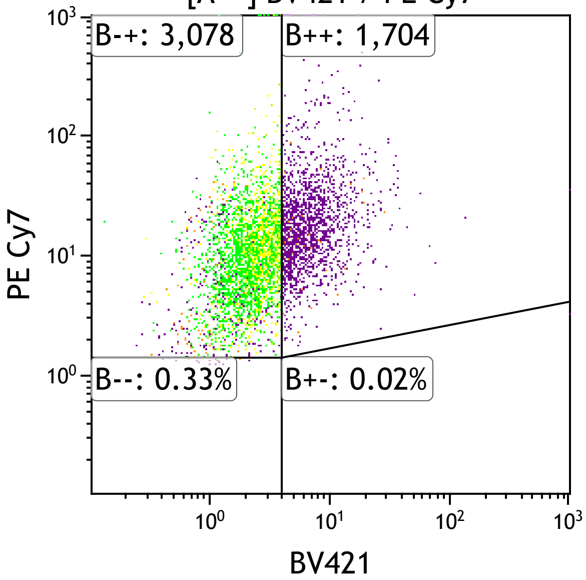

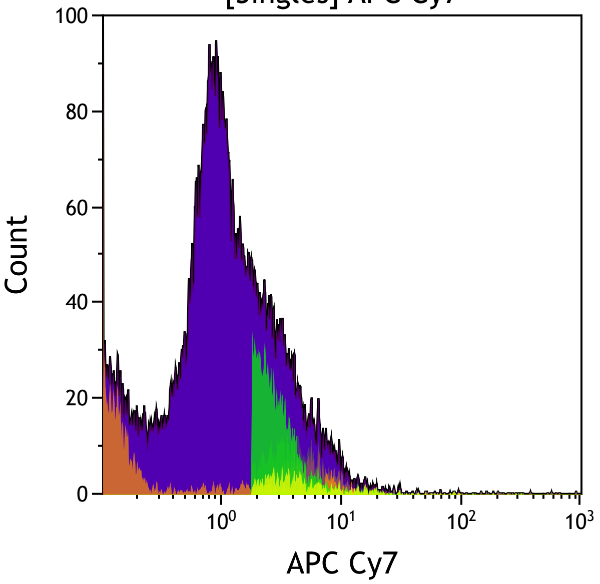

Gate	X-Med	Y-Med
All	3.03	11.72
B--	1.19	1.35
B-+	2.20	9.46
B+-	1,014.08	3.26
B++	6.28	17.31

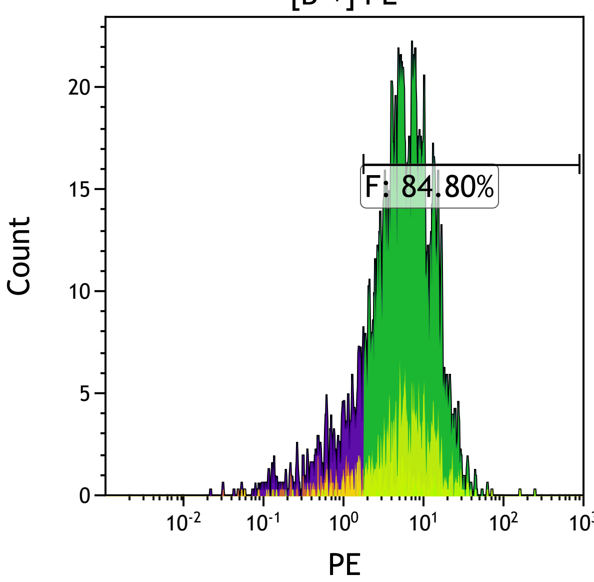

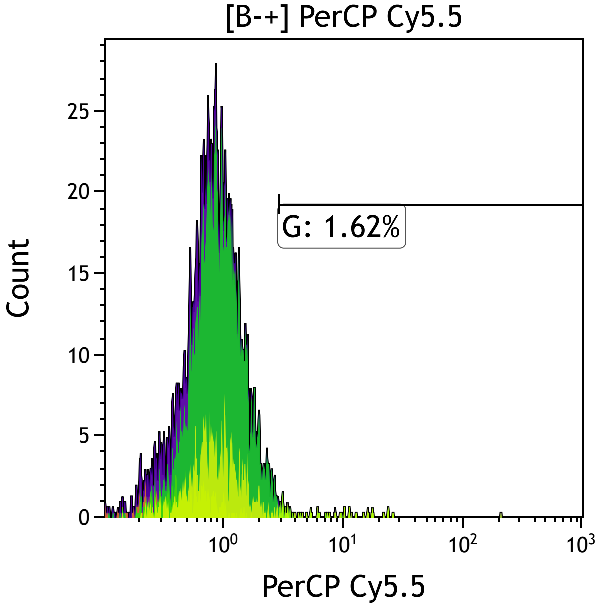

Gate	X-Med
All	5.38
F	6.43

Gate	X-Med
All	0.84
G	6.19

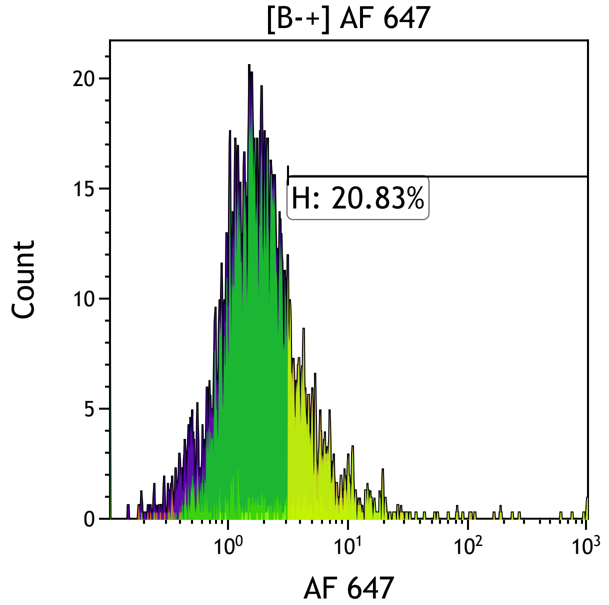

Gate	X-Med
All	1.74
H	4.93

6 contra-2017-03-14-163213-18
Rat microglia M1M2 protocol - Imported

[Ungated] FS Area / SS

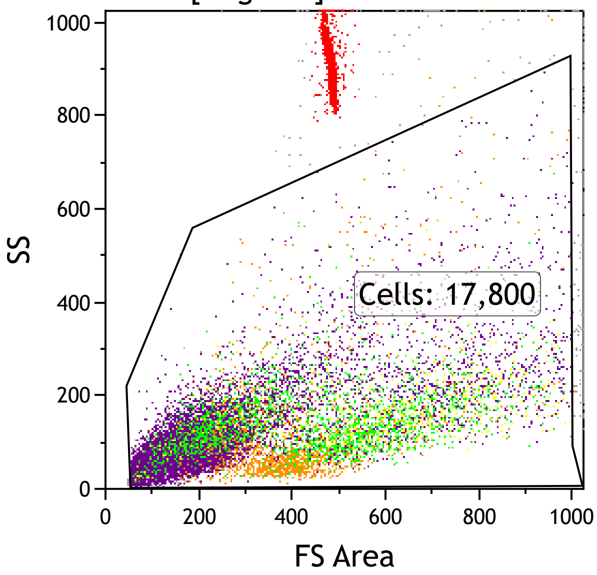

[Ungated] FL1 / FS Area

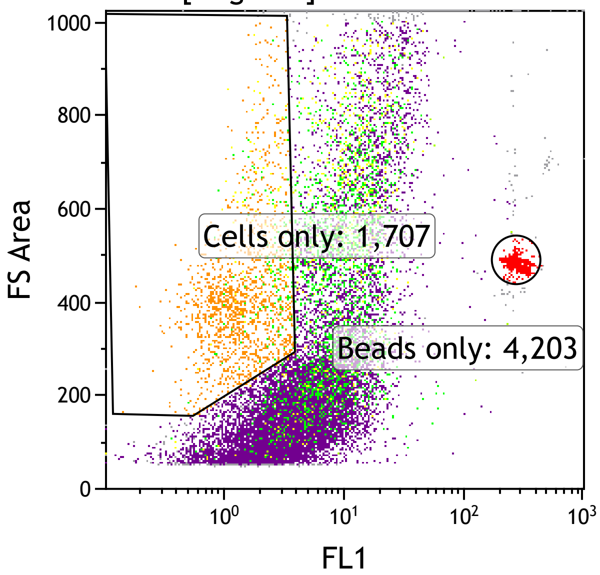

[Cells] BV510 / FS Area

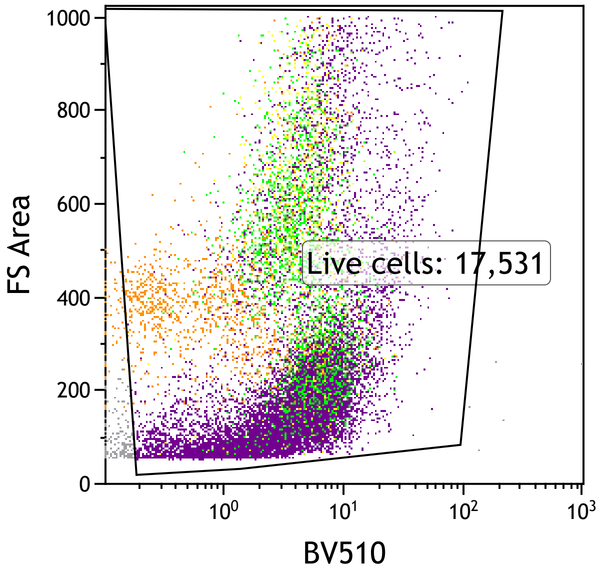

[Live cells] FS Area / FS Width

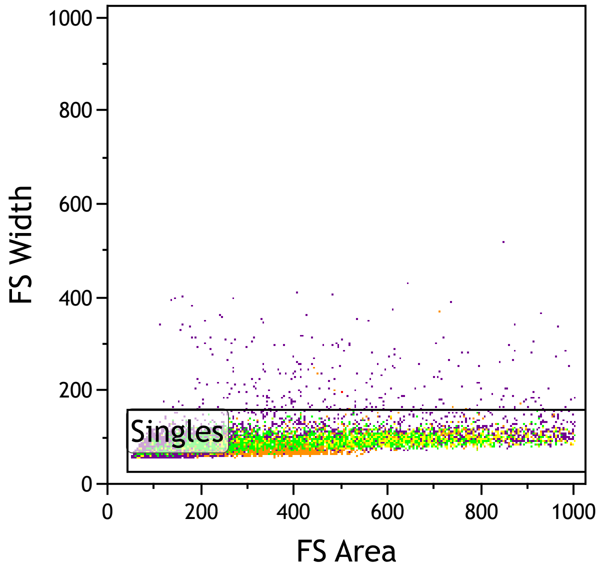

[Singles] APC Cy7 / PE Cy7

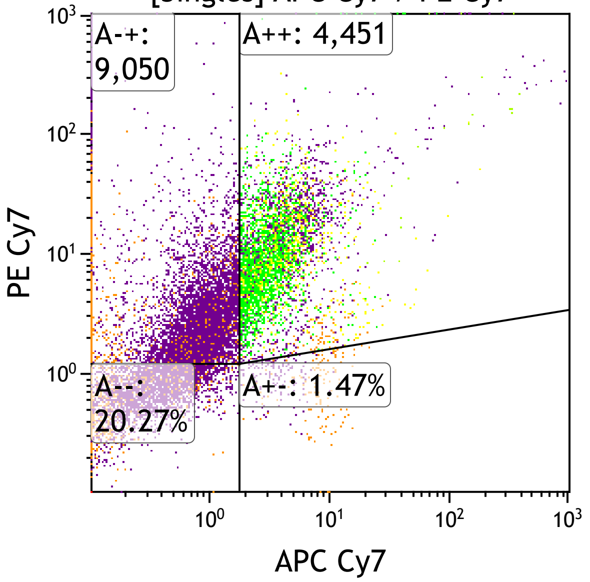

6 contra-2017-03-14-163213-18
Rat microglia M1M2 protocol - Imported

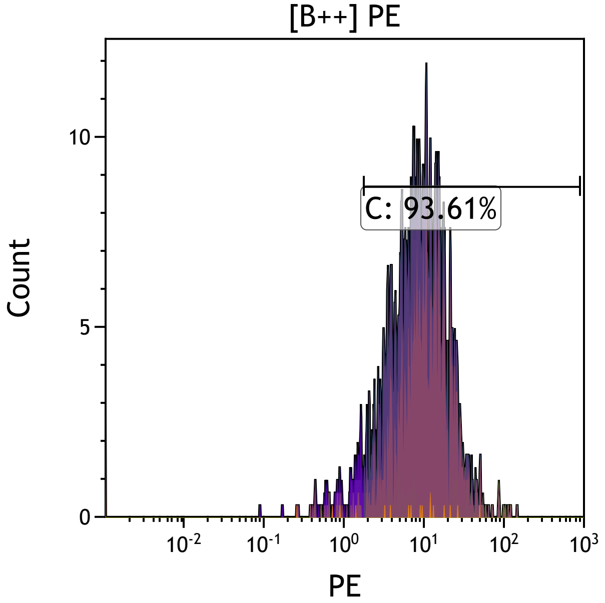

Gate	X-Med
All	8.57
C	9.09

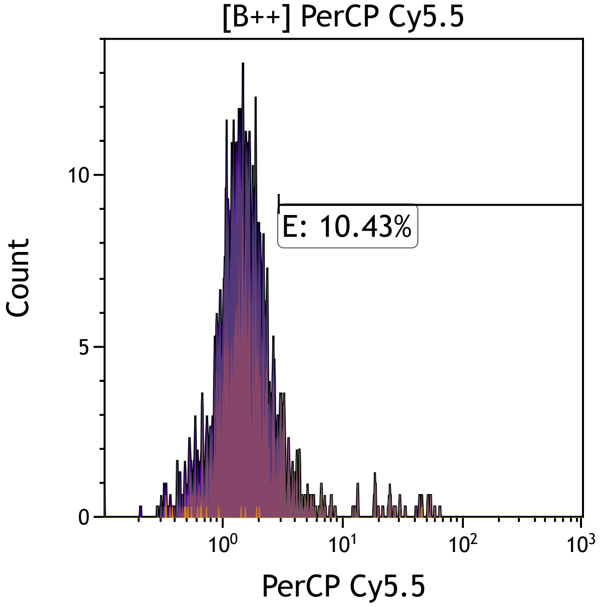

Gate	X-Med
All	1.48
E	4.14

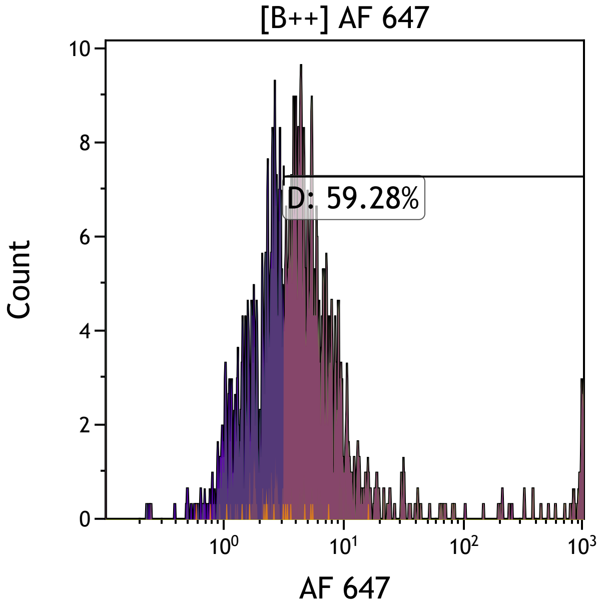

Gate	X-Med
All	3.82
D	5.47

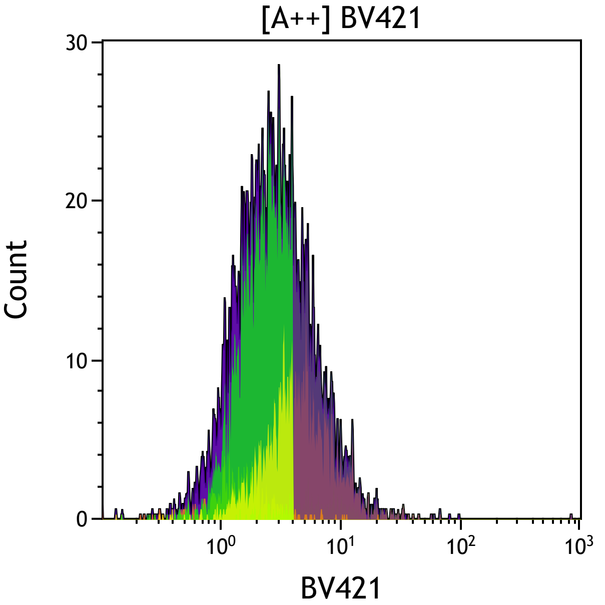

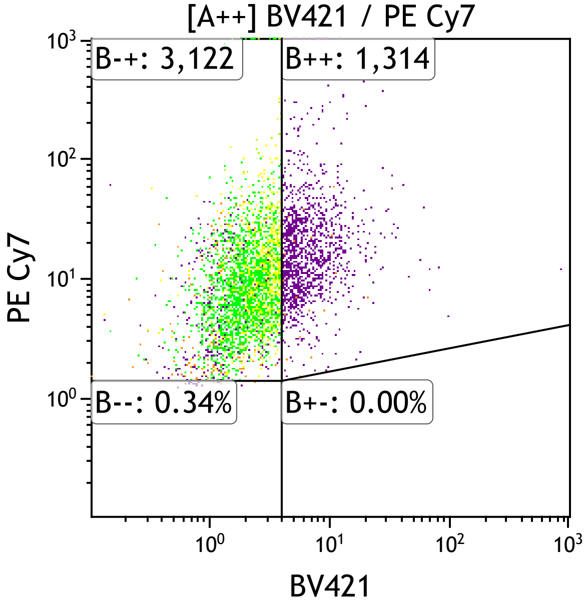

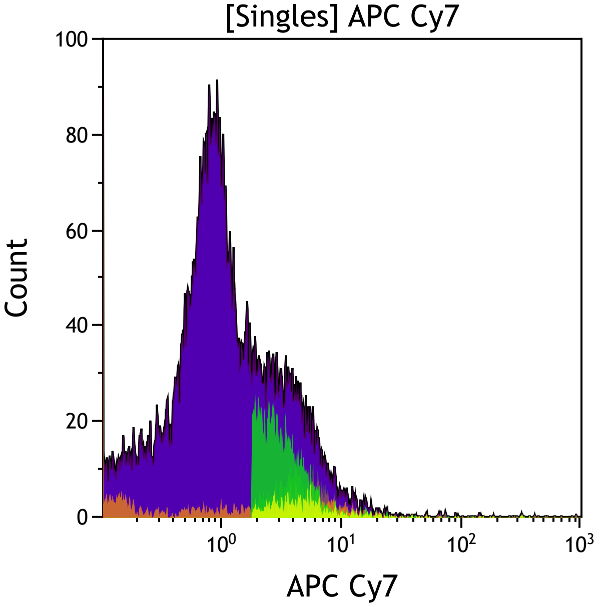

Gate	X-Med	Y-Med
All	2.74	9.94
B--	0.78	1.35
B-+	2.09	8.08
B+-	N/A	N/A
B++	5.93	15.53

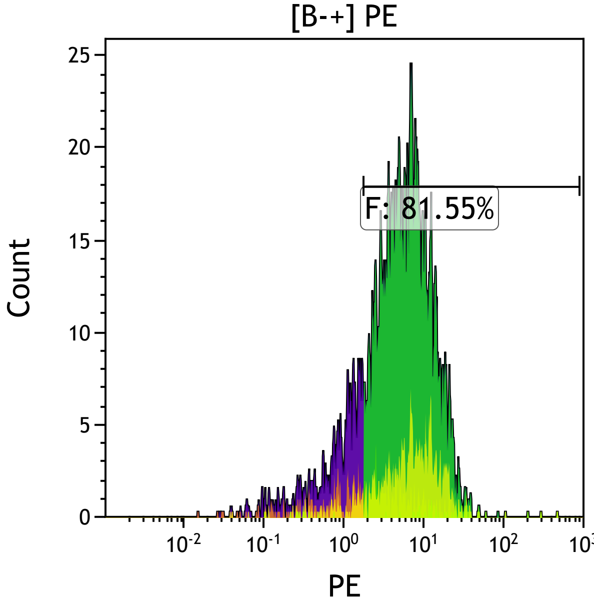

Gate	X-Med
All	5.03
F	6.37

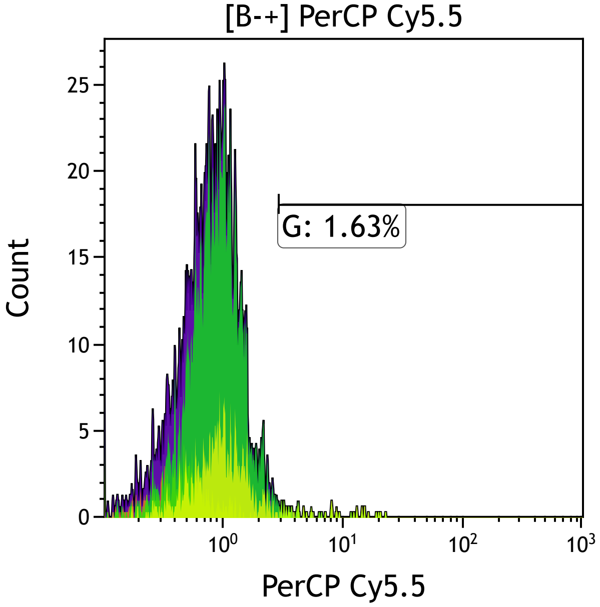

Gate	X-Med
All	0.81
G	5.43

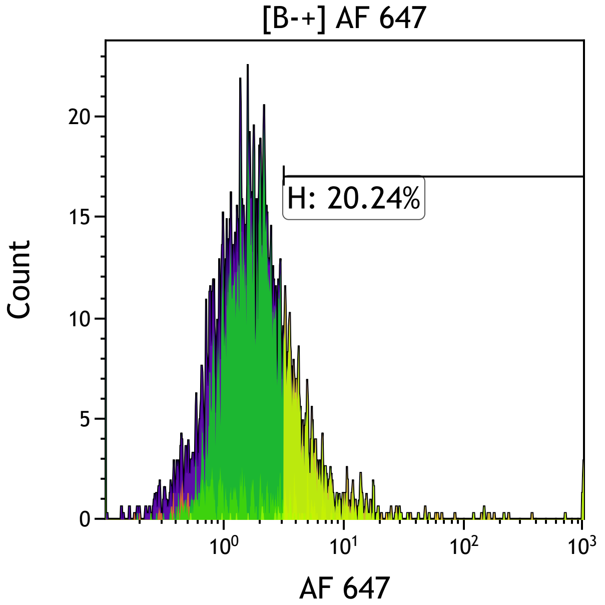

Gate	X-Med
All	1.68
H	4.69

7 contra-2017-03-14-163448-19

Rat microglia M1M2 protocol - Imported

[Ungated] FS Area / SS

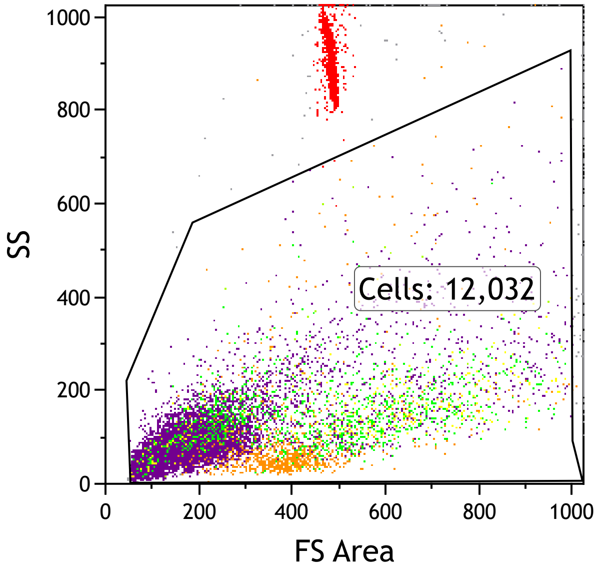

[Ungated] FL1 / FS Area

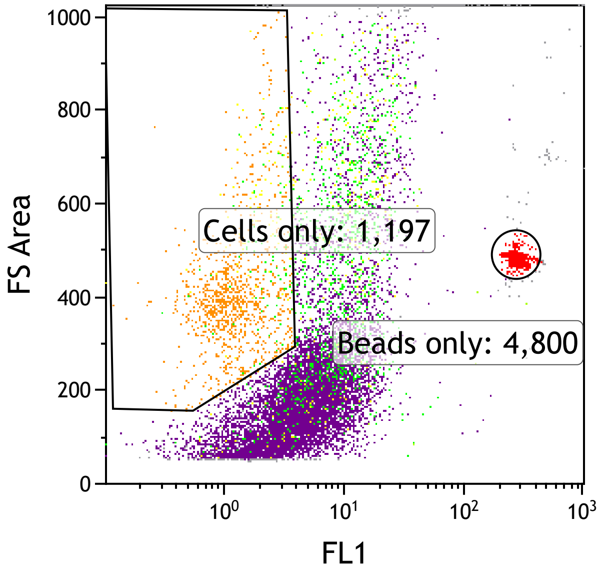

[Cells] BV510 / FS Area

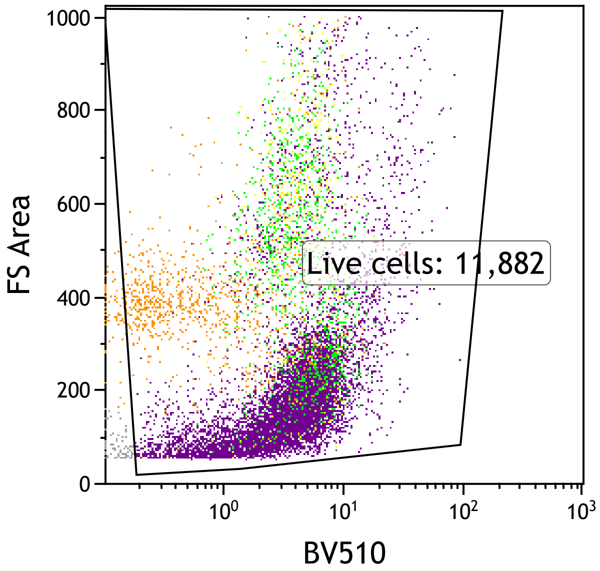

[Live cells] FS Area / FS Width

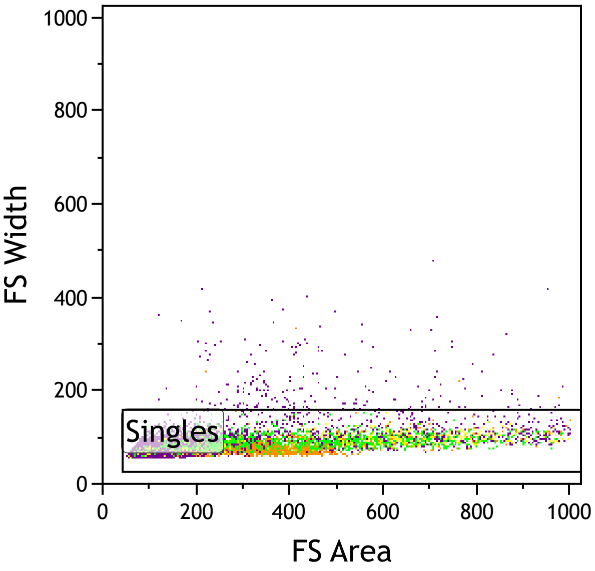

[Singles] APC Cy7 / PE Cy7

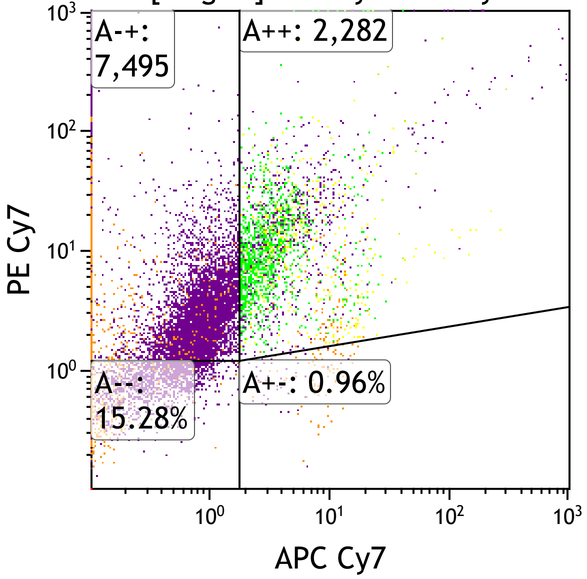

7 contra-2017-03-14-163448-19
Rat microglia M1M2 protocol - Imported

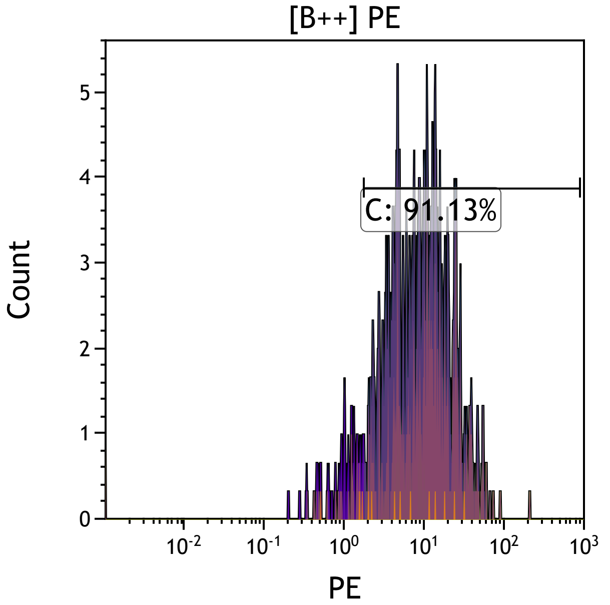

Gate	X-Med
All	8.39
C	9.51

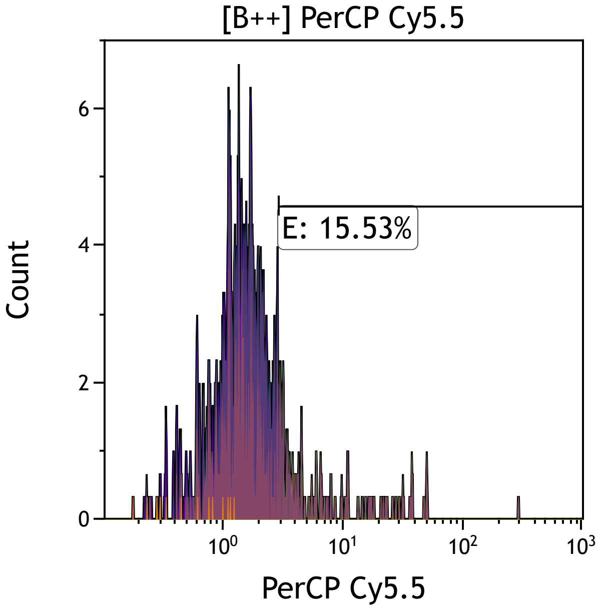

Gate	X-Med
All	1.54
E	5.22

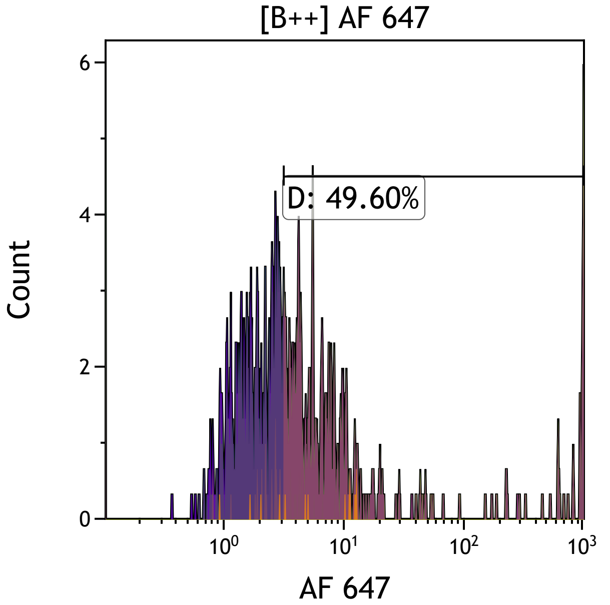

Gate	X-Med
All	3.13
D	6.74

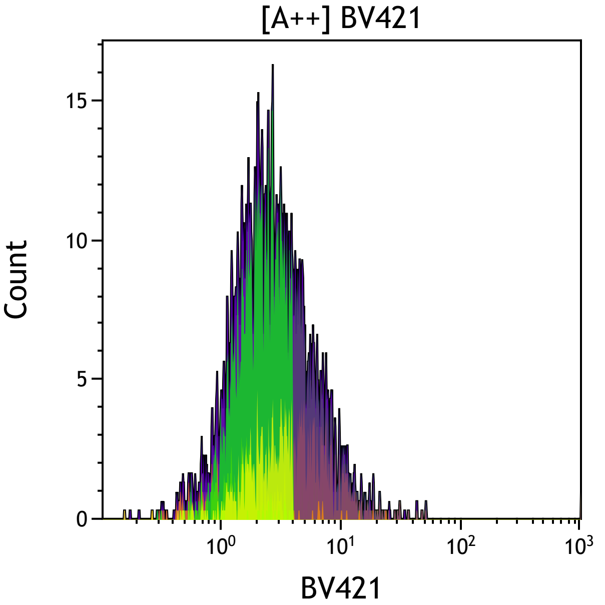

7 contra-2017-03-14-163448-19
Rat microglia M1M2 protocol - Imported

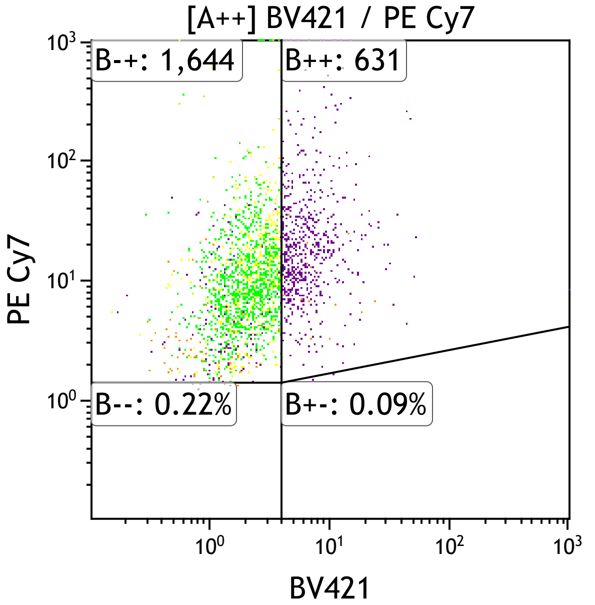

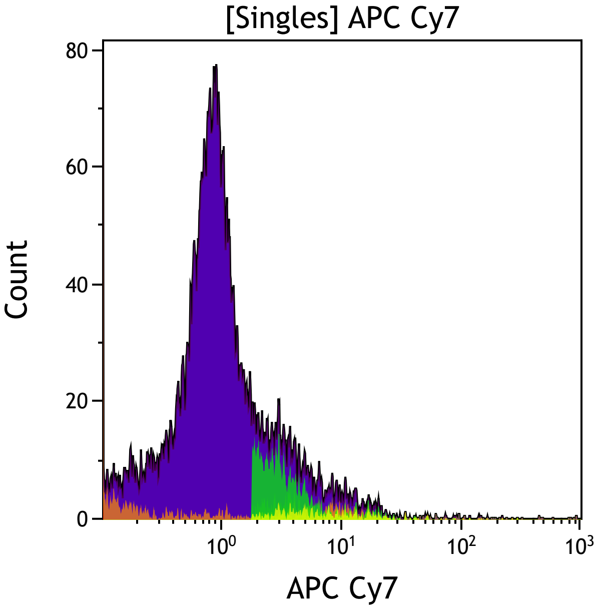

Gate	X-Med	Y-Med
All	2.58	10.56
B--	1.49	1.36
B-+	2.02	8.78
B+-	9.03	1.57
B++	6.03	17.23

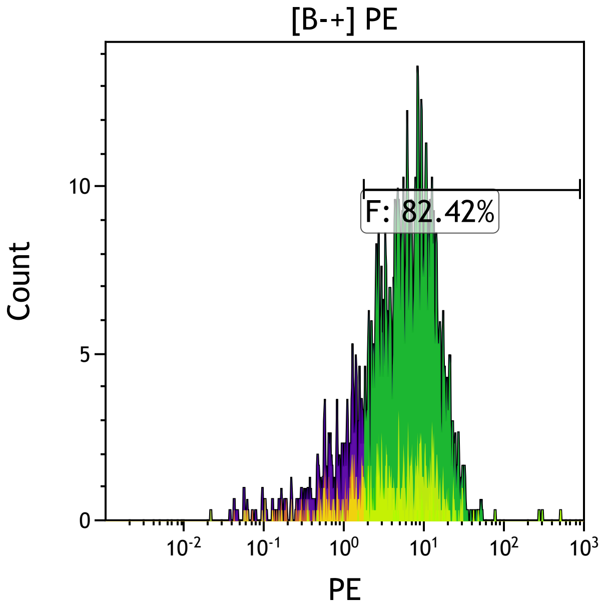

Gate	X-Med
All	5.61
F	7.09

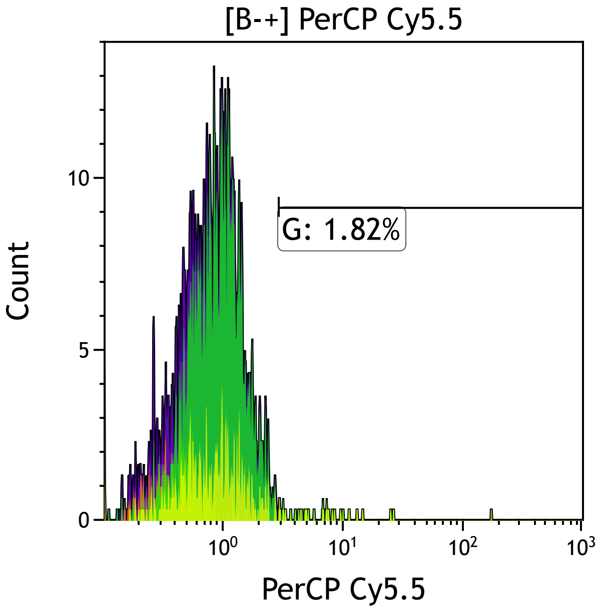

Gate	X-Med
All	0.82
G	6.77

[B-+] AF 647

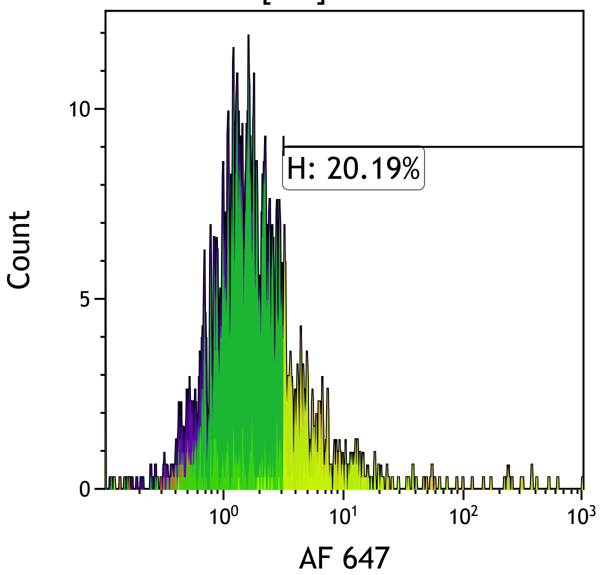

Gate	X-Med
All	1.63
H	5.46

8 contra-2017-03-14-163722-20

Rat microglia M1M2 protocol - Imported

[Ungated] FS Area / SS

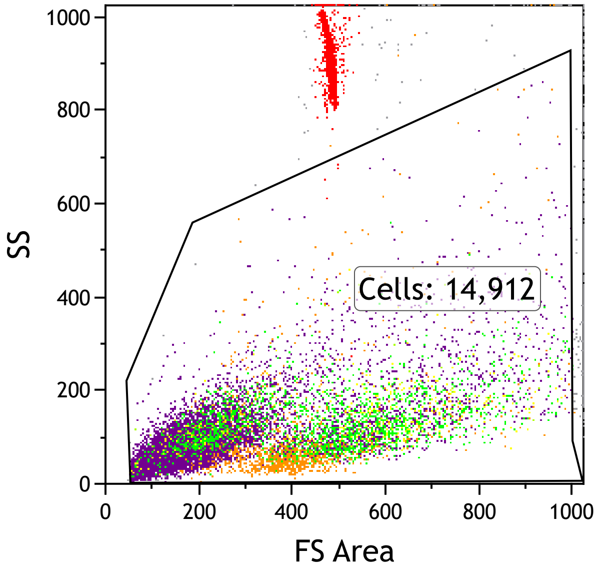

[Ungated] FL1 / FS Area

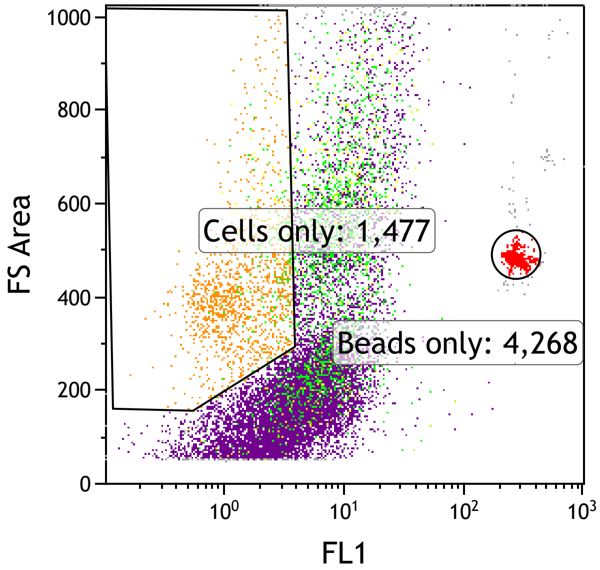

[Cells] BV510 / FS Area

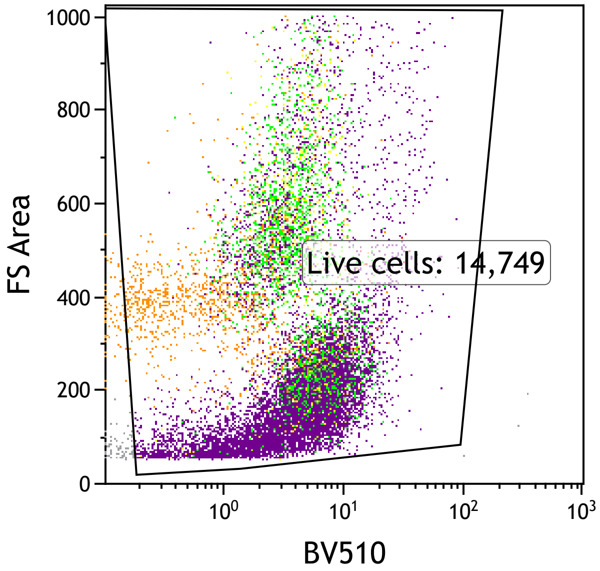

[Live cells] FS Area / FS Width

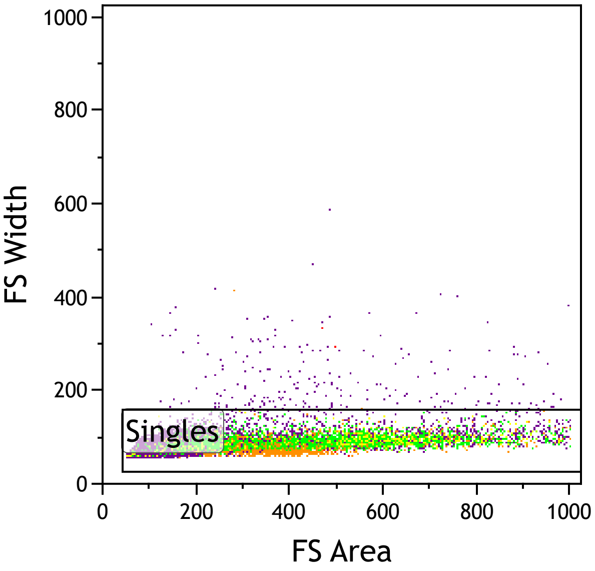

[Singles] APC Cy7 / PE Cy7

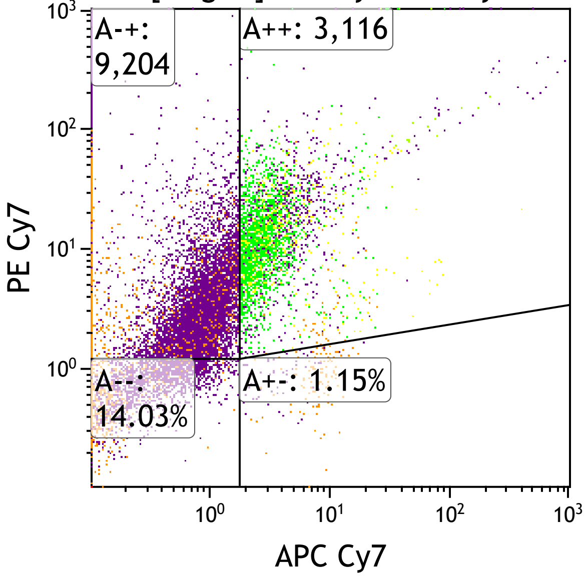

8 contra-2017-03-14-163722-20

Rat microglia M1M2 protocol - Imported

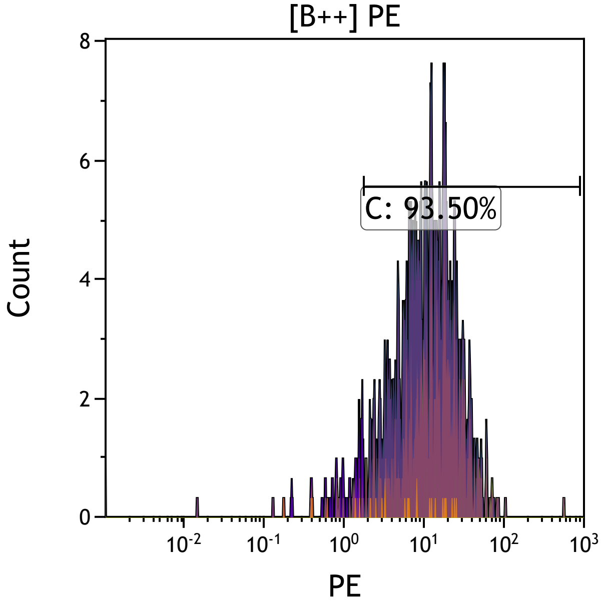

Gate	X-Med
All	10.78
C	11.87

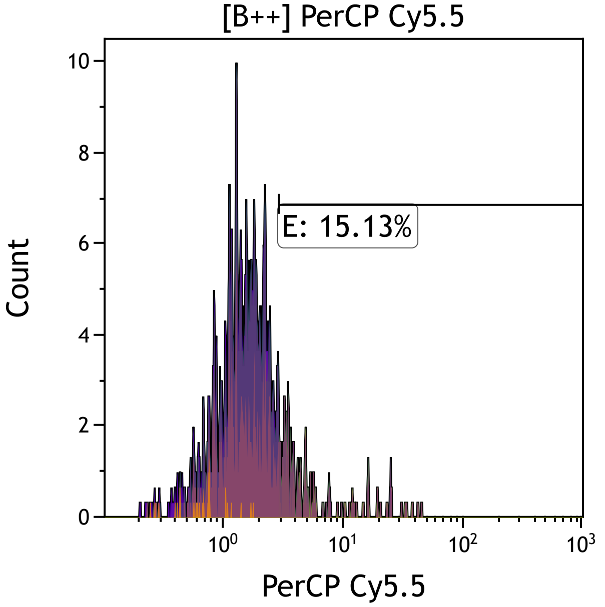

Gate	X-Med
All	1.61
E	4.21

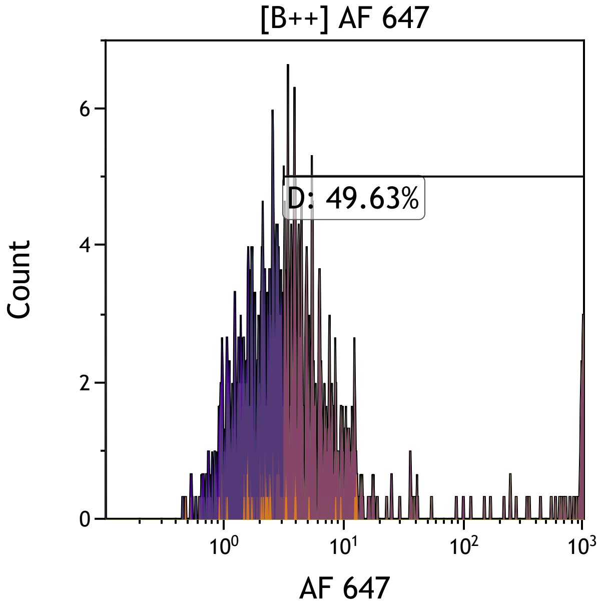

Gate	X-Med
All	3.10
D	5.66

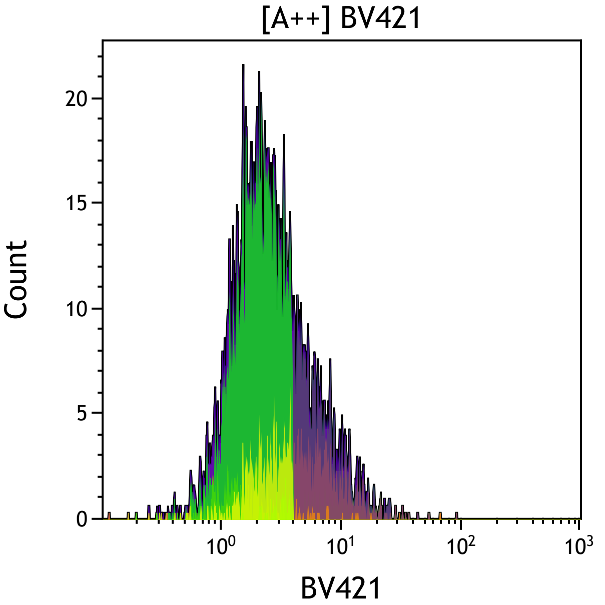

8 contra-2017-03-14-163722-20

Rat microglia M1M2 protocol - Imported

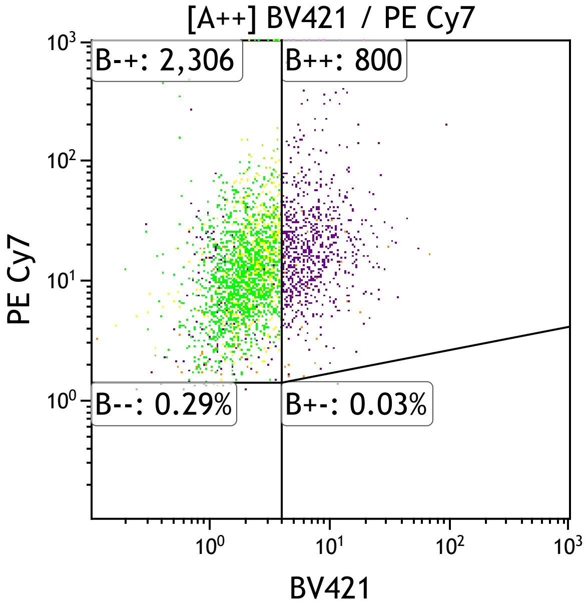

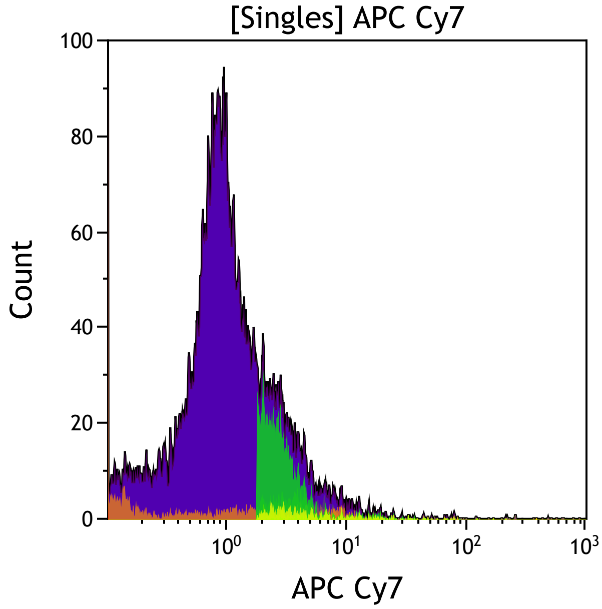

Gate	X-Med	Y-Med
All	2.45	12.32
B--	0.93	1.34
B-+	1.99	10.78
B+-	11.56	1.40
B++	6.45	17.72

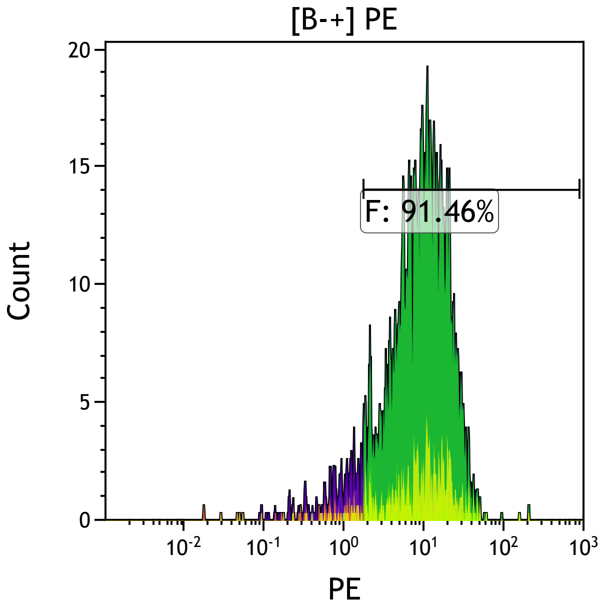

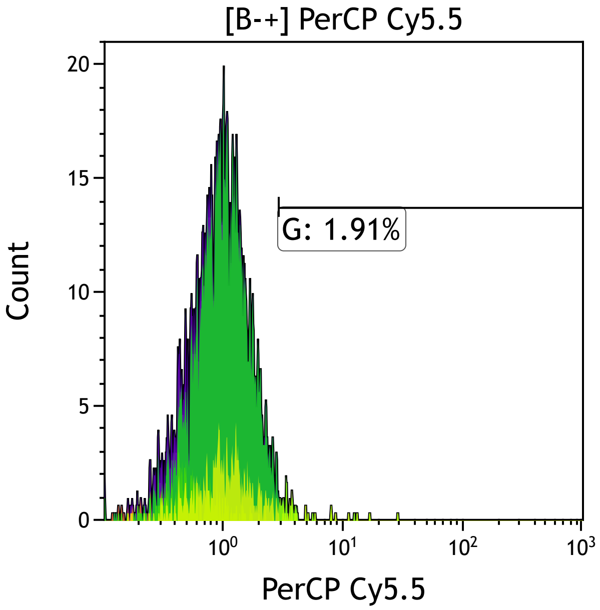

Gate	X-Med
All	9.31
F	10.12

Gate	X-Med
All	0.96
G	3.74

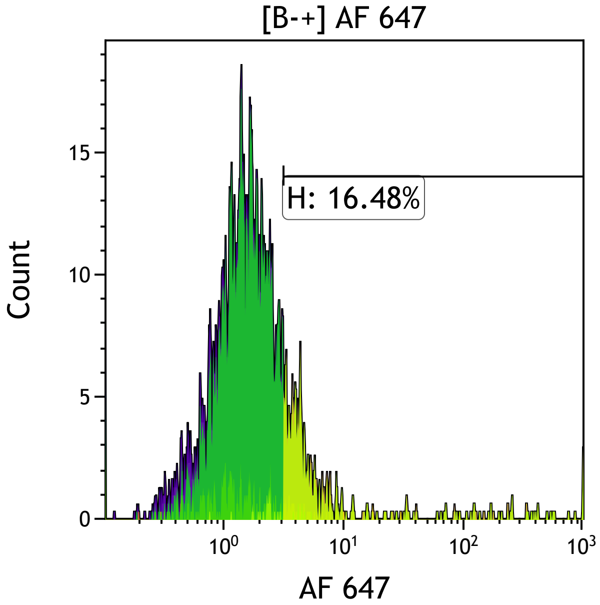

Gate	X-Med
All	1.63
H	4.54

9 contra-2017-03-14-163954-21

Rat microglia M1M2 protocol - Imported

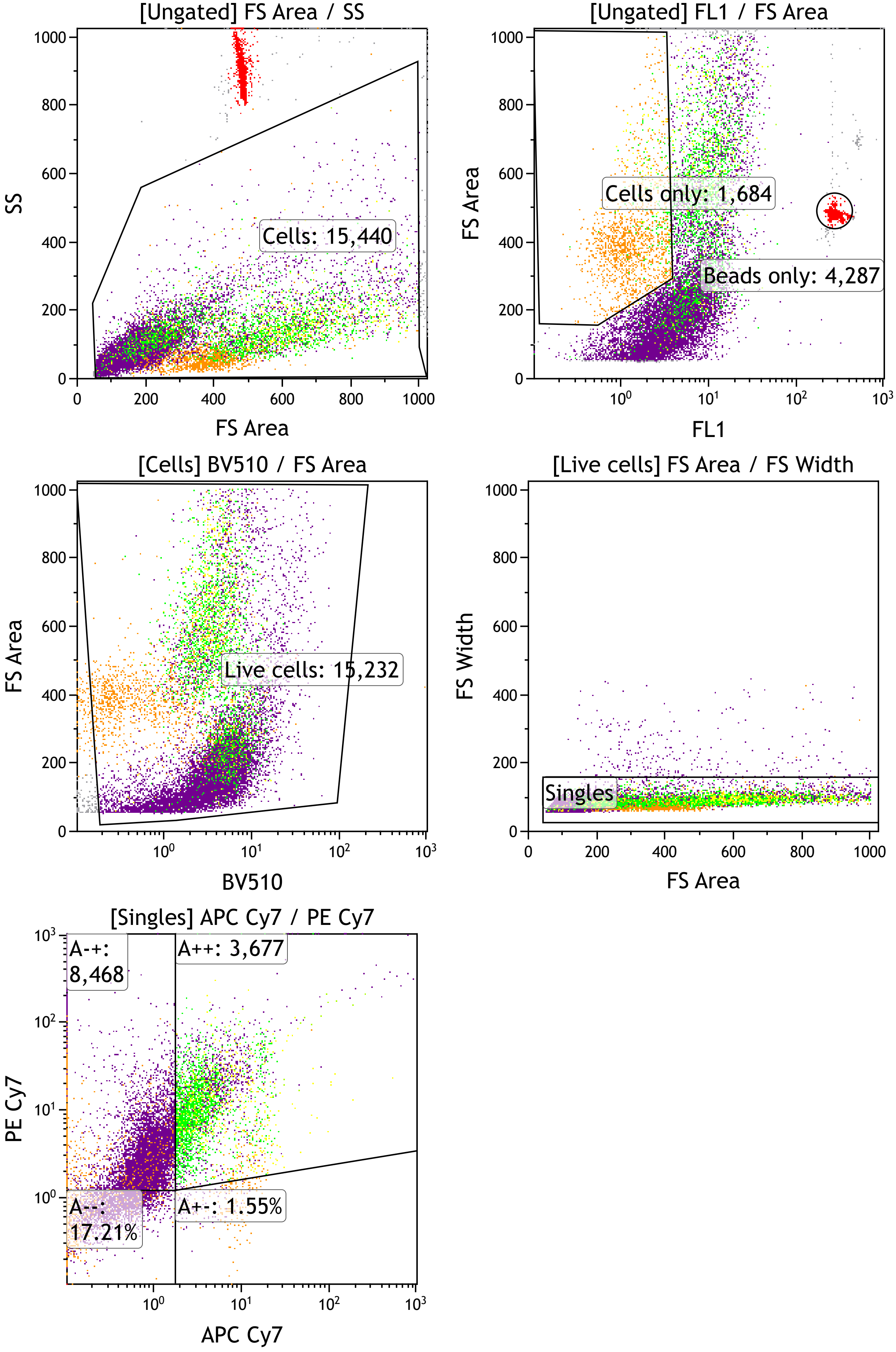

[B++] PE

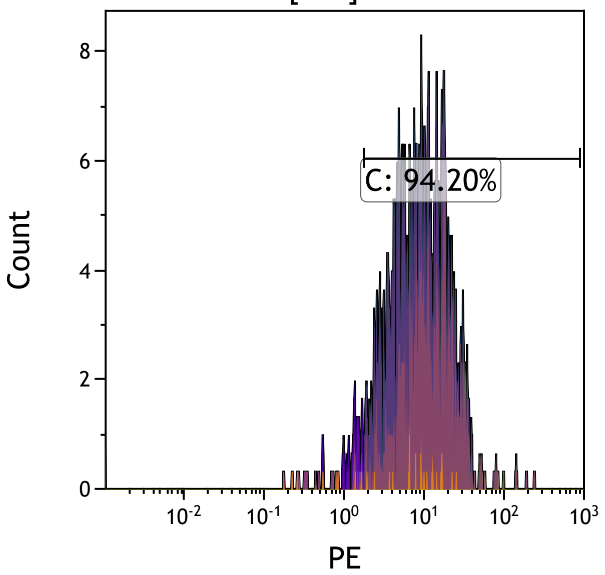

Gate	X-Med
All	8.69
C	9.23

[B++] PerCP Cy5.5

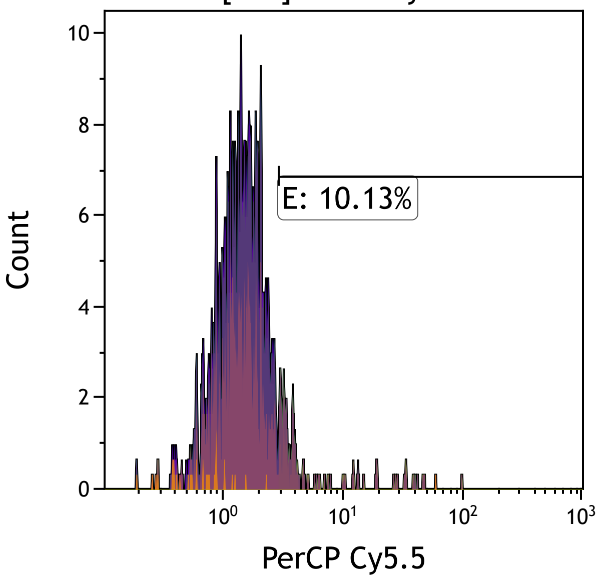

Gate	X-Med
All	1.49
E	3.83

[B++] AF 647

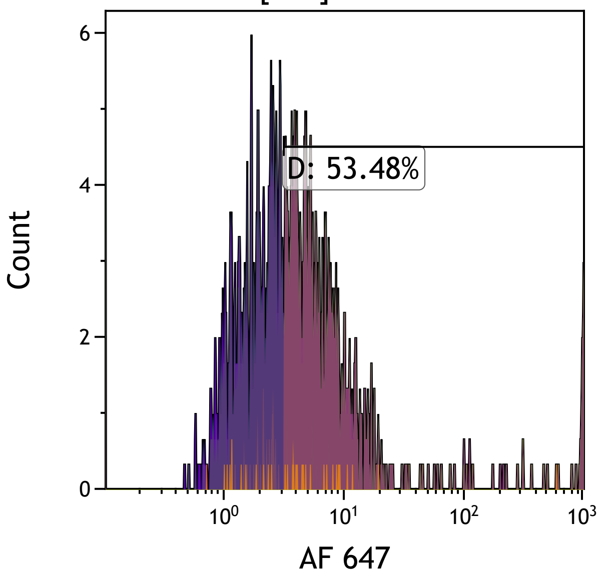

Gate	X-Med
All	3.43
D	6.03

[A++] BV421

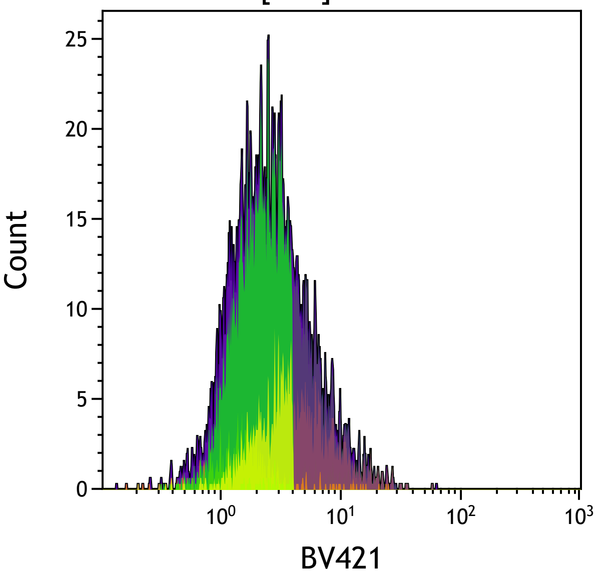

9 contra-2017-03-14-163954-21

Rat microglia M1M2 protocol - Imported

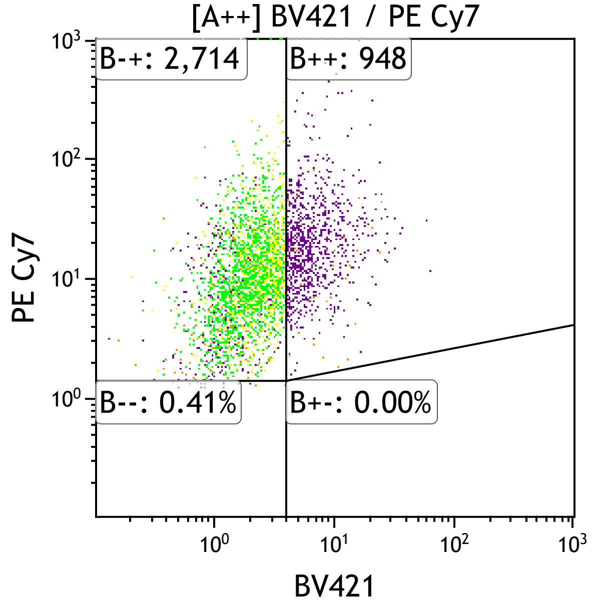

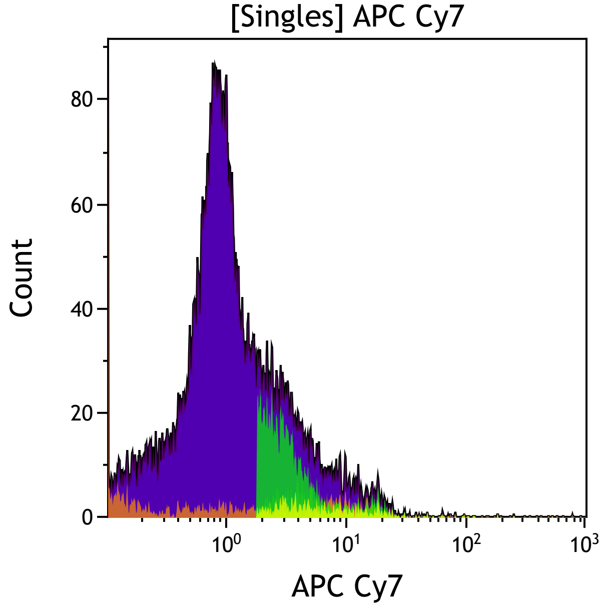

Gate	X-Med	Y-Med
All	2.49	11.20
B--	0.85	1.32
B-+	1.97	9.31
B+-	N/A	N/A
B++	6.10	18.71

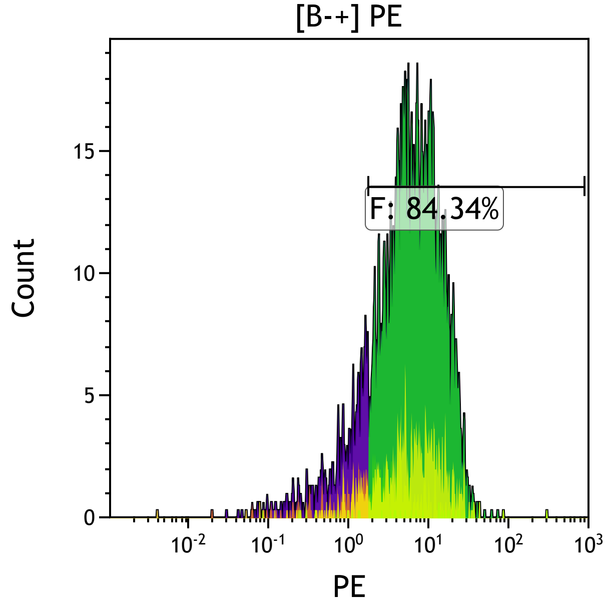

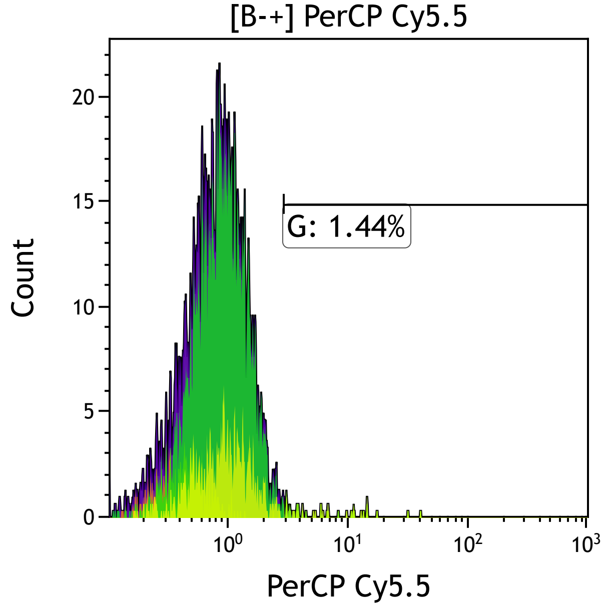

Gate	X-Med
All	5.74
F	7.05

Gate	X-Med
All	0.83
G	5.38

9 contra-2017-03-14-163954-21
Rat microglia M1M2 protocol - Imported

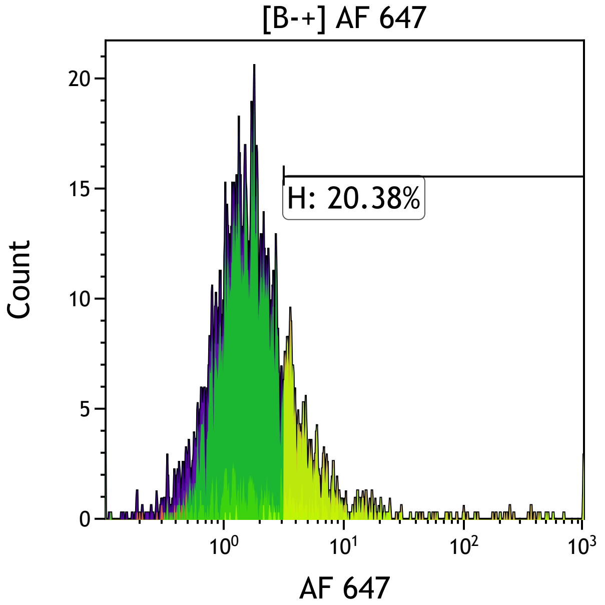

Gate	X-Med
All	1.66
H	4.80

10 contra-2017-03-14-164227-22

Rat microglia M1M2 protocol - Imported

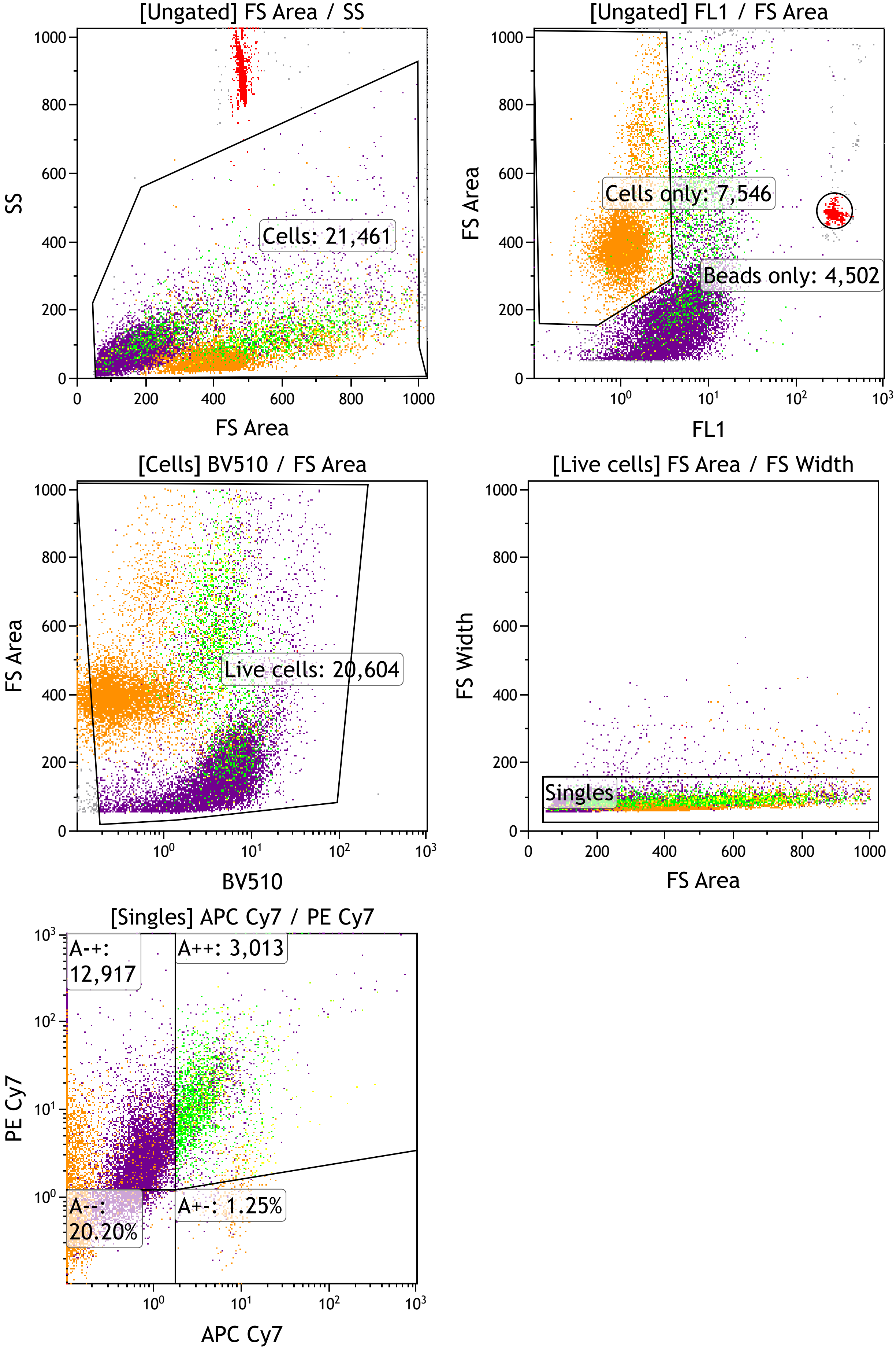

10 contra-2017-03-14-164227-22
Rat microglia M1M2 protocol - Imported

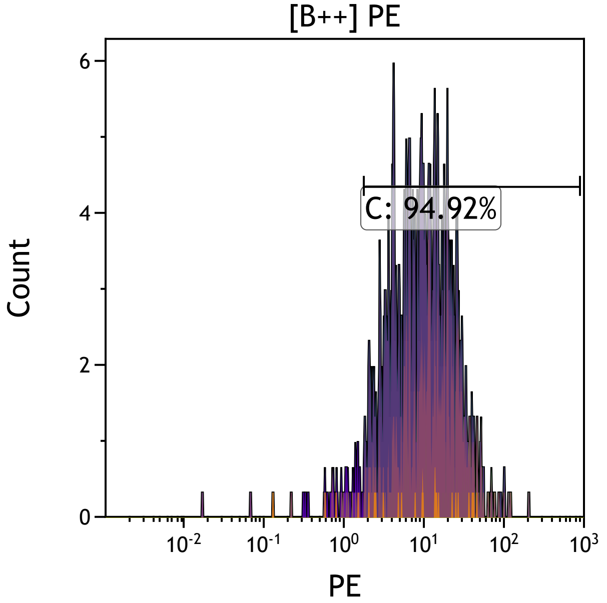

Gate	X-Med
All	9.31
C	9.81

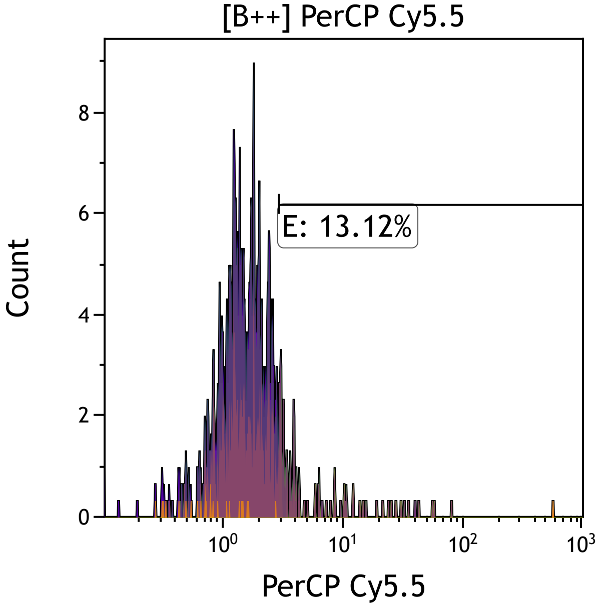

Gate	X-Med
All	1.58
E	3.93

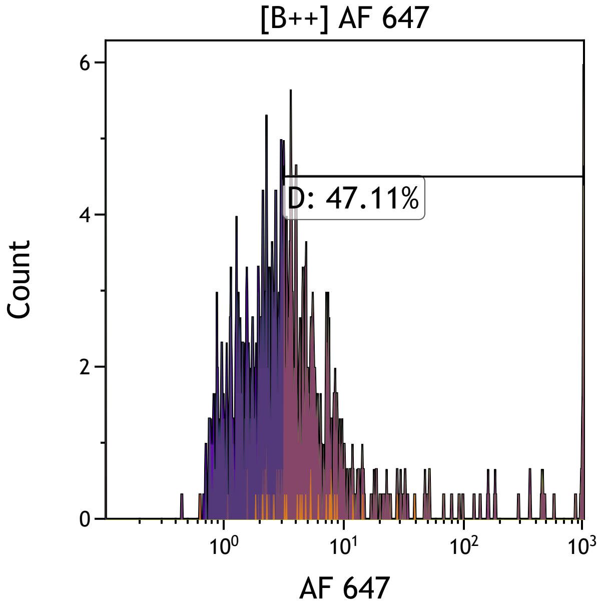

Gate	X-Med
All	3.01
D	5.53

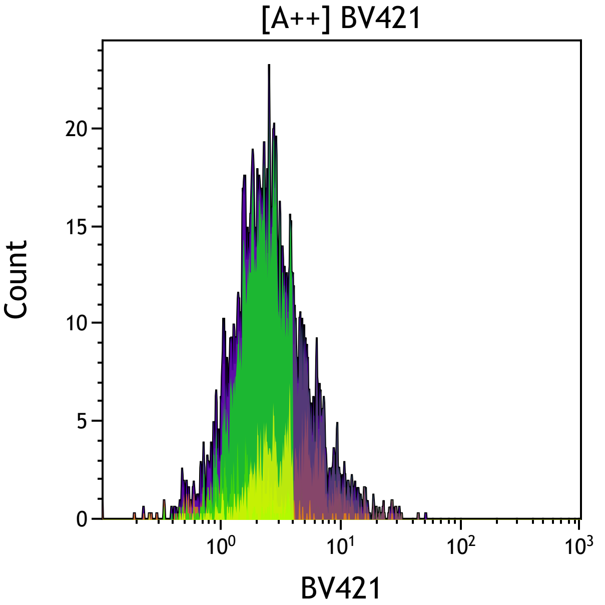

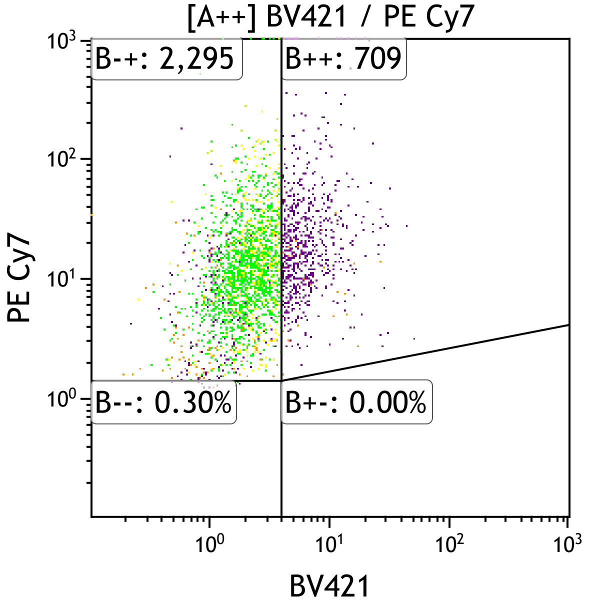

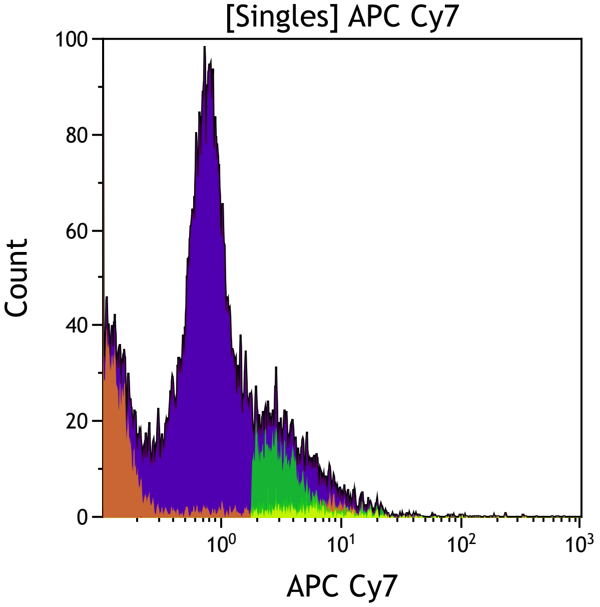

Gate	X-Med	Y-Med
All	2.46	12.09
B--	1.01	1.30
B-+	2.04	10.70
B+-	N/A	N/A
B++	5.92	18.40

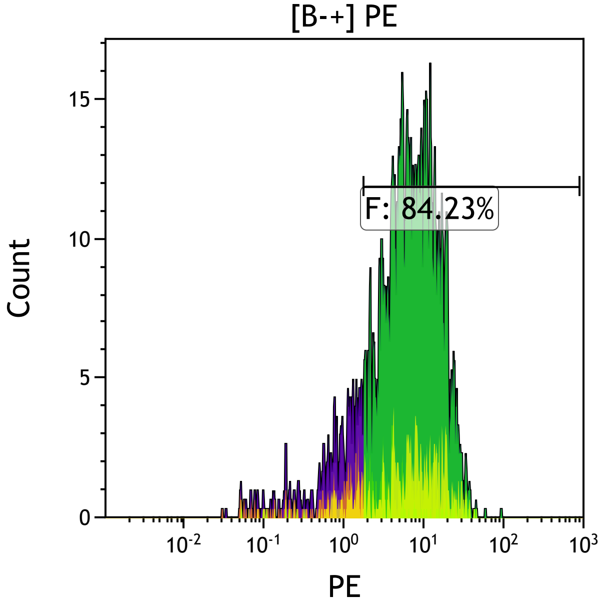

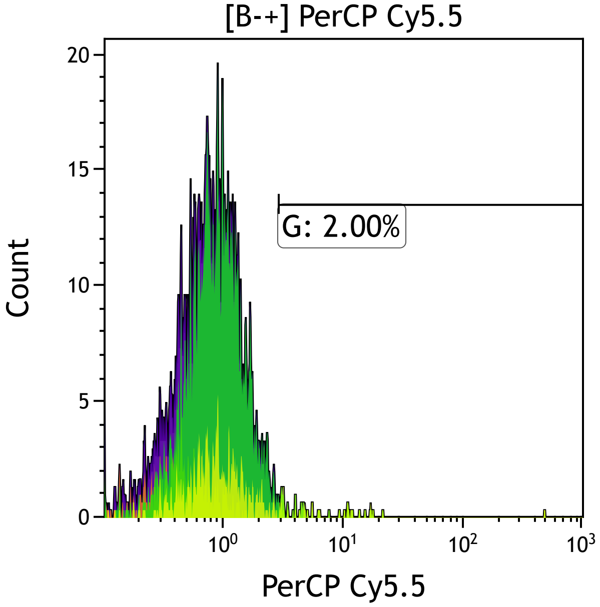

Gate	X-Med
All	6.18
F	7.49

Gate	X-Med
All	0.79
G	4.68

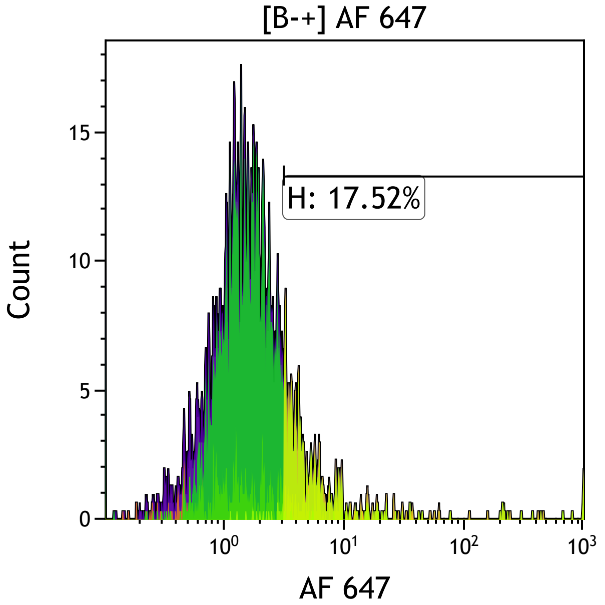

Gate	X-Med
All	1.59
H	4.70

11 contra-2017-03-14-164500-23

Rat microglia M1M2 protocol - Imported

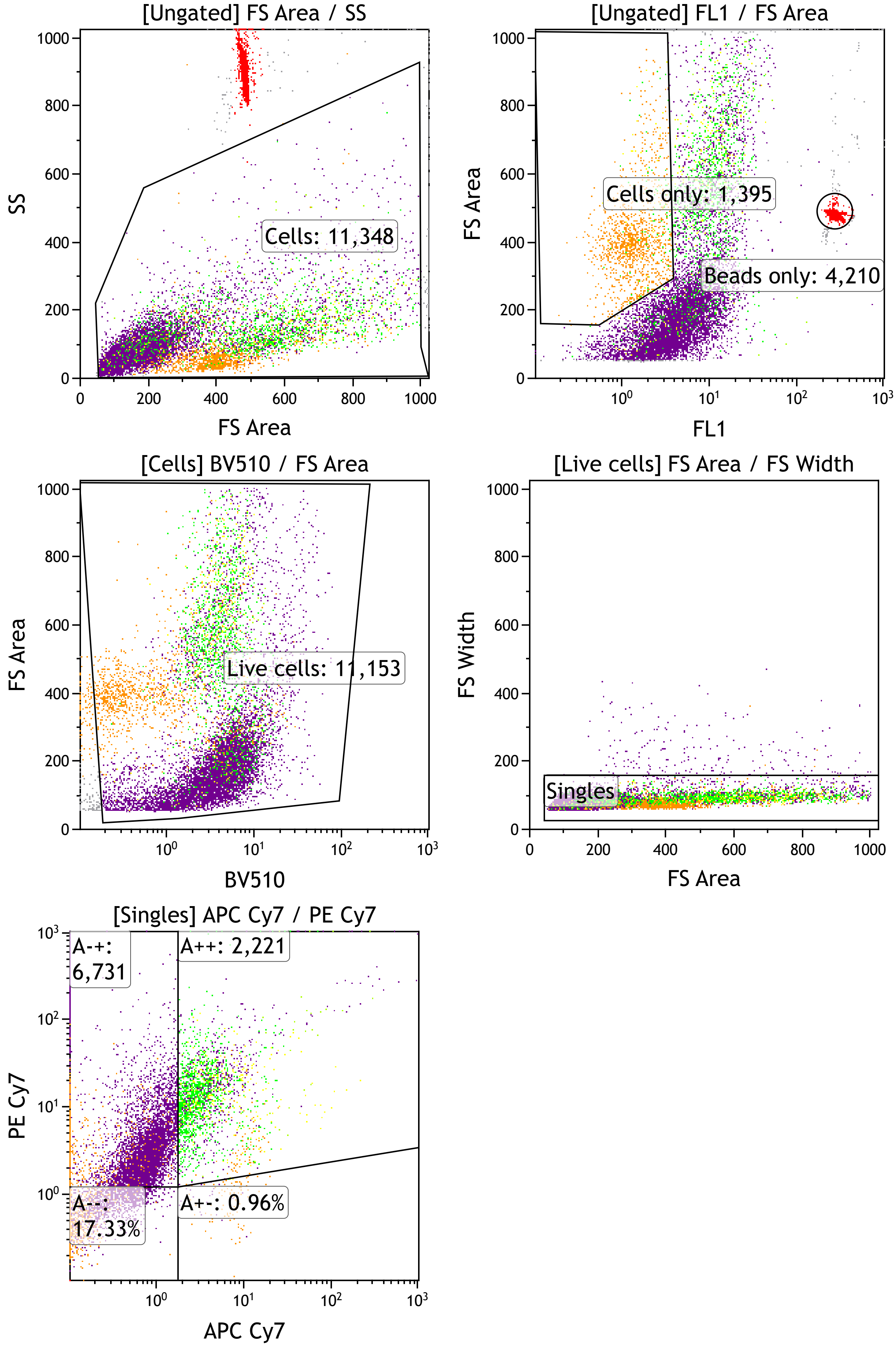

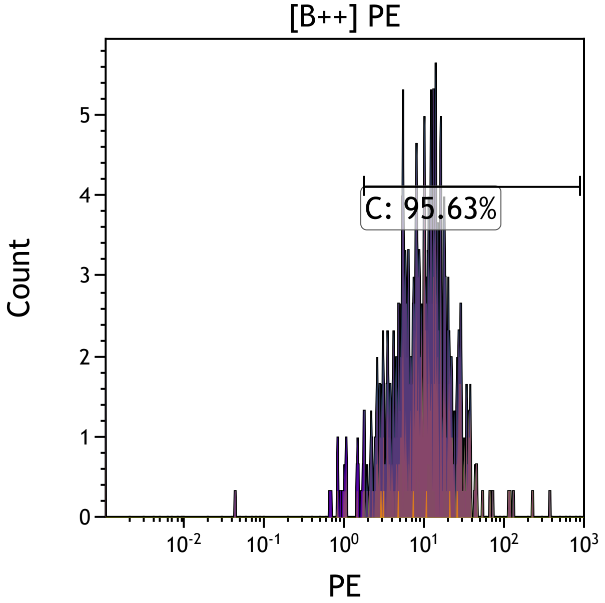

Gate	X-Med
All	9.81
C	10.19

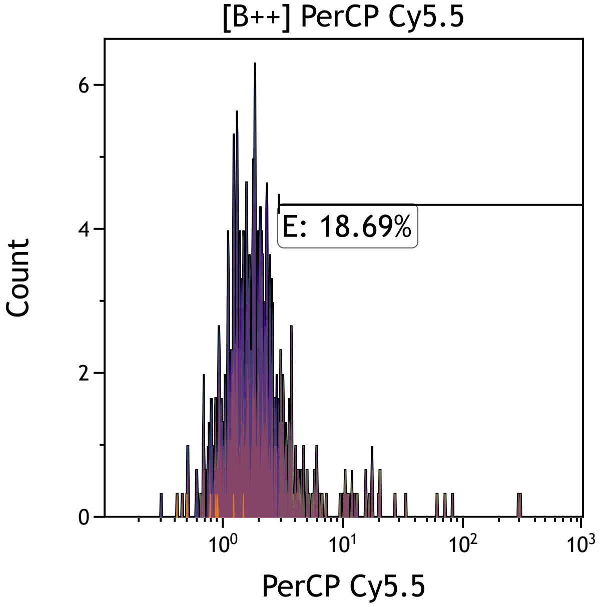

Gate	X-Med
All	1.79
E	4.20

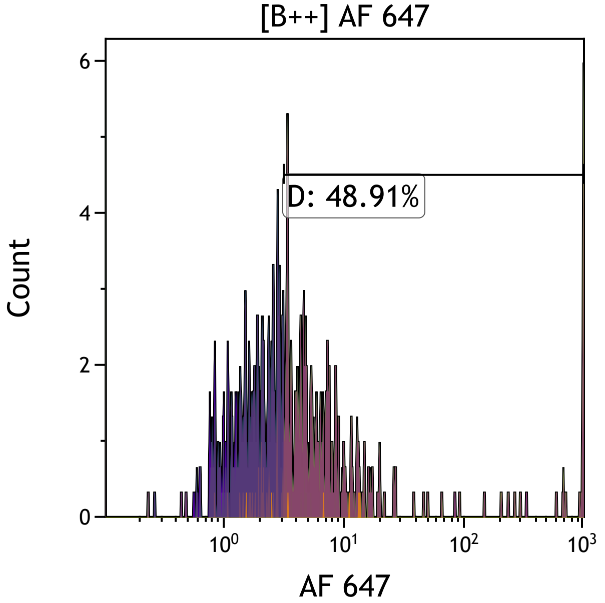

Gate	X-Med
All	3.11
D	6.40

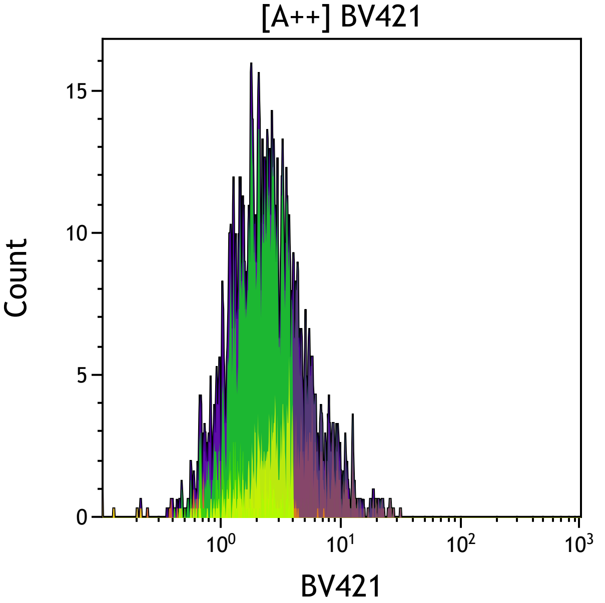

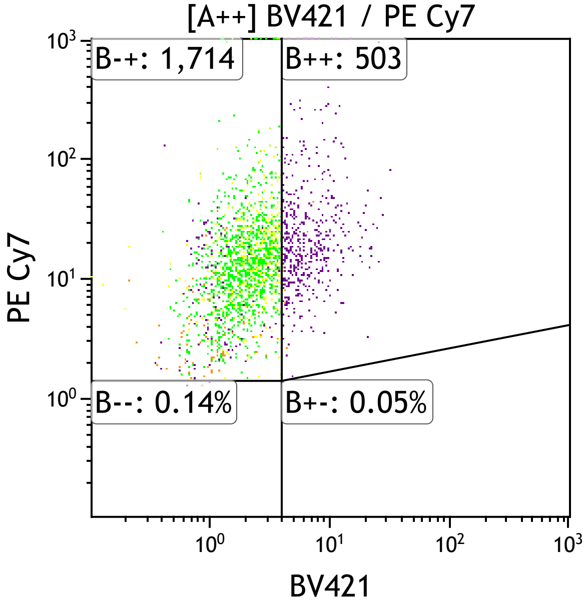

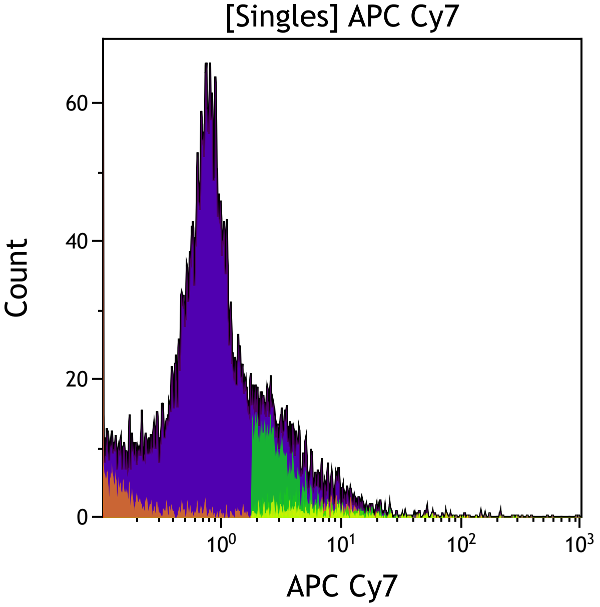

Gate	X-Med	Y-Med
All	2.36	13.54
B--	0.88	1.31
B-+	1.94	12.21
B+-	4.08	1.39
B++	5.81	18.31

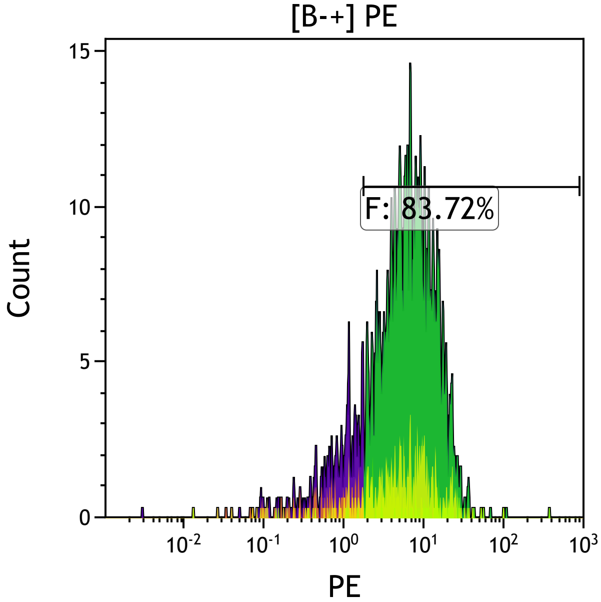

Gate	X-Med
All	5.87
F	6.93

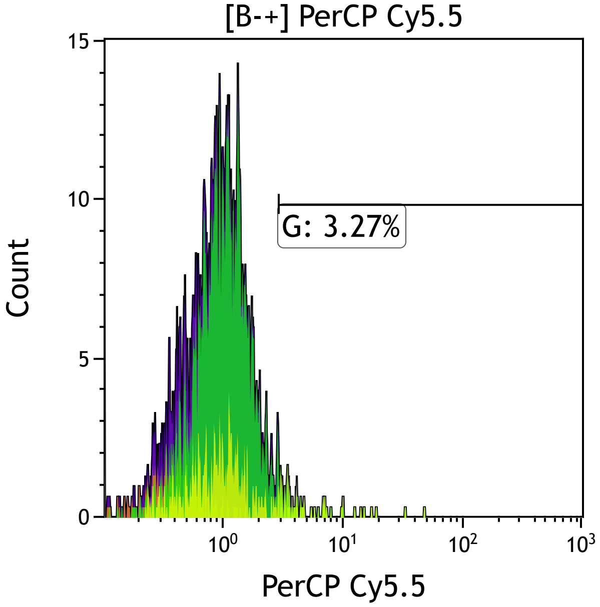

Gate	X-Med
All	0.93
G	4.09

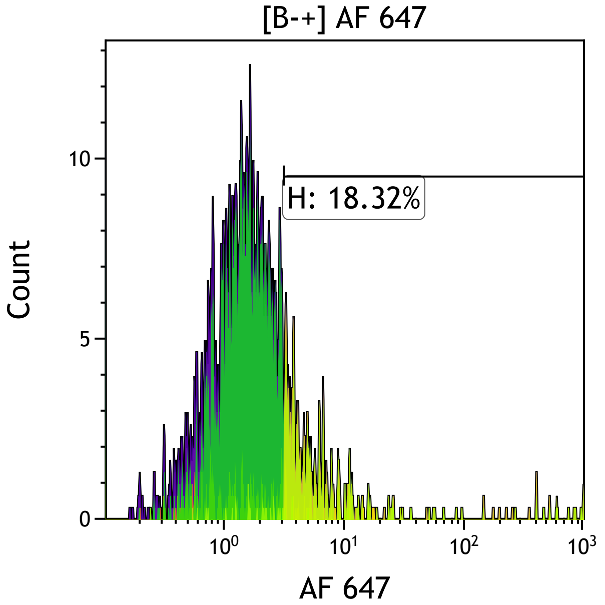

Gate	X-Med
All	1.60
H	5.07

12 contra-2017-03-14-164733-24

Rat microglia M1M2 protocol - Imported

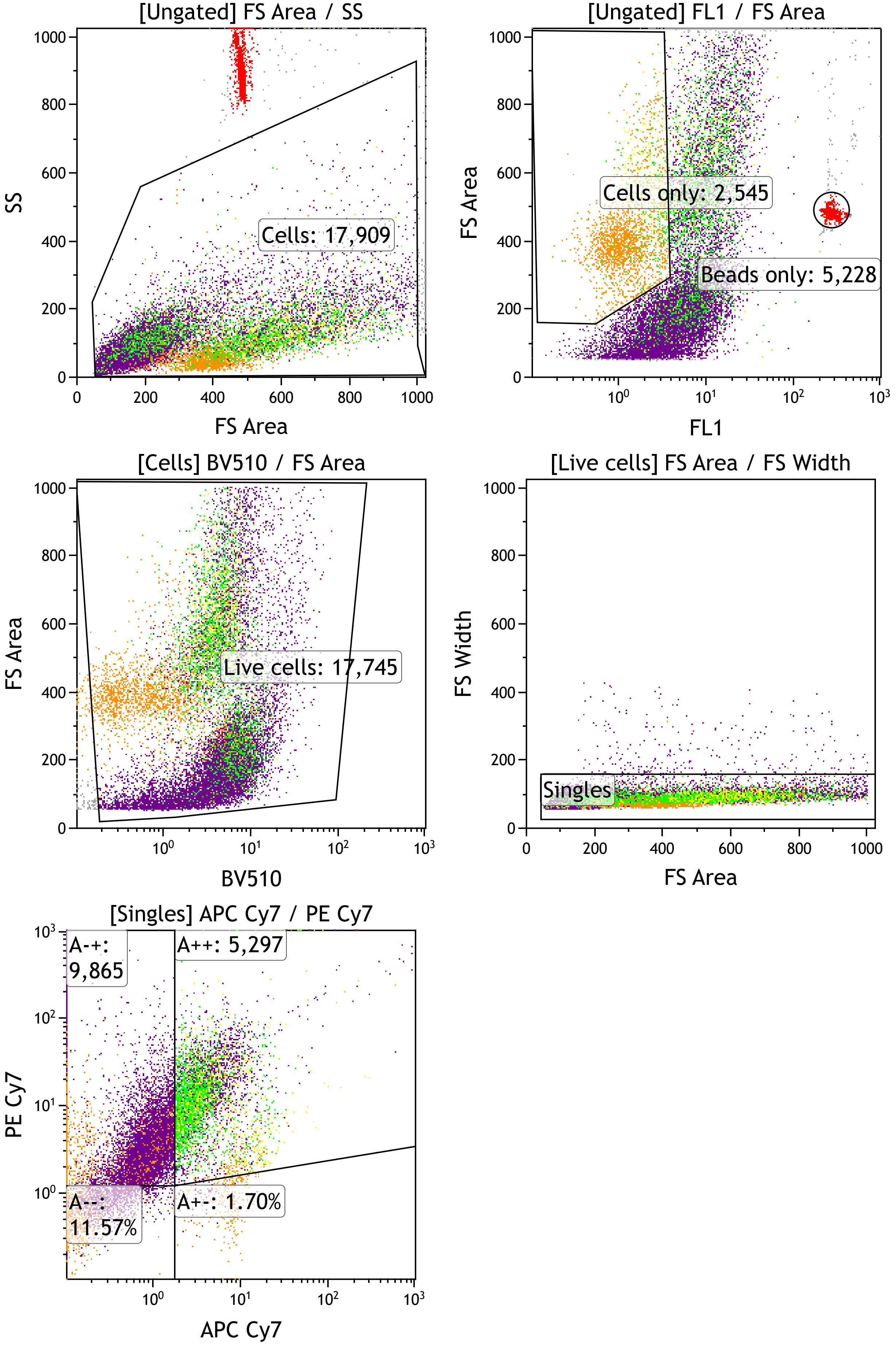

12 contra-2017-03-14-164733-24

Rat microglia M1M2 protocol - Imported

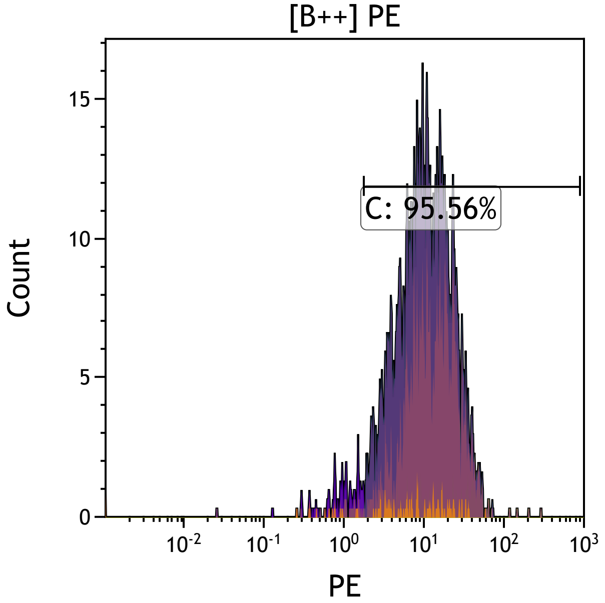

Gate	X-Med
All	10.14
C	10.66

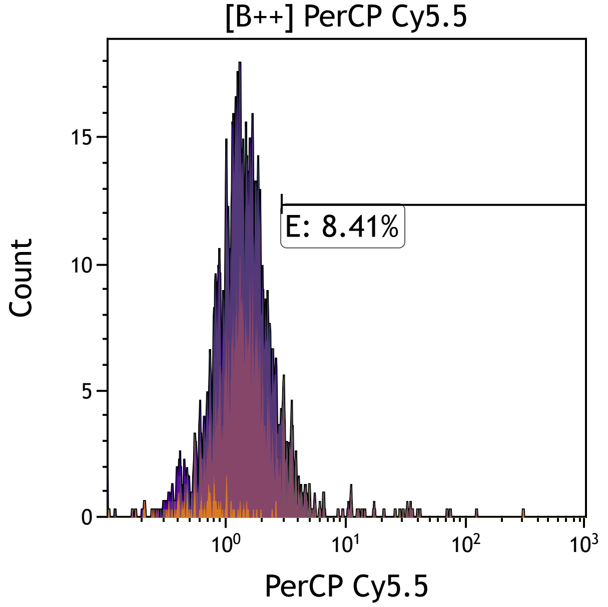

Gate	X-Med
All	1.37
E	3.64

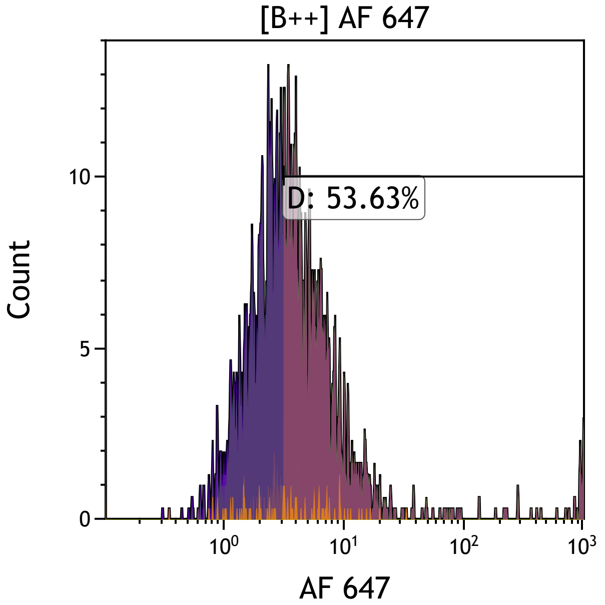

Gate	X-Med
All	3.36
D	5.39

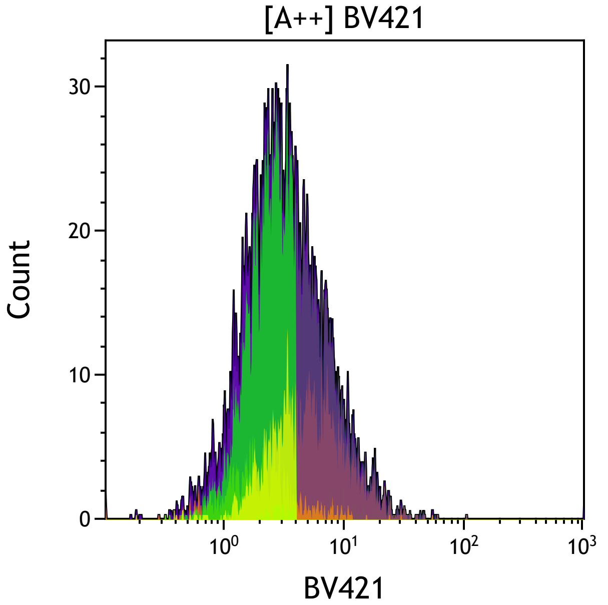

12 contra-2017-03-14-164733-24
Rat microglia M1M2 protocol - Imported

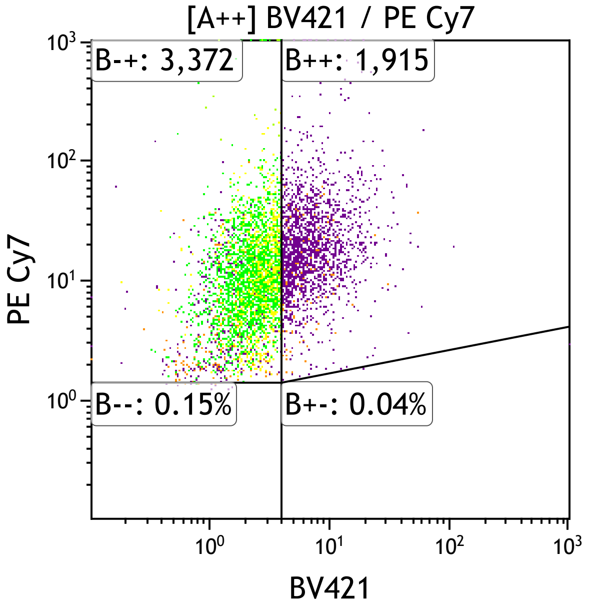

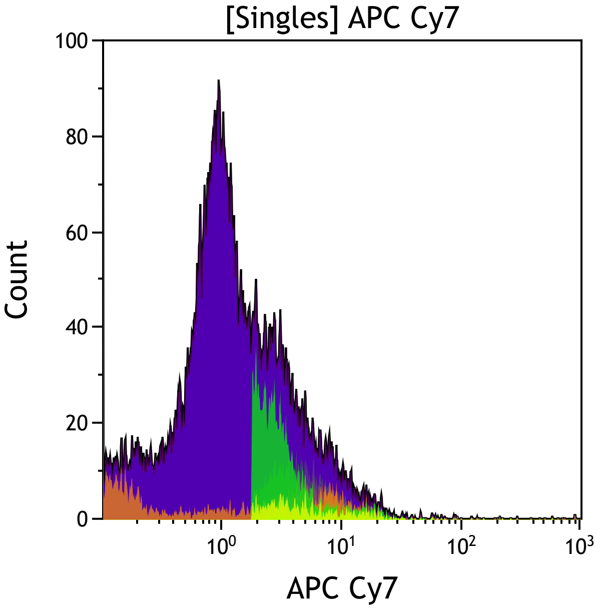

Gate	X-Med	Y-Med
All	3.07	12.31
B--	1.04	1.34
B-+	2.24	9.66
B+-	519.54	2.38
B++	6.50	18.16

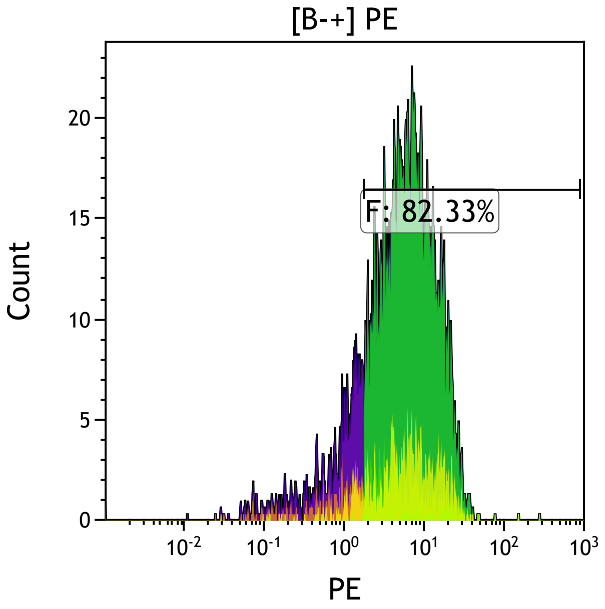

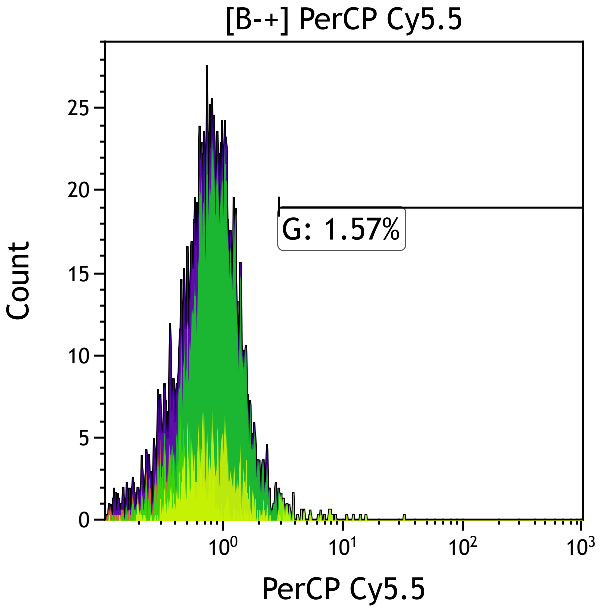

Gate	X-Med
All	5.41
F	6.74

Gate	X-Med
All	0.78
G	3.85

12 contra-2017-03-14-164733-24
Rat microglia M1M2 protocol - Imported

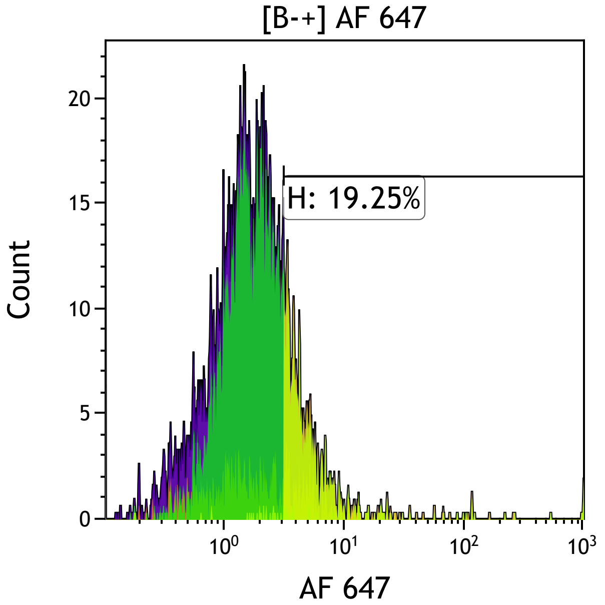

Gate	X-Med
All	1.69
H	4.41

1 ipsi-2017-03-21-112503-1
Rat microglia M1M2 protocol - Imported

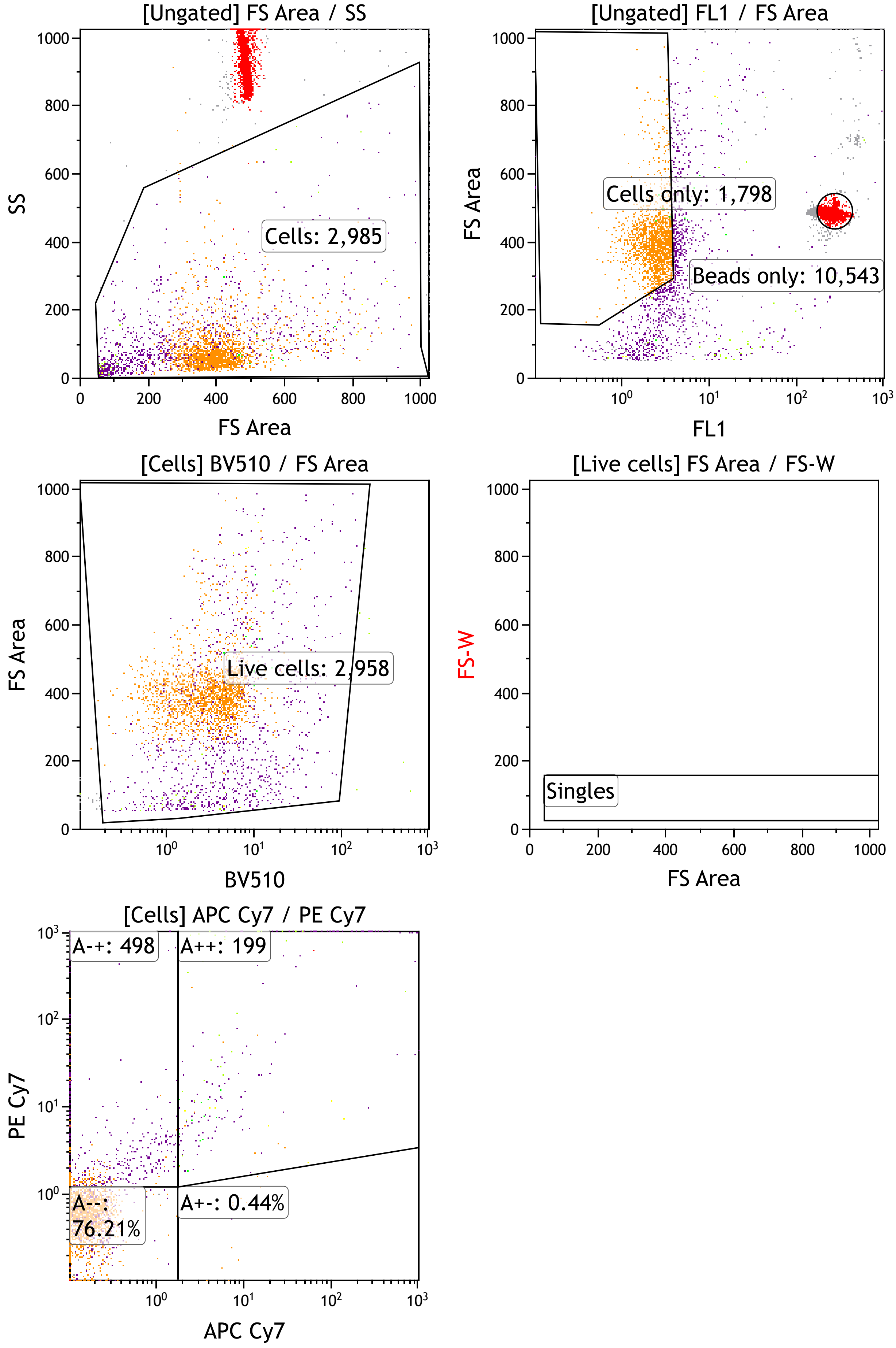

1 ipsi-2017-03-21-112503-1
Rat microglia M1M2 protocol - Imported

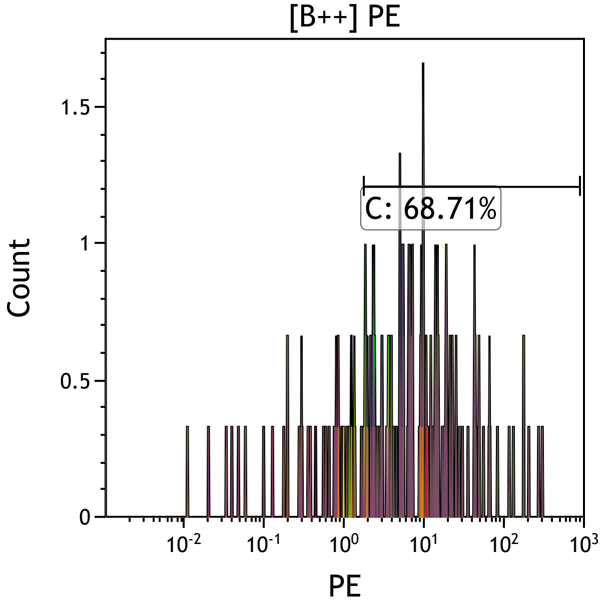

Gate	X-Med
All	4.98
C	9.42

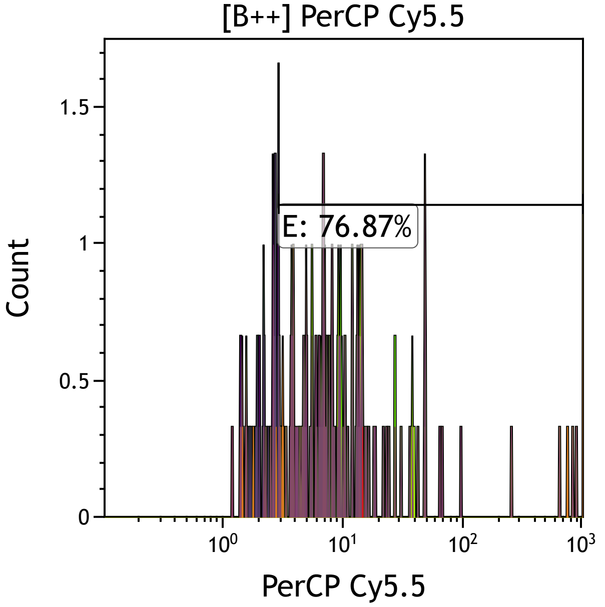

Gate	X-Med
All	6.94
E	9.55

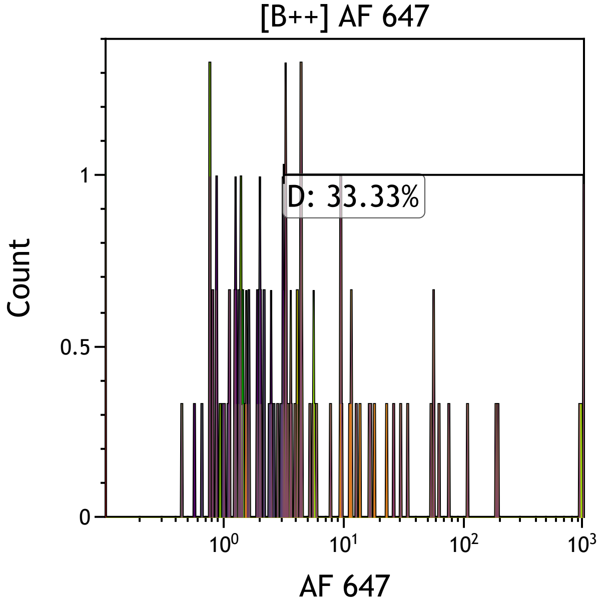

Gate	X-Med
All	1.39
D	9.66

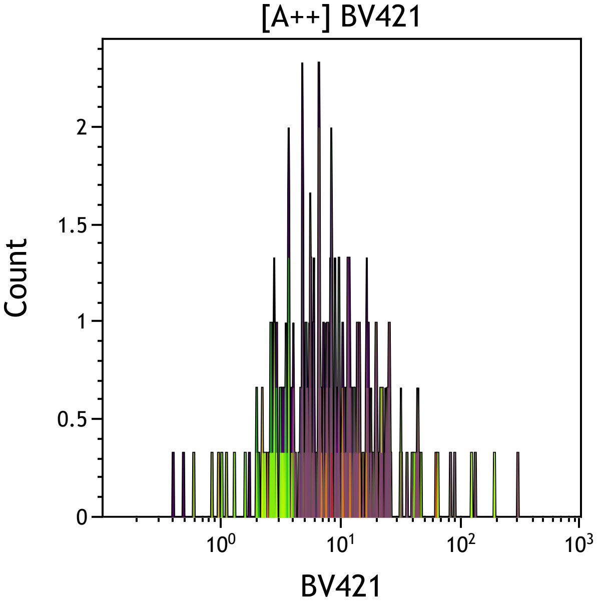

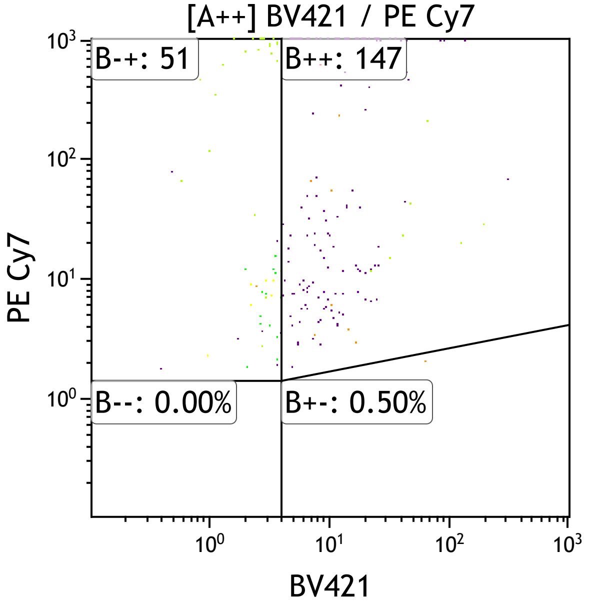

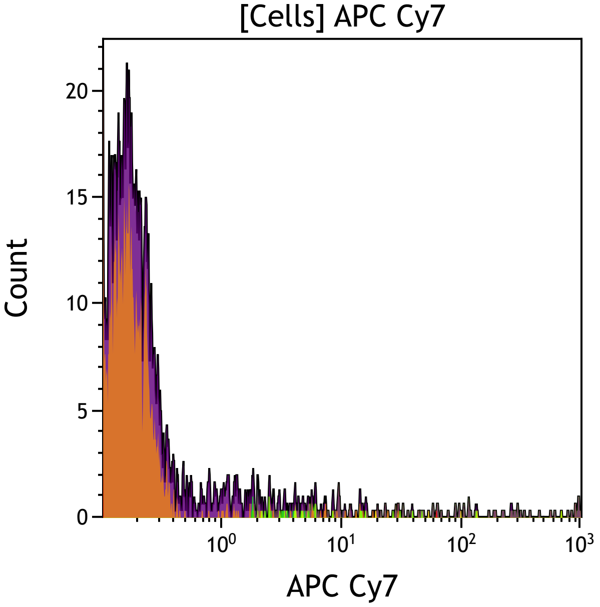

Gate	X-Med	Y-Med
All	7.64	23.96
B--	N/A	N/A
B-+	2.79	11.99
B+-	62.49	2.10
B++	10.45	31.95

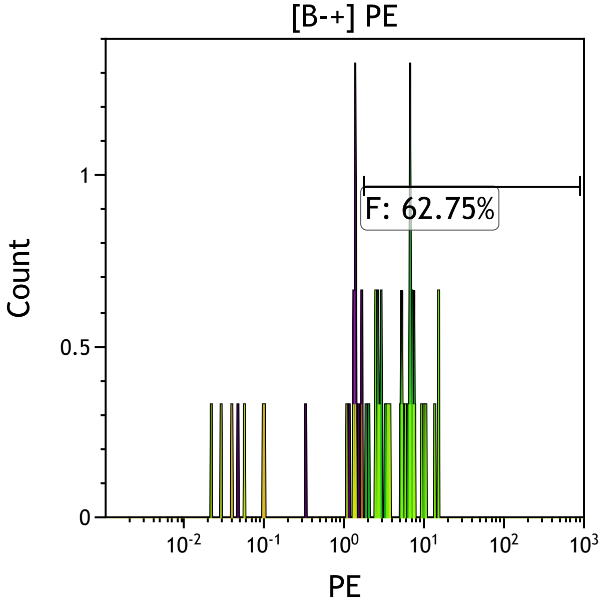

Gate	X-Med
All	2.80
F	5.84

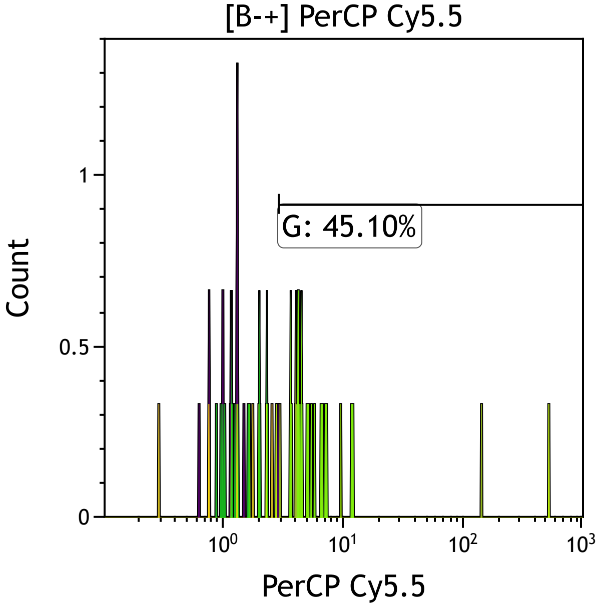

Gate	X-Med
All	2.57
G	5.19

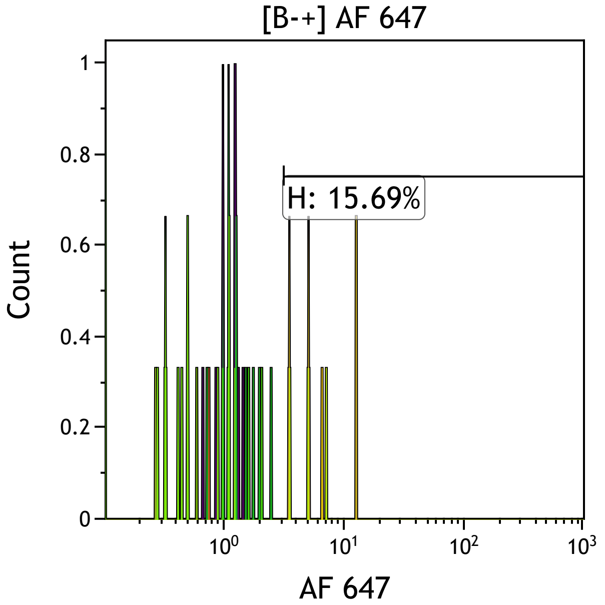

Gate	X-Med
All	0.98
H	6.59

2 ipsi-2017-03-21-112921-2
Rat microglia M1M2 protocol - Imported

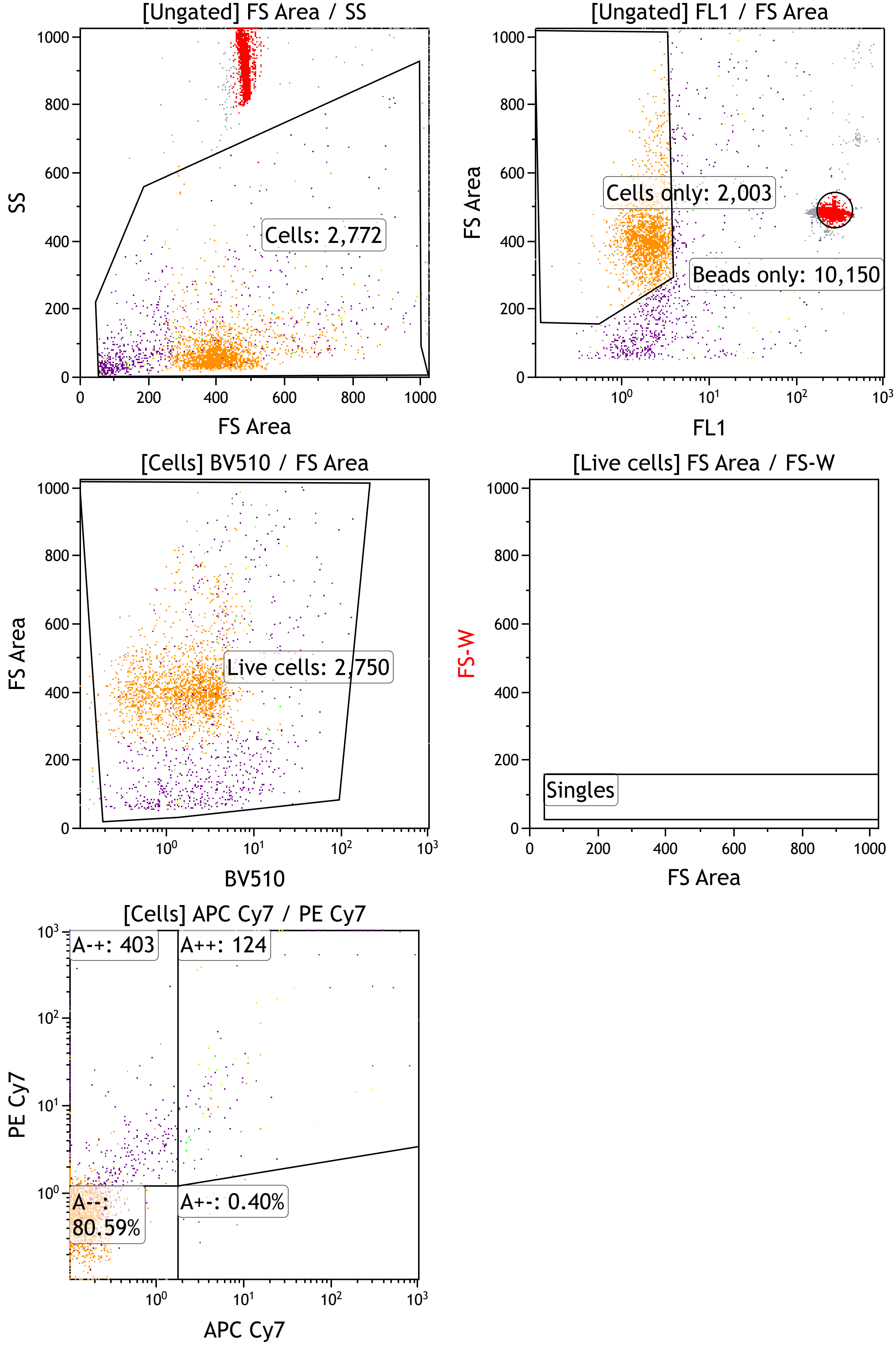

2 ipsi-2017-03-21-112921-2
Rat microglia M1M2 protocol - Imported

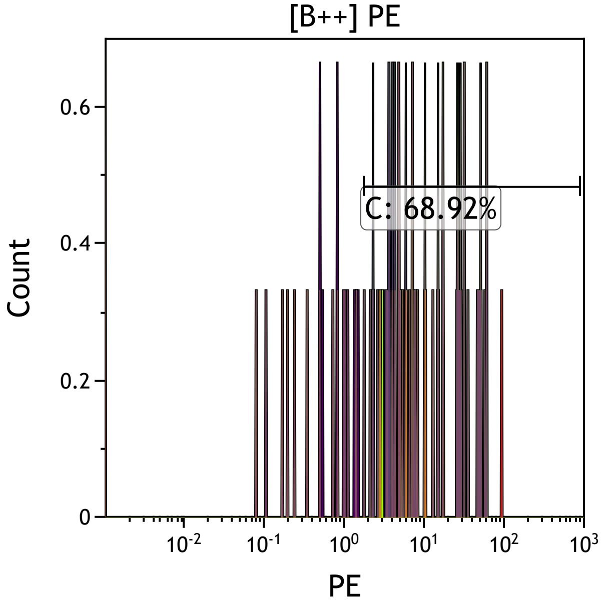

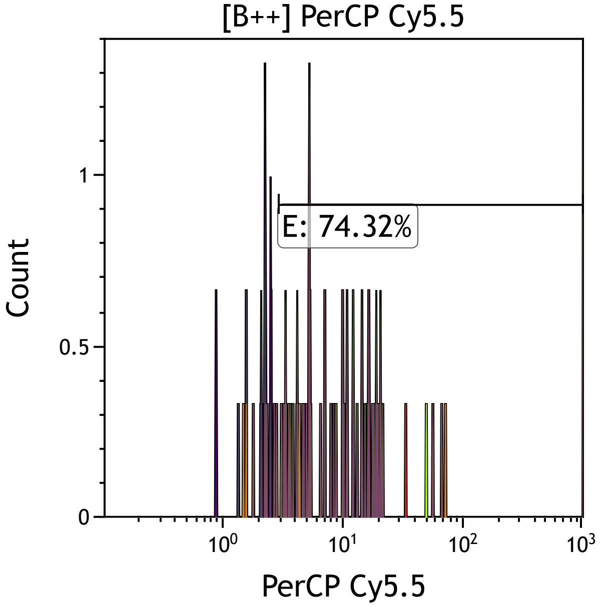

Gate	X-Med
All	4.28
C	7.59

Gate	X-Med
All	6.53
E	10.86

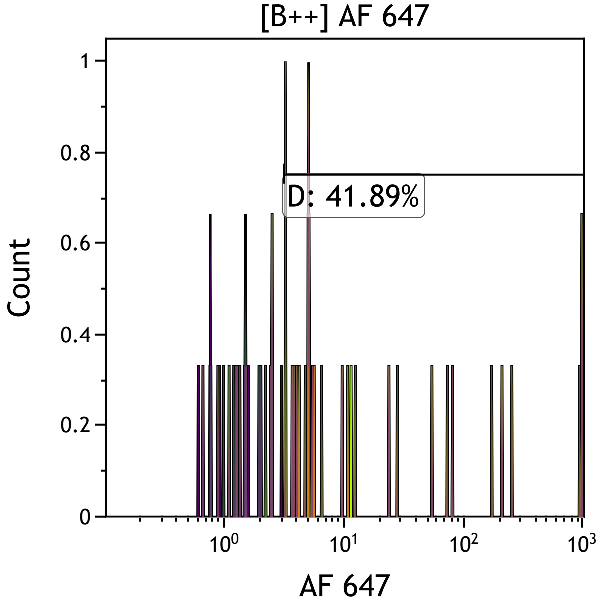

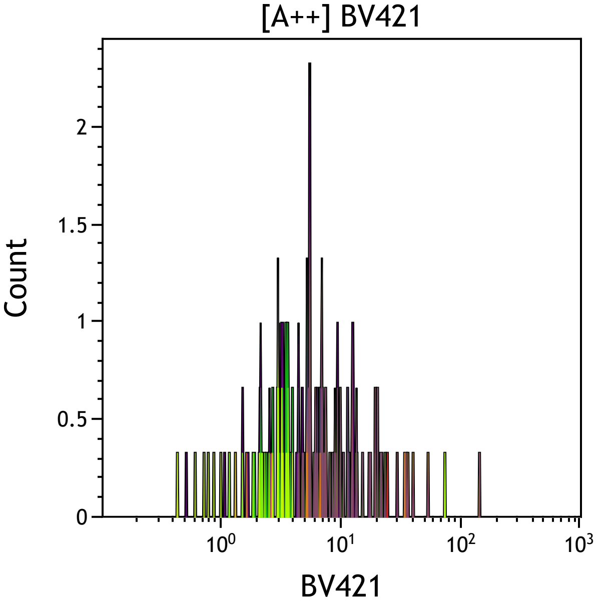

Gate	X-Med
All	2.03
D	9.75

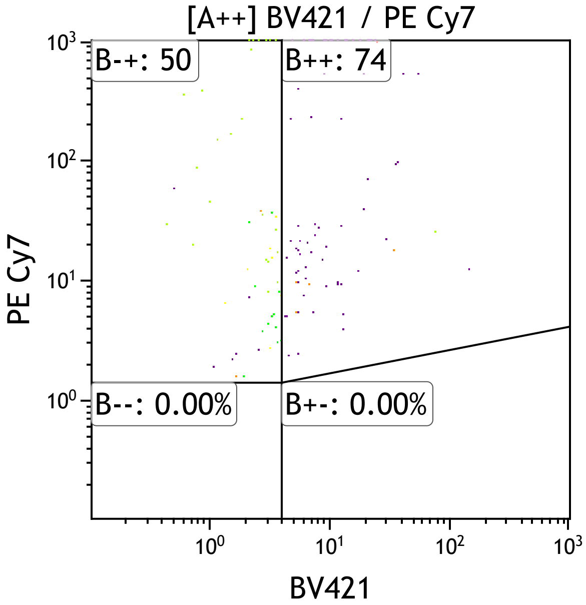

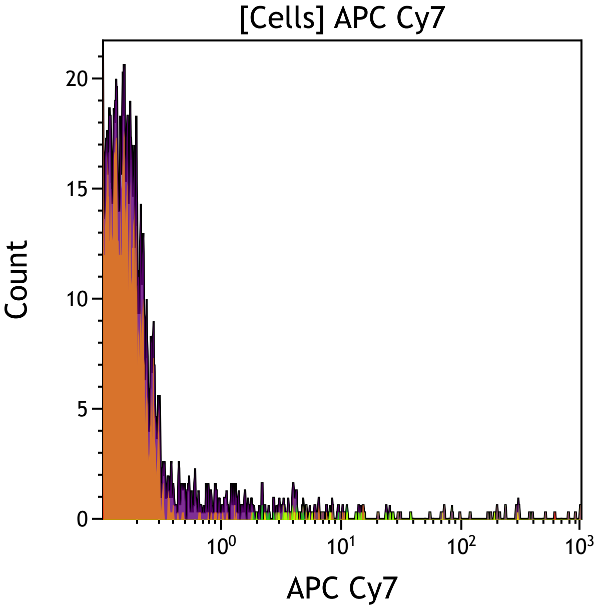

Gate	X-Med	Y-Med
All	5.43	22.39
B--	N/A	N/A
B-+	2.63	16.95
B+-	N/A	N/A
B++	8.87	27.29

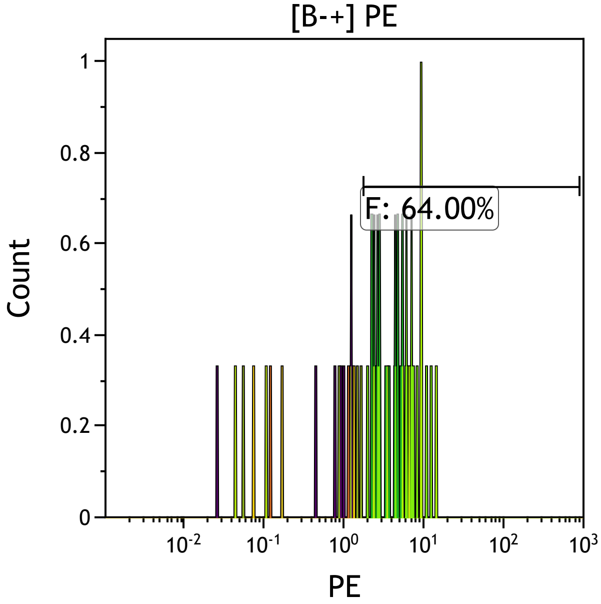

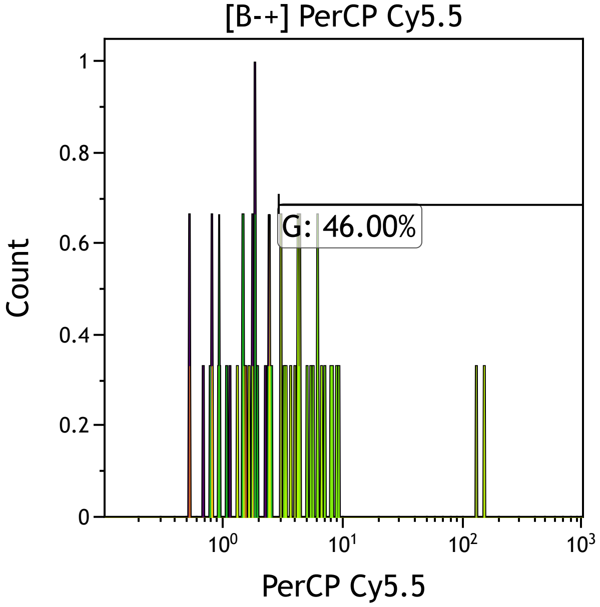

Gate	X-Med
All	2.78
F	5.17

Gate	X-Med
All	2.47
G	5.43

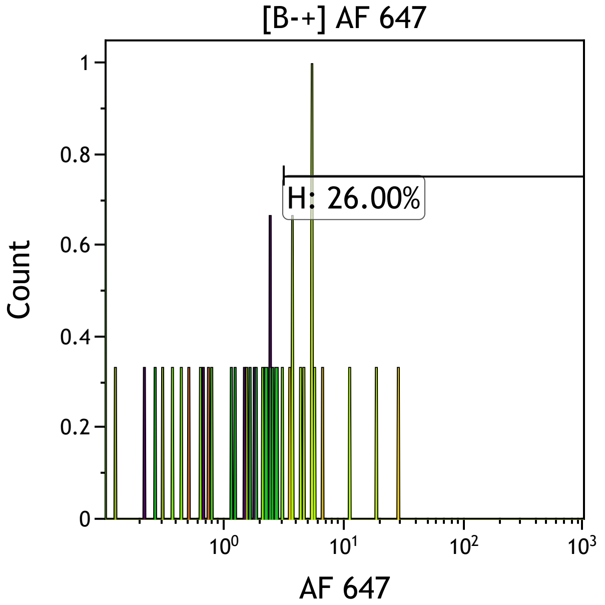

Gate	X-Med
All	1.65
H	5.44

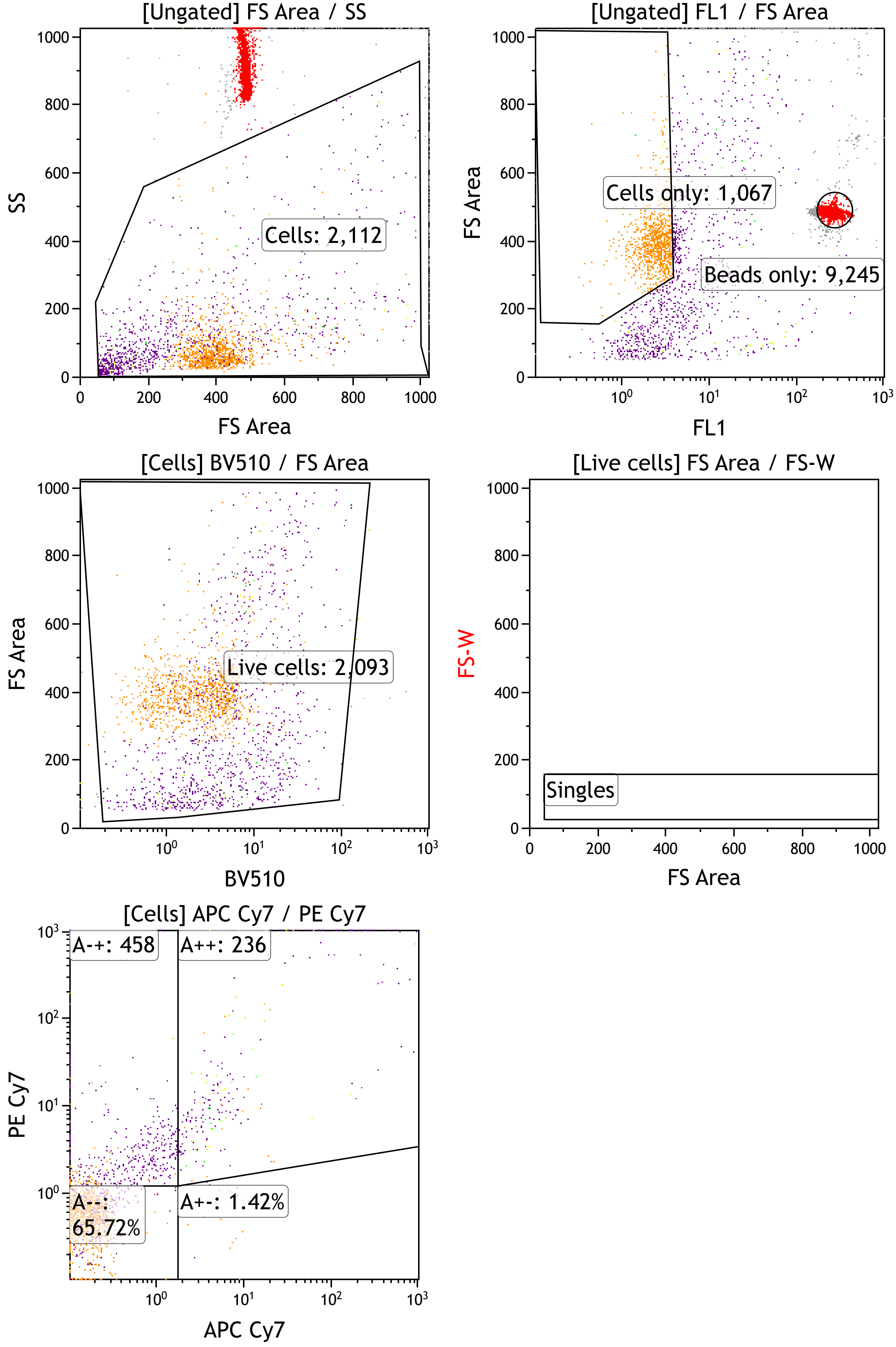
[Ungated] FS Area / SS
Cells: 2,112
SS
FS Area
[Ungated] FL1 / FS Area
Cells only: 1,067
Beads only: 9,245
FS Area
FL1
[Cells] BV510 / FS Area
Live cells: 2,093
FS Area
BV510
[Live cells] FS Area / FS-W
Singles
FS-W
FS Area
[Cells] APC Cy7 / PE Cy7
A-+: 458
A++: 236
A--: 65.72%
A+-: 1.42%
PE Cy7
APC Cy7

3 ipsi-2017-03-21-113155-3
Rat microglia M1M2 protocol - Imported

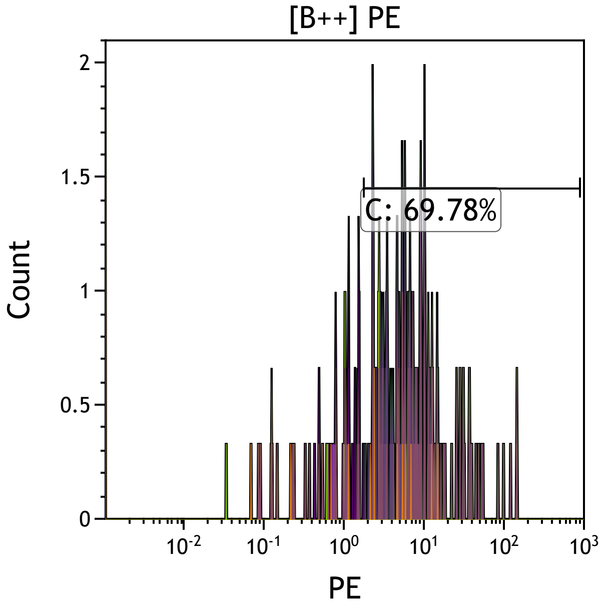

Gate	X-Med
All	4.17
C	6.82

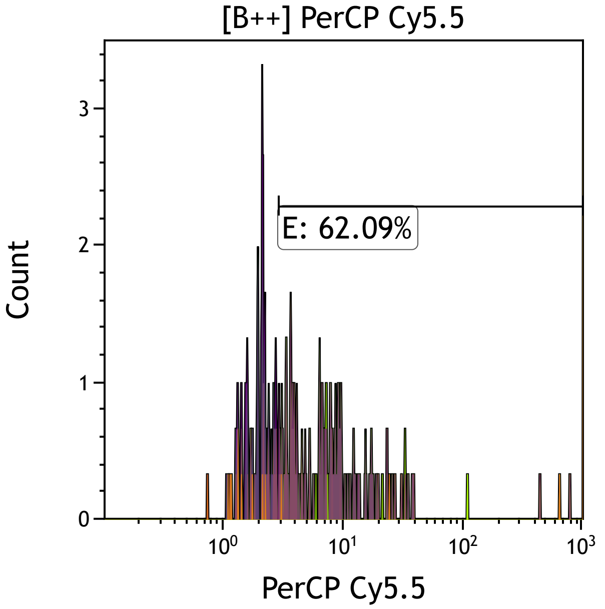

Gate	X-Med
All	3.91
E	8.14

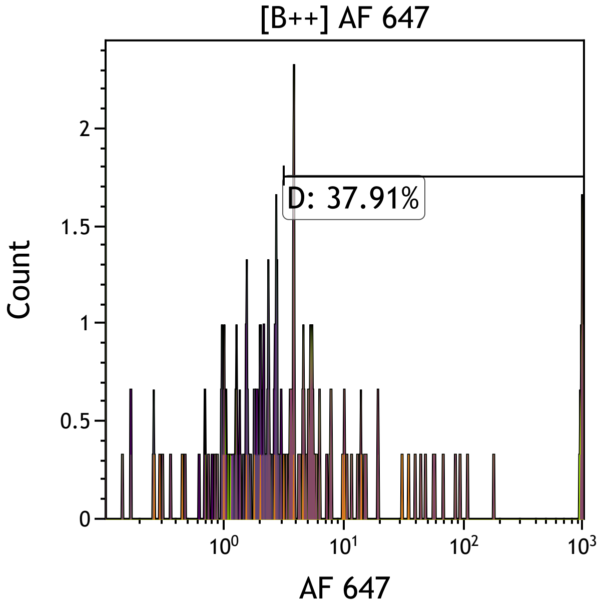

Gate	X-Med
All	2.15
D	7.24

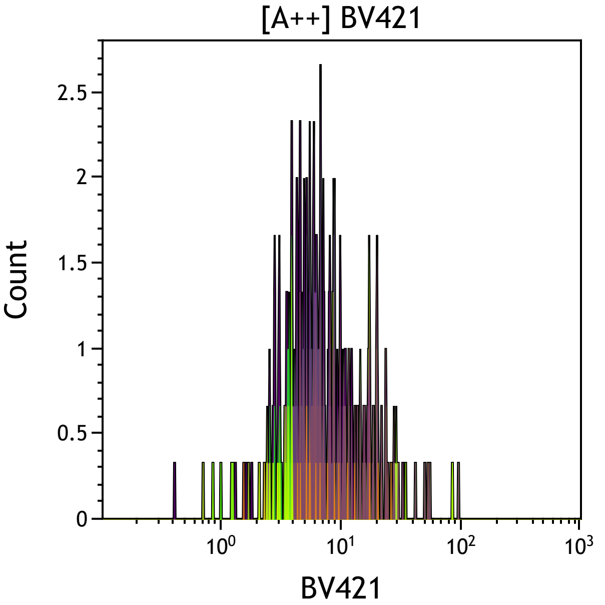

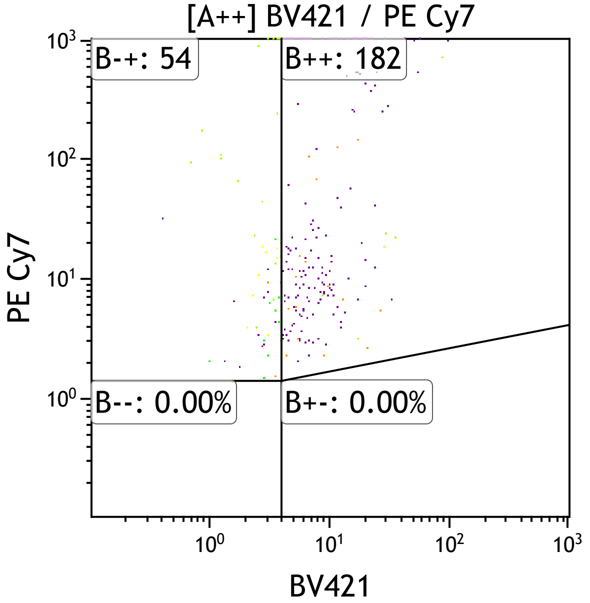

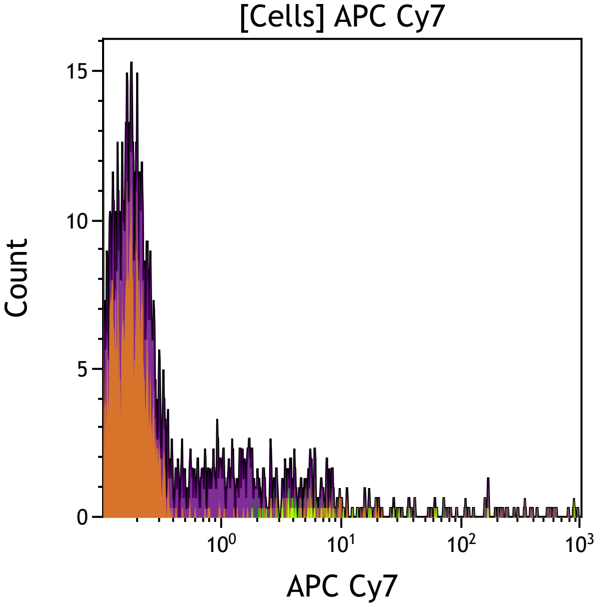

Gate	X-Med	Y-Med
All	6.27	12.59
B--	N/A	N/A
B-+	3.04	9.44
B+-	N/A	N/A
B++	7.92	13.97

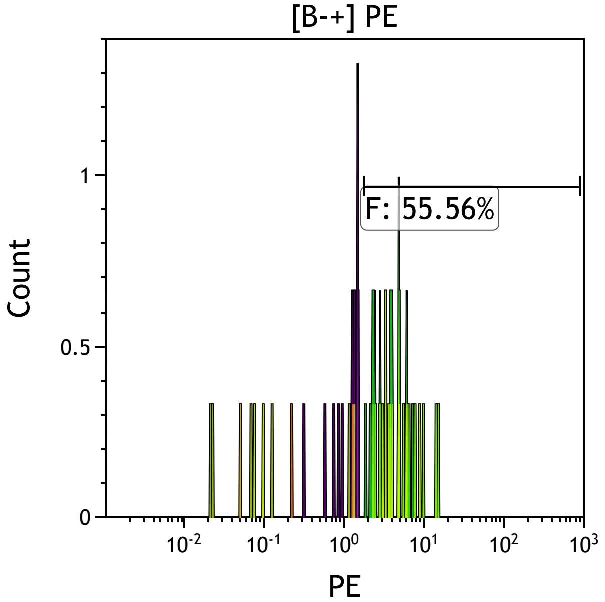

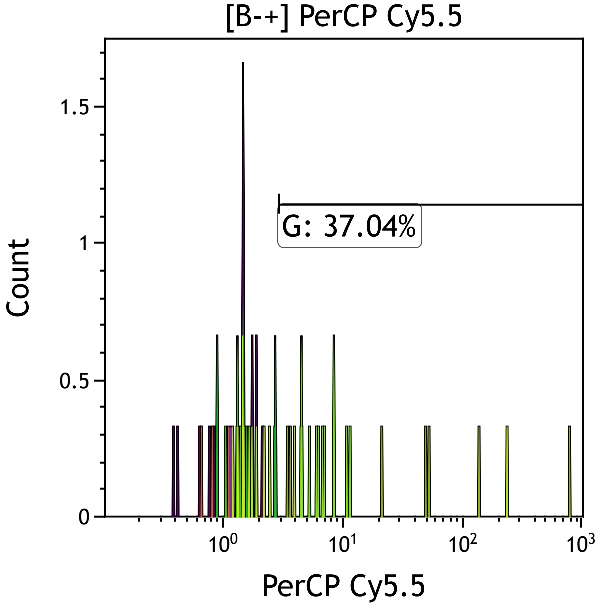

Gate	X-Med
All	2.30
F	3.95

Gate	X-Med
All	1.89
G	8.40

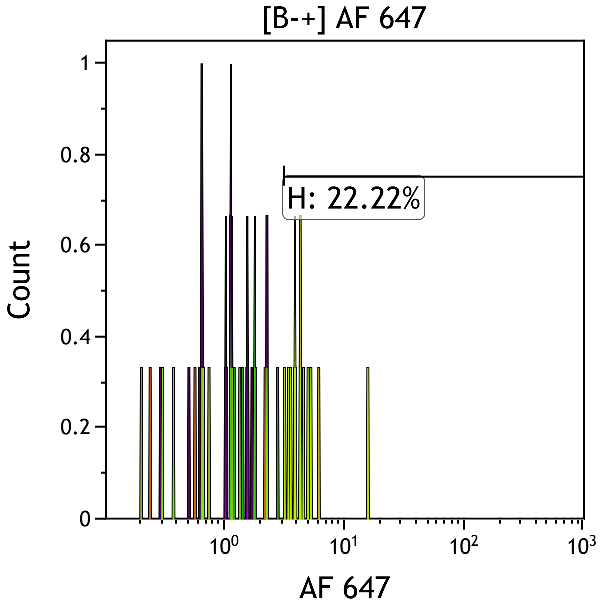

Gate	X-Med
All	1.14
H	4.36

4 ipsi-2017-03-21-113429-4
Rat microglia M1M2 protocol - Imported

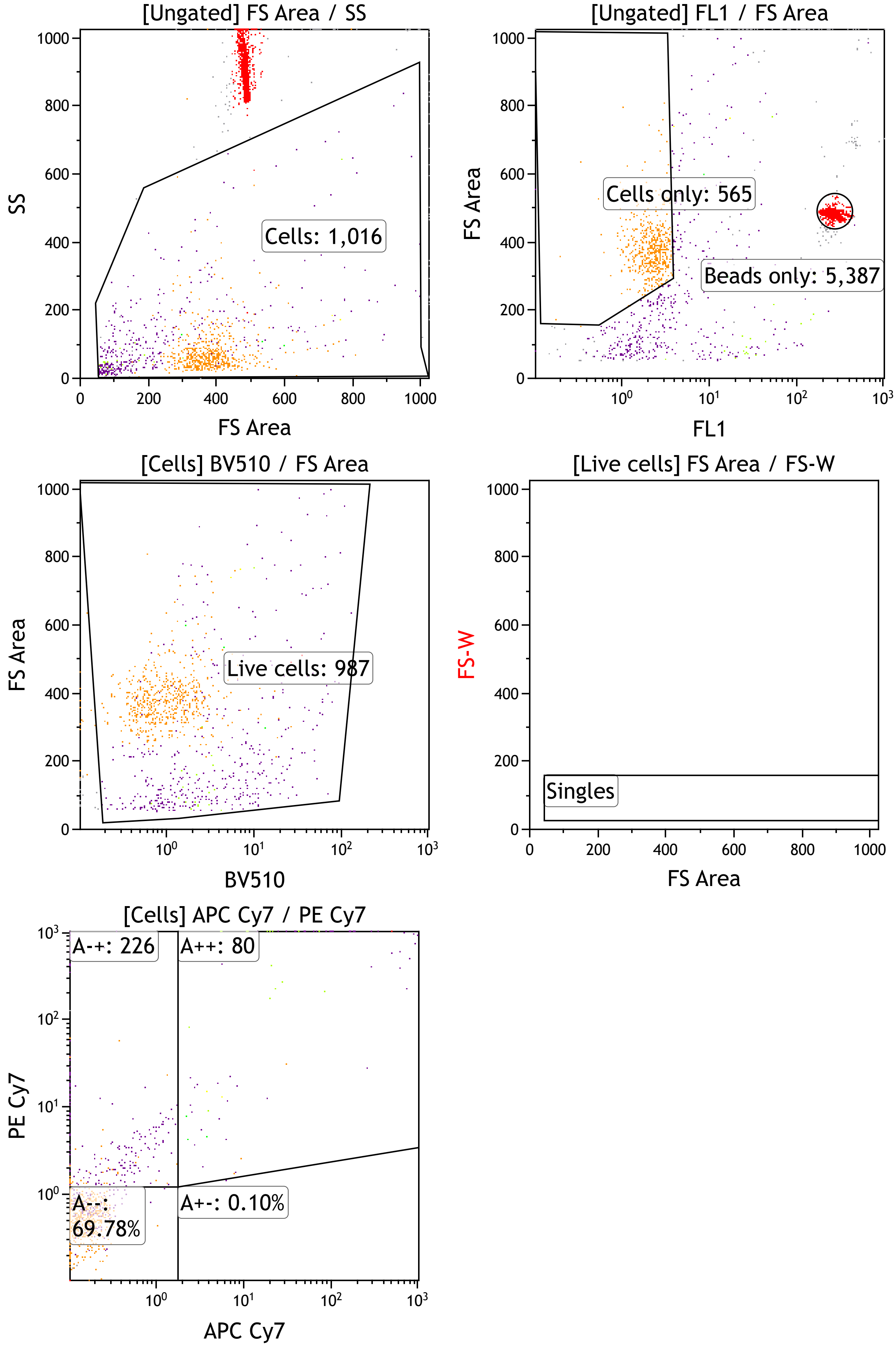

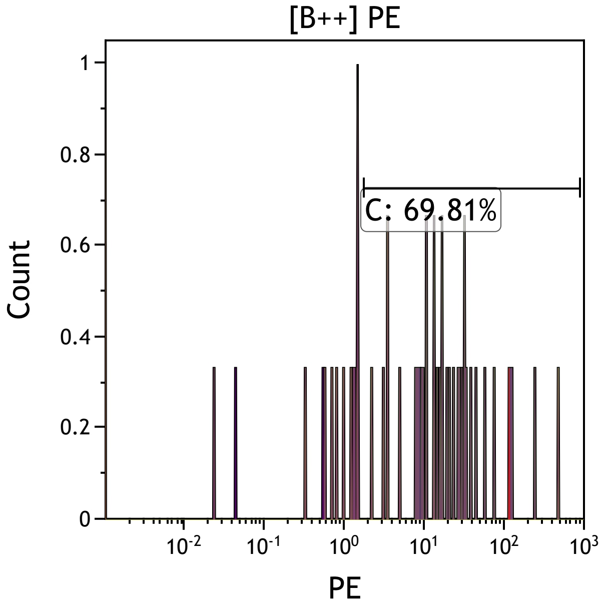

Gate	X-Med
All	9.81
C	17.06

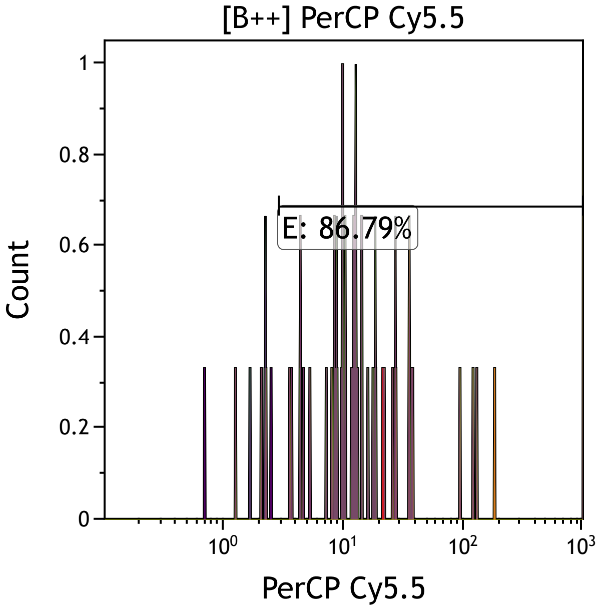

Gate	X-Med
All	12.20
E	12.82

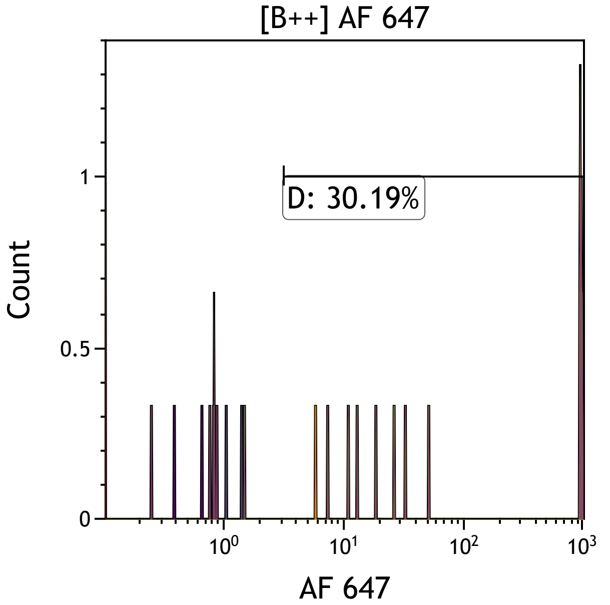

Gate	X-Med
All	0.10
D	935.92

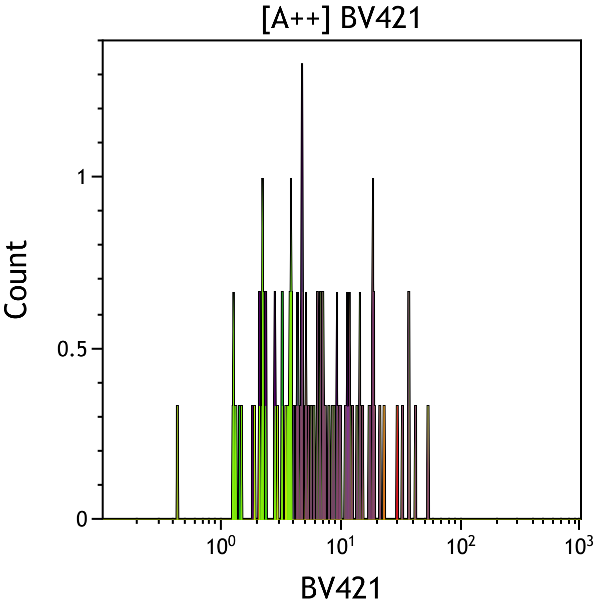

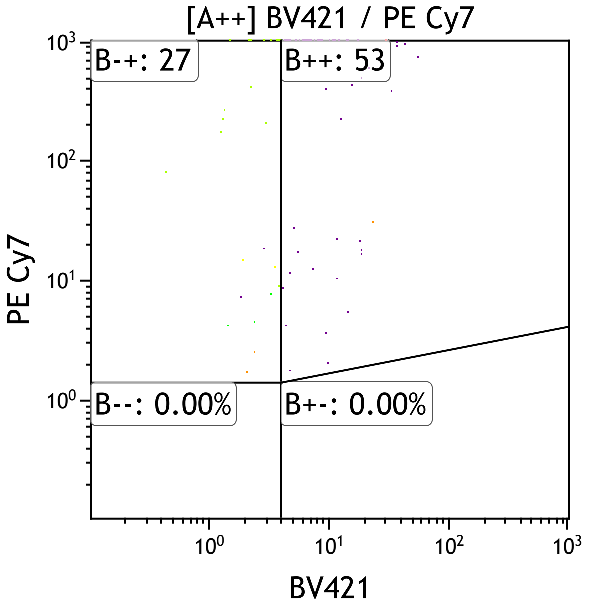

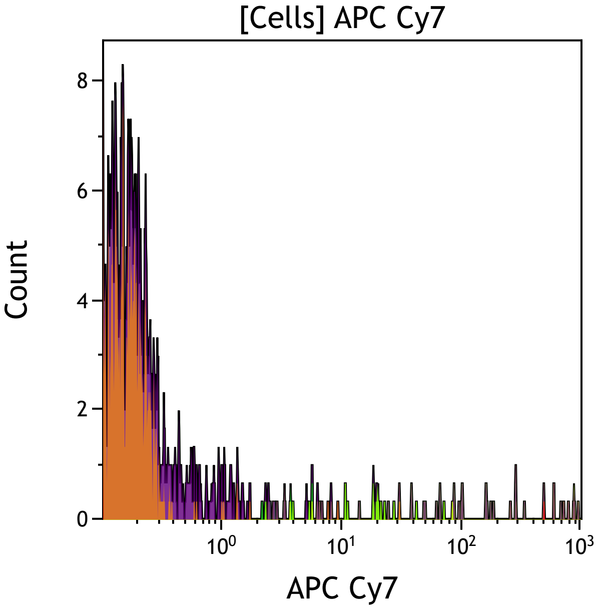

Gate	X-Med	Y-Med
All	5.97	911.00
B--	N/A	N/A
B-+	2.37	224.96
B+-	N/A	N/A
B++	9.40	983.38

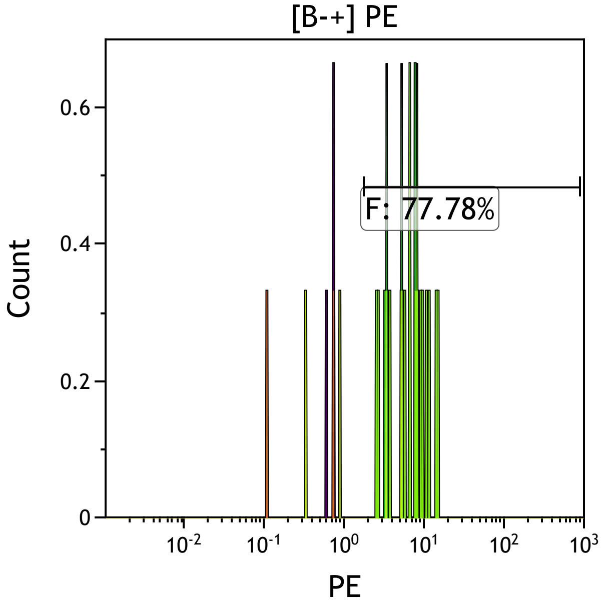

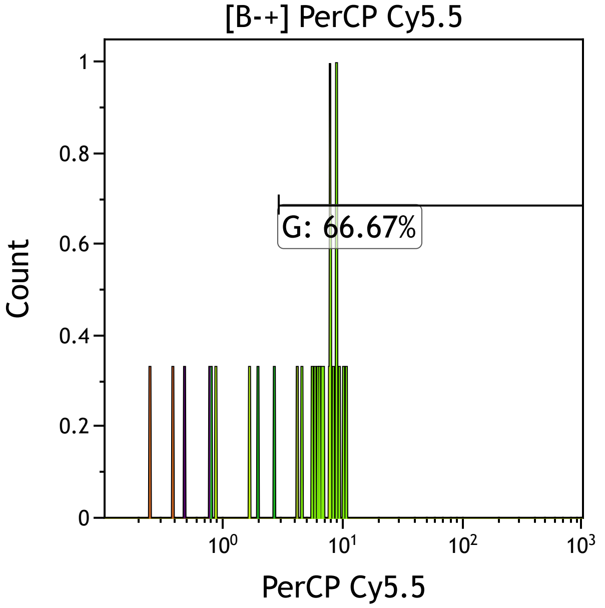

Gate	X-Med
All	5.35
F	6.72

Gate	X-Med
All	6.22
G	7.89

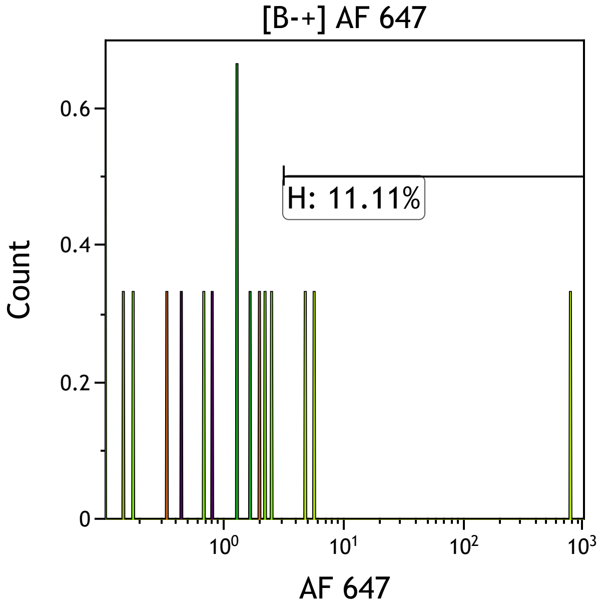

Gate	X-Med
All	0.17
H	5.68

5 ipsi-2017-03-21-113701-5

Rat microglia M1M2 protocol - Imported

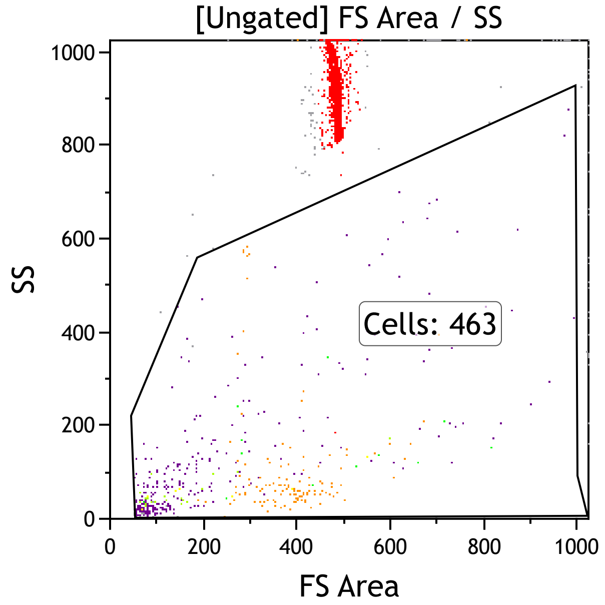

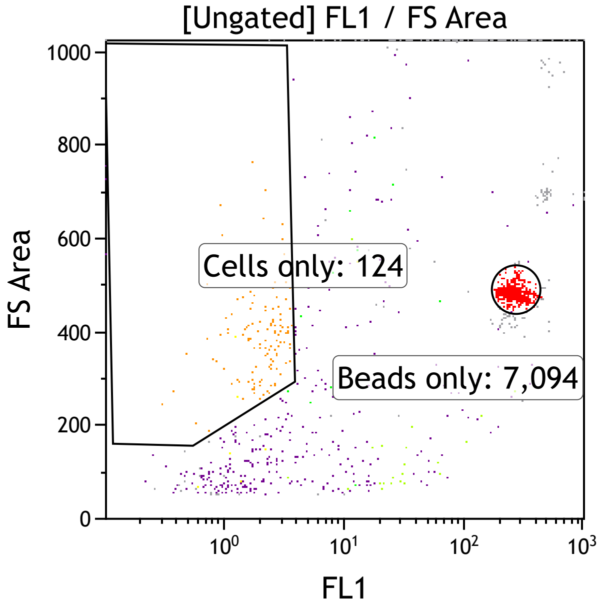

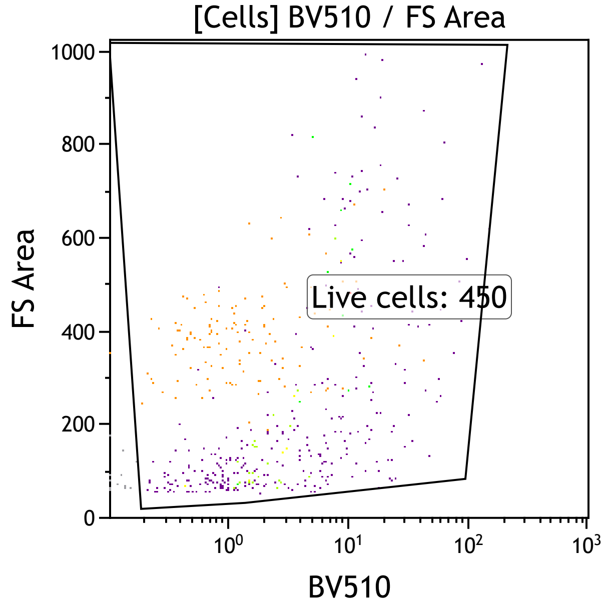

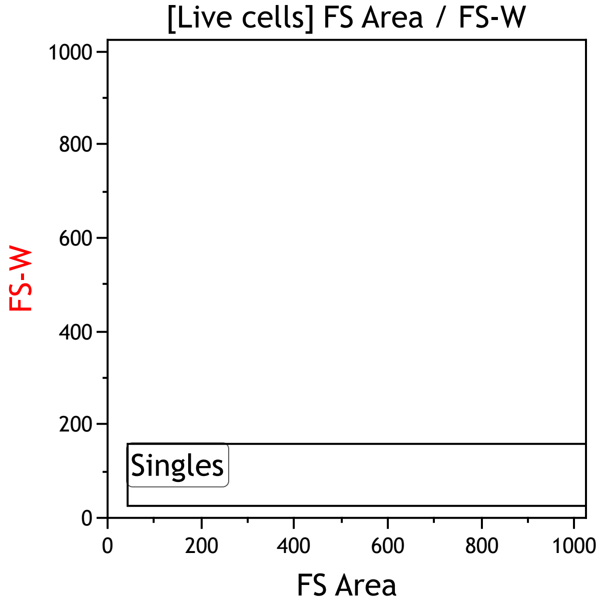

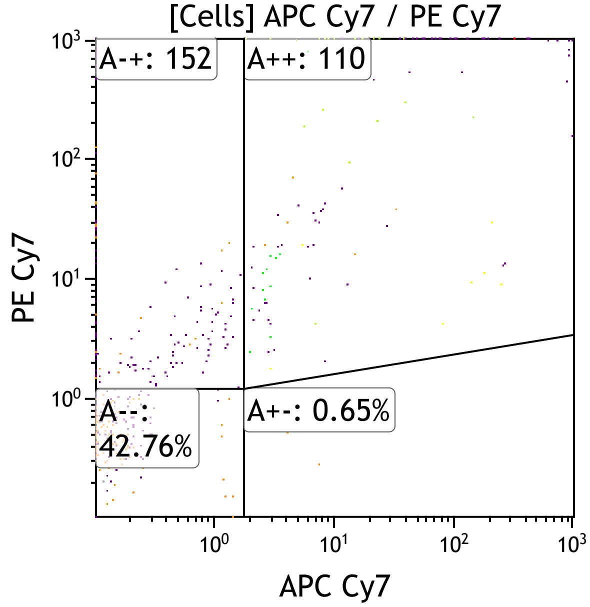

5 ipsi-2017-03-21-113701-5
Rat microglia M1M2 protocol - Imported

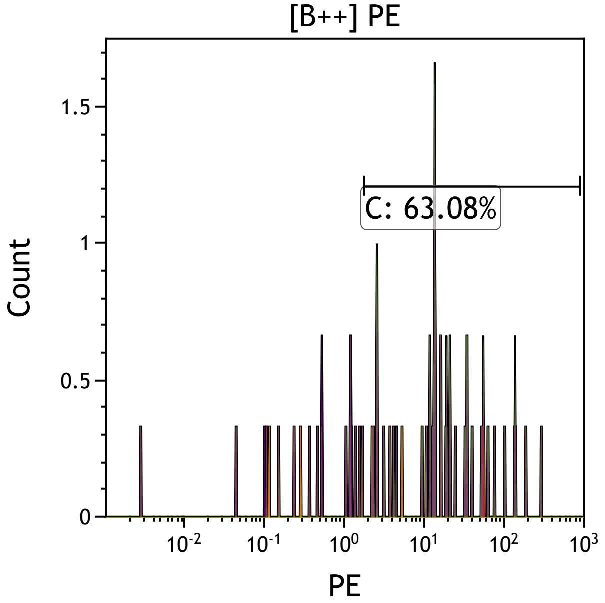

Gate	X-Med
All	4.55
C	16.33

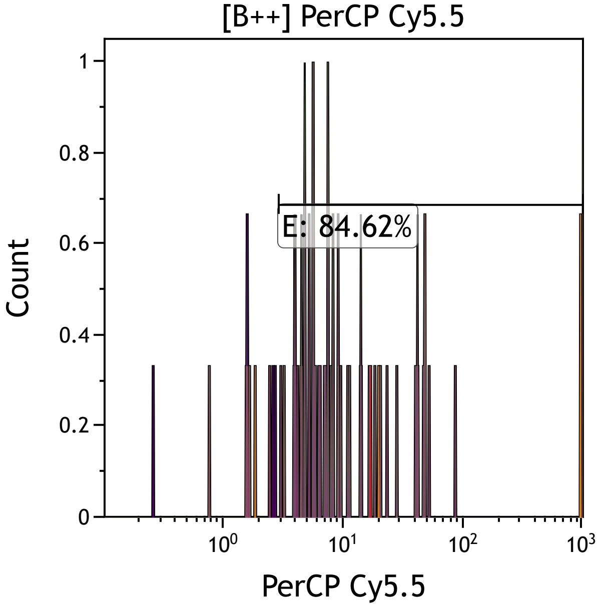

Gate	X-Med
All	7.51
E	8.36

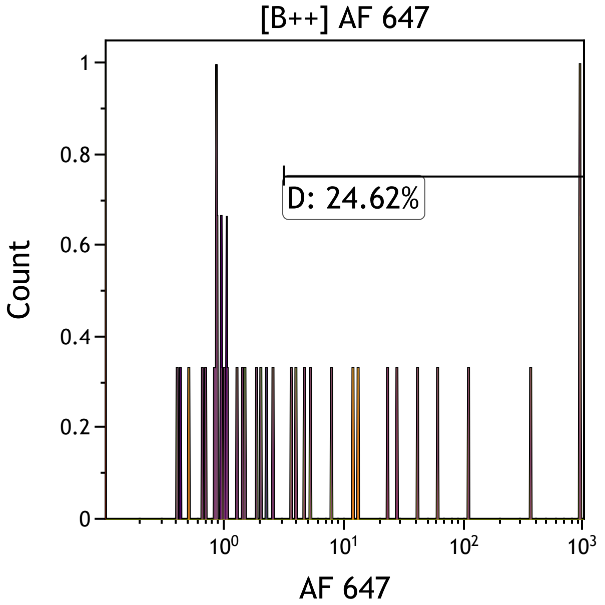

Gate	X-Med
All	0.71
D	27.79

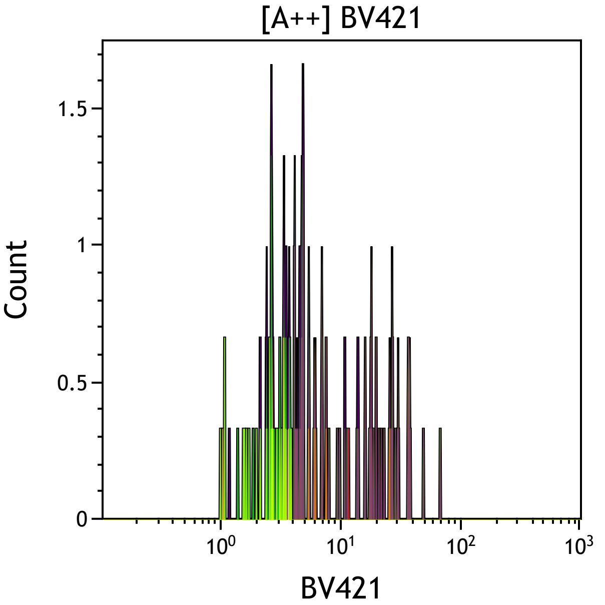

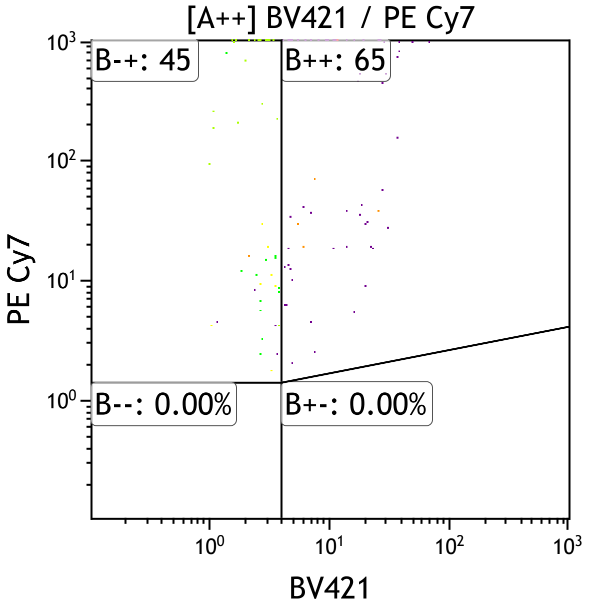

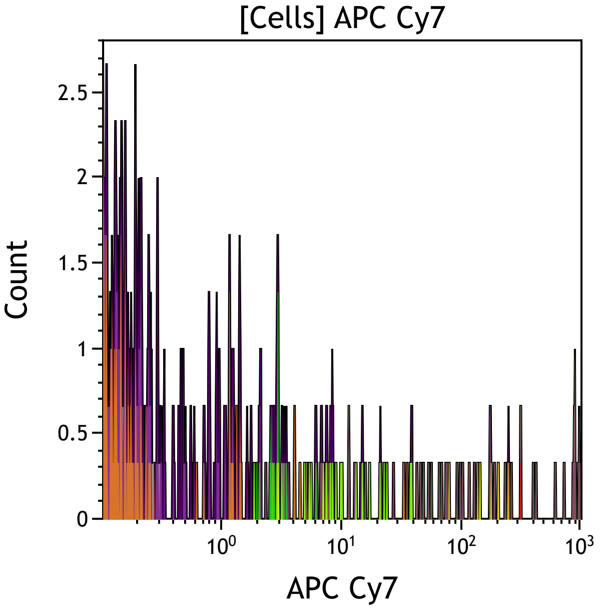

Gate	X-Med	Y-Med
All	4.70	156.28
B--	N/A	N/A
B-+	2.65	19.13
B+-	N/A	N/A
B++	10.83	461.95

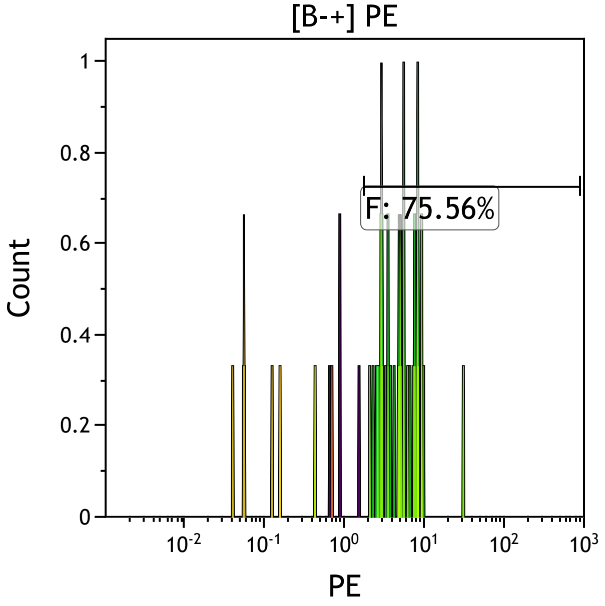

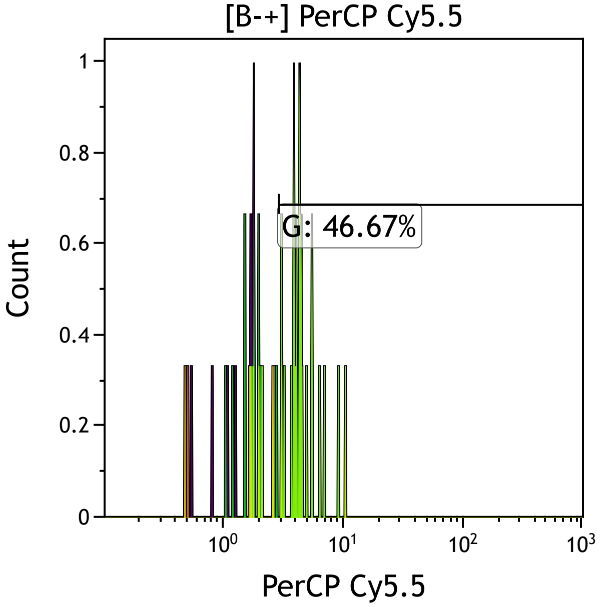

Gate	X-Med
All	3.58
F	5.17

Gate	X-Med
All	2.69
G	4.38

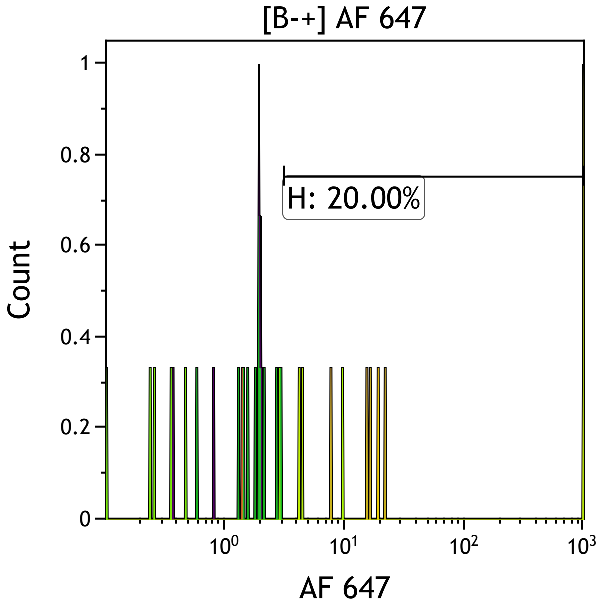

Gate	X-Med
All	1.32
H	15.70

6 ipsi-2017-03-21-113934-6

Rat microglia M1M2 protocol - Imported

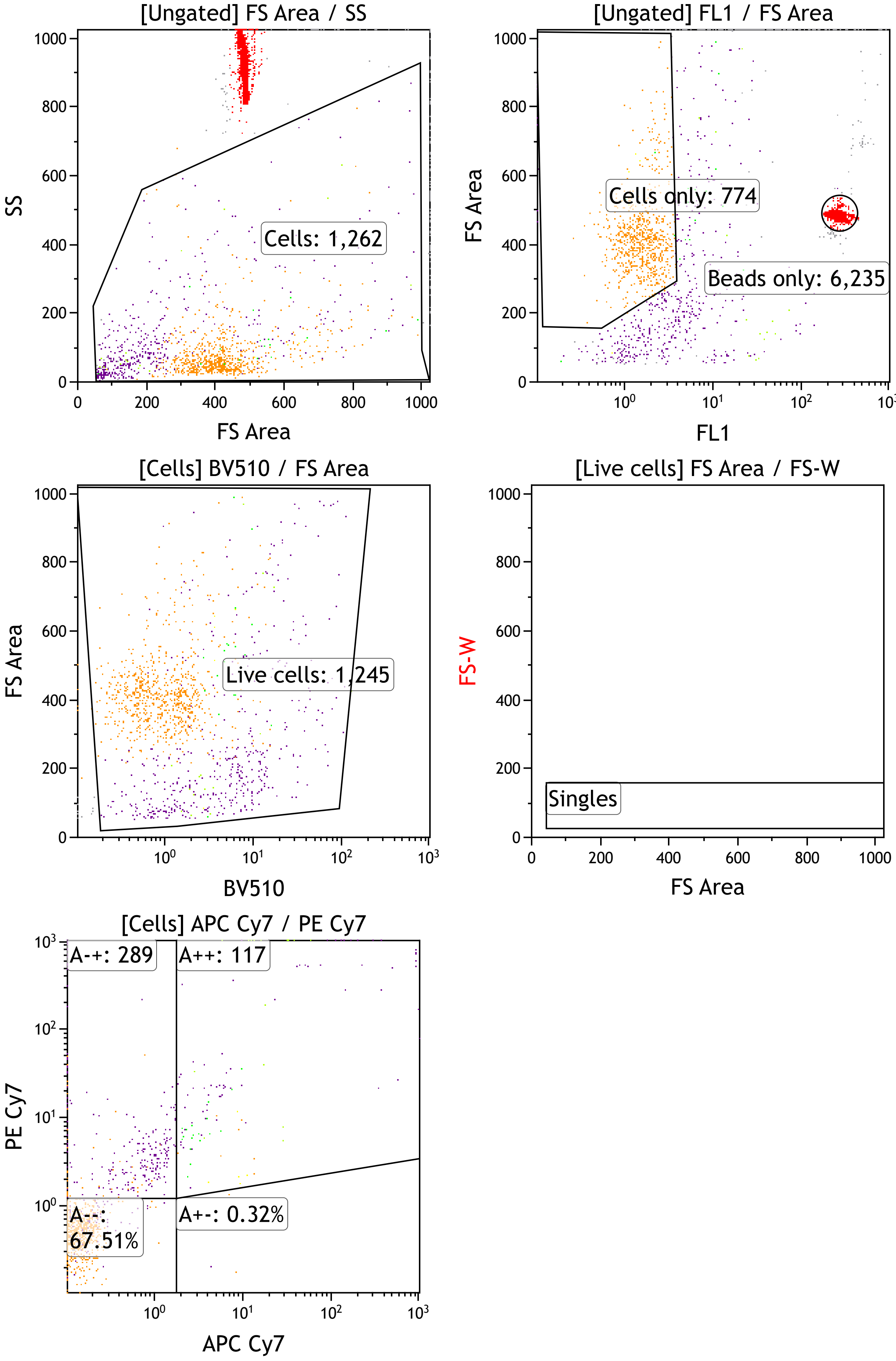

6 ipsi-2017-03-21-113934-6
Rat microglia M1M2 protocol - Imported

[B++] PE

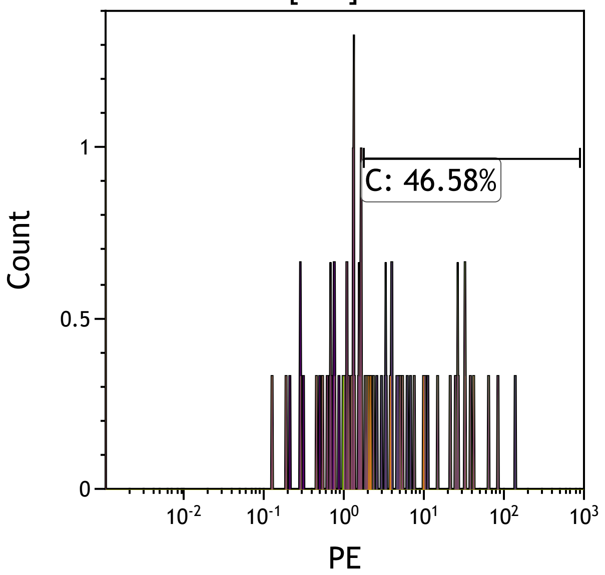

Gate	X-Med
All	1.63
C	6.77

[B++] PerCP Cy5.5

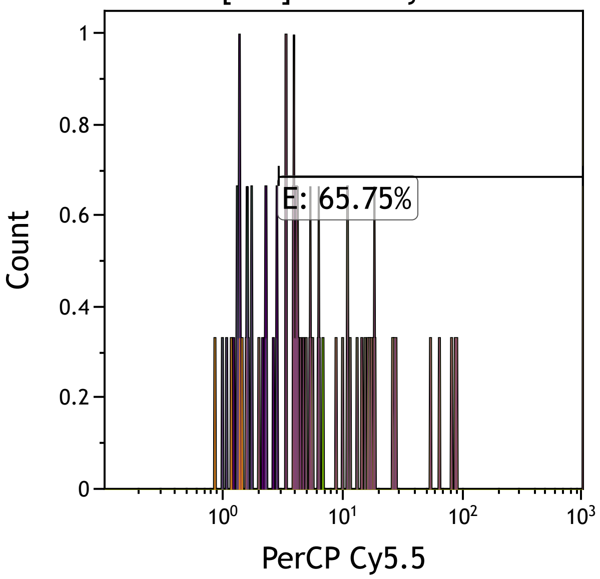

Gate	X-Med
All	4.70
E	13.17

[B++] AF 647

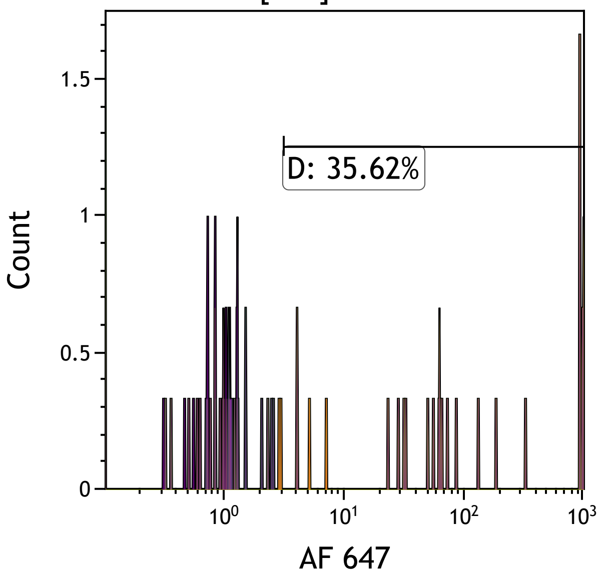

Gate	X-Med
All	1.29
D	73.41

[A++] BV421

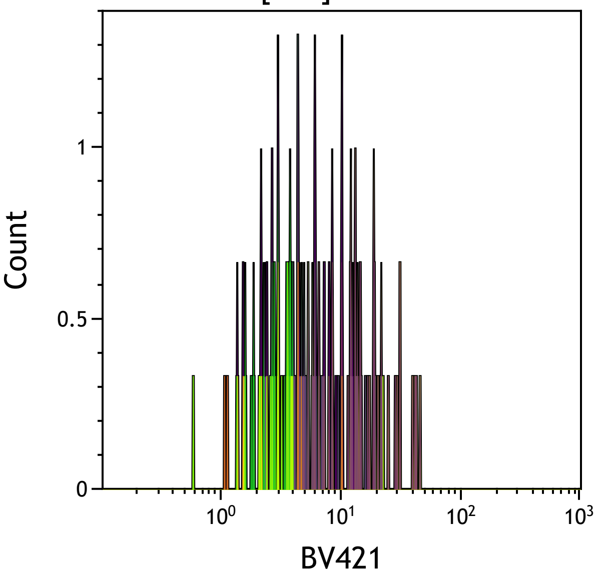

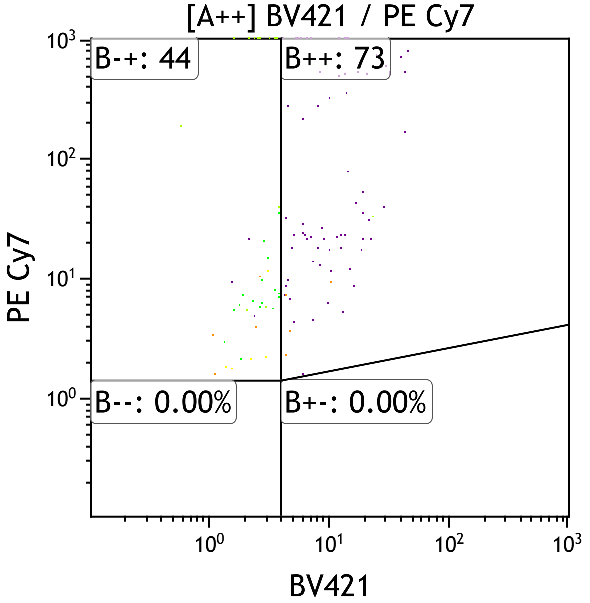

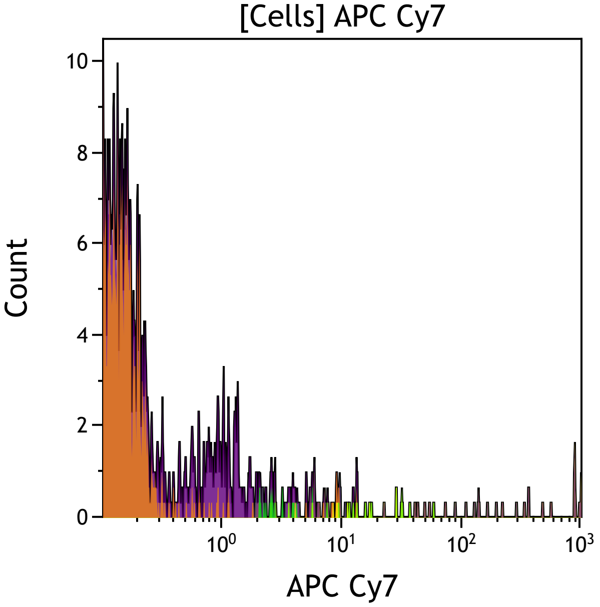

Gate	X-Med	Y-Med
All	5.38	21.70
B--	N/A	N/A
B-+	2.66	7.61
B+-	N/A	N/A
B++	10.22	30.82

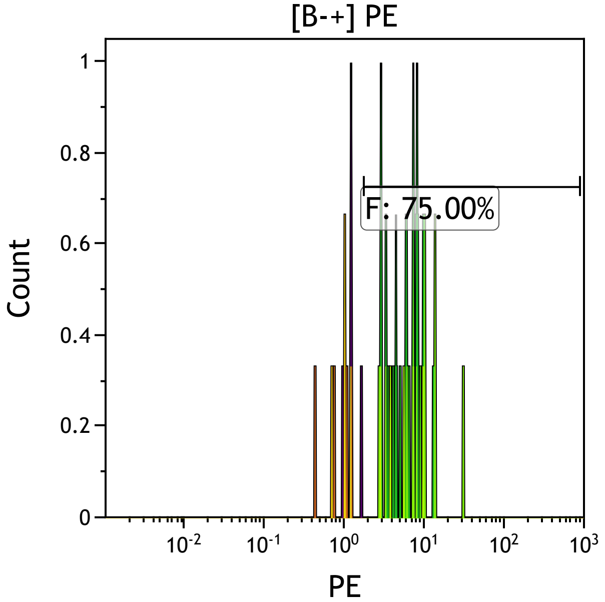

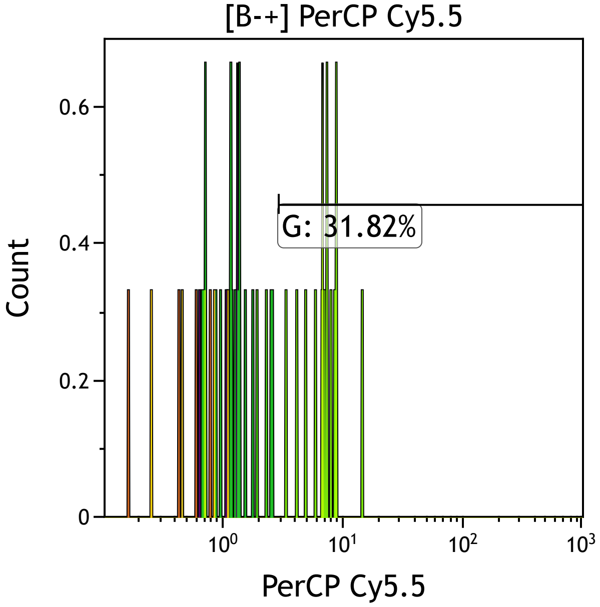

Gate	X-Med
All	5.03
F	6.55

Gate	X-Med
All	1.37
G	7.34

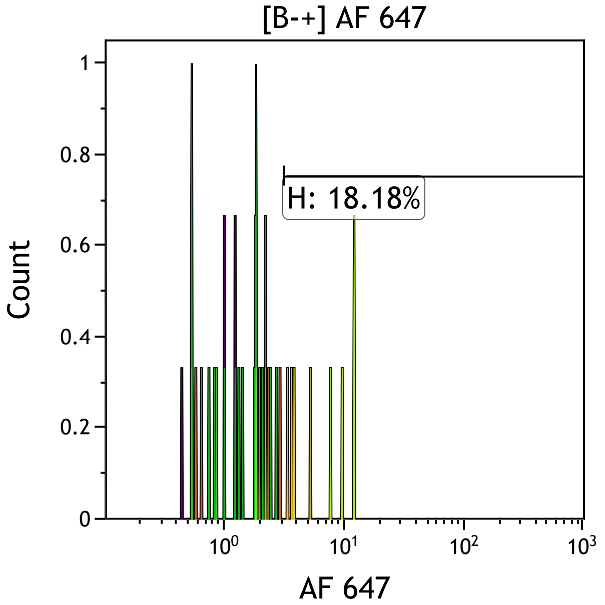

Gate	X-Med
All	1.33
H	7.75

7 ipsi-2017-03-21-114207-7

Rat microglia M1M2 protocol - Imported

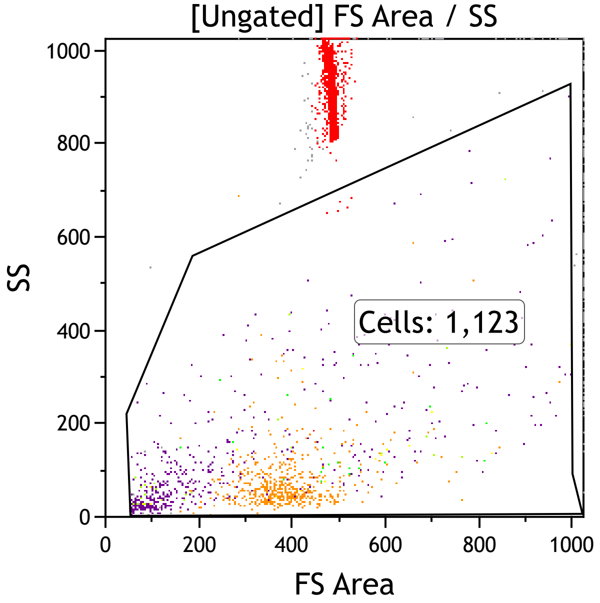

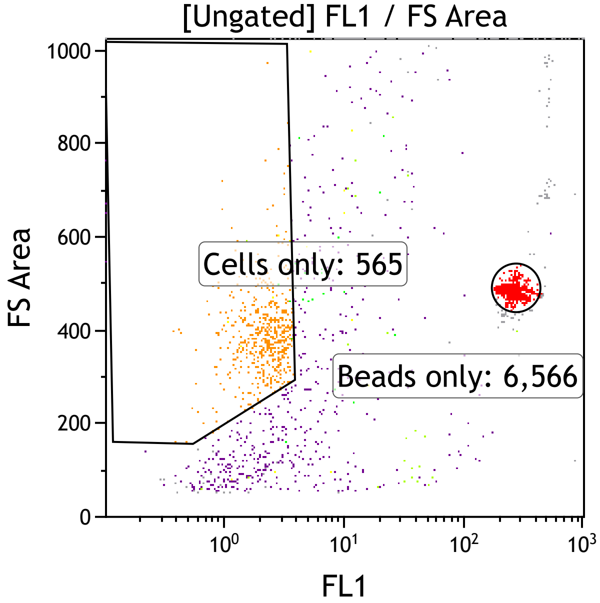

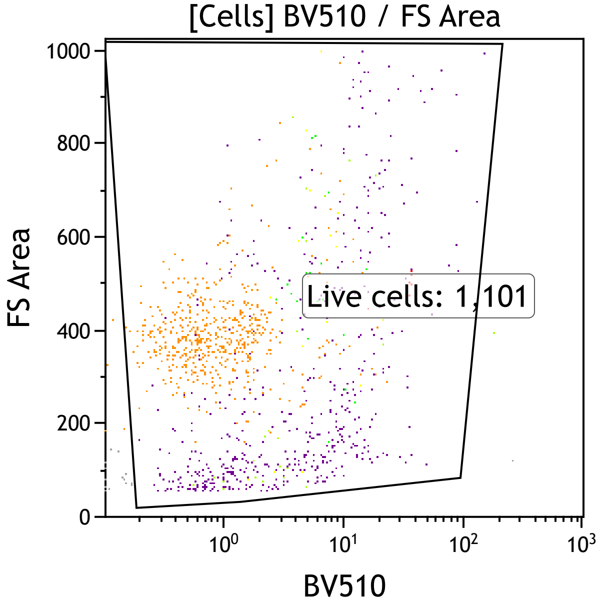

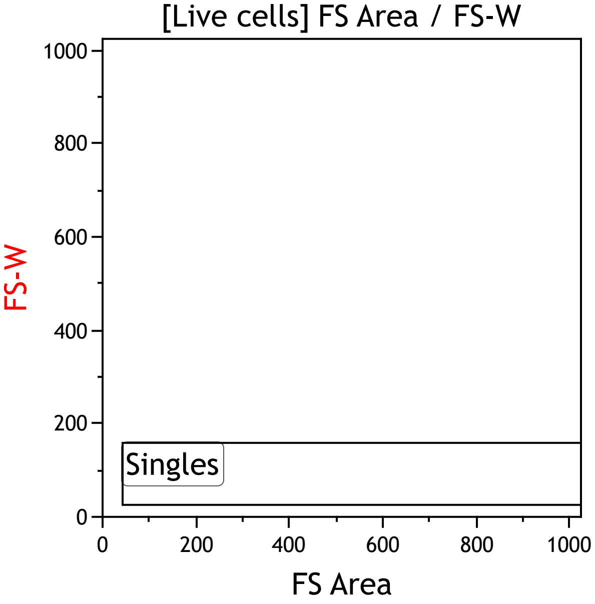

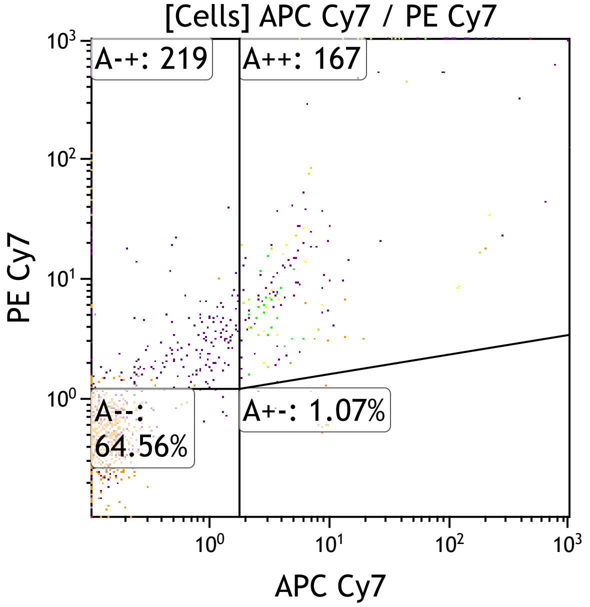

7 ipsi-2017-03-21-114207-7

Rat microglia M1M2 protocol - Imported

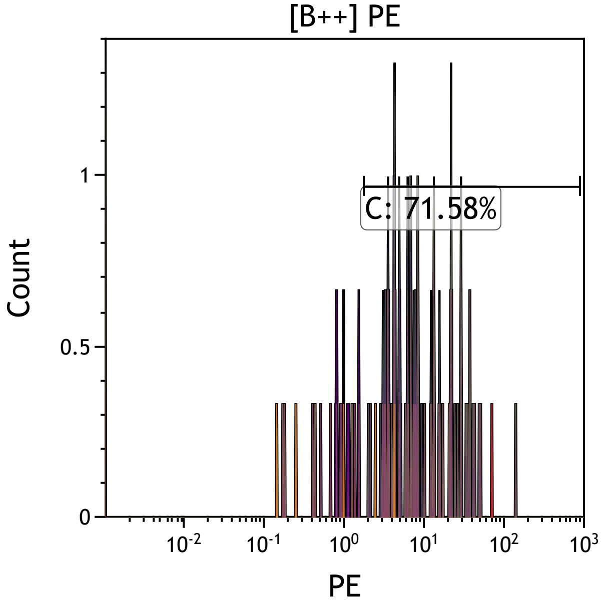

Gate	X-Med
All	4.93
C	8.18

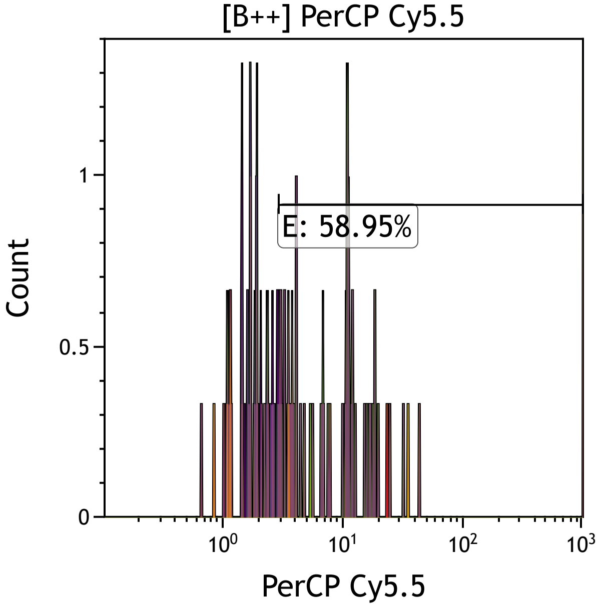

Gate	X-Med
All	3.56
E	10.91

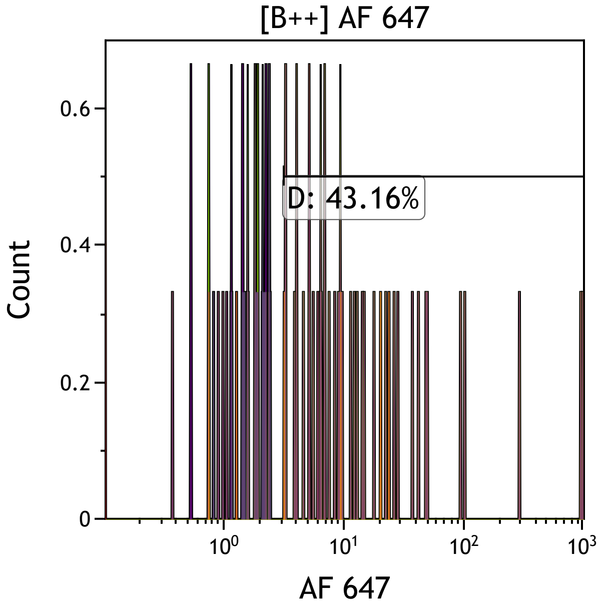

Gate	X-Med
All	2.13
D	9.75

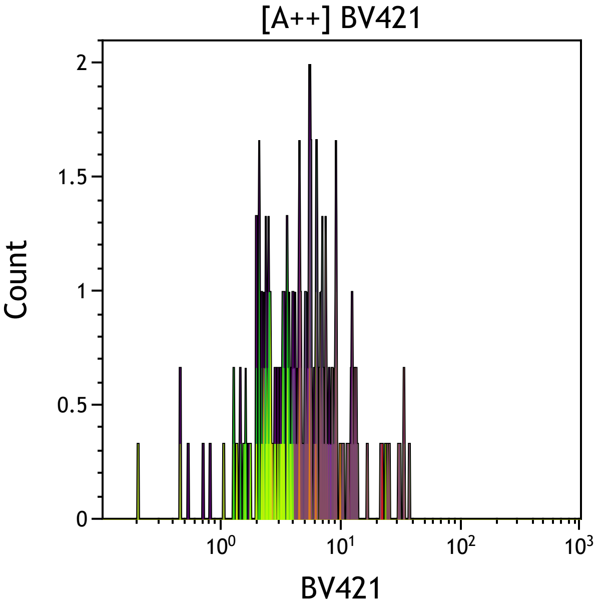

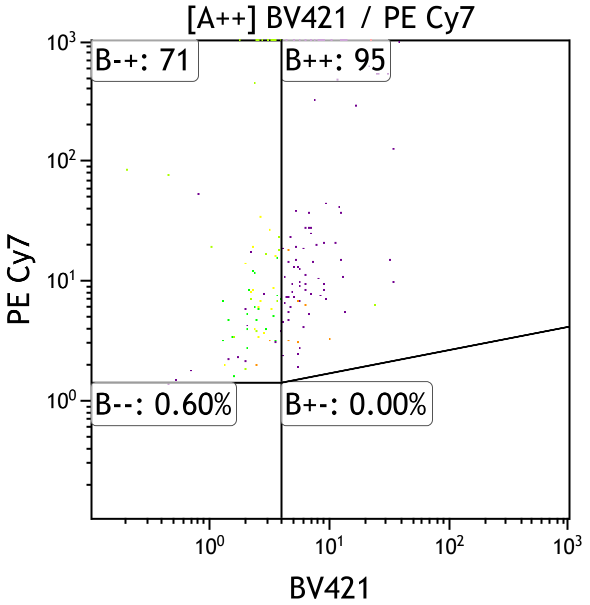

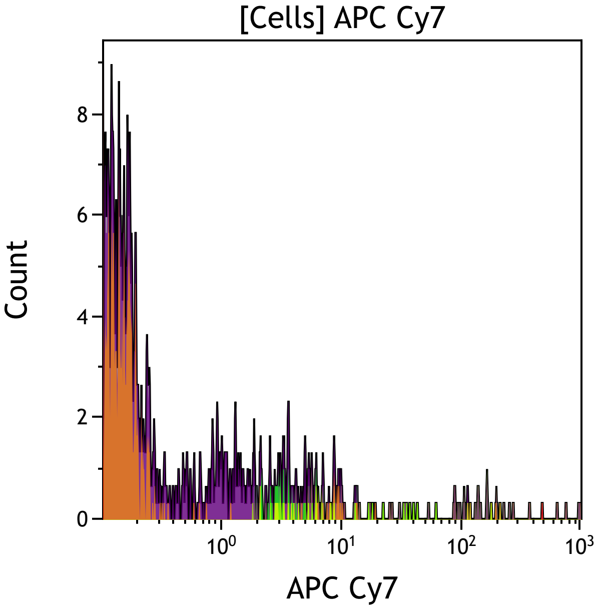

Gate	X-Med	Y-Med
All	4.58	11.46
B--	0.46	1.38
B-+	2.44	6.88
B+-	N/A	N/A
B++	6.97	17.80

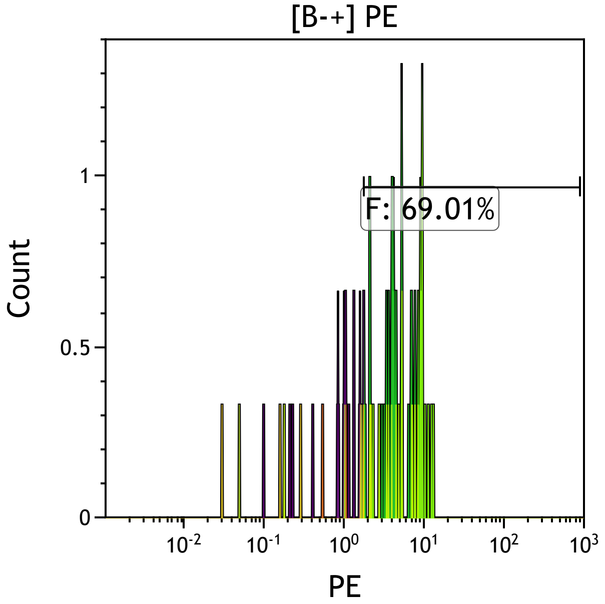

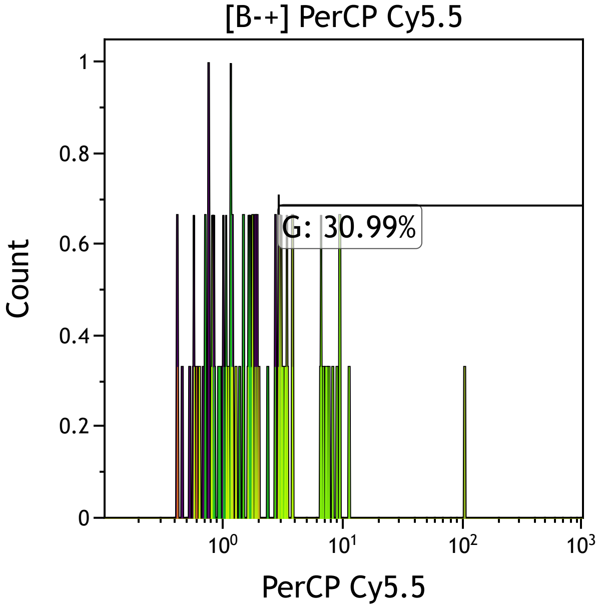

Gate	X-Med
All	3.62
F	4.55

Gate	X-Med
All	1.66
G	6.65

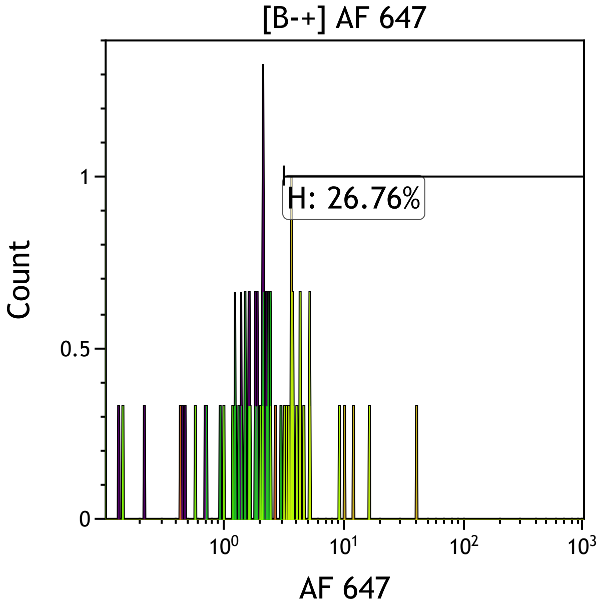

Gate	X-Med
All	1.85
H	4.33

8 ipsi-2017-03-21-114440-8
Rat microglia M1M2 protocol - Imported

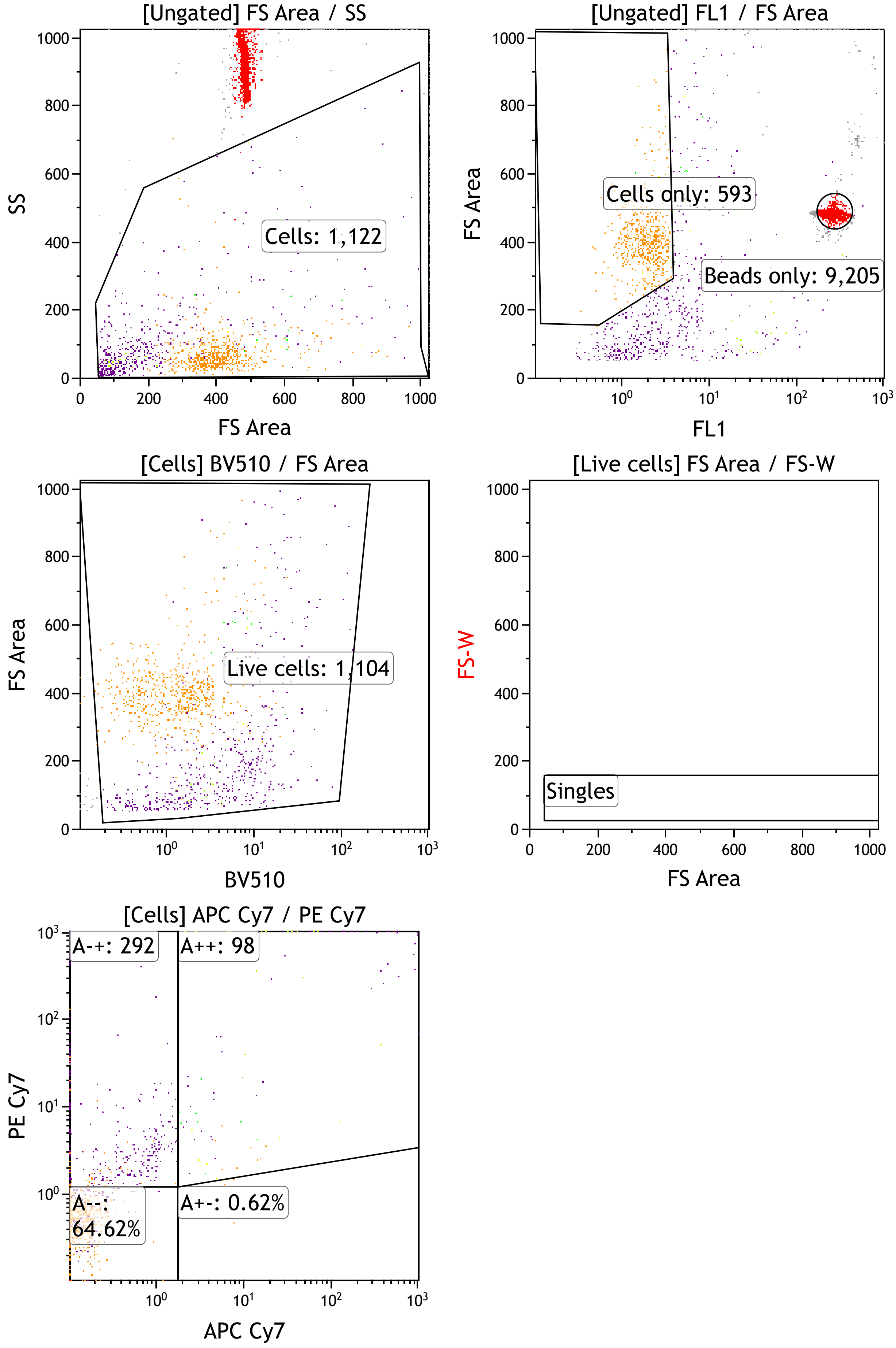

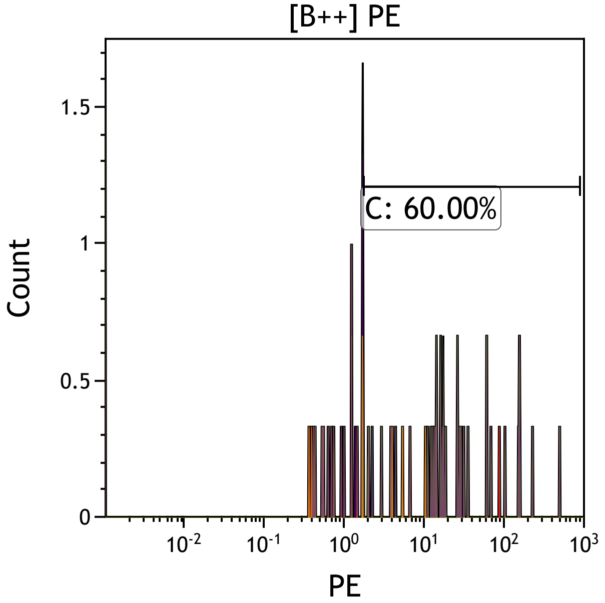

Gate	X-Med
All	5.38
C	17.65

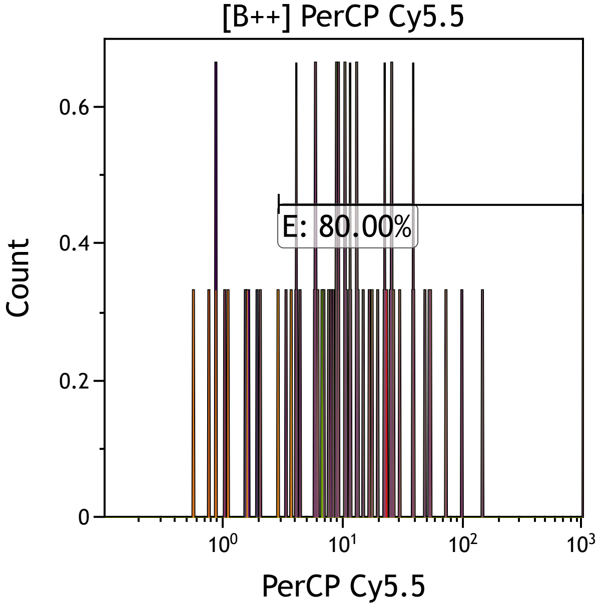

Gate	X-Med
All	10.43
E	13.11

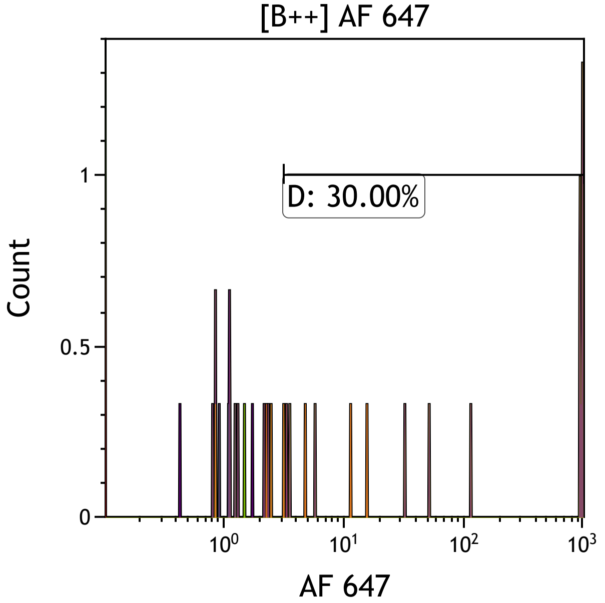

Gate	X-Med
All	0.91
D	115.10

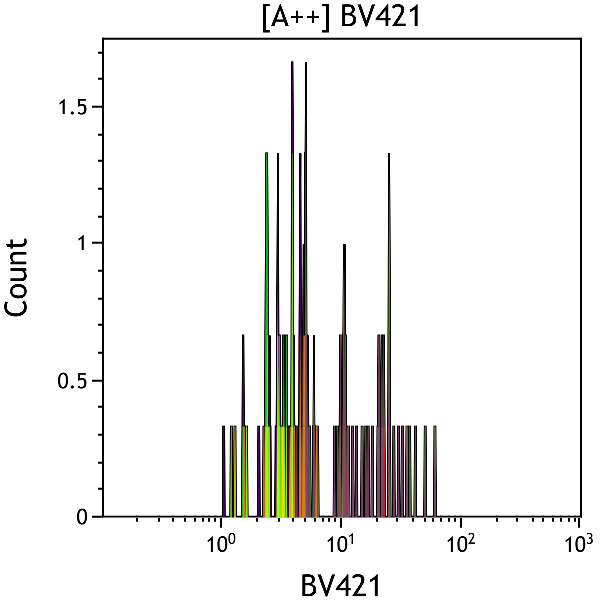

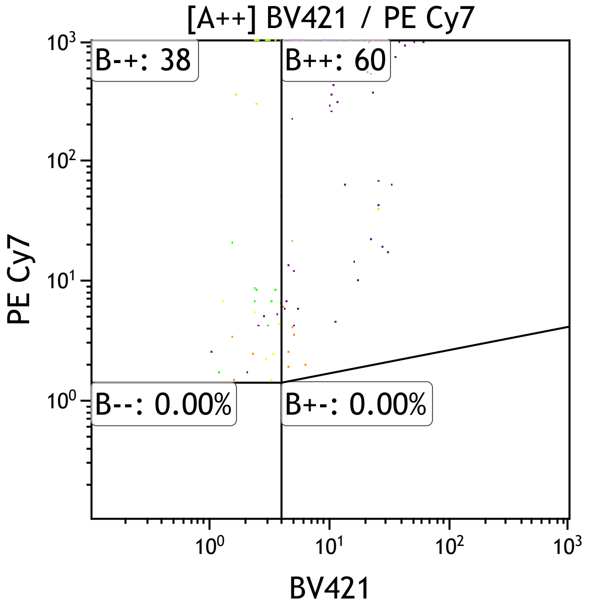

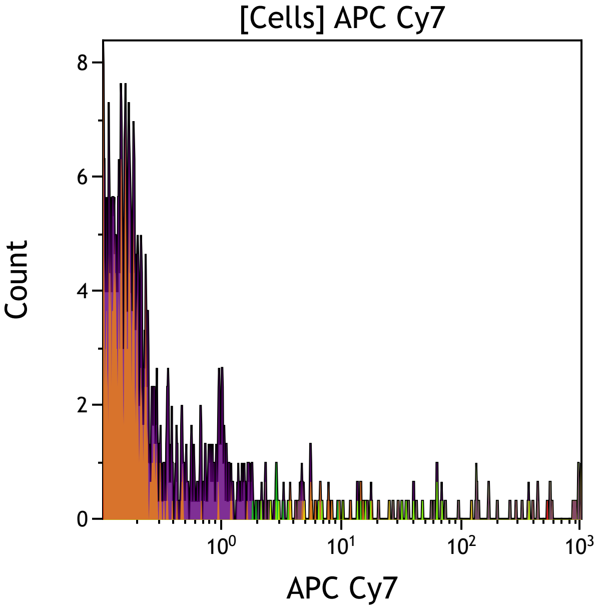

Gate	X-Med	Y-Med
All	4.99	254.00
B--	N/A	N/A
B-+	2.96	8.33
B+-	N/A	N/A
B++	10.71	416.56

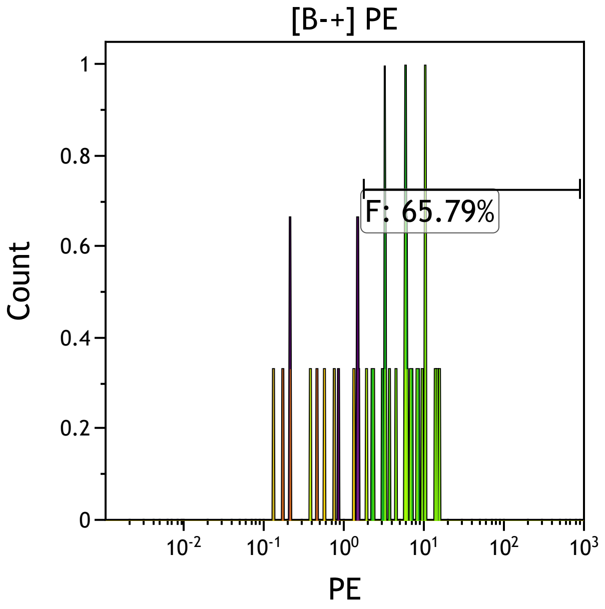

Gate	X-Med
All	3.29
F	6.04

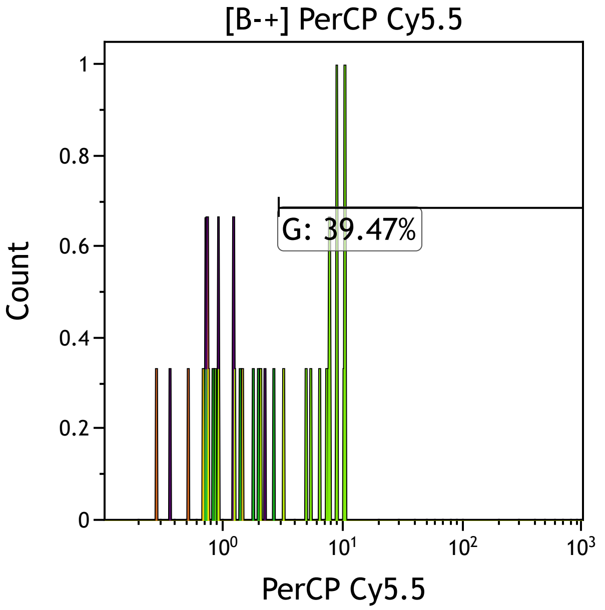

Gate	X-Med
All	1.97
G	7.85

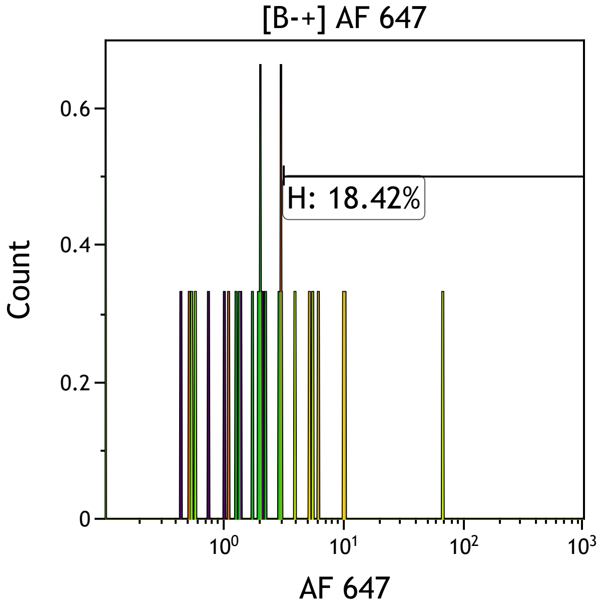

Gate	X-Med
All	1.26
H	6.16

9 ipsi-2017-03-21-114713-9
Rat microglia M1M2 protocol - Imported

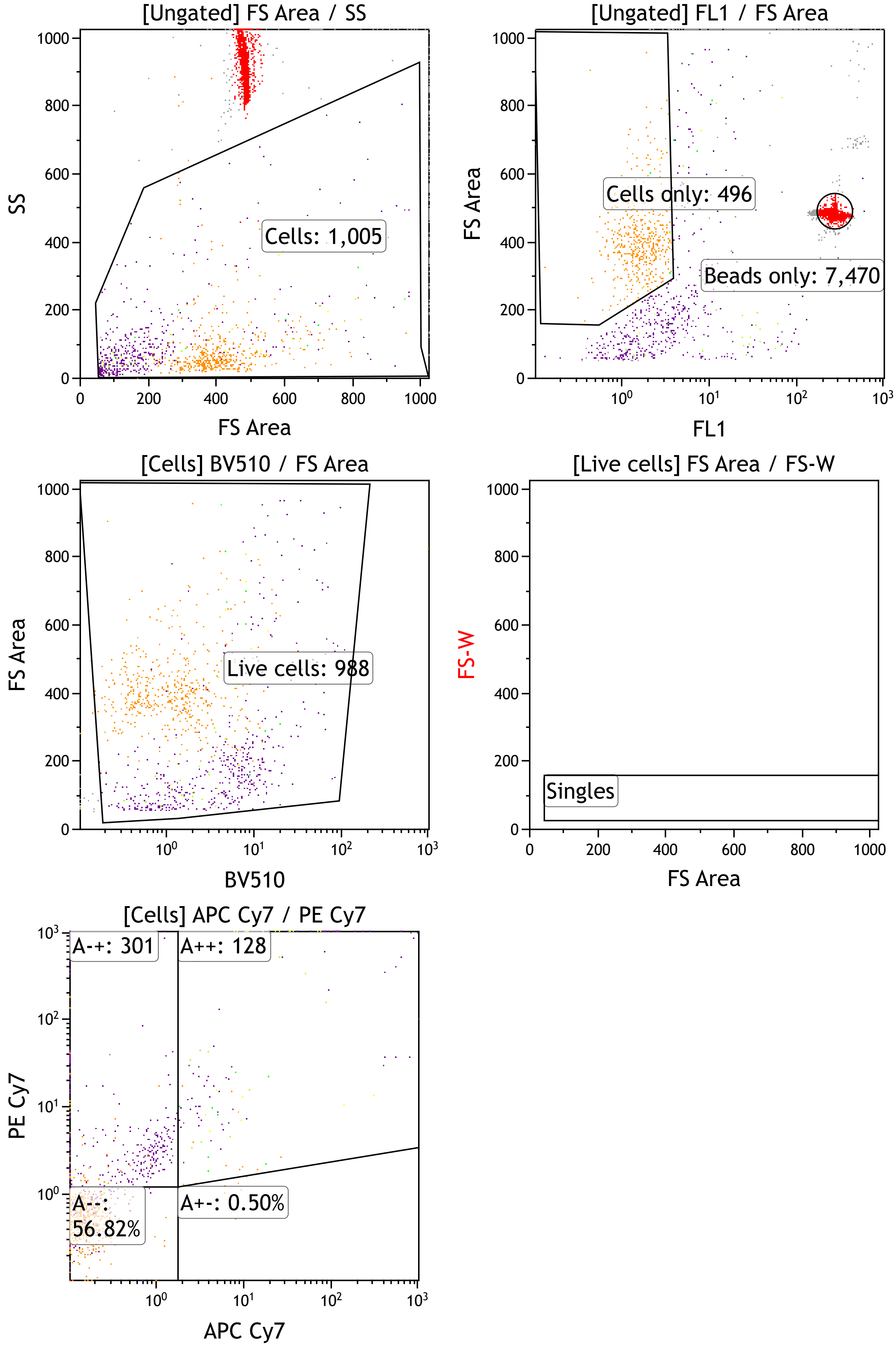

9 ipsi-2017-03-21-114713-9

Rat microglia M1M2 protocol - Imported

[B++] PE

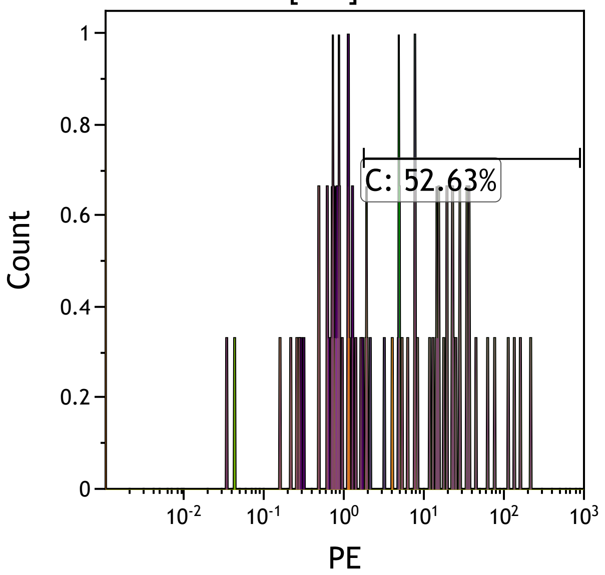

Gate	X-Med
All	2.12
C	17.65

[B++] PerCP Cy5.5

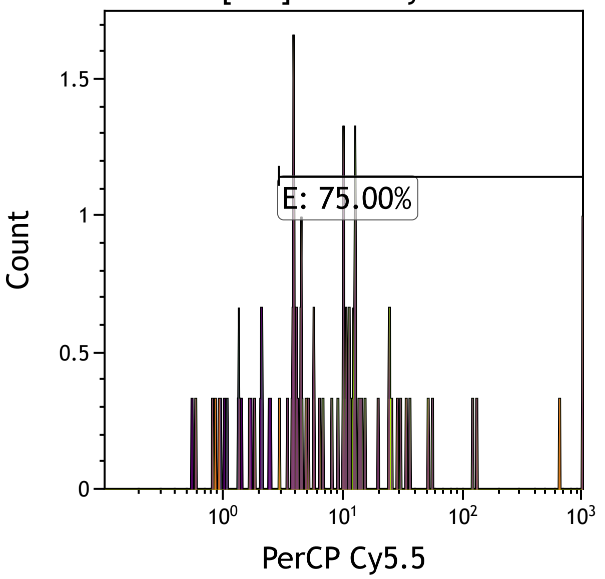

Gate	X-Med
All	6.83
E	11.25

[B++] AF 647

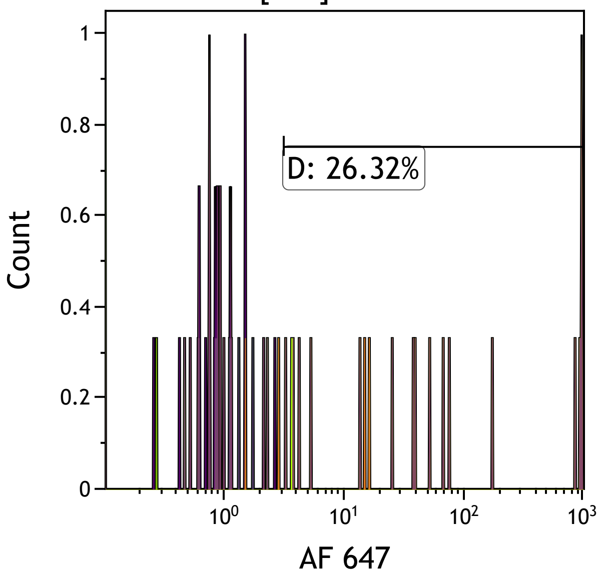

Gate	X-Med
All	0.86
D	39.47

[A++] BV421

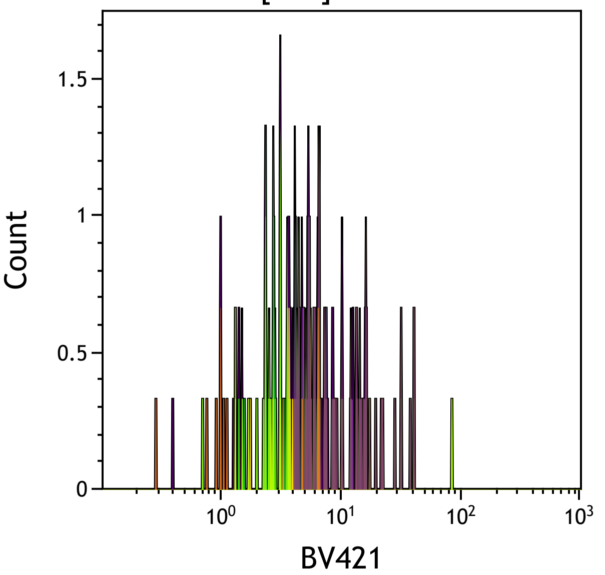

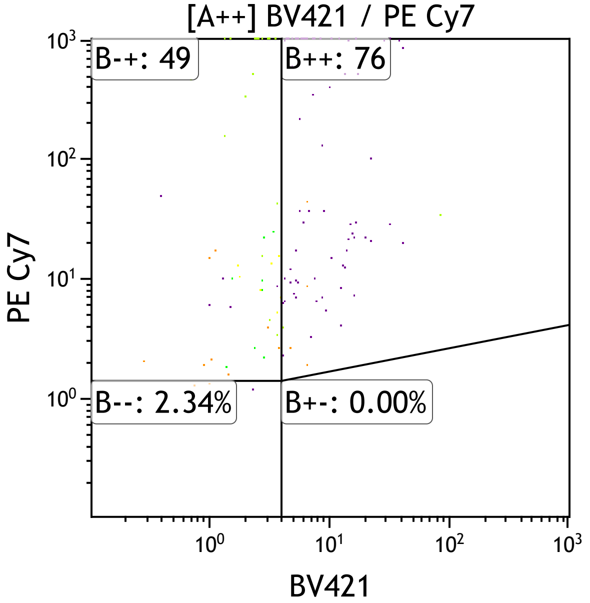

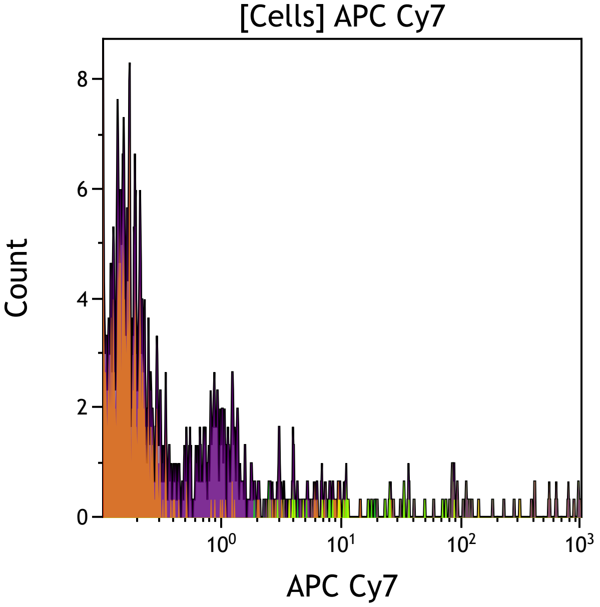

Gate	X-Med	Y-Med
All	4.79	23.64
B--	1.00	1.29
B-+	2.51	14.74
B+-	N/A	N/A
B++	7.34	36.40

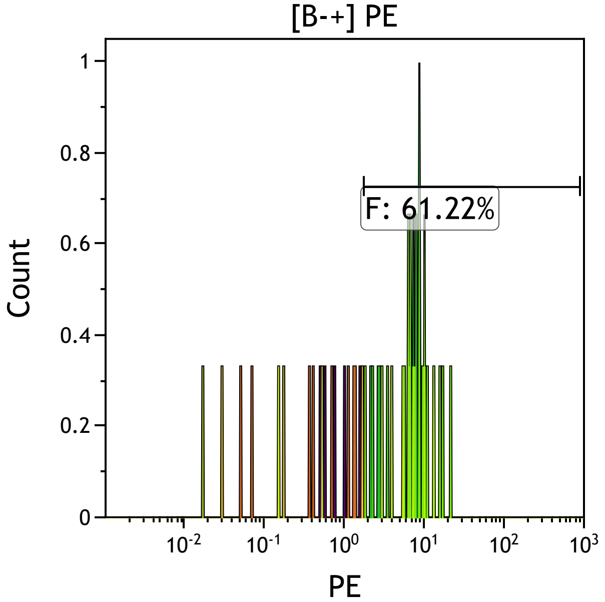

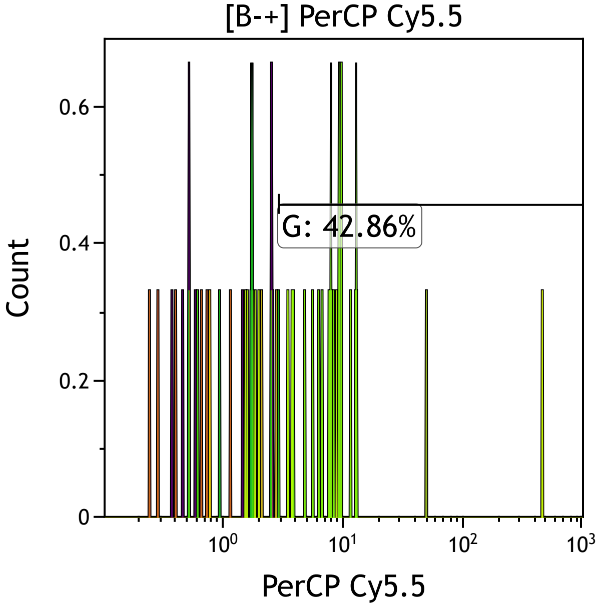

Gate	X-Med
All	2.99
F	7.44

Gate	X-Med
All	2.55
G	8.44

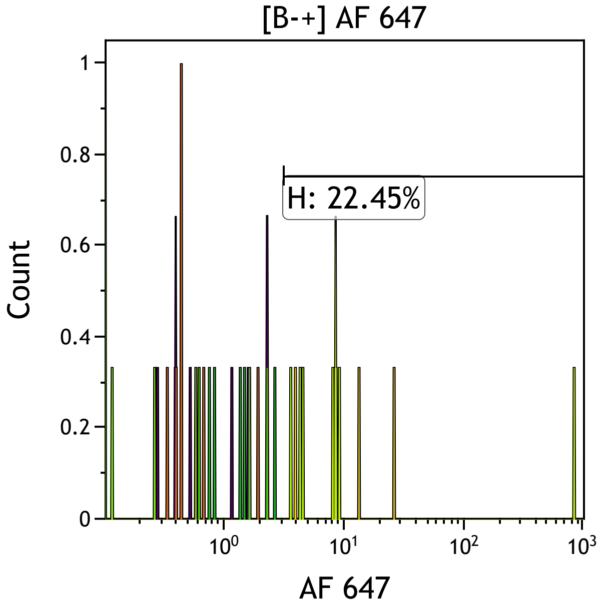

Gate	X-Med
All	0.58
H	8.52

10 ipsi-2017-03-21-114945-10
Rat microglia M1M2 protocol - Imported

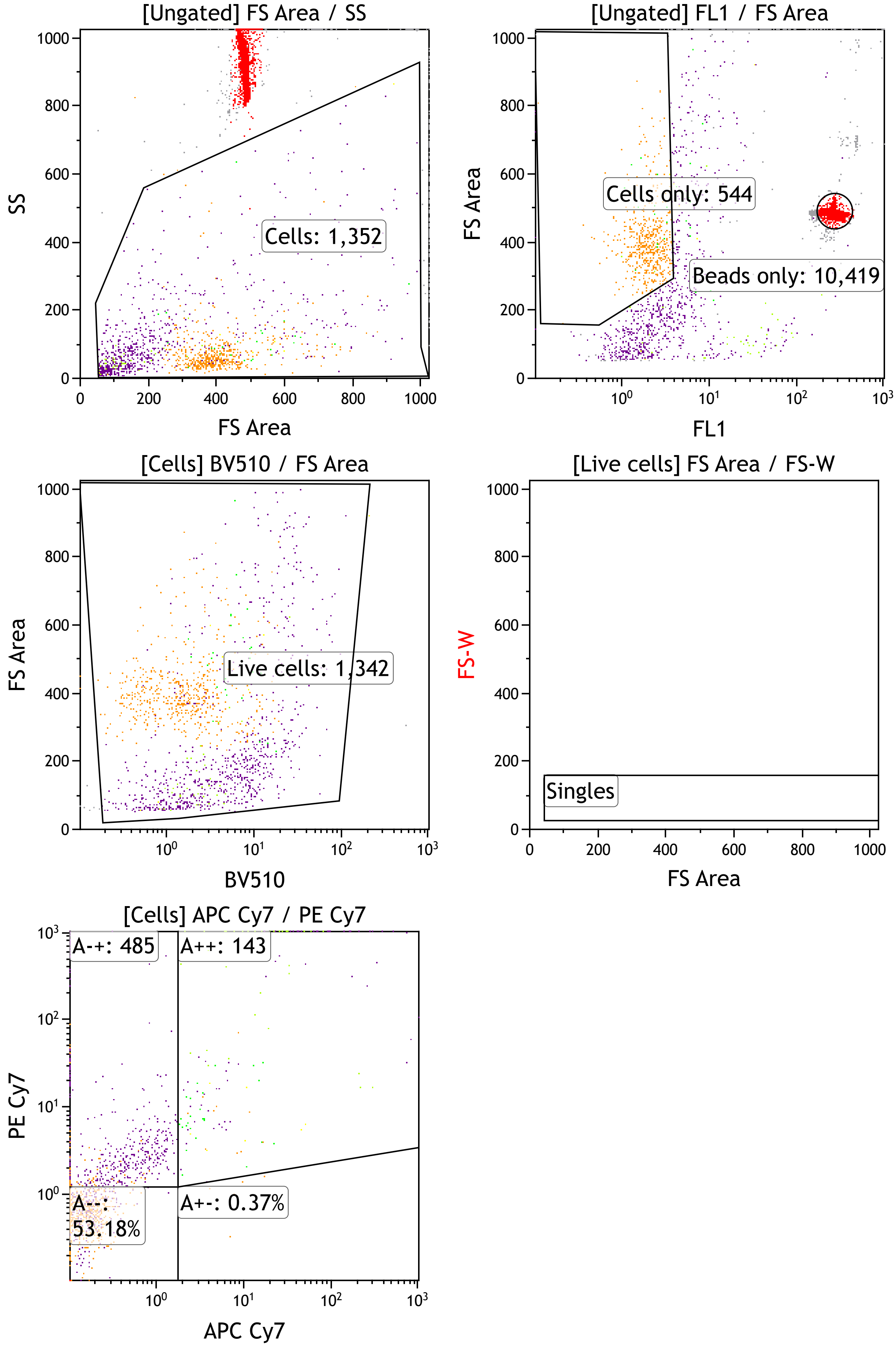

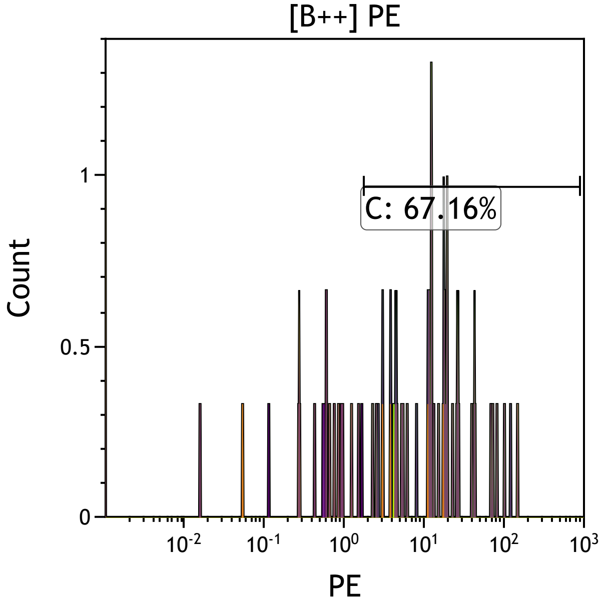

Gate	X-Med
All	5.27
C	12.57

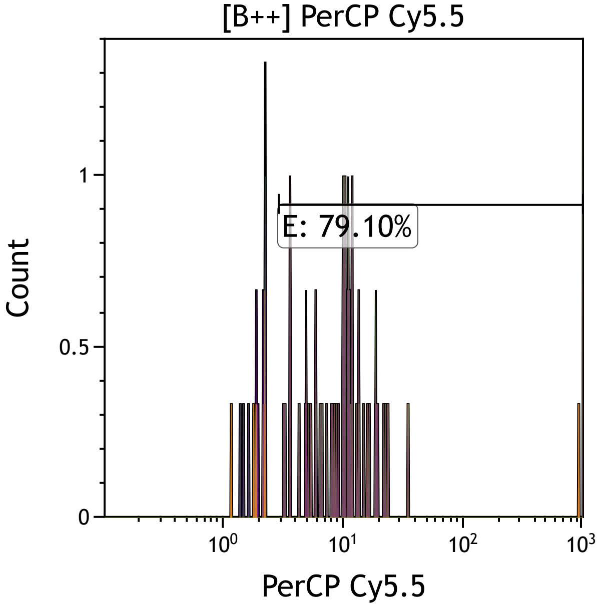

Gate	X-Med
All	9.23
E	10.57

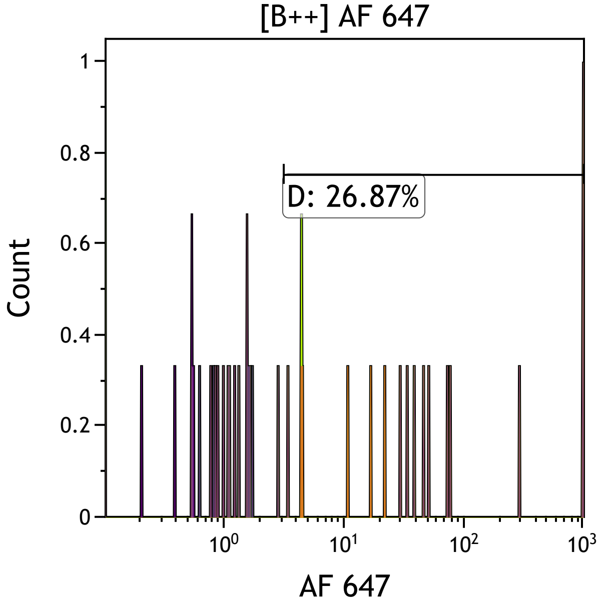

Gate	X-Med
All	0.63
D	38.76

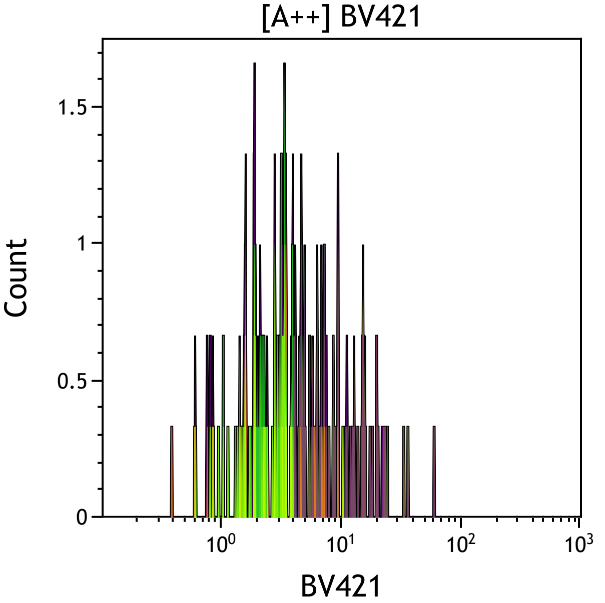

10 ipsi-2017-03-21-114945-10

Rat microglia M1M2 protocol - Imported

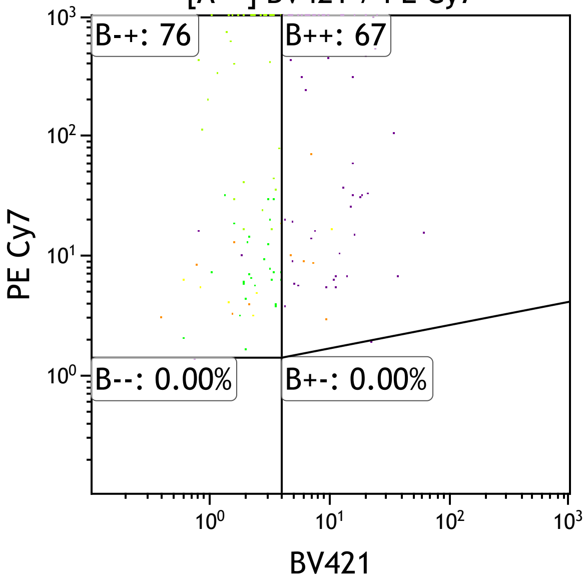

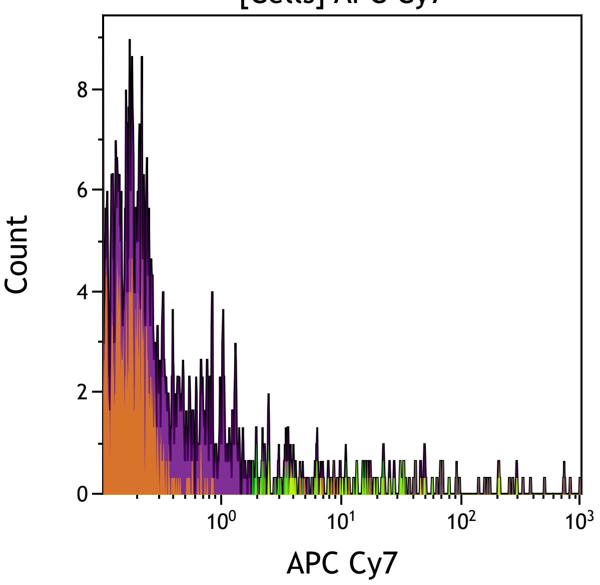

Gate	X-Med	Y-Med
All	3.82	32.53
B--	N/A	N/A
B-+	2.14	20.10
B+-	N/A	N/A
B++	8.67	302.69

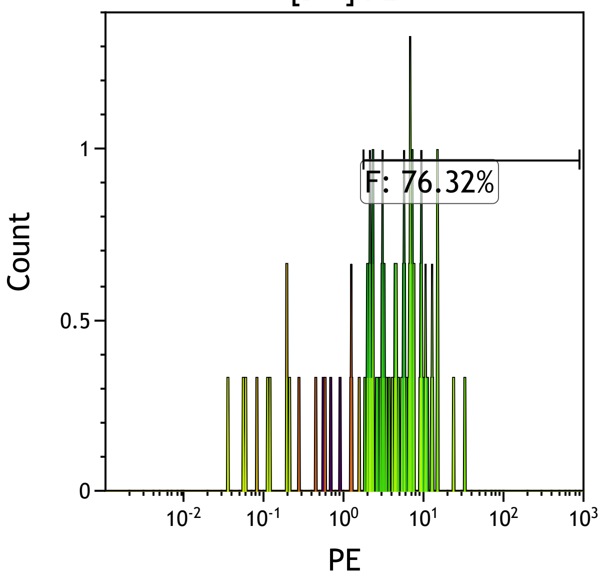

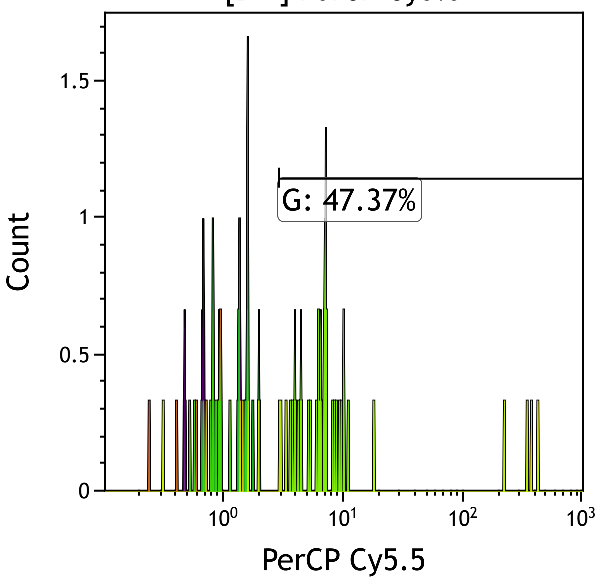

Gate	X-Med
All	3.69
F	5.61

Gate	X-Med
All	1.97
G	7.02

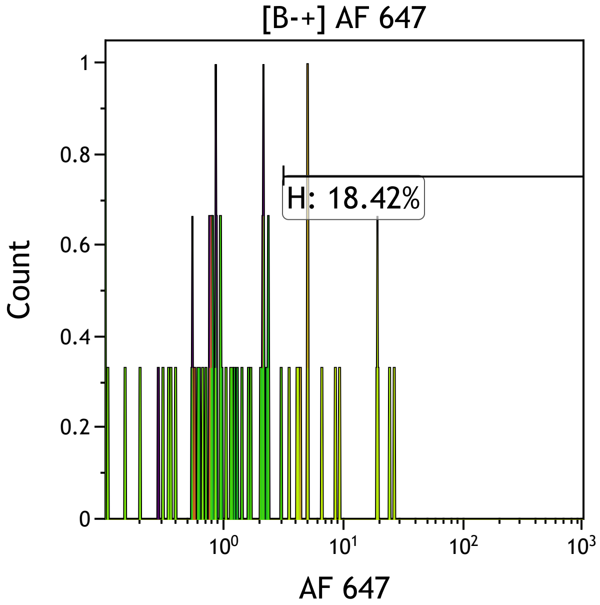

Gate	X-Med
All	0.81
H	6.59

1 contra-2017-03-21-115218-11
Rat microglia M1M2 protocol - Imported

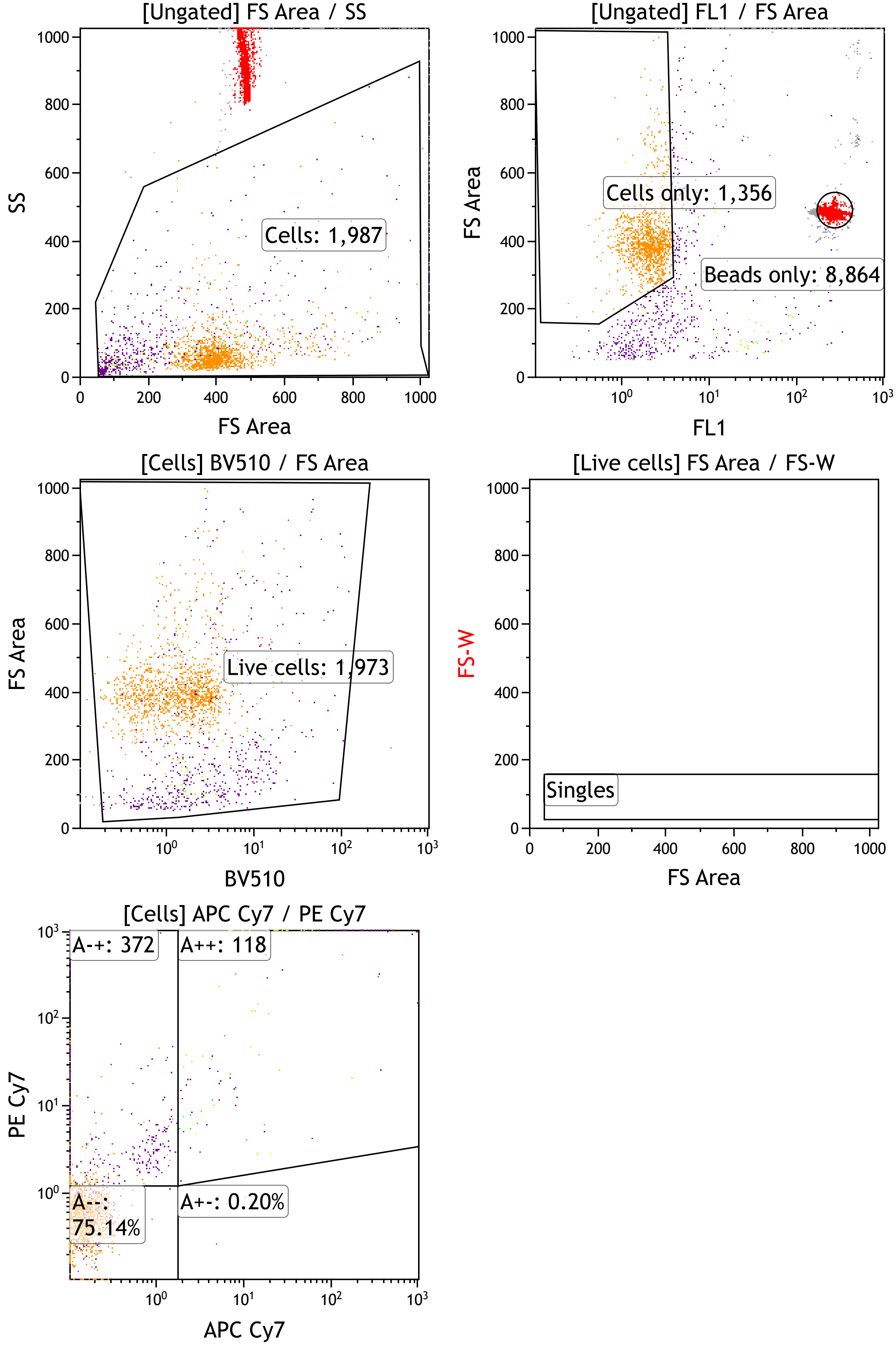

1 contra-2017-03-21-115218-11
Rat microglia M1M2 protocol - Imported

[B++] PE

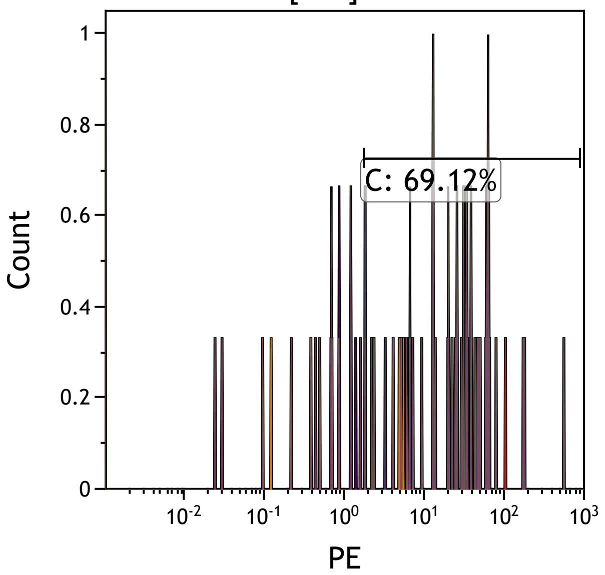

Gate	X-Med
All	9.36
C	26.27

[B++] PerCP Cy5.5

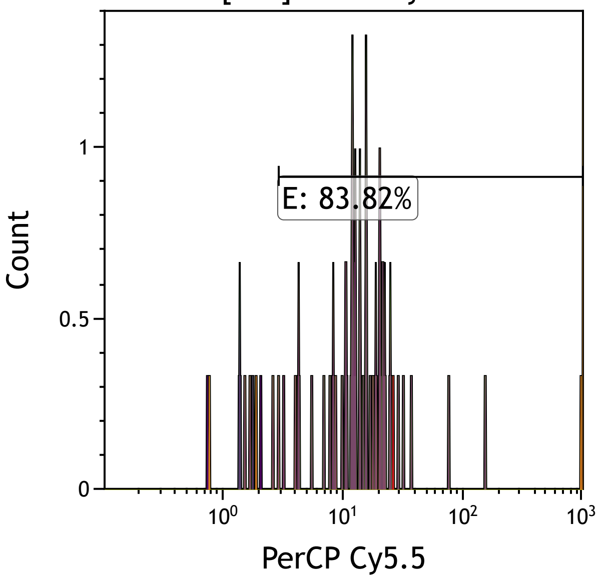

Gate	X-Med
All	13.78
E	15.70

[B++] AF 647

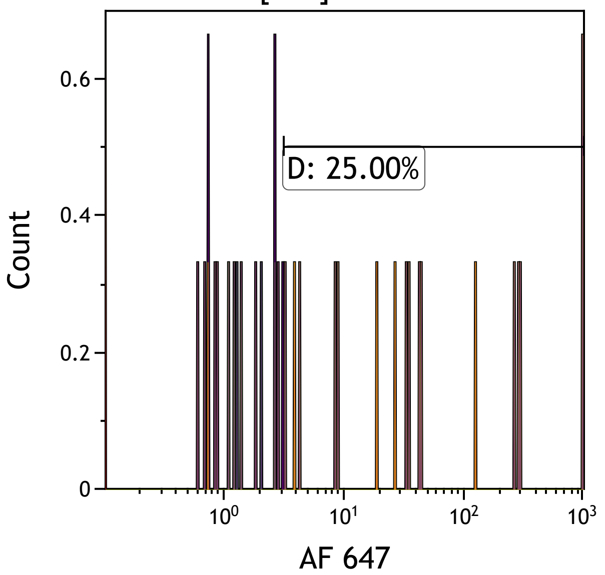

Gate	X-Med
All	0.10
D	35.27

[A++] BV421

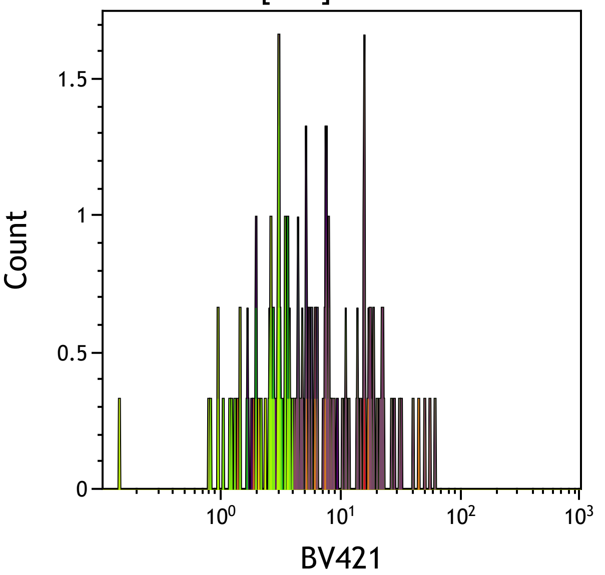

1 contra-2017-03-21-115218-11
Rat microglia M1M2 protocol - Imported

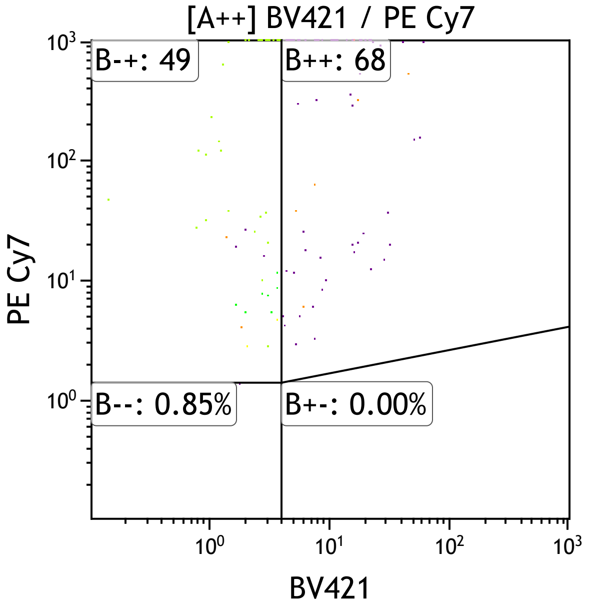

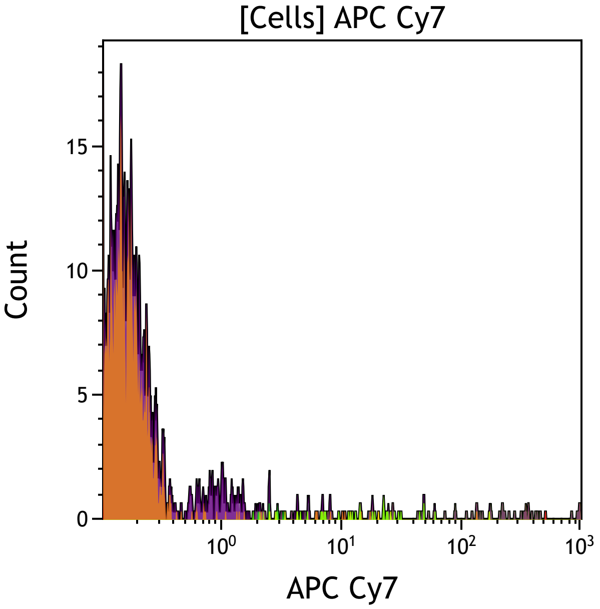

Gate	X-Med	Y-Med
All	5.12	320.92
B--	1.81	1.37
B-+	2.63	112.54
B+-	N/A	N/A
B++	10.33	961.52

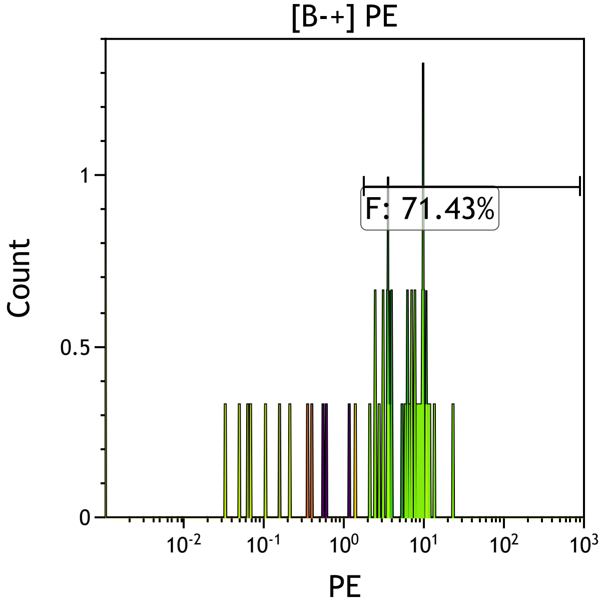

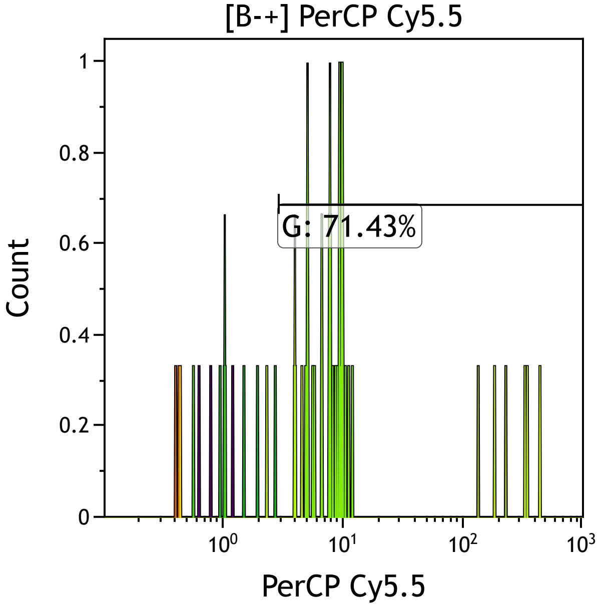

Gate	X-Med
All	3.92
F	7.00

Gate	X-Med
All	6.74
G	9.07

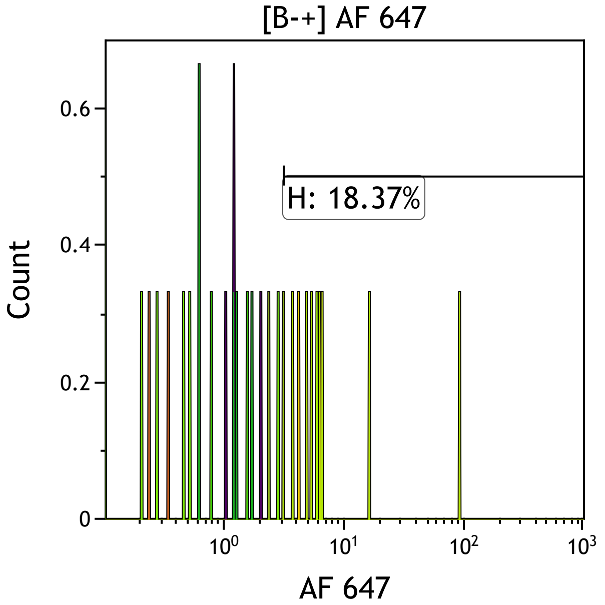

Gate	X-Med
All	0.34
H	6.00

2 contra-2017-03-21-115451-12
Rat microglia M1M2 protocol - Imported

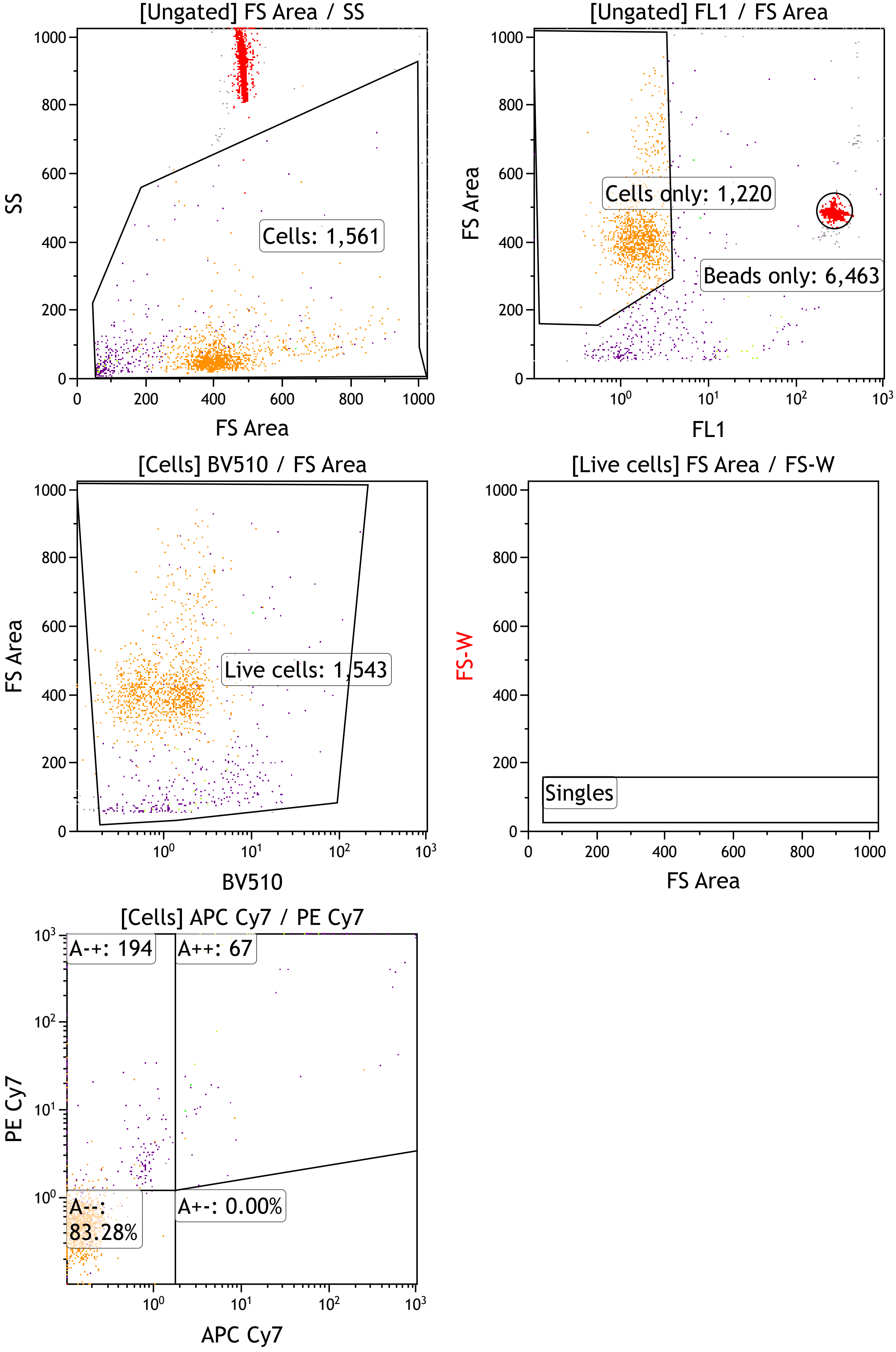

2 contra-2017-03-21-115451-12
Rat microglia M1M2 protocol - Imported

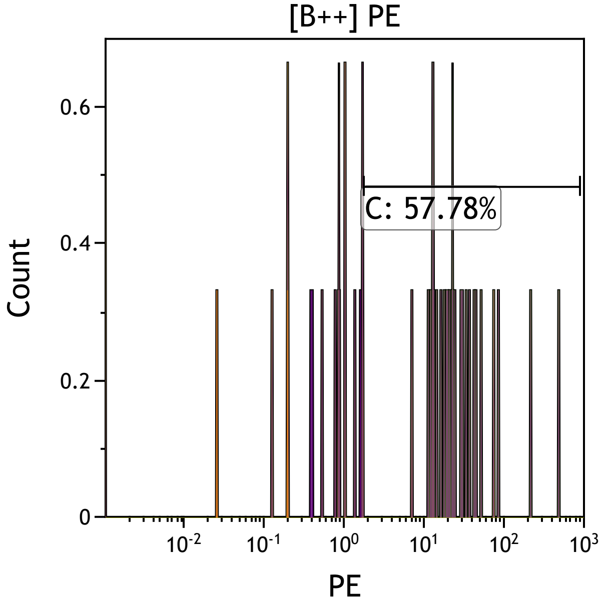

Gate	X-Med
All	12.85
C	23.11

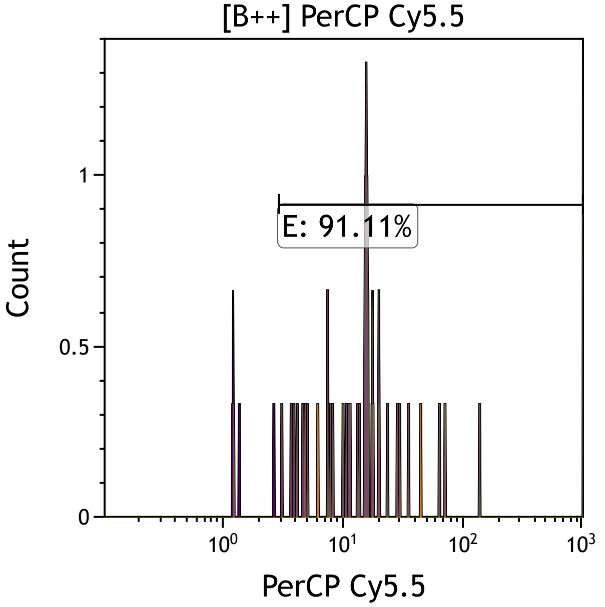

Gate	X-Med
All	15.28
E	15.56

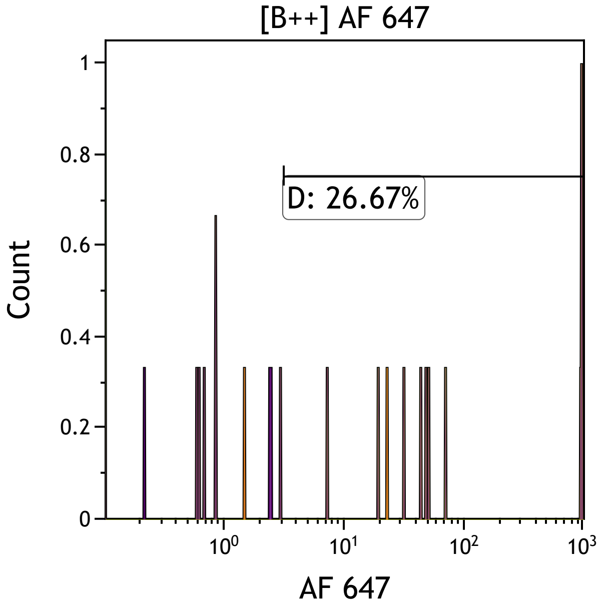

Gate	X-Med
All	0.10
D	51.23

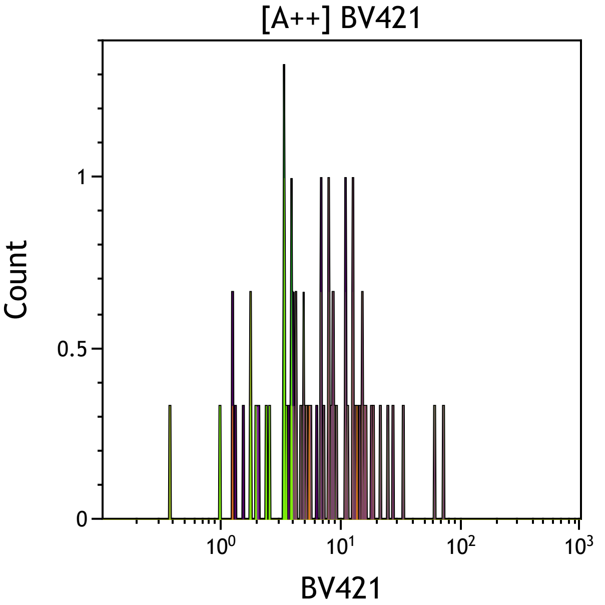

2 contra-2017-03-21-115451-12
Rat microglia M1M2 protocol - Imported

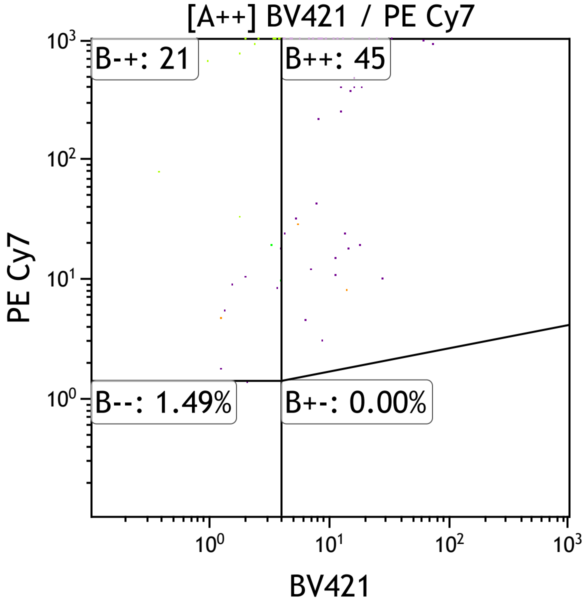

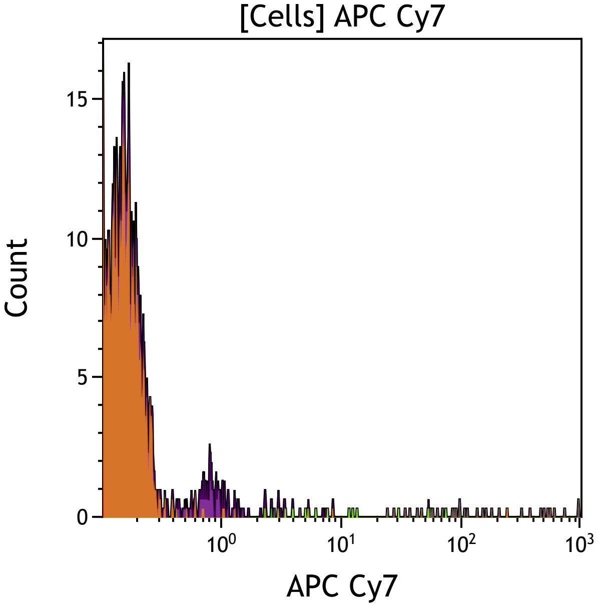

Gate	X-Med	Y-Med
All	6.85	650.18
B--	2.08	1.39
B-+	2.40	650.18
B+-	N/A	N/A
B++	9.23	915.11

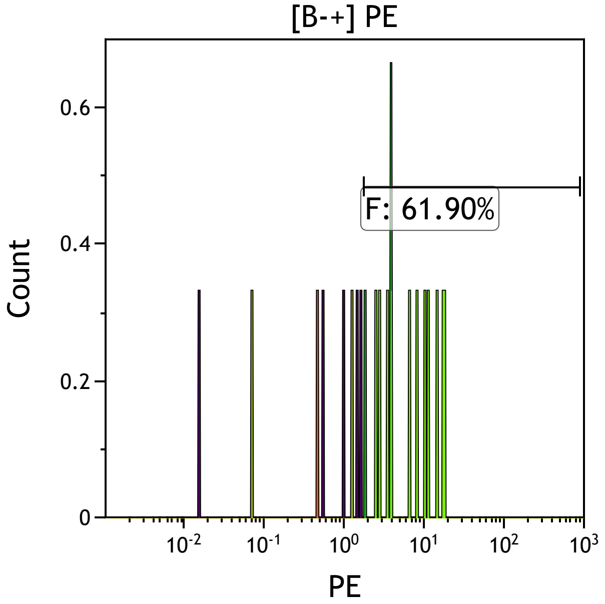

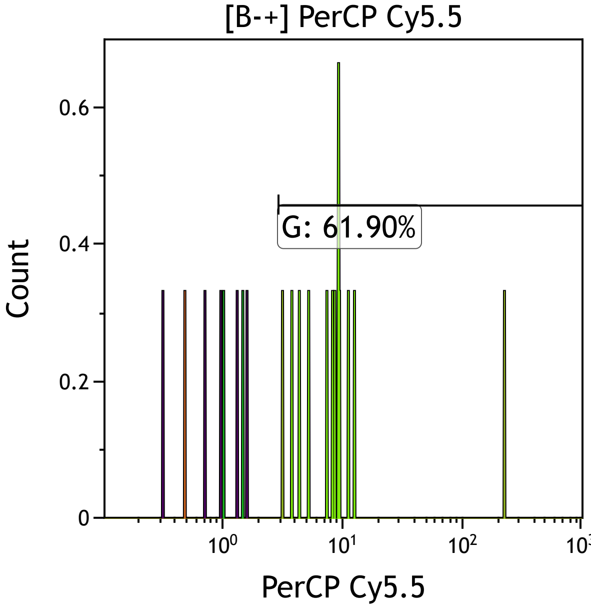

Gate	X-Med
All	2.80
F	6.63

Gate	X-Med
All	4.38
G	8.67

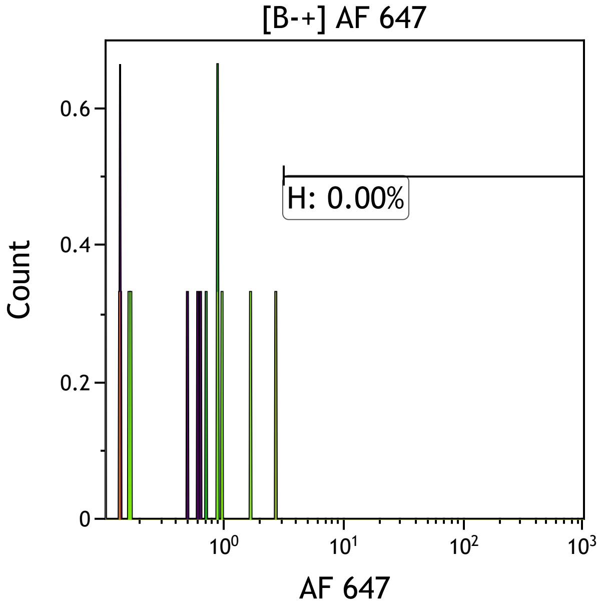

Gate	X-Med
All	0.16
H	N/A

3 contra-2017-03-21-115724-13
Rat microglia M1M2 protocol - Imported

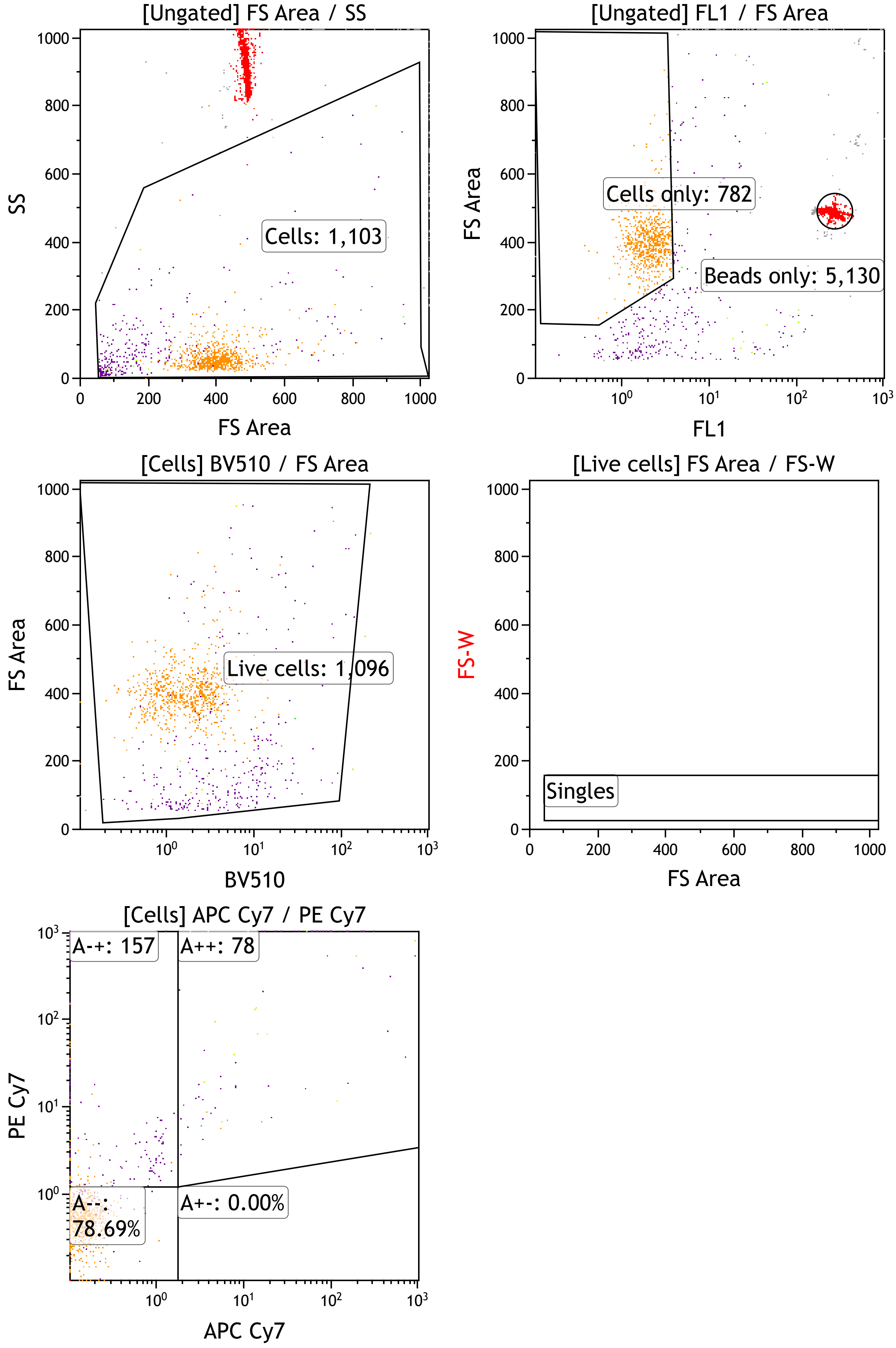

3 contra-2017-03-21-115724-13
Rat microglia M1M2 protocol - Imported

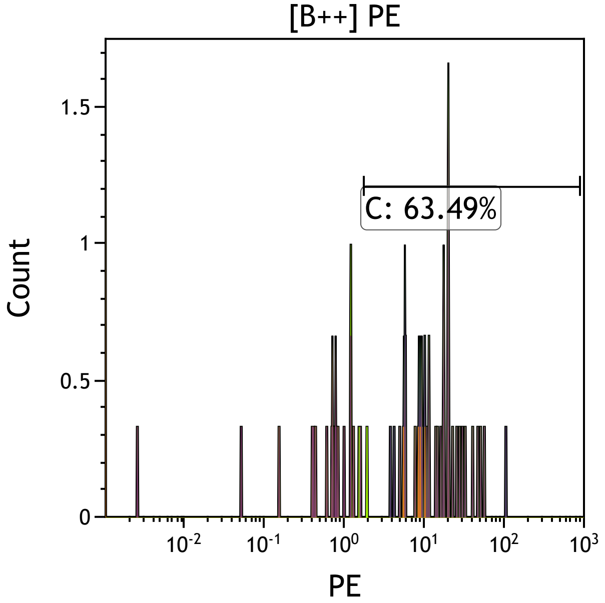

Gate	X-Med
All	7.80
C	14.81

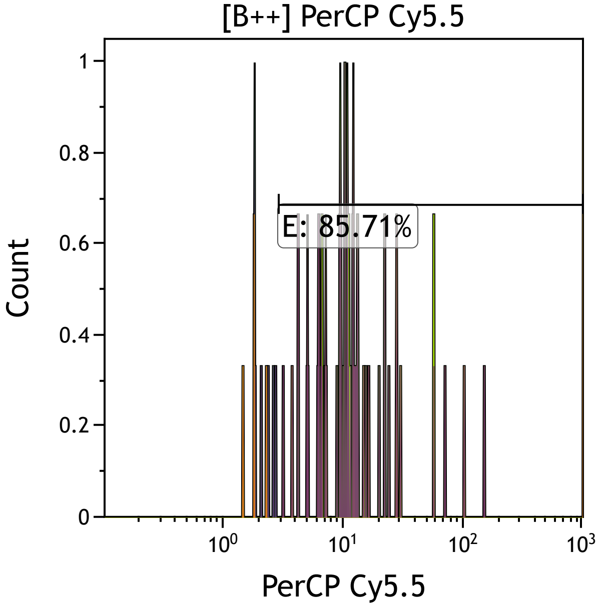

Gate	X-Med
All	10.50
E	11.30

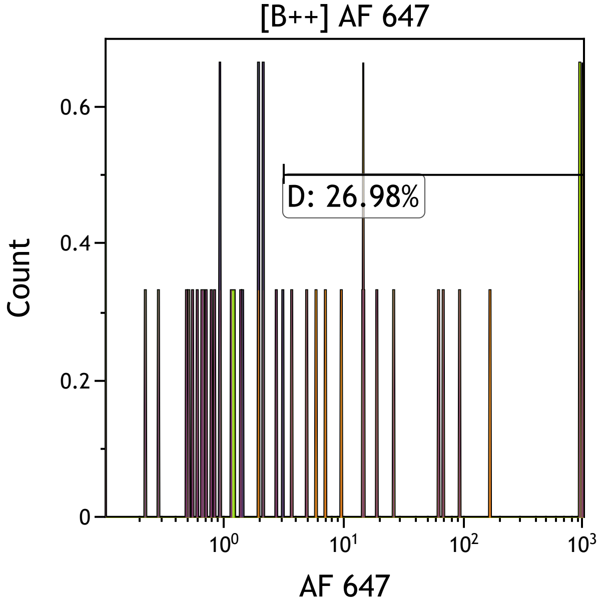

Gate	X-Med
All	0.79
D	26.21

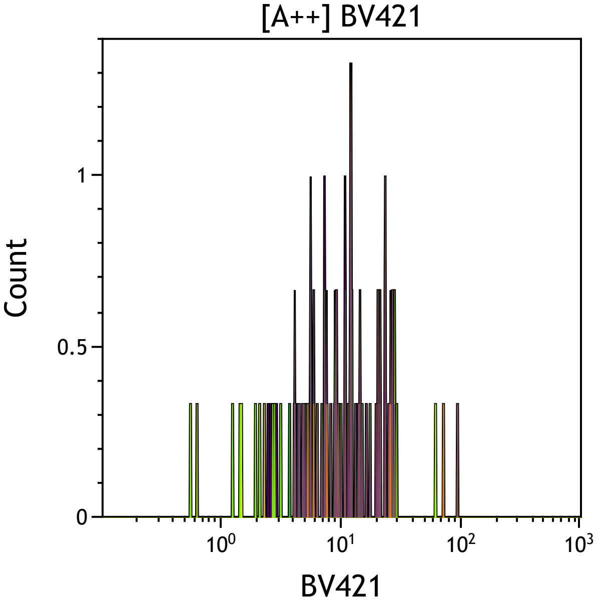

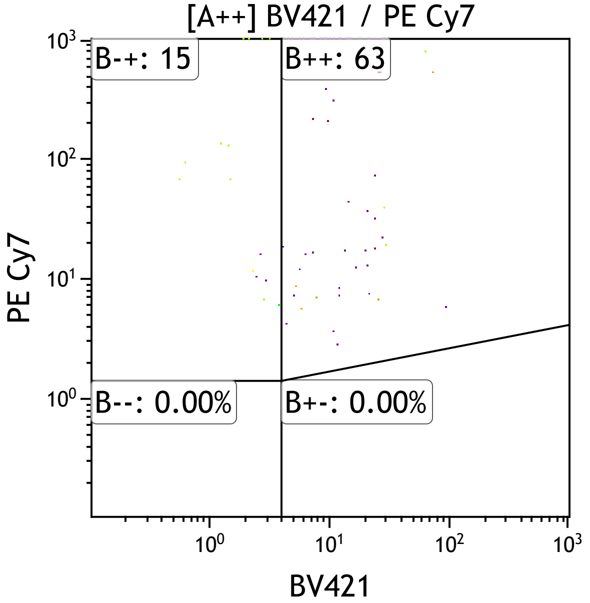

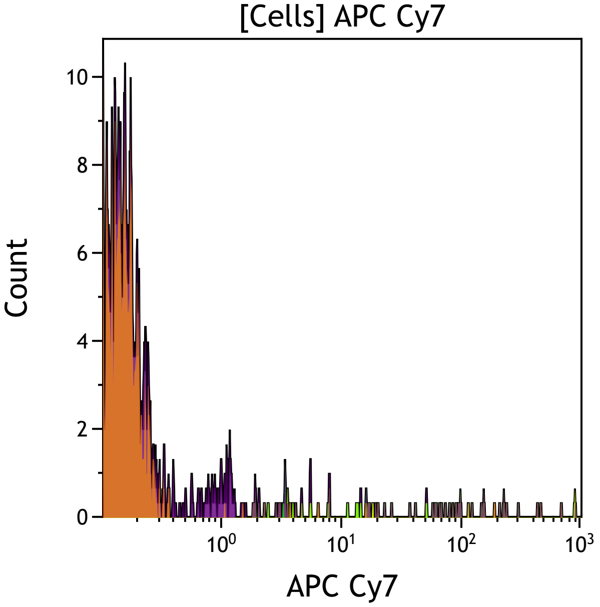

Gate	X-Med	Y-Med
All	9.28	206.53
B--	N/A	N/A
B-+	2.31	68.01
B+-	N/A	N/A
B++	11.99	375.62

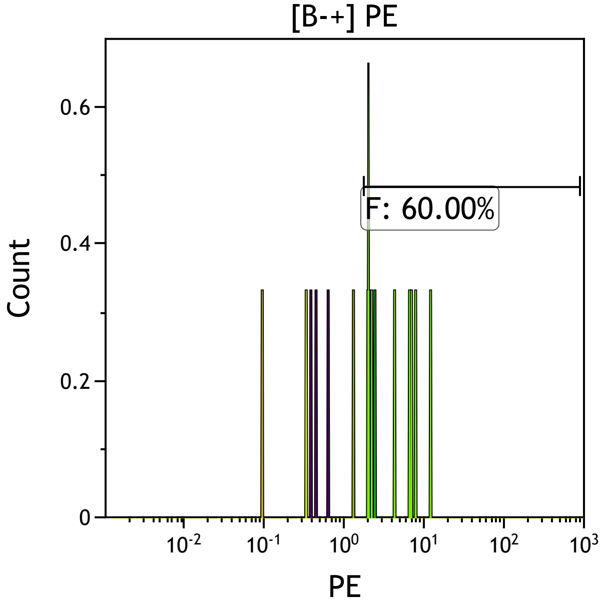

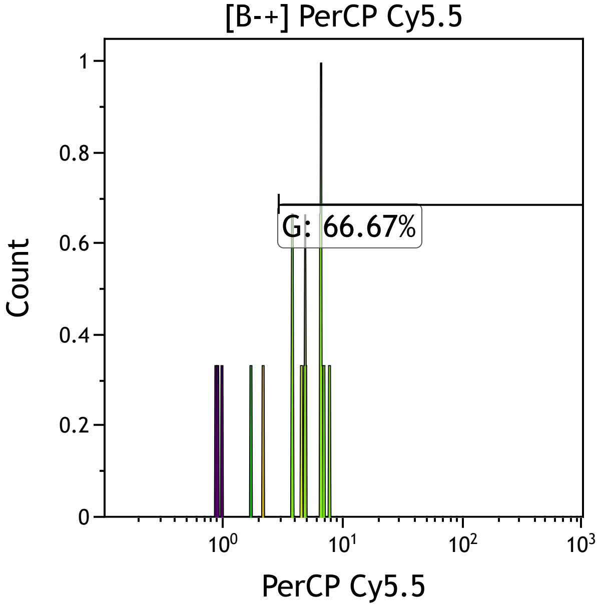

Gate	X-Med
All	2.05
F	4.31

Gate	X-Med
All	4.54
G	6.53

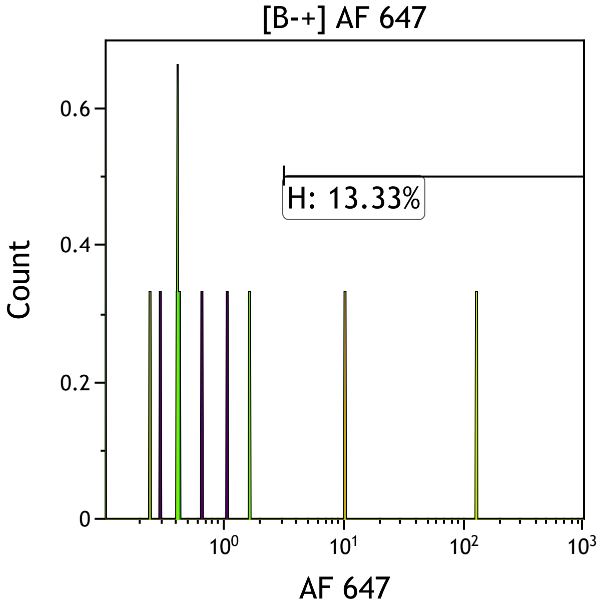

Gate	X-Med
All	0.41
H	69.30

4 contra-2017-03-21-115957-14
Rat microglia M1M2 protocol - Imported

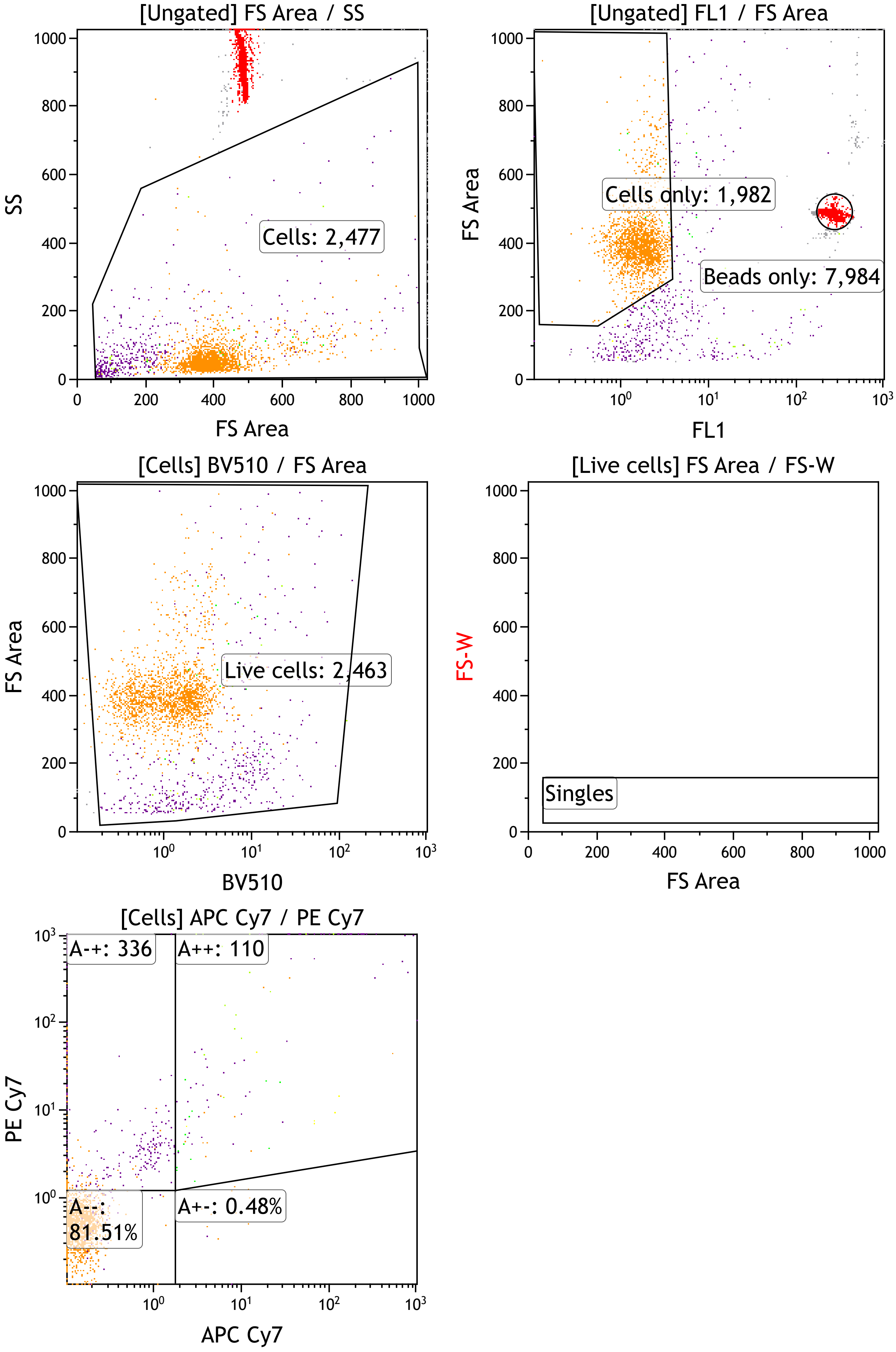

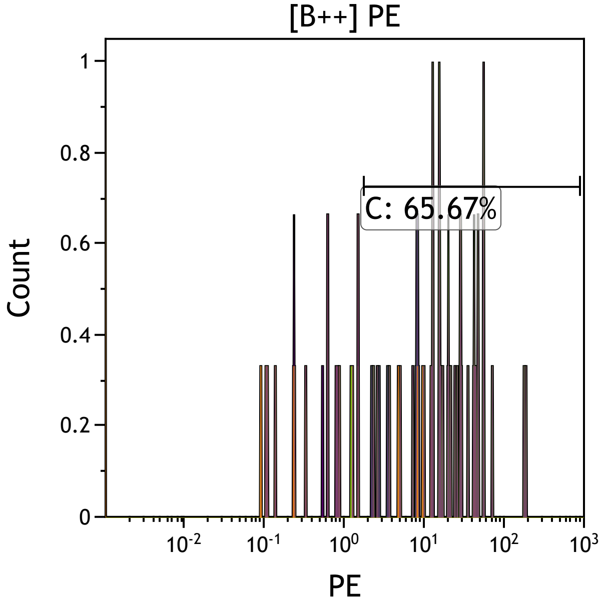

Gate	X-Med
All	8.23
C	16.27

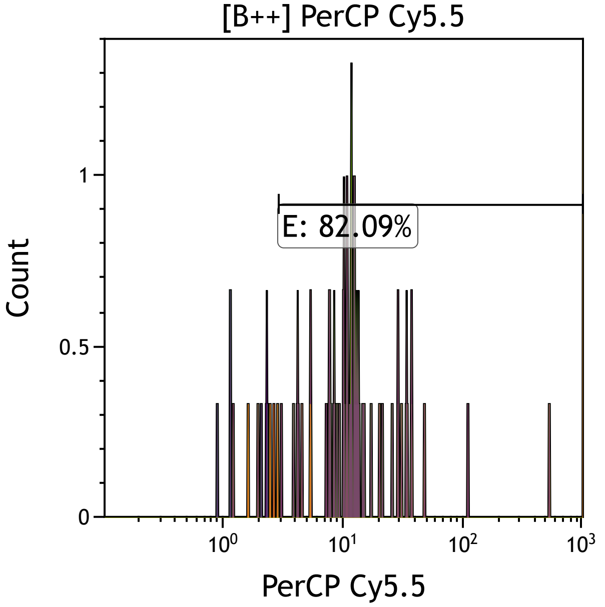

Gate	X-Med
All	11.05
E	12.42

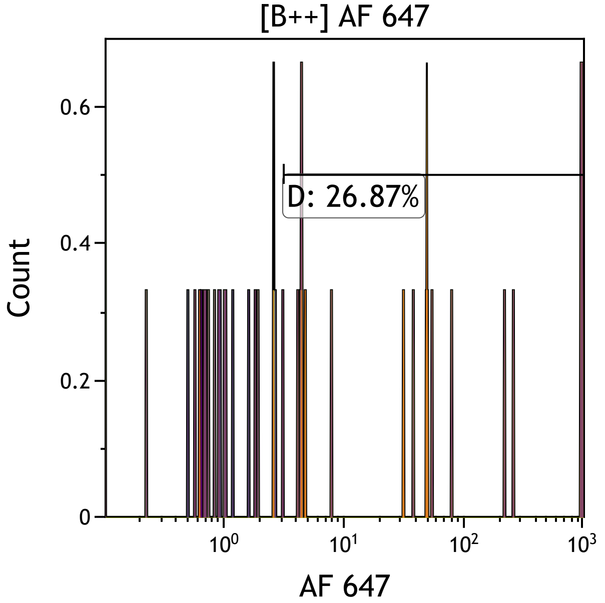

Gate	X-Med
All	0.69
D	49.87

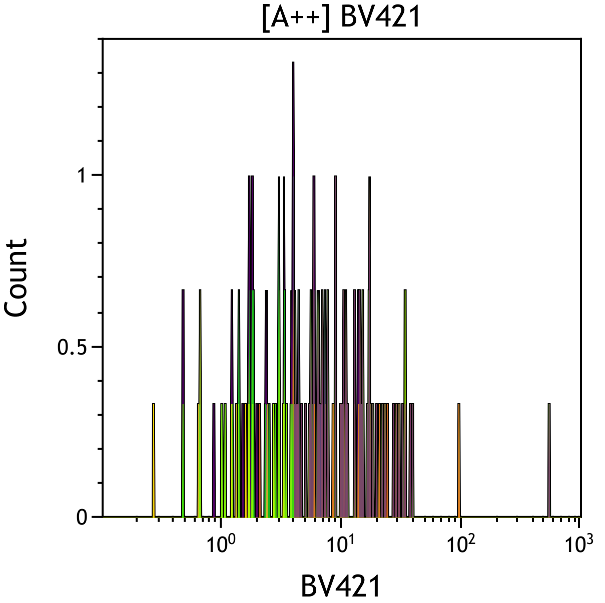

4 contra-2017-03-21-115957-14
Rat microglia M1M2 protocol - Imported

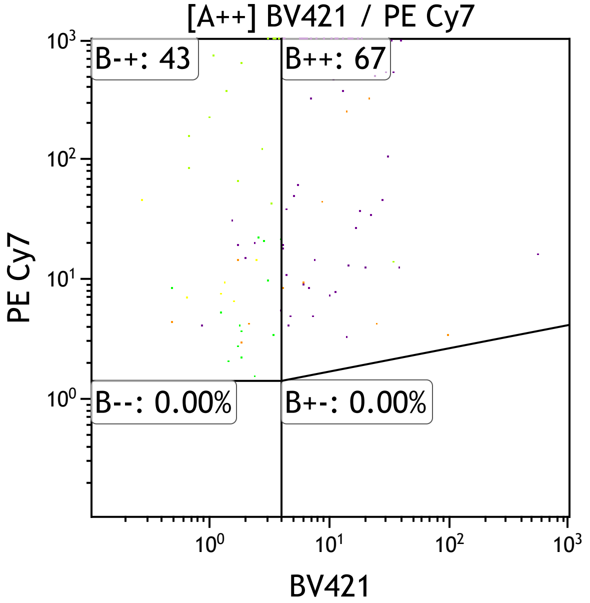

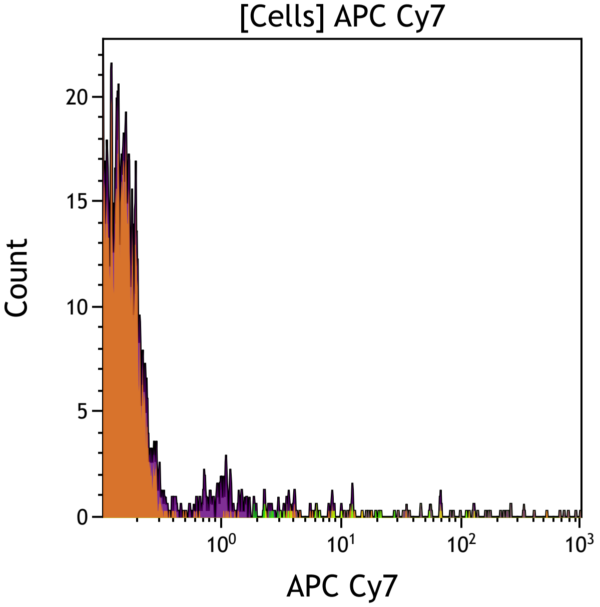

Gate	X-Med	Y-Med
All	5.66	45.17
B--	N/A	N/A
B-+	1.83	15.01
B+-	N/A	N/A
B++	10.57	362.35

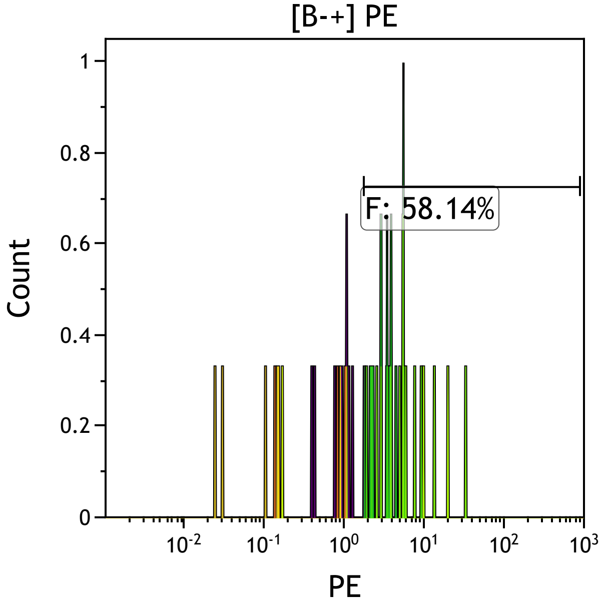

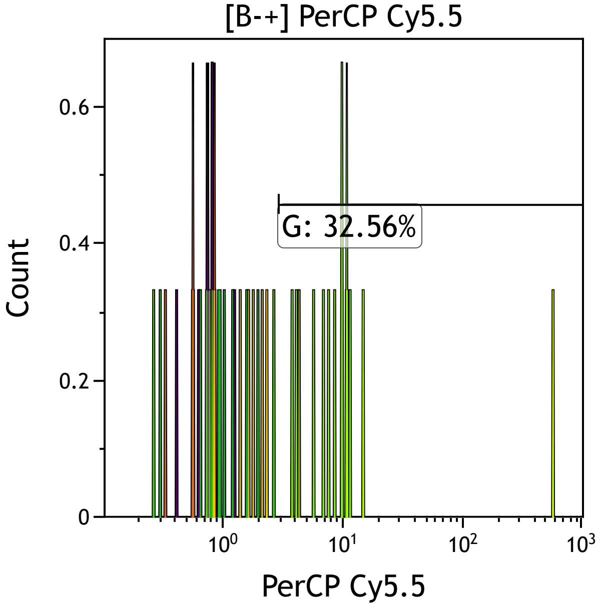

Gate	X-Med
All	2.22
F	3.92

Gate	X-Med
All	1.40
G	9.79

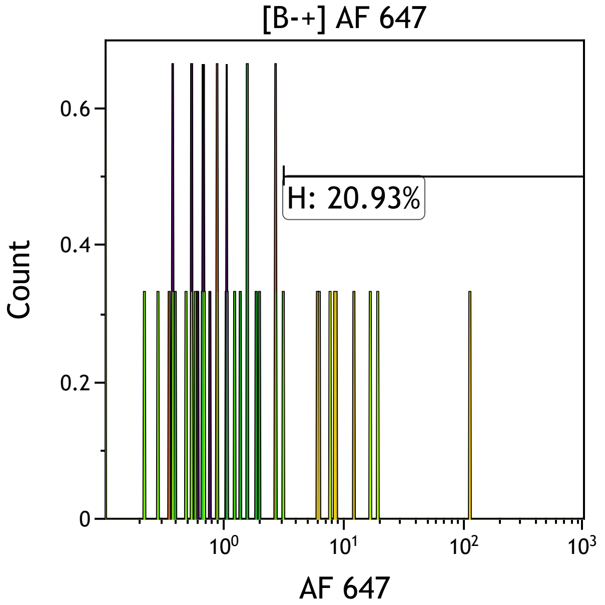

Gate	X-Med
All	0.87
H	8.67

5 contra-2017-03-21-120229-15

Rat microglia M1M2 protocol - Imported

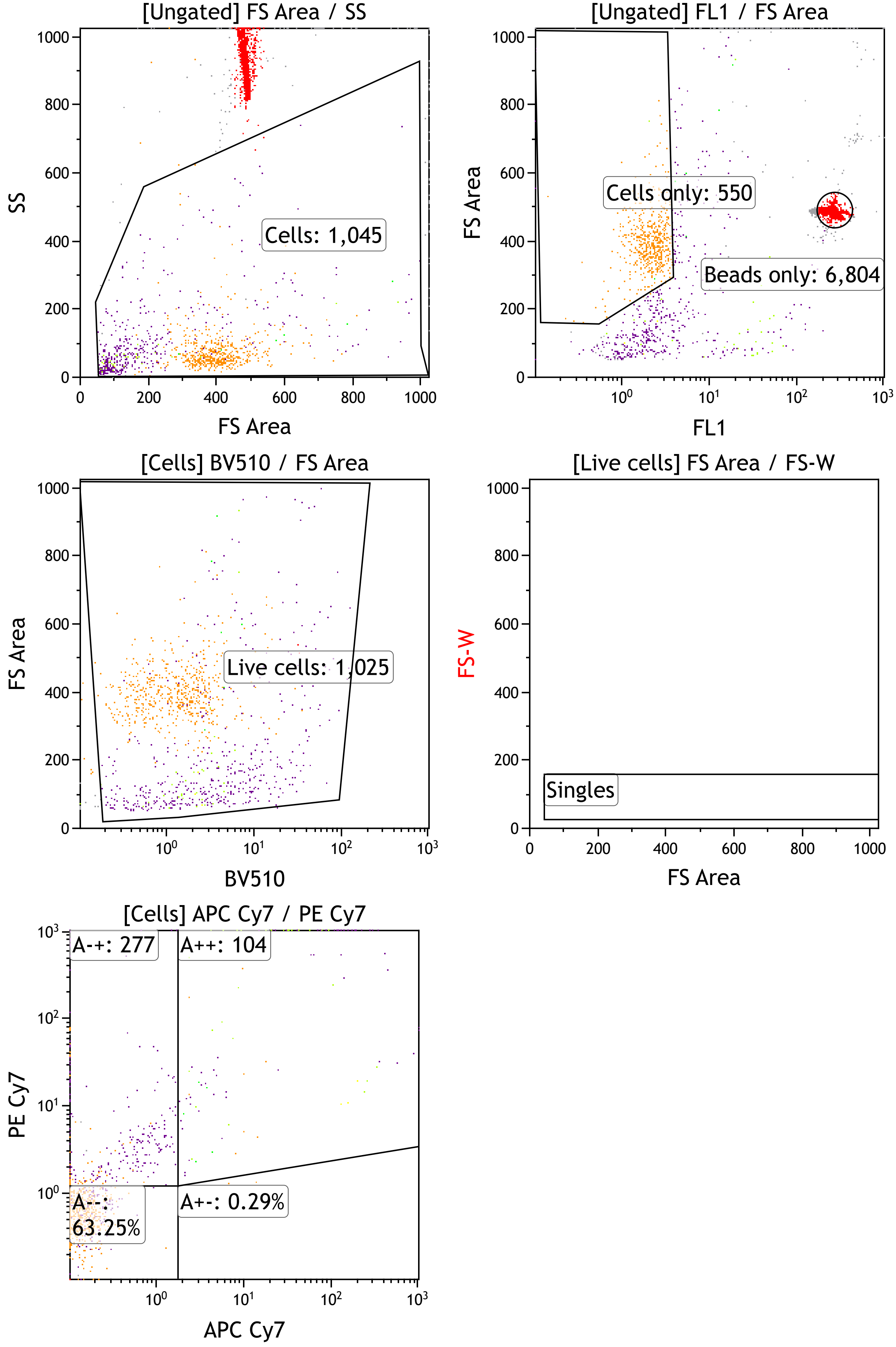

5 contra-2017-03-21-120229-15
Rat microglia M1M2 protocol - Imported

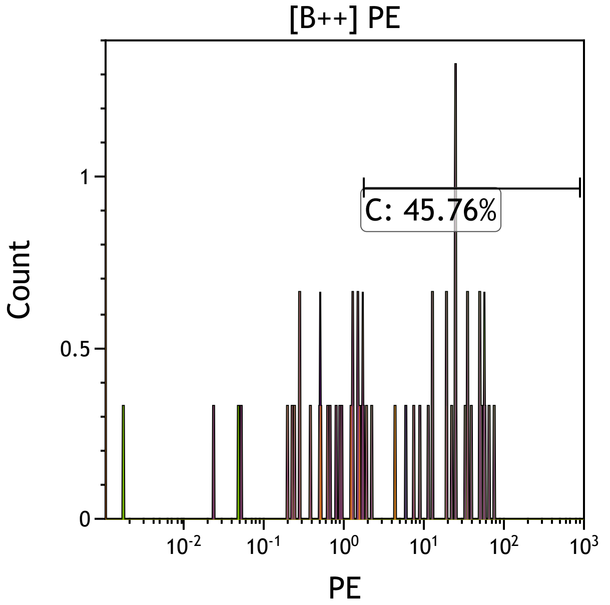

Gate	X-Med
All	1.57
C	24.89

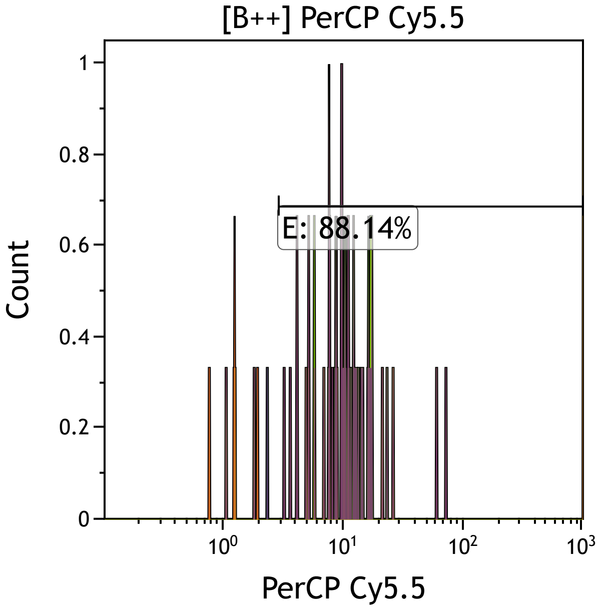

Gate	X-Med
All	10.47
E	11.30

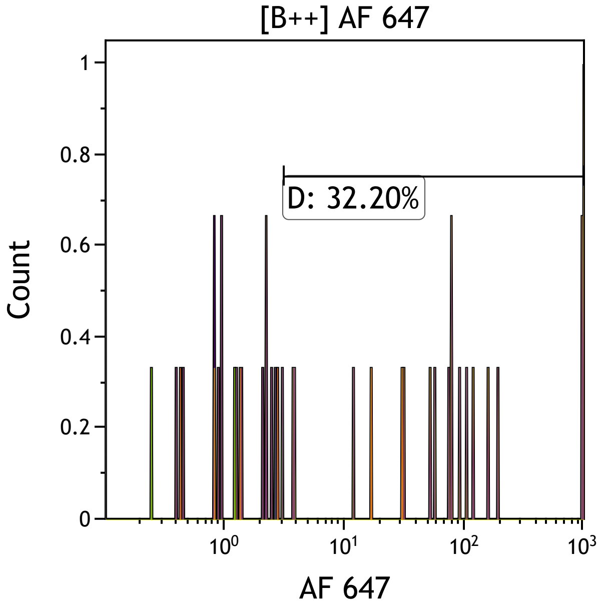

Gate	X-Med
All	1.23
D	79.24

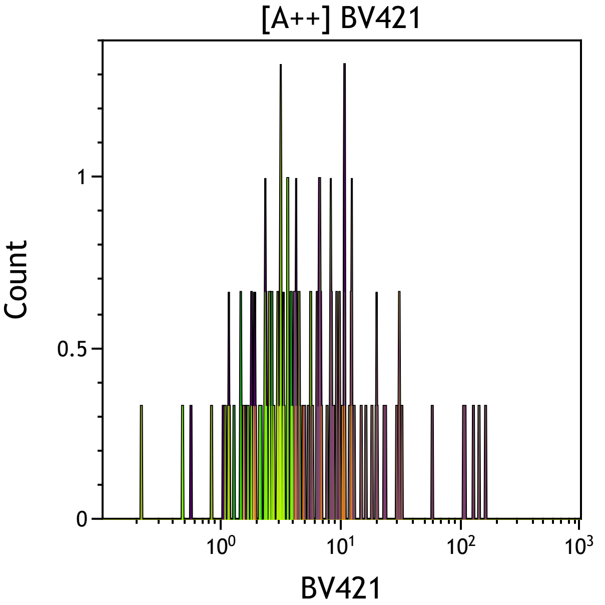

5 contra-2017-03-21-120229-15
Rat microglia M1M2 protocol - Imported

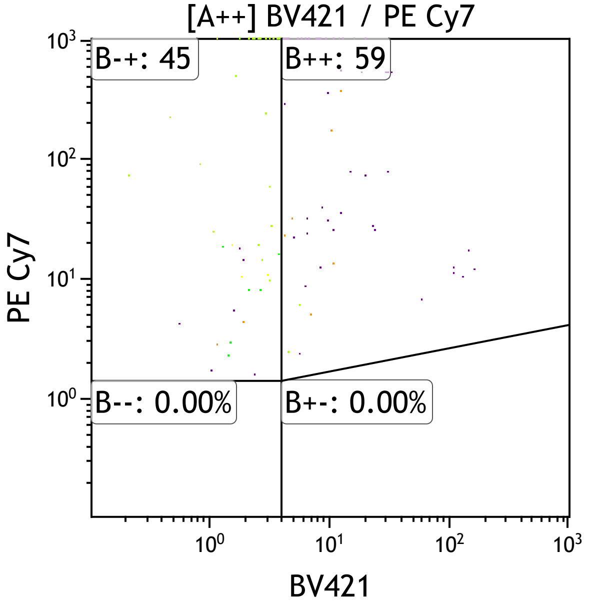

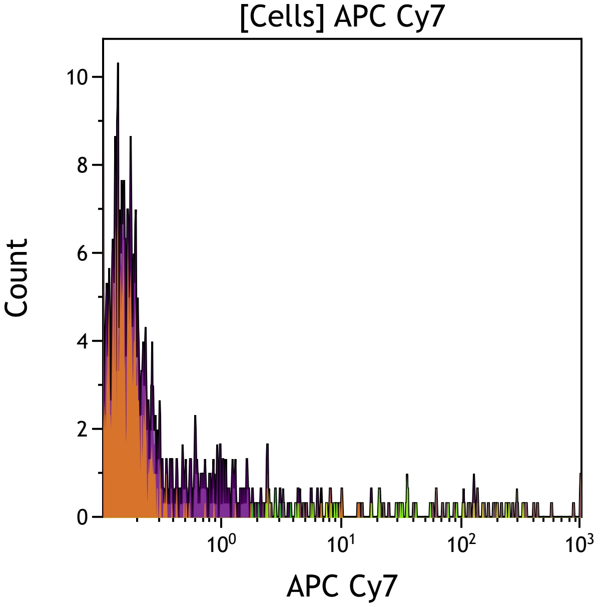

Gate	X-Med	Y-Med
All	4.56	88.67
B--	N/A	N/A
B-+	2.35	58.89
B+-	N/A	N/A
B++	9.32	281.68

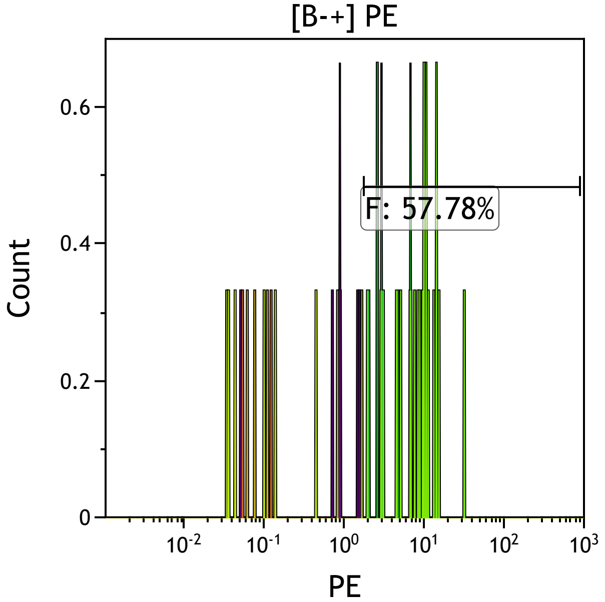

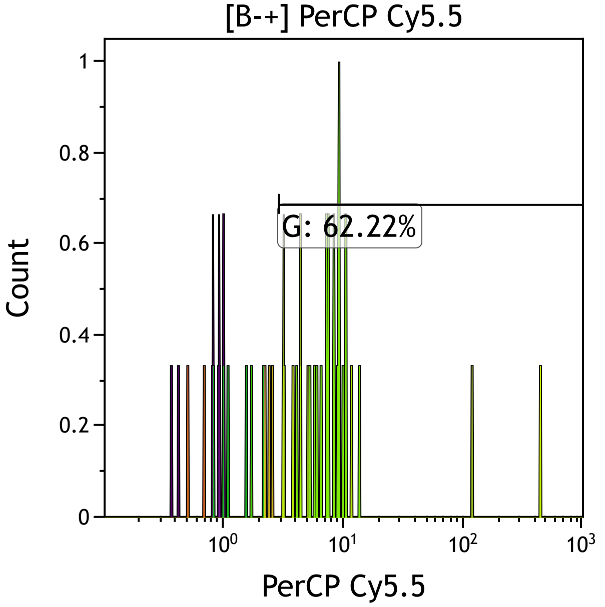

Gate	X-Med
All	2.62
F	8.40

Gate	X-Med
All	4.47
G	7.61

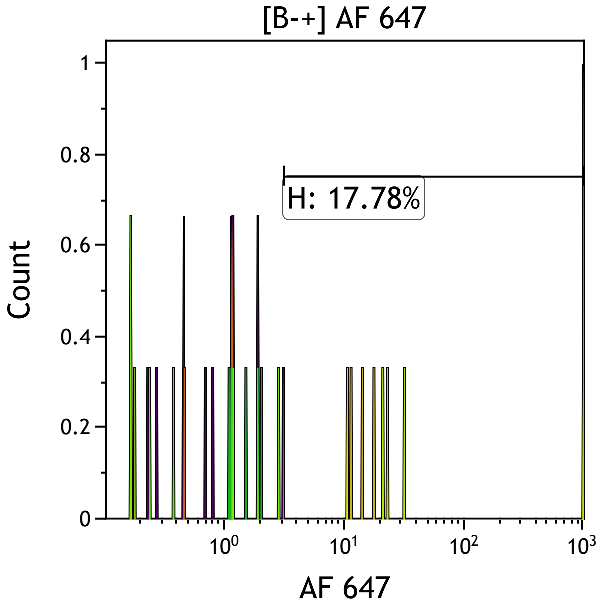

Gate	X-Med
All	0.46
H	21.22

6 contra-2017-03-21-120503-16

Rat microglia M1M2 protocol - Imported

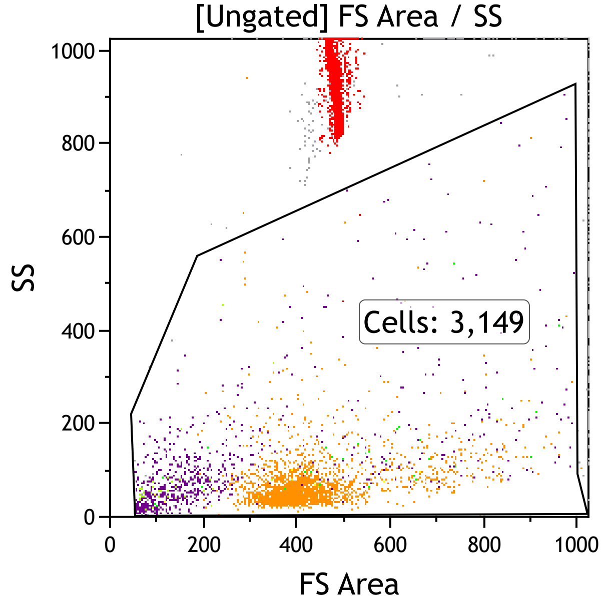

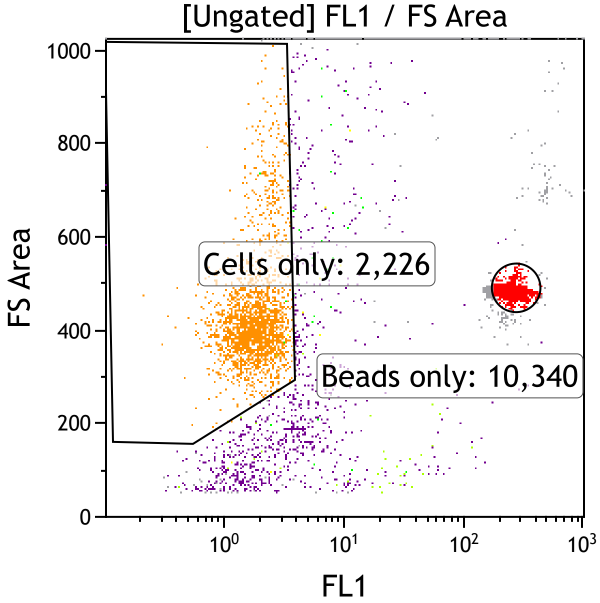

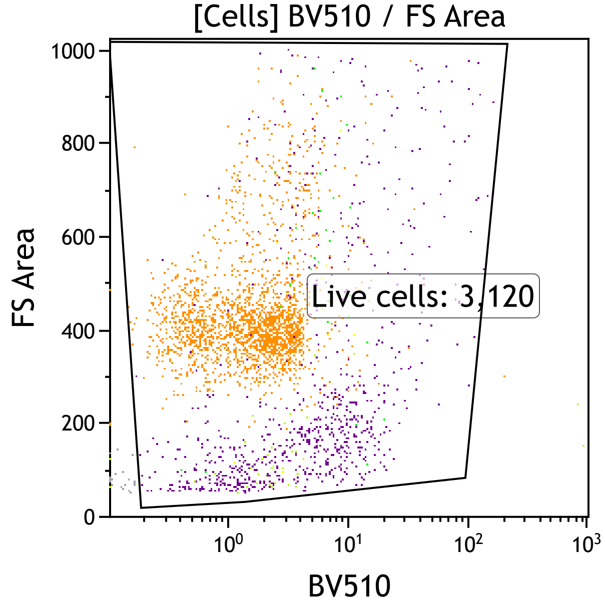

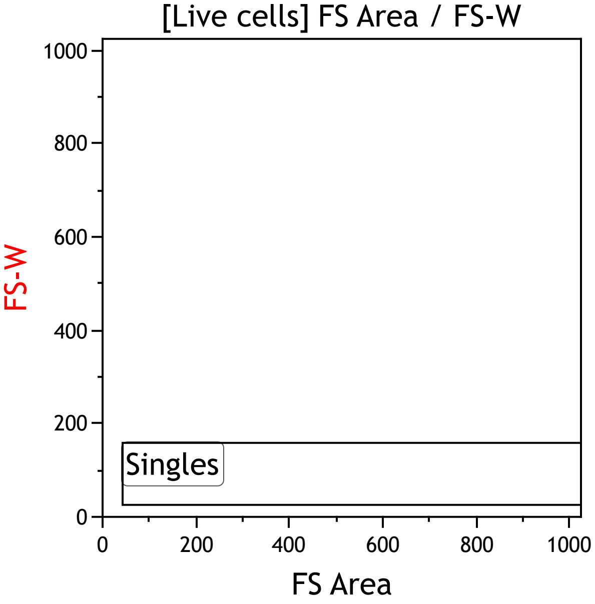

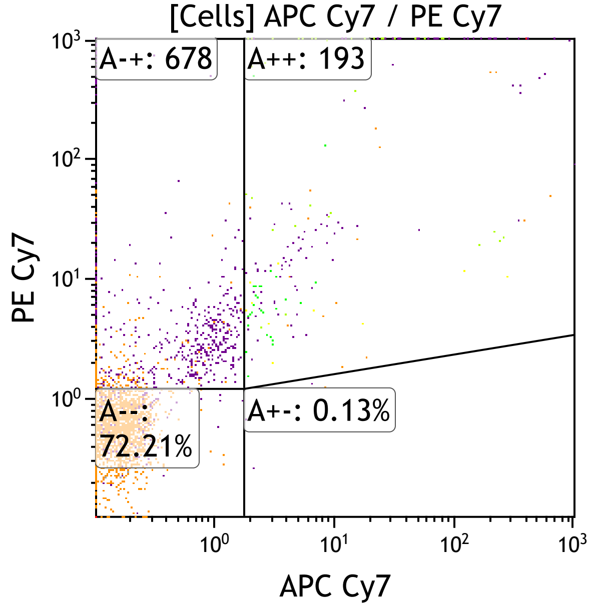

6 contra-2017-03-21-120503-16
Rat microglia M1M2 protocol - Imported

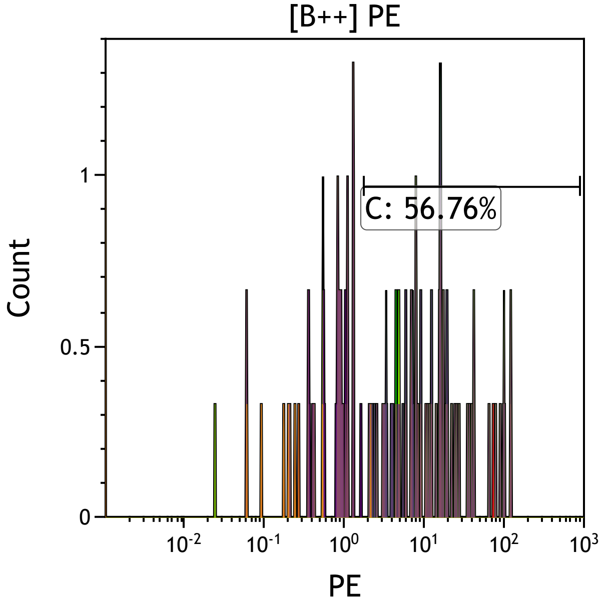

Gate	X-Med
All	3.33
C	11.85

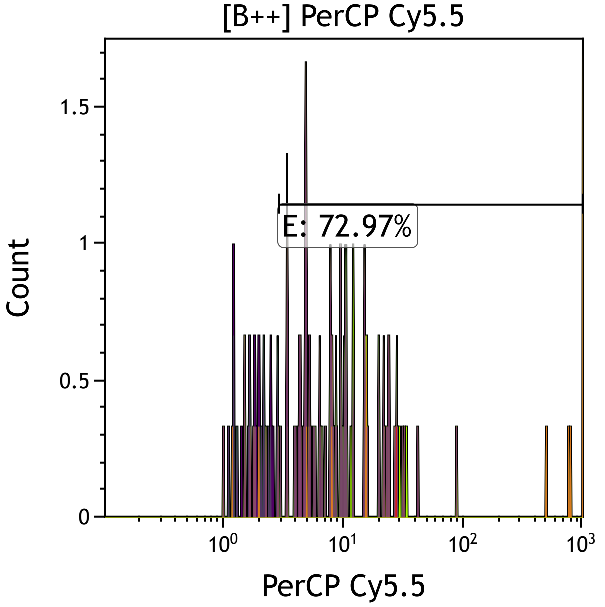

Gate	X-Med
All	6.50
E	10.29

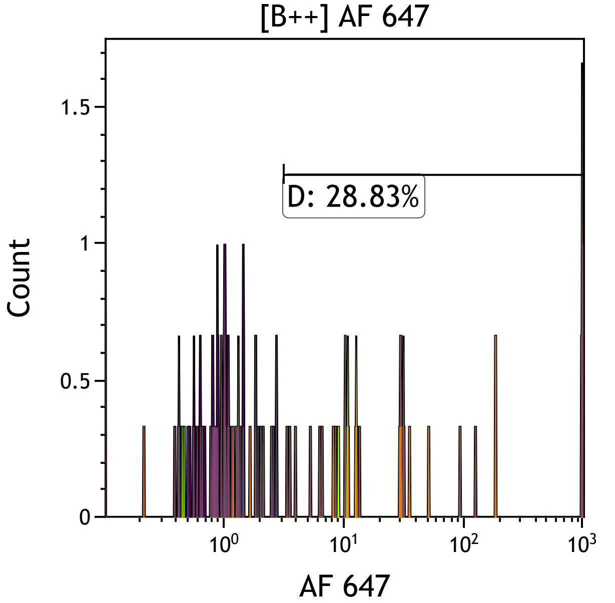

Gate	X-Med
All	1.03
D	29.33

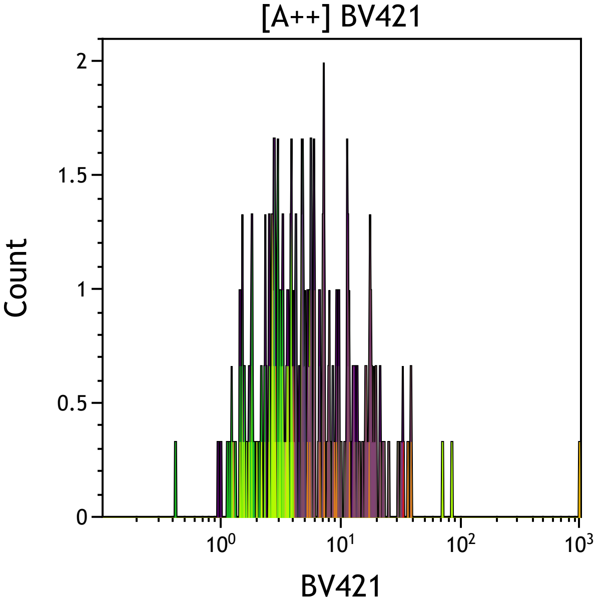

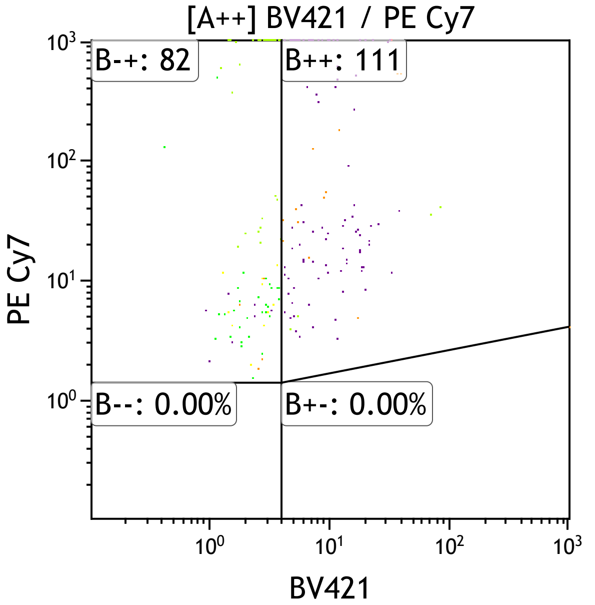

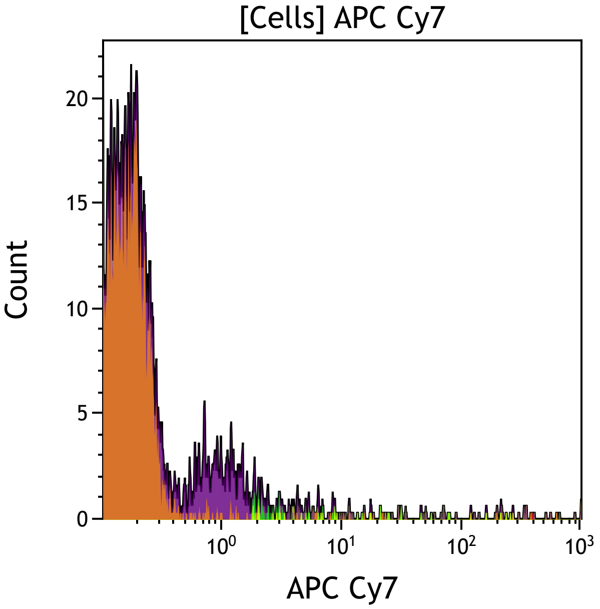

Gate	X-Med	Y-Med
All	4.83	24.61
B--	N/A	N/A
B-+	2.66	9.49
B+-	N/A	N/A
B++	8.91	30.89

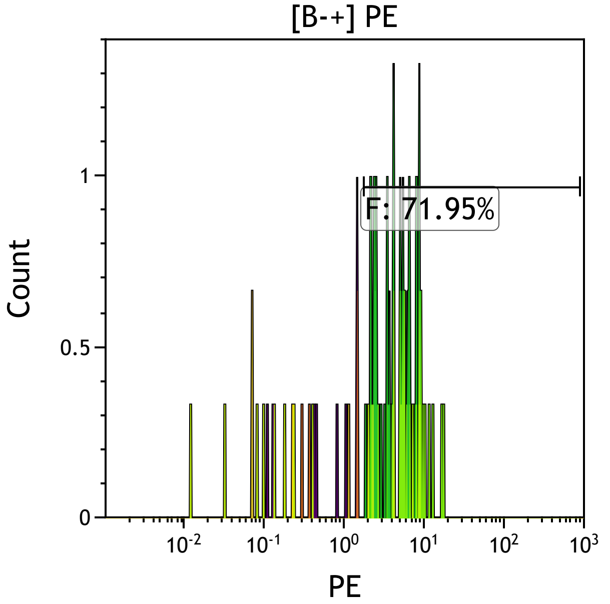

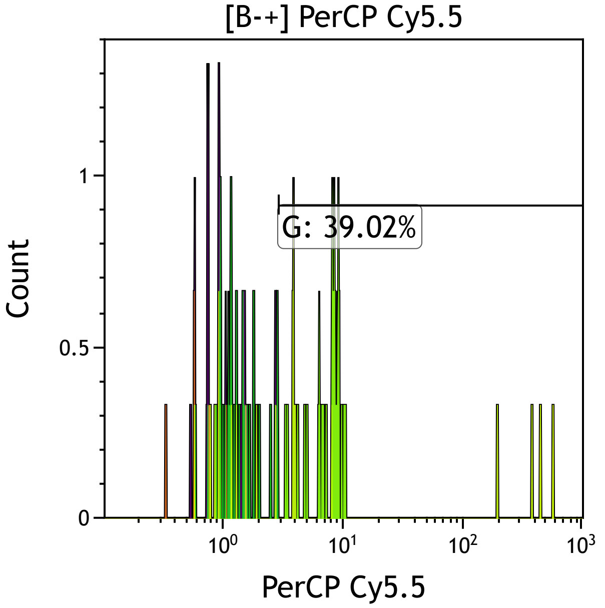

Gate	X-Med
All	3.47
F	5.13

Gate	X-Med
All	1.80
G	8.25

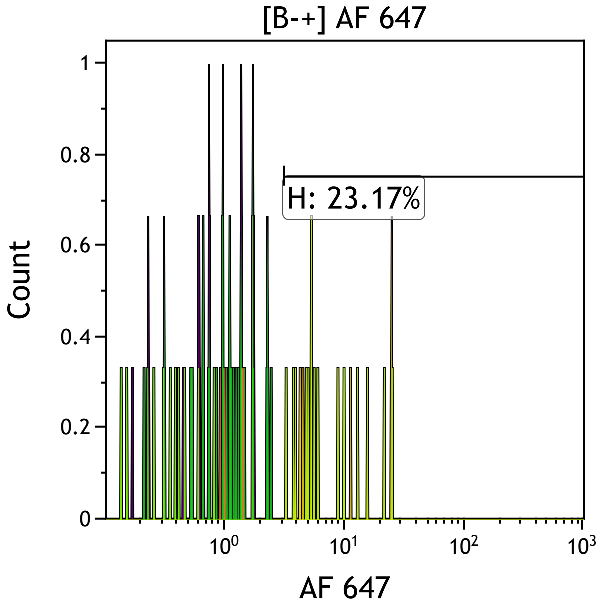

Gate	X-Med
All	0.97
H	5.68

7 contra-2017-03-21-120735-17

Rat microglia M1M2 protocol - Imported

[Ungated] FS Area / SS

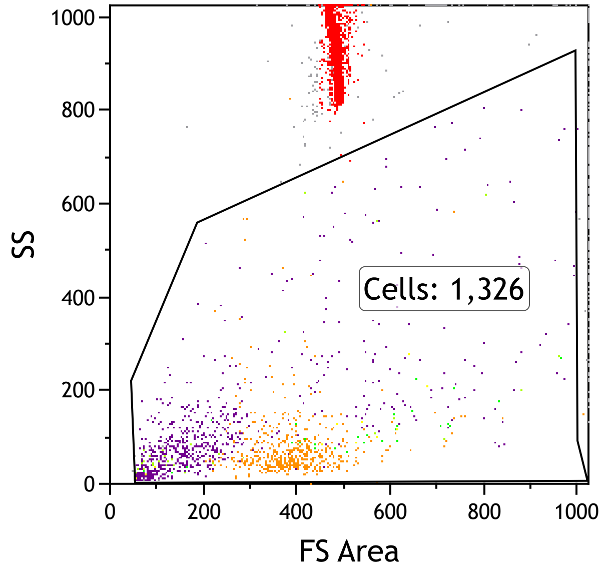

[Ungated] FL1 / FS Area

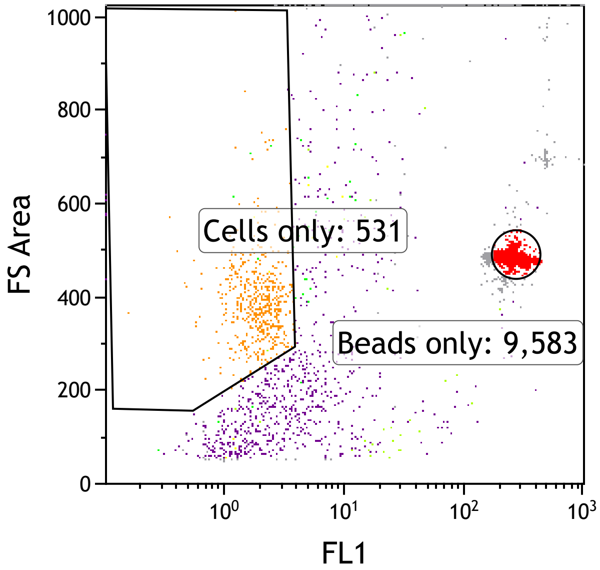

[Cells] BV510 / FS Area

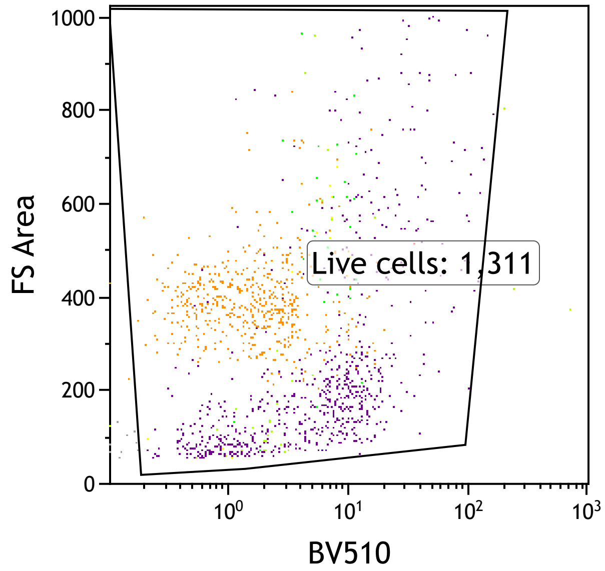

[Live cells] FS Area / FS-W

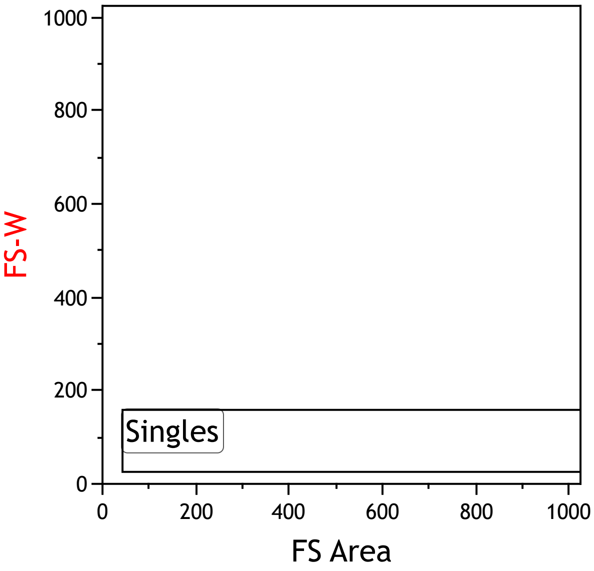

[Cells] APC Cy7 / PE Cy7

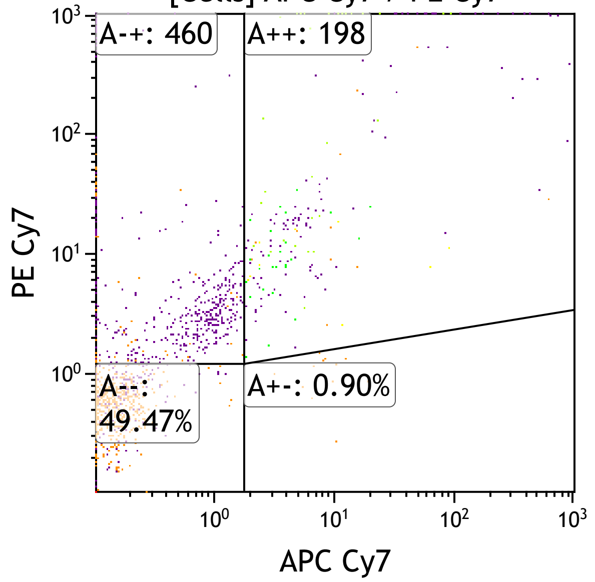

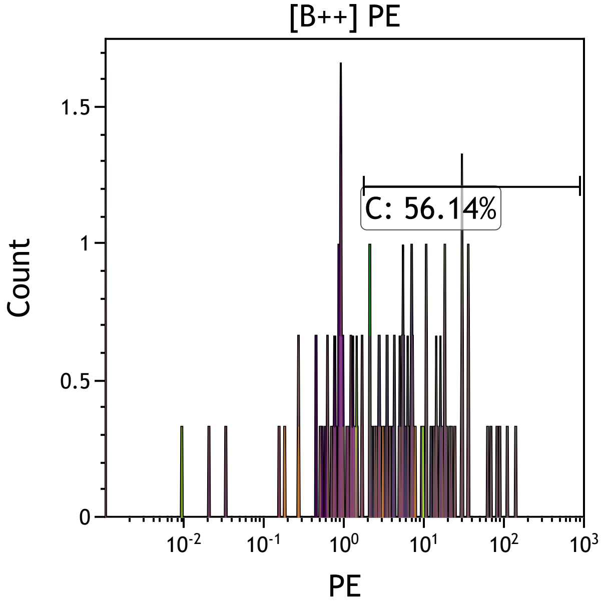

Gate	X-Med
All	2.74
C	9.36

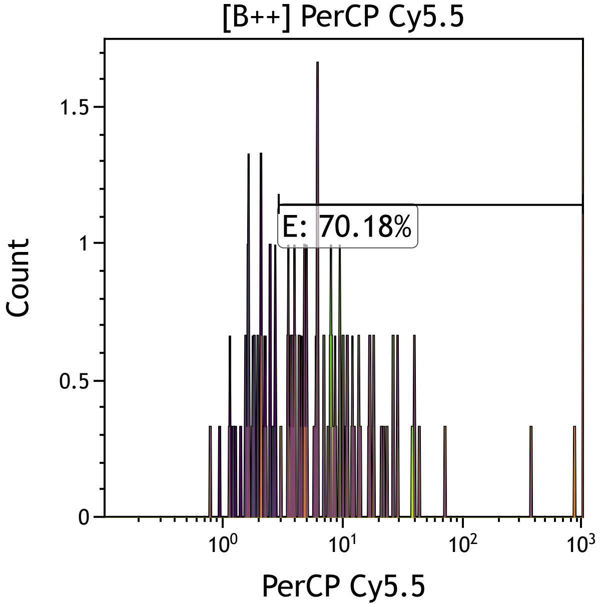

Gate	X-Med
All	6.02
E	9.44

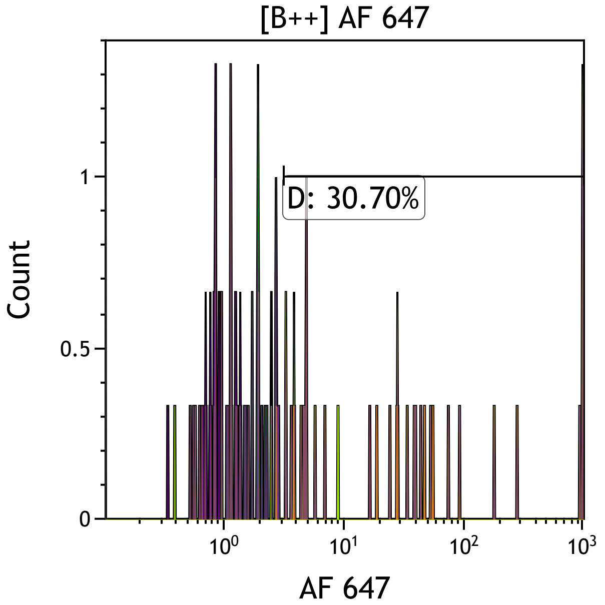

Gate	X-Med
All	1.35
D	27.92

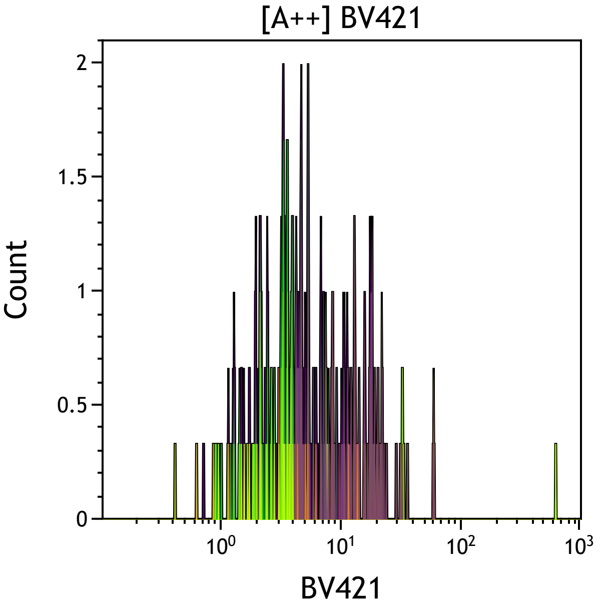

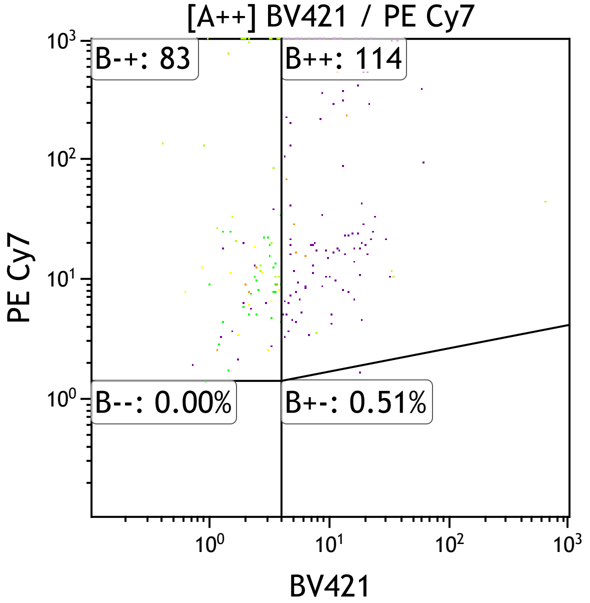

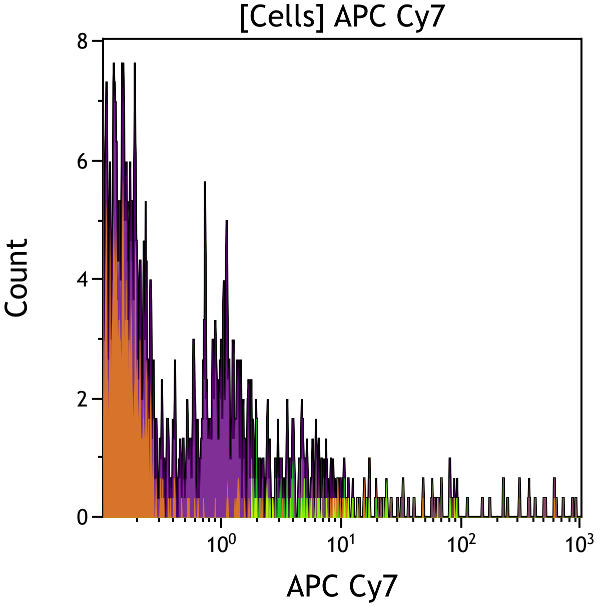

Gate	X-Med	Y-Med
All	4.70	17.67
B--	N/A	N/A
B-+	2.42	11.67
B+-	17.98	1.69
B++	9.28	21.60

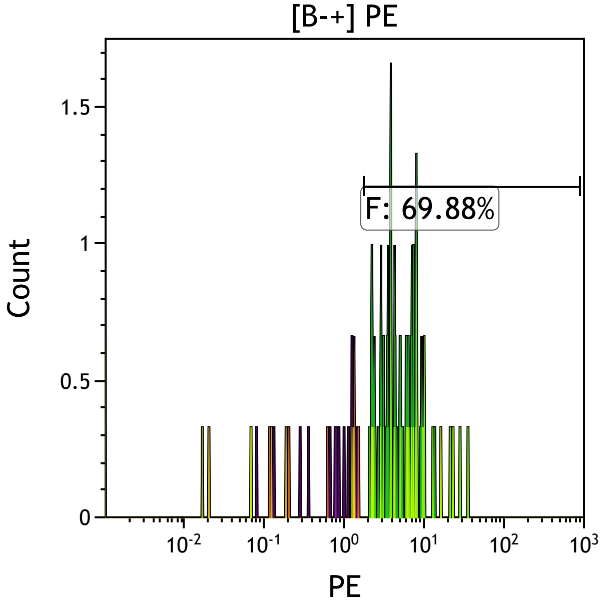

Gate	X-Med
All	3.62
F	5.38

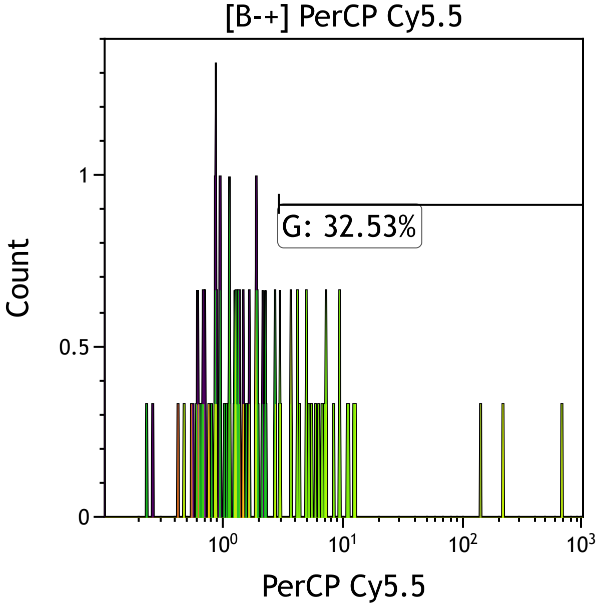

Gate	X-Med
All	1.49
G	6.74

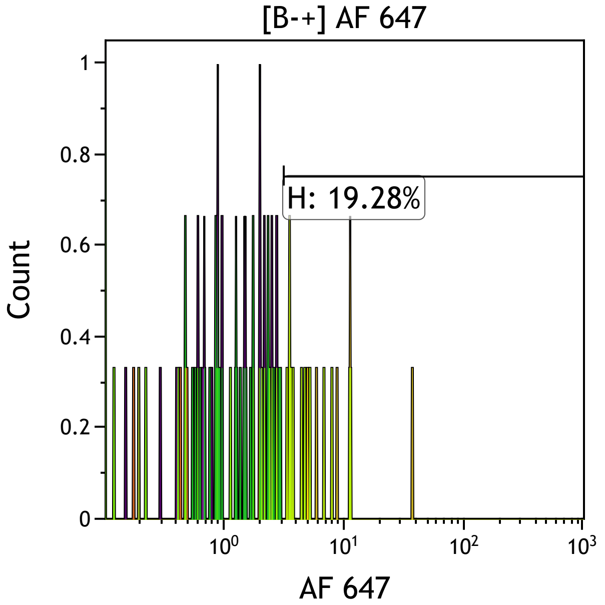

Gate	X-Med
All	1.14
H	5.22

8 contra-2017-03-21-121008-18

Rat microglia M1M2 protocol - Imported

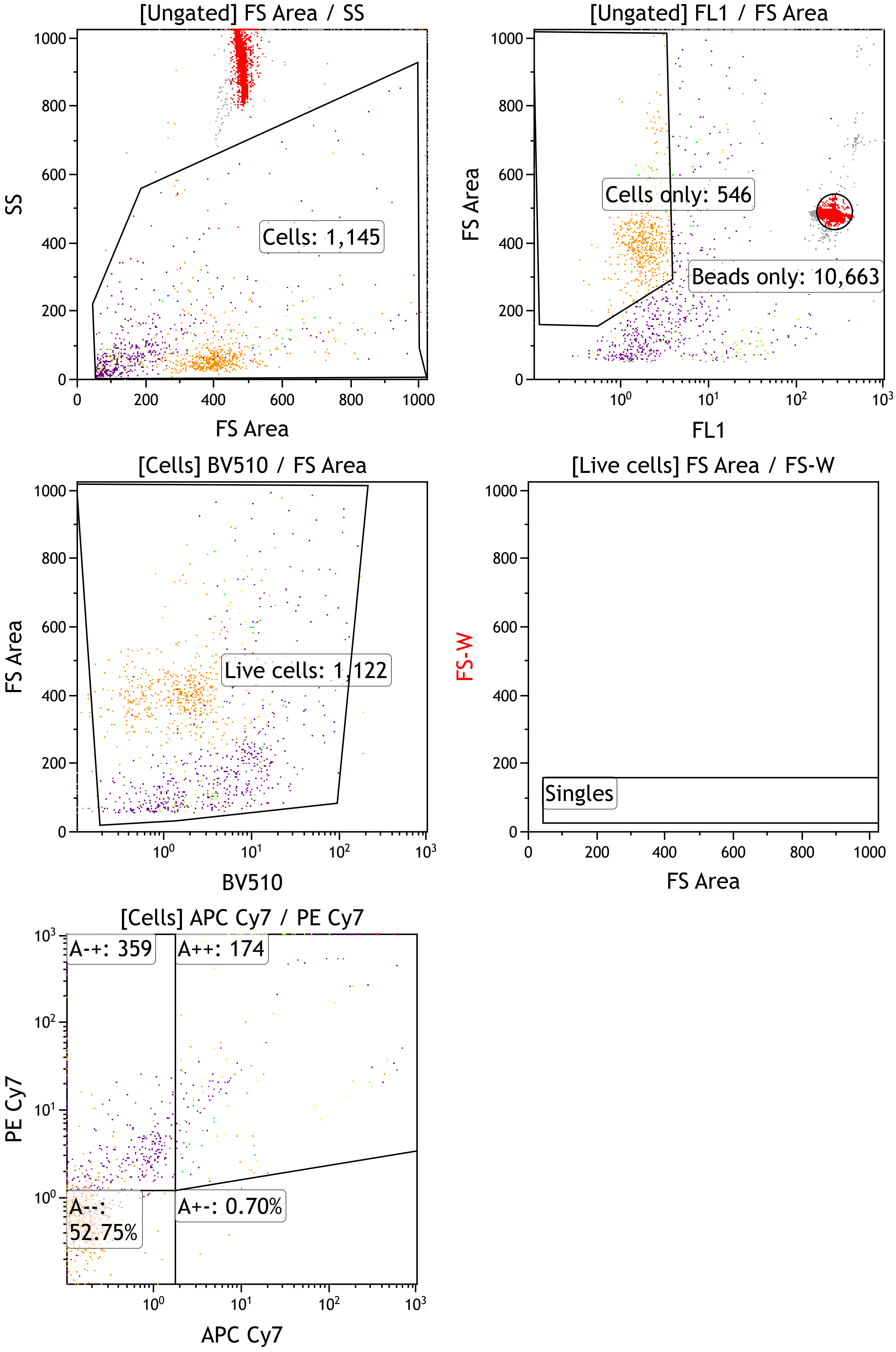

8 contra-2017-03-21-121008-18
Rat microglia M1M2 protocol - Imported

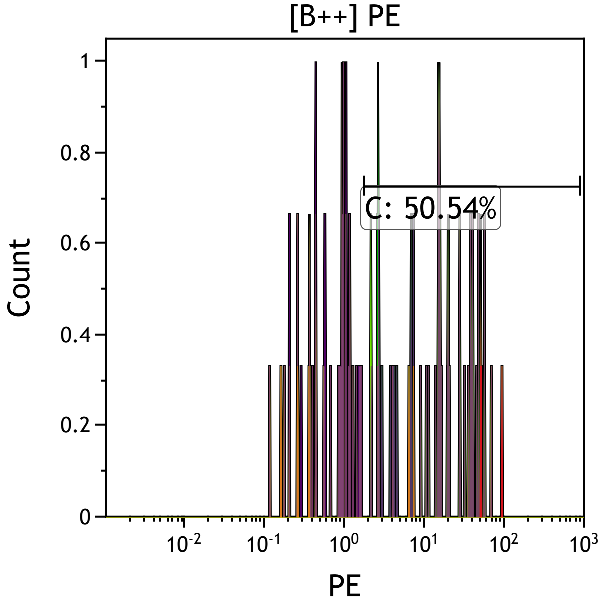

Gate	X-Med
All	2.17
C	15.89

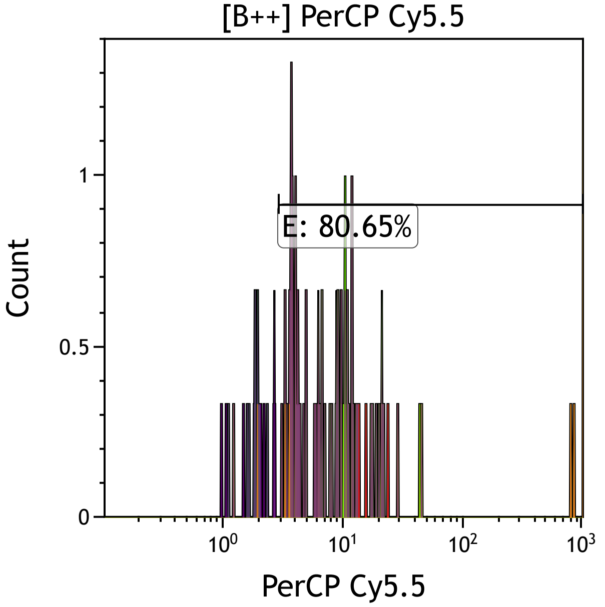

Gate	X-Med
All	6.72
E	9.72

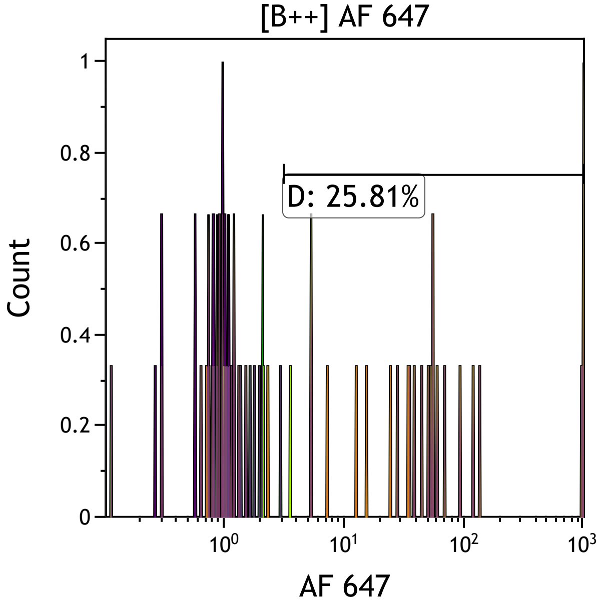

Gate	X-Med
All	0.97
D	50.77

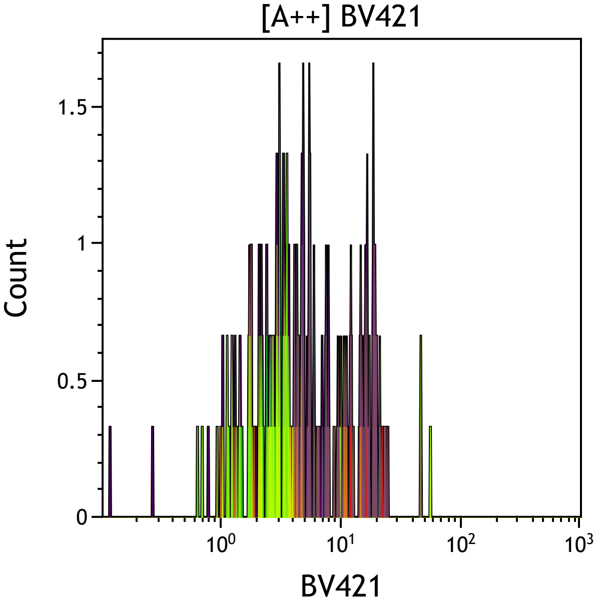

8 contra-2017-03-21-121008-18

Rat microglia M1M2 protocol - Imported

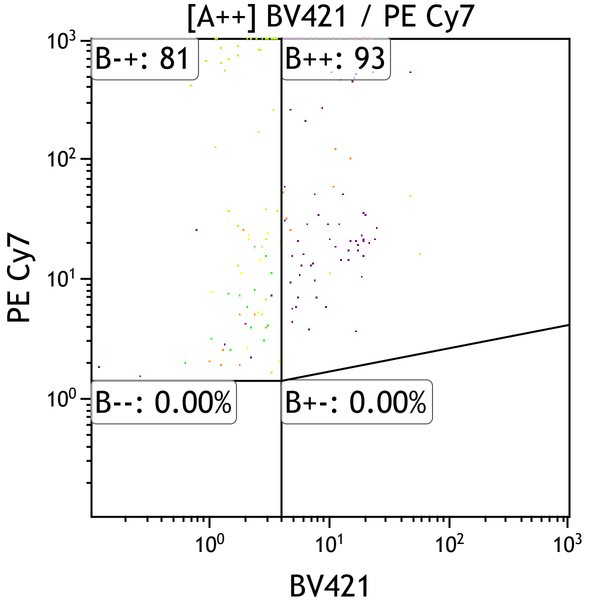

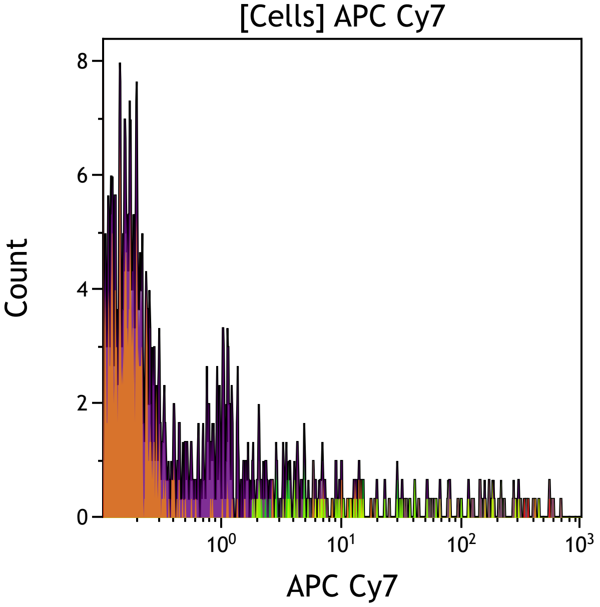

Gate	X-Med	Y-Med
All	4.34	31.80
B--	N/A	N/A
B-+	2.40	22.70
B+-	N/A	N/A
B++	9.75	49.20

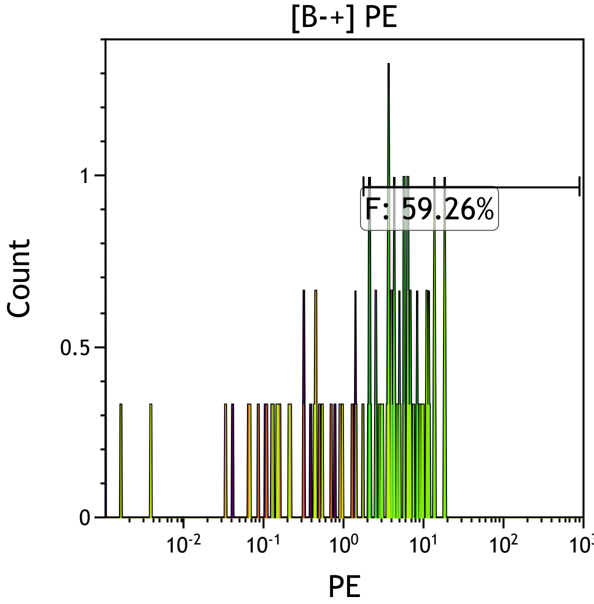

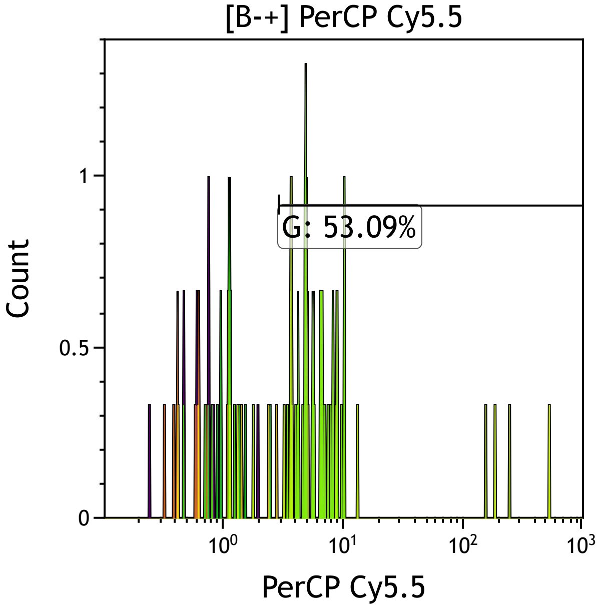

Gate	X-Med
All	2.95
F	5.71

Gate	X-Med
All	3.66
G	6.50

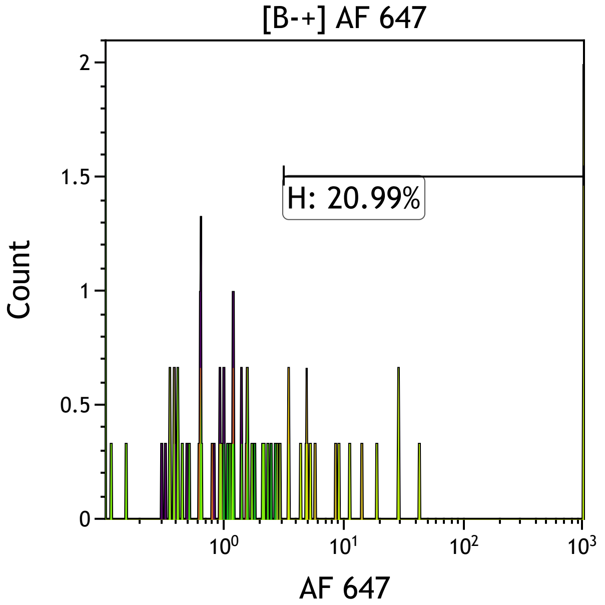

Gate	X-Med
All	0.93
H	9.15

9 contra-2017-03-21-121241-19
Rat microglia M1M2 protocol - Imported

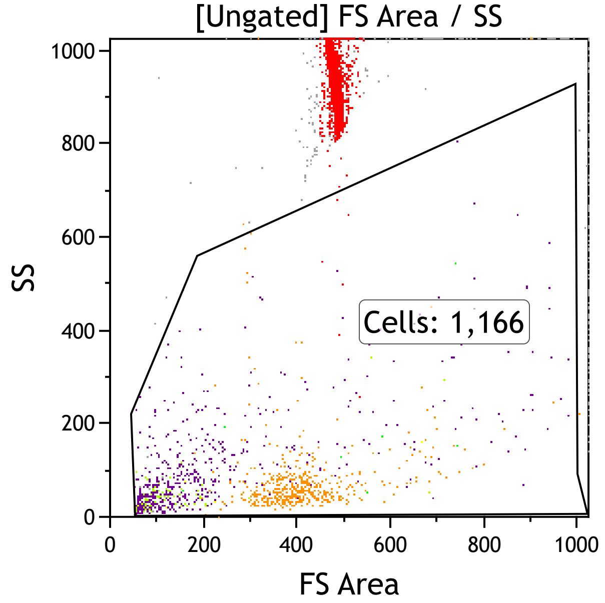

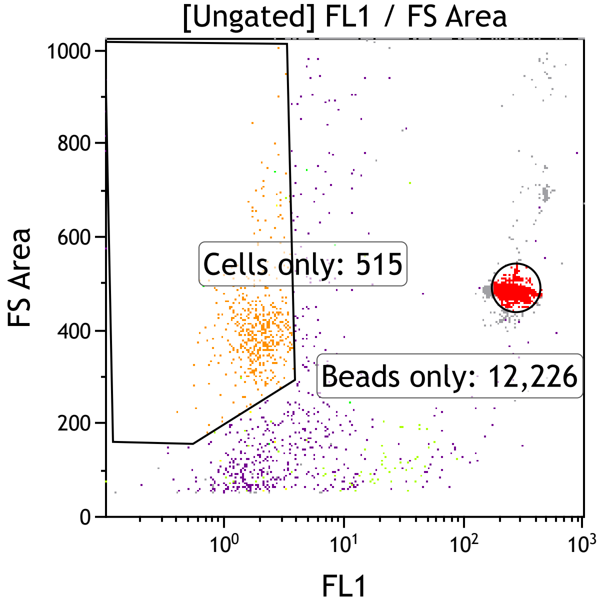

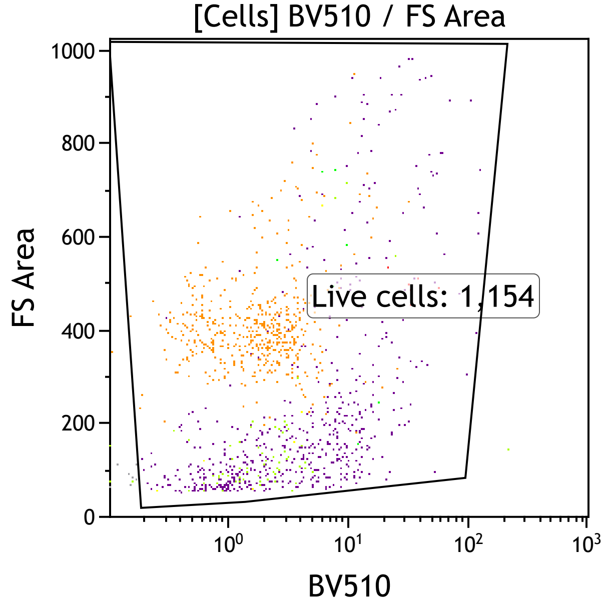

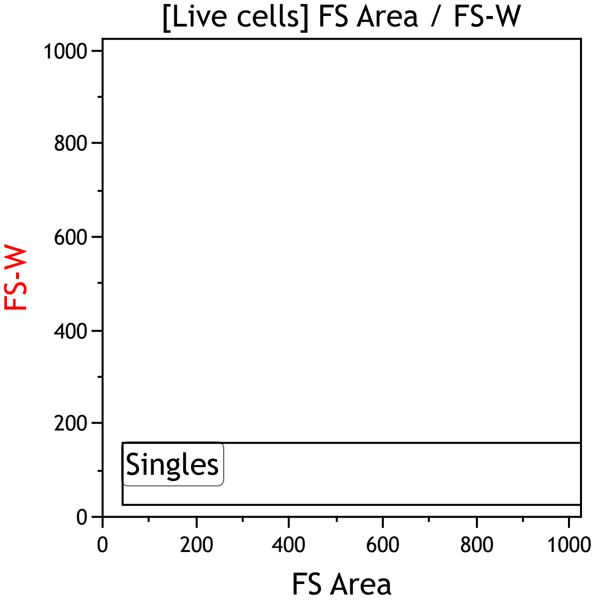

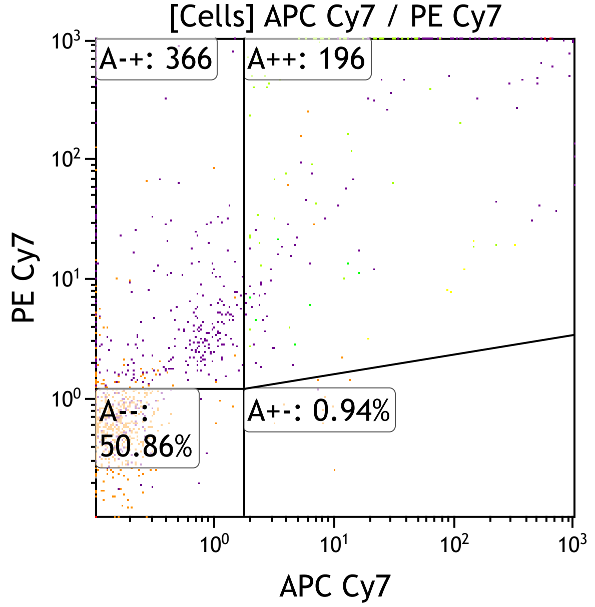

9 contra-2017-03-21-121241-19
Rat microglia M1M2 protocol - Imported

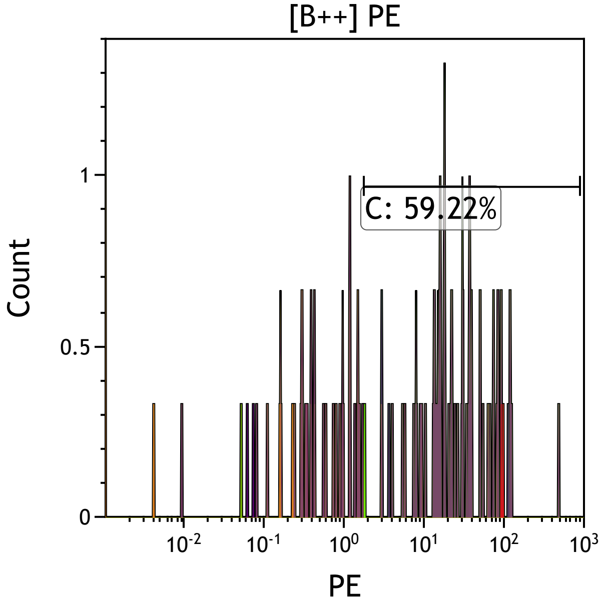

Gate	X-Med
All	8.12
C	22.65

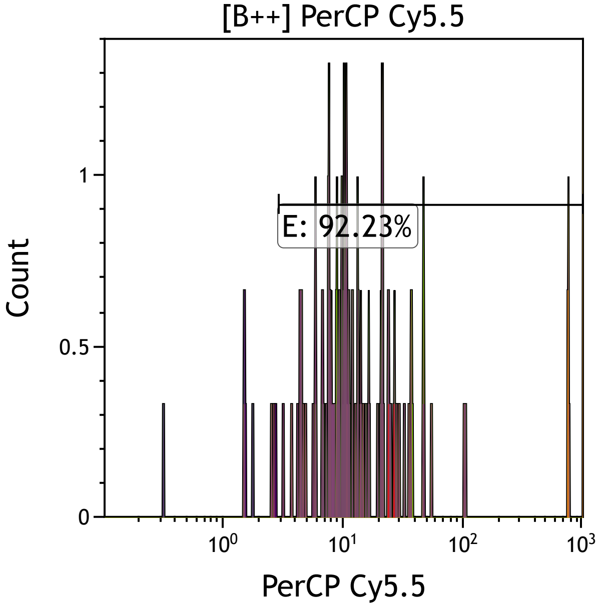

Gate	X-Med
All	10.88
E	12.07

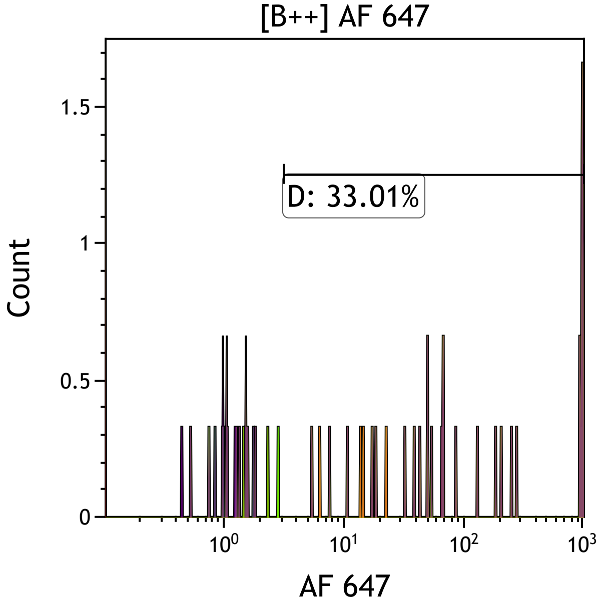

Gate	X-Med
All	0.53
D	68.01

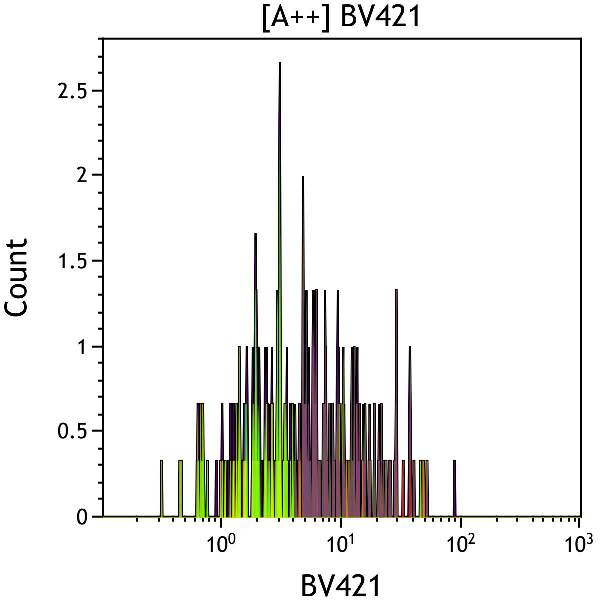

[A++] BV421 / PE Cy7

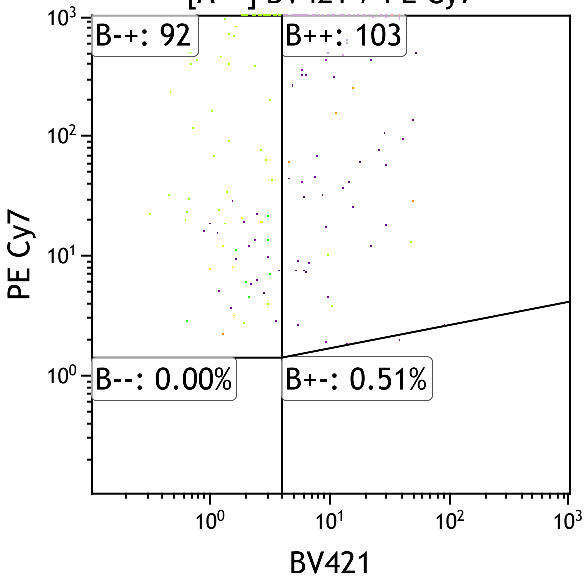

[Cells] APC Cy7

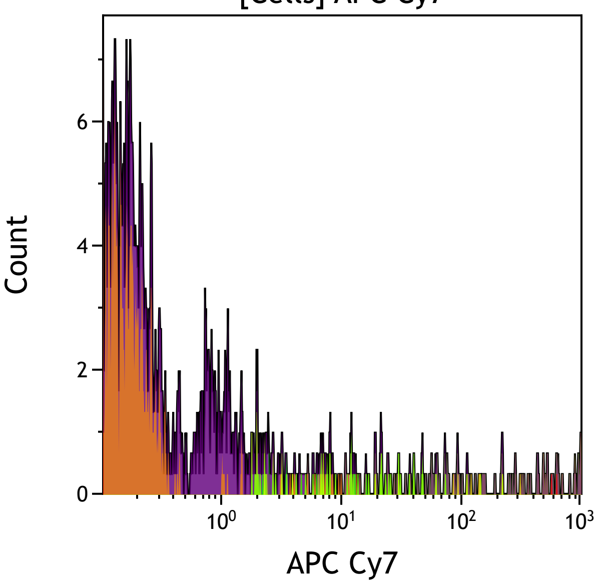

Gate	X-Med	Y-Med
All	4.83	420.32
B--	N/A	N/A
B-+	1.97	74.08
B+-	37.48	1.98
B++	9.55	757.60

[B-+] PE

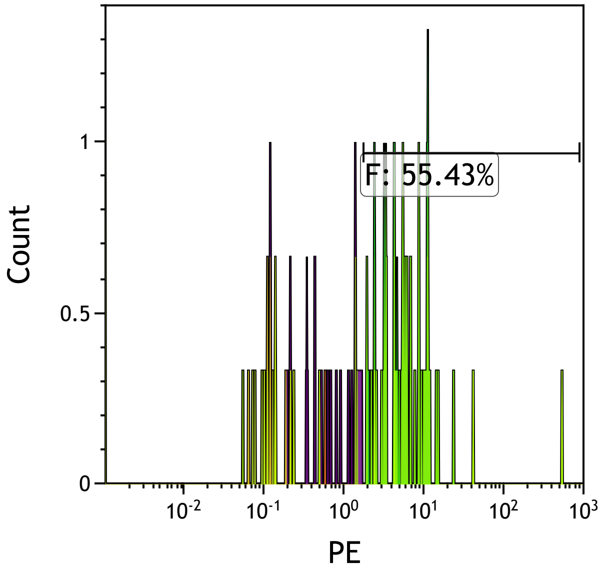

[B-+] PerCP Cy5.5

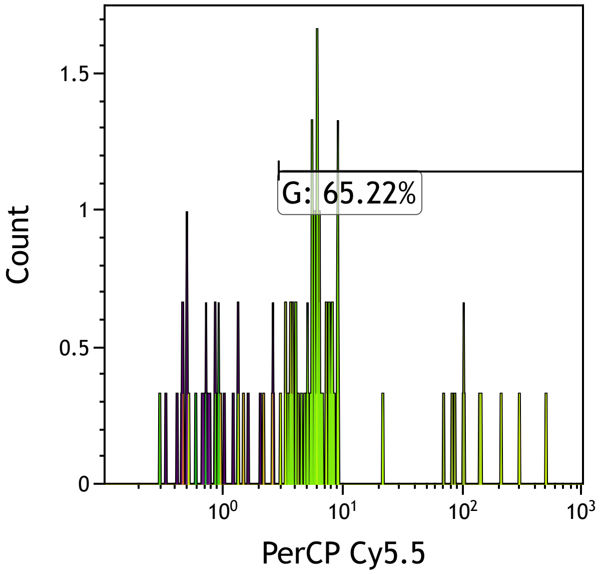

Gate	X-Med
All	2.43
F	5.59

Gate	X-Med
All	5.03
G	6.24

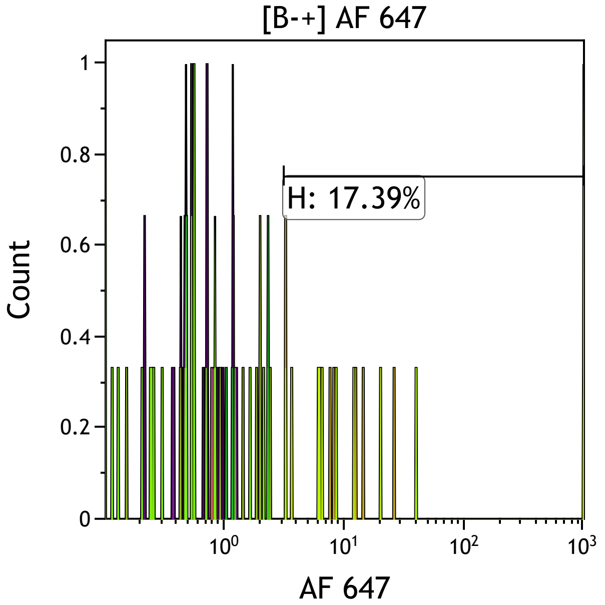

Gate	X-Med
All	0.56
H	8.63

10 contra-2017-03-21-121514-20
Rat microglia M1M2 protocol - Imported

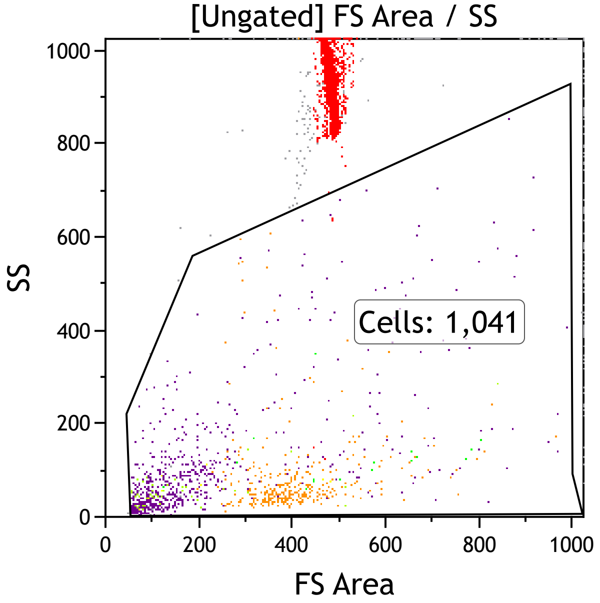

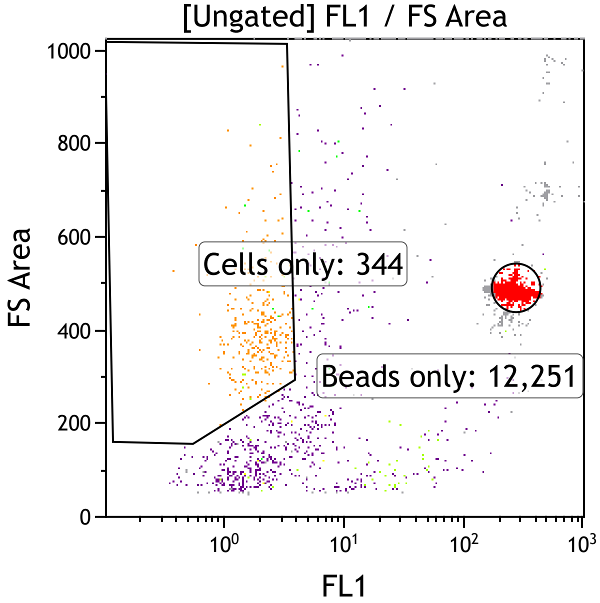

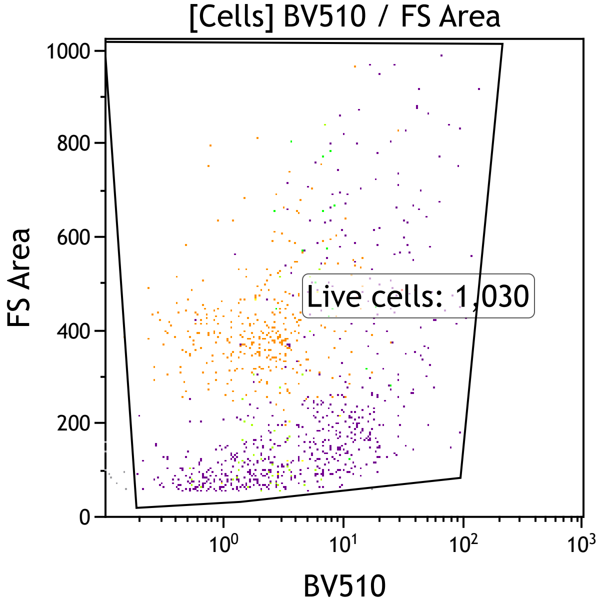

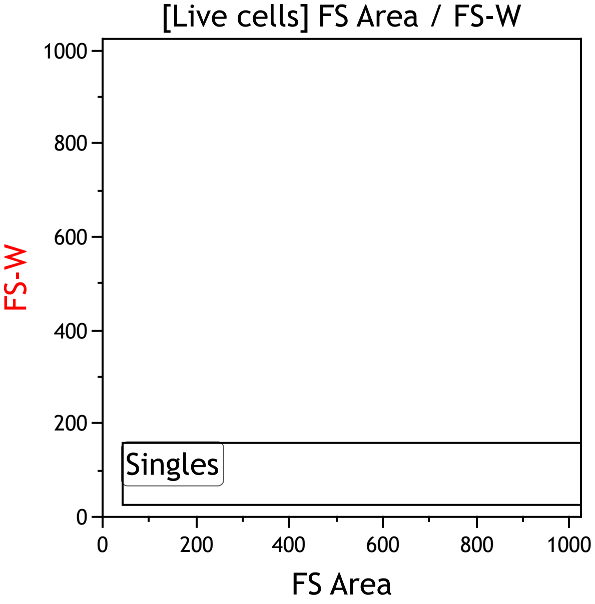

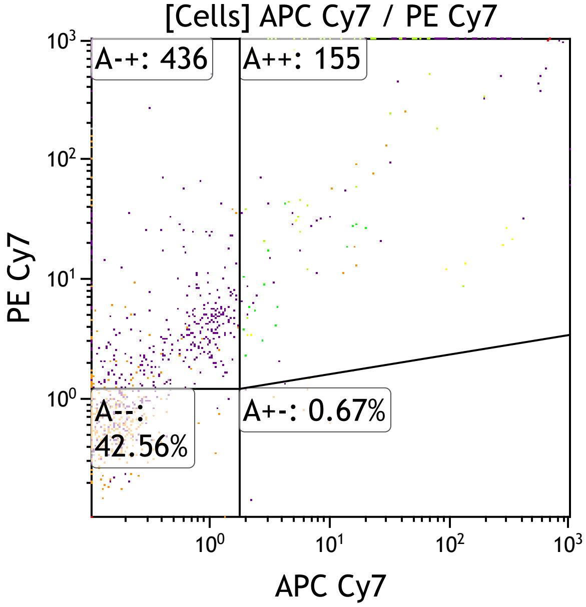

10 contra-2017-03-21-121514-20
Rat microglia M1M2 protocol - Imported

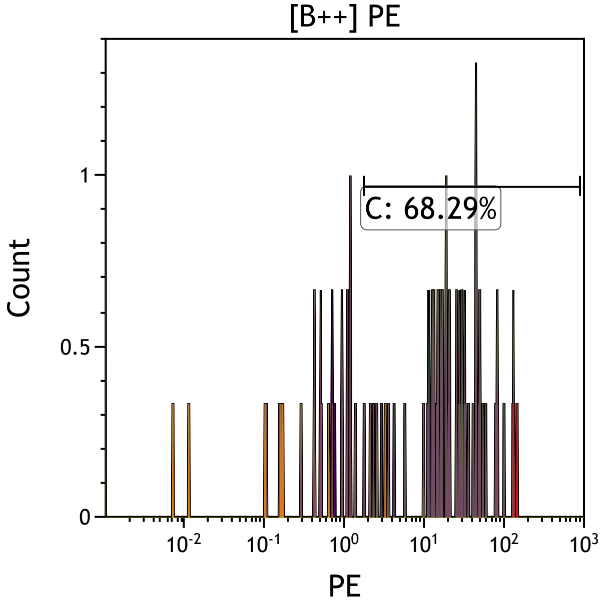

Gate	X-Med
All	13.29
C	20.75

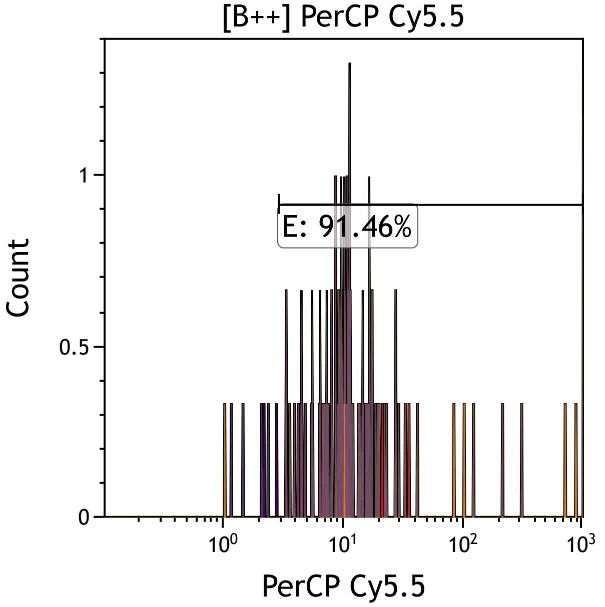

Gate	X-Med
All	11.00
E	11.38

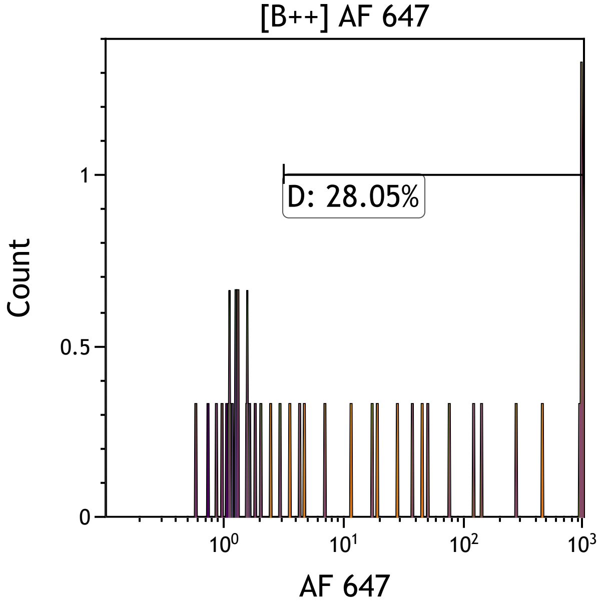

Gate	X-Med
All	0.73
D	76.44

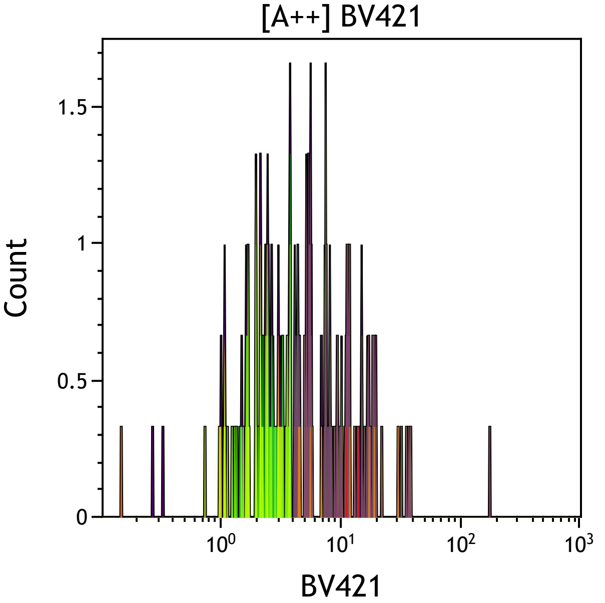

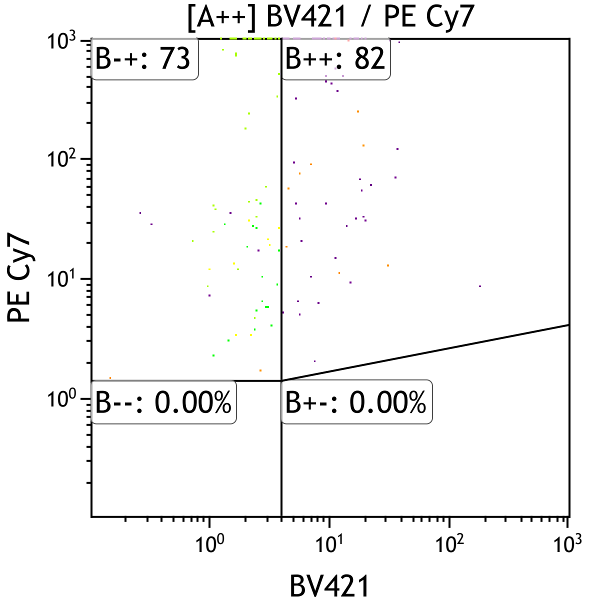

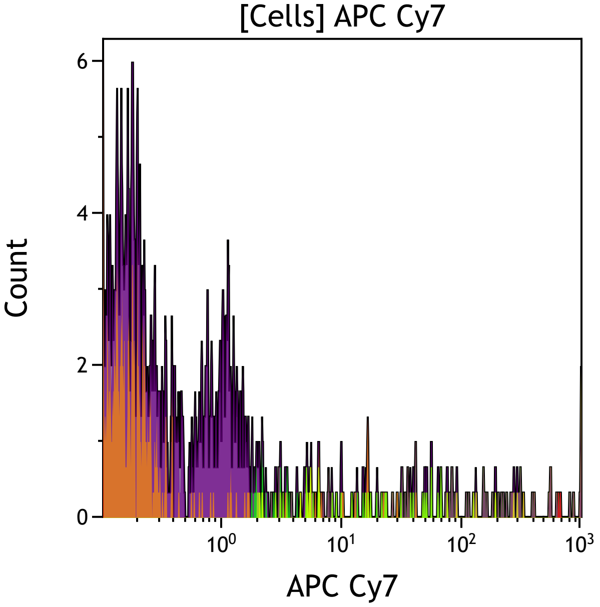

Gate	X-Med	Y-Med
All	4.34	334.17
B--	N/A	N/A
B-+	2.25	38.24
B+-	N/A	N/A
B++	9.19	983.38

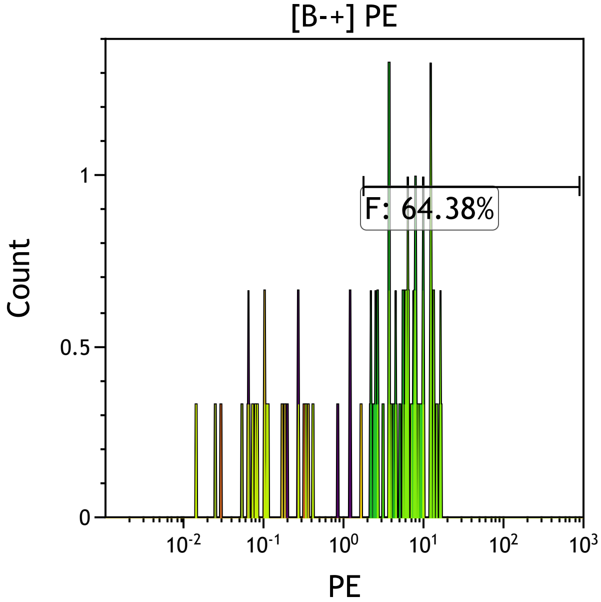

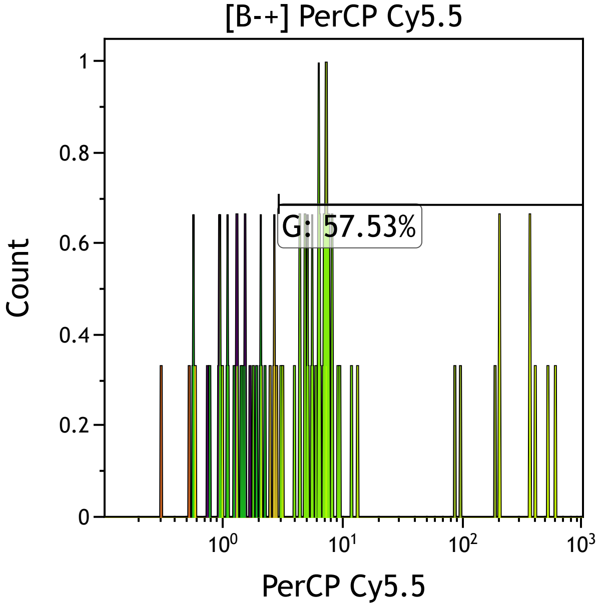

Gate	X-Med
All	3.72
F	6.44

Gate	X-Med
All	4.83
G	7.31

10 contra-2017-03-21-121514-20
Rat microglia M1M2 protocol - Imported

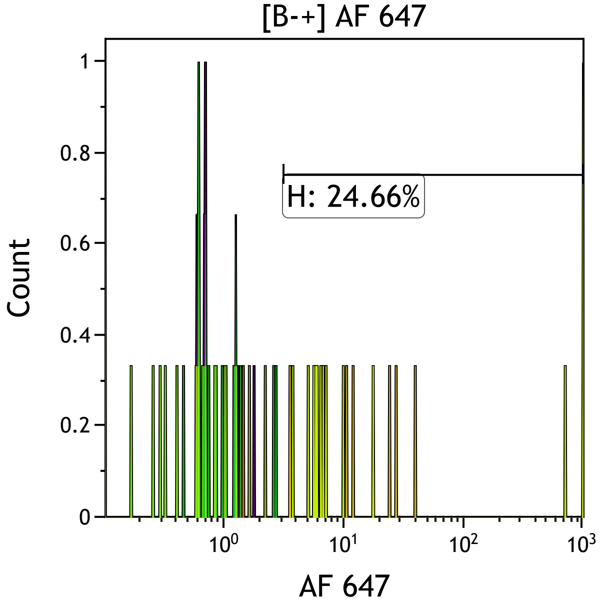

Gate	X-Med
All	0.71
H	9.97
