# Supplementary material for: Human adipose-derived mesenchymal stem cells for acute and sub-acute TBI
Source: PLoS One. 2020 May 26;15(5):e0233263. doi: 10.1371/journal.pone.0233263 (PMC7250455; doi:10.1371/journal.pone.0233263)

## Slide 1
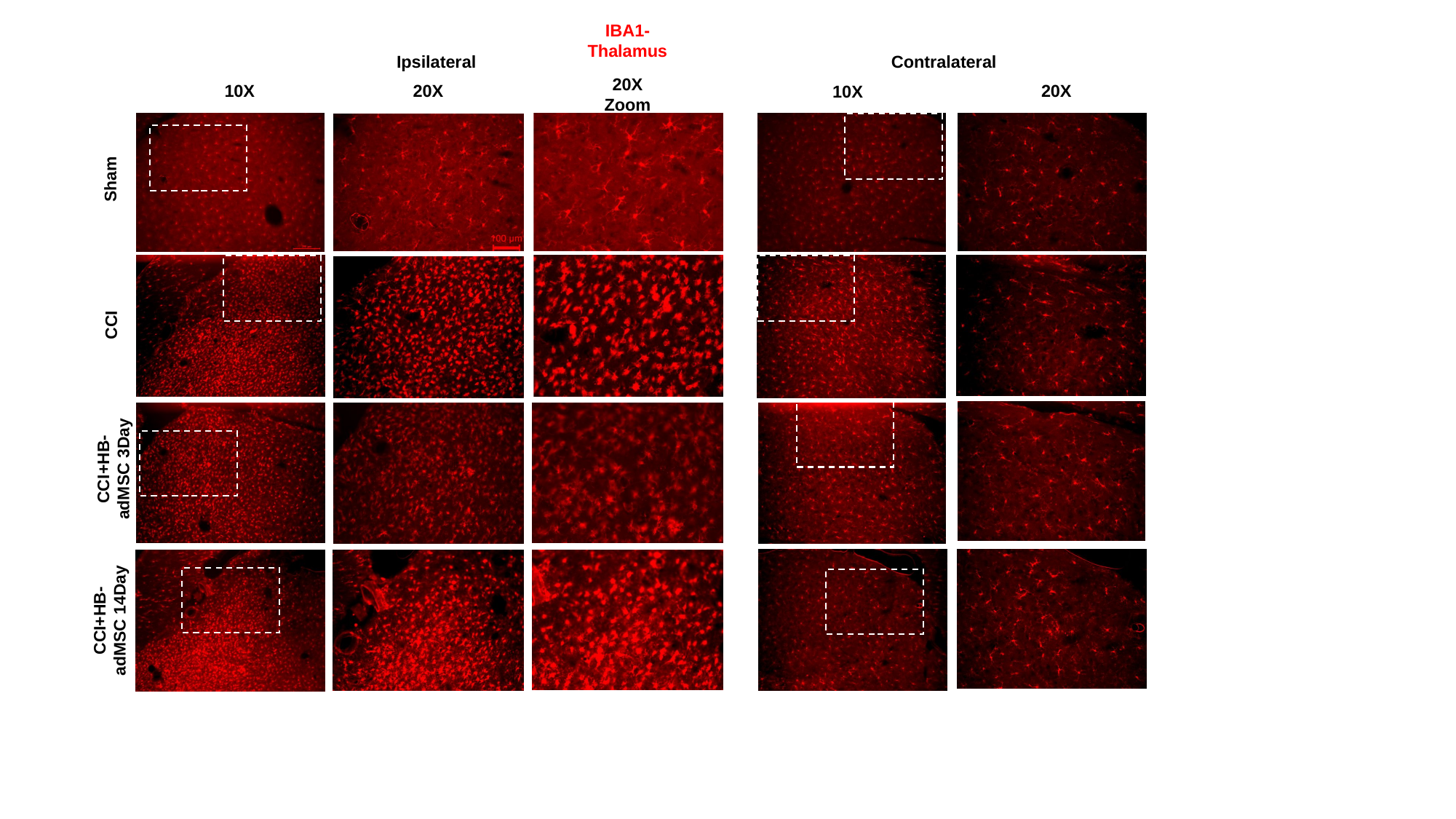

IBA1-Thalamus
Ipsilateral
Contralateral
20X Zoom
20X
20X
10X
10X
Sham
CCI
CCI+HB-adMSC 3Day
CCI+HB-adMSC 14Day

## Slide 2
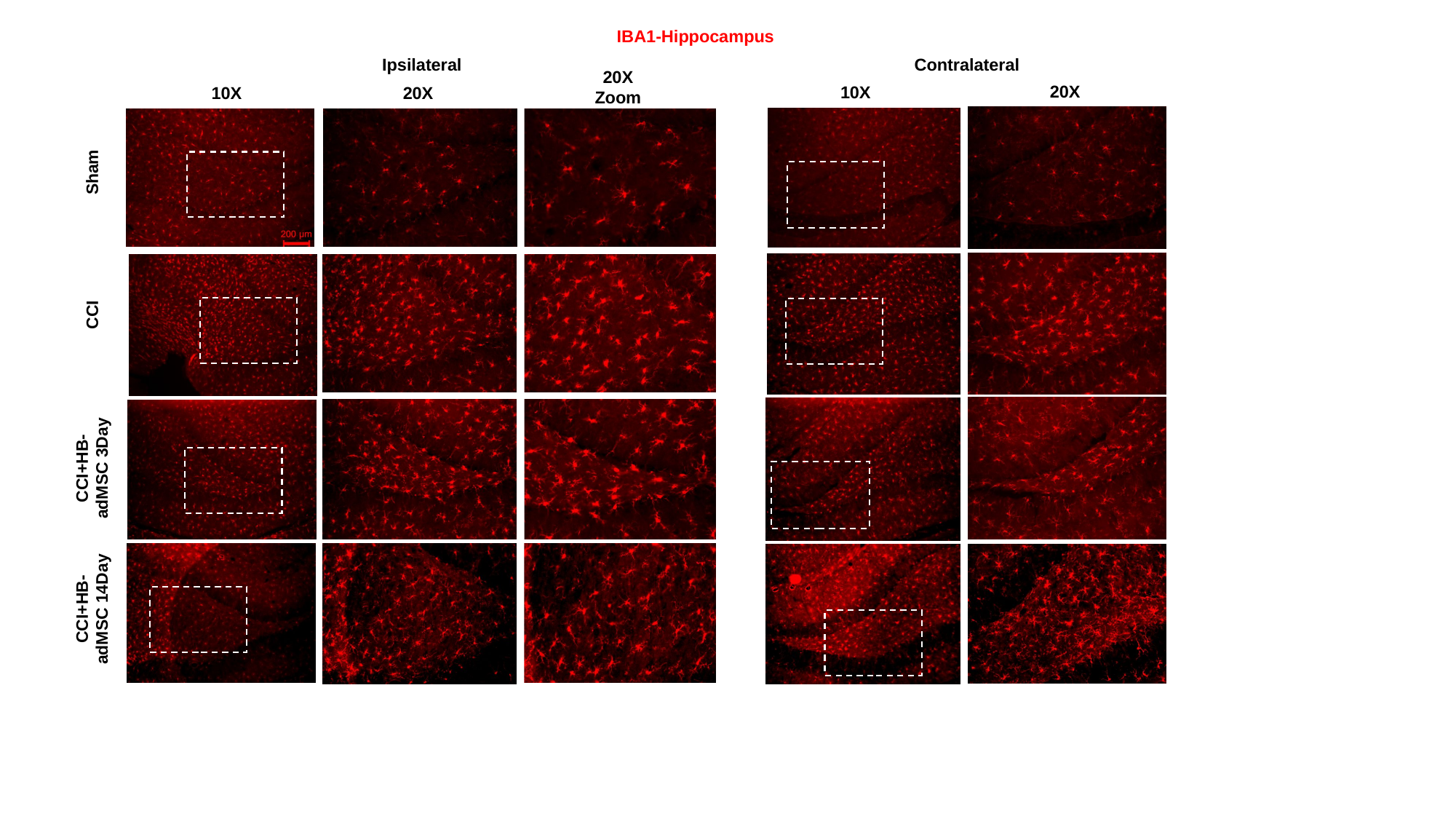

IBA1-Hippocampus
Contralateral
Ipsilateral
20X Zoom
20X
10X
10X
20X
Sham
CCI
CCI+HB-adMSC 3Day
CCI+HB-adMSC 14Day

## Slide 3
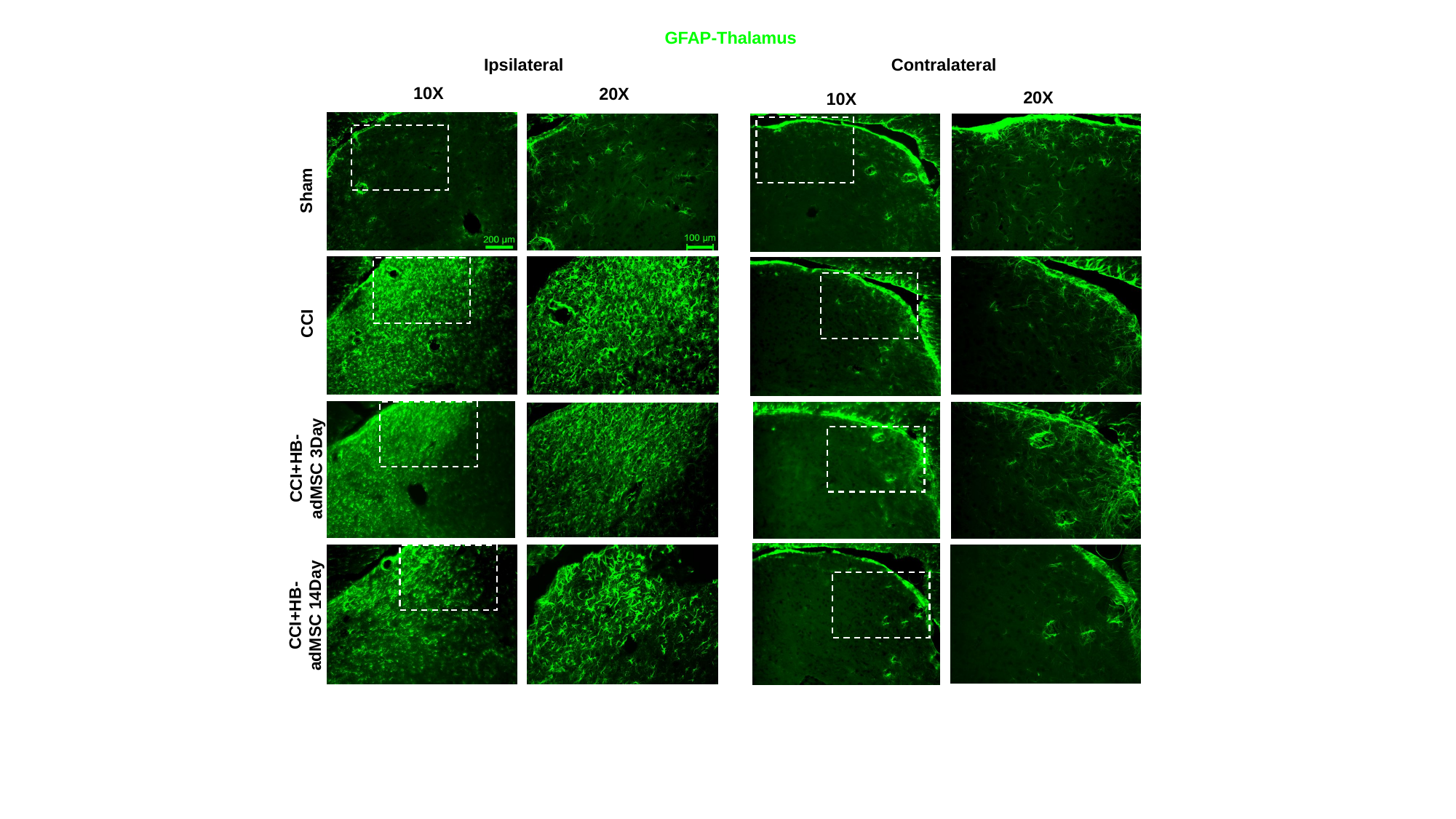

GFAP-Thalamus
Ipsilateral
Contralateral
10X
20X
20X
10X
Sham
CCI
CCI+HB-adMSC 3Day
CCI+HB-adMSC 14Day

## Slide 4
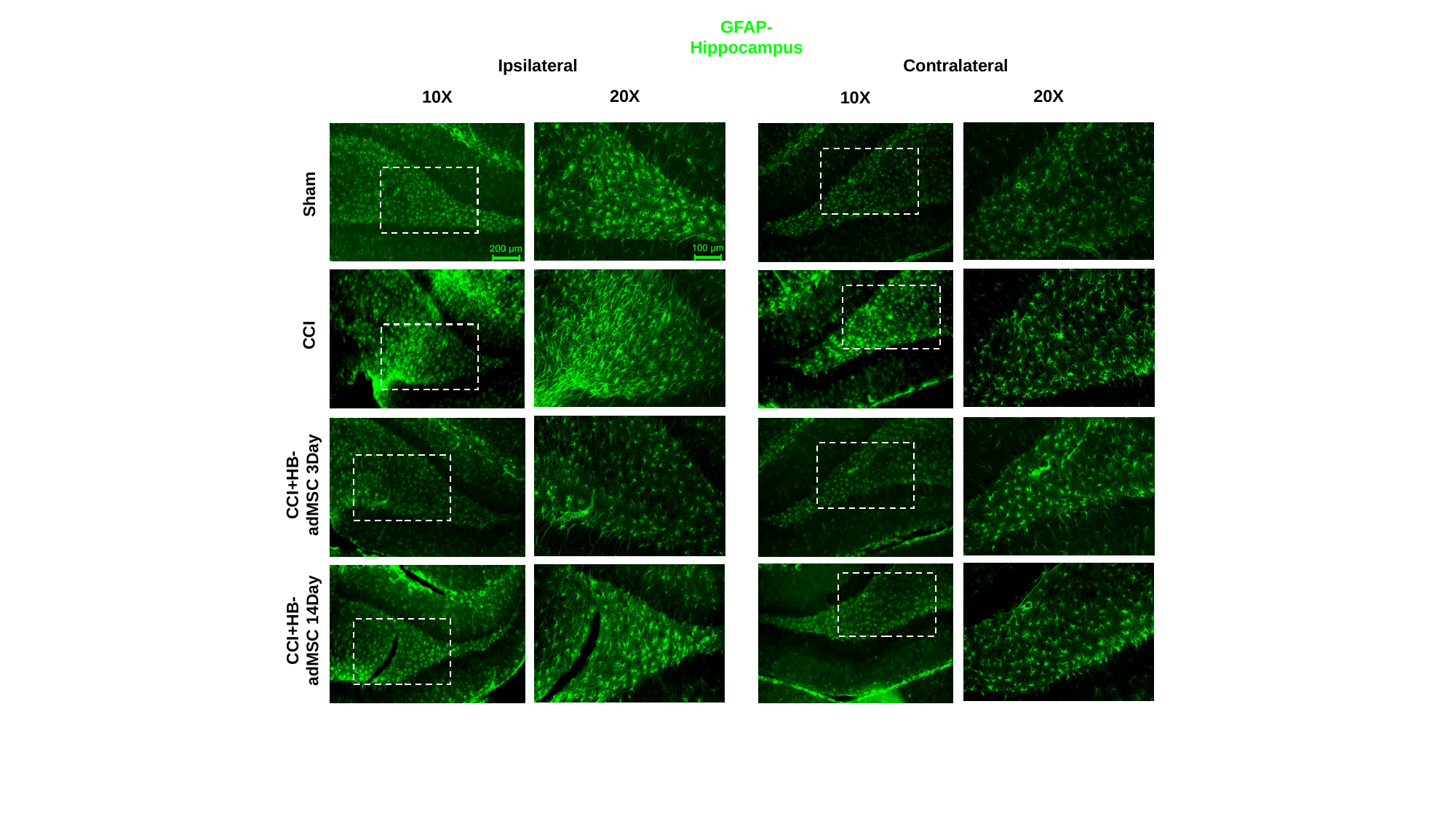

GFAP-Hippocampus
Contralateral
Ipsilateral
20X
20X
10X
10X
Sham
CCI
CCI+HB-adMSC 3Day
CCI+HB-adMSC 14Day

## Slide 5
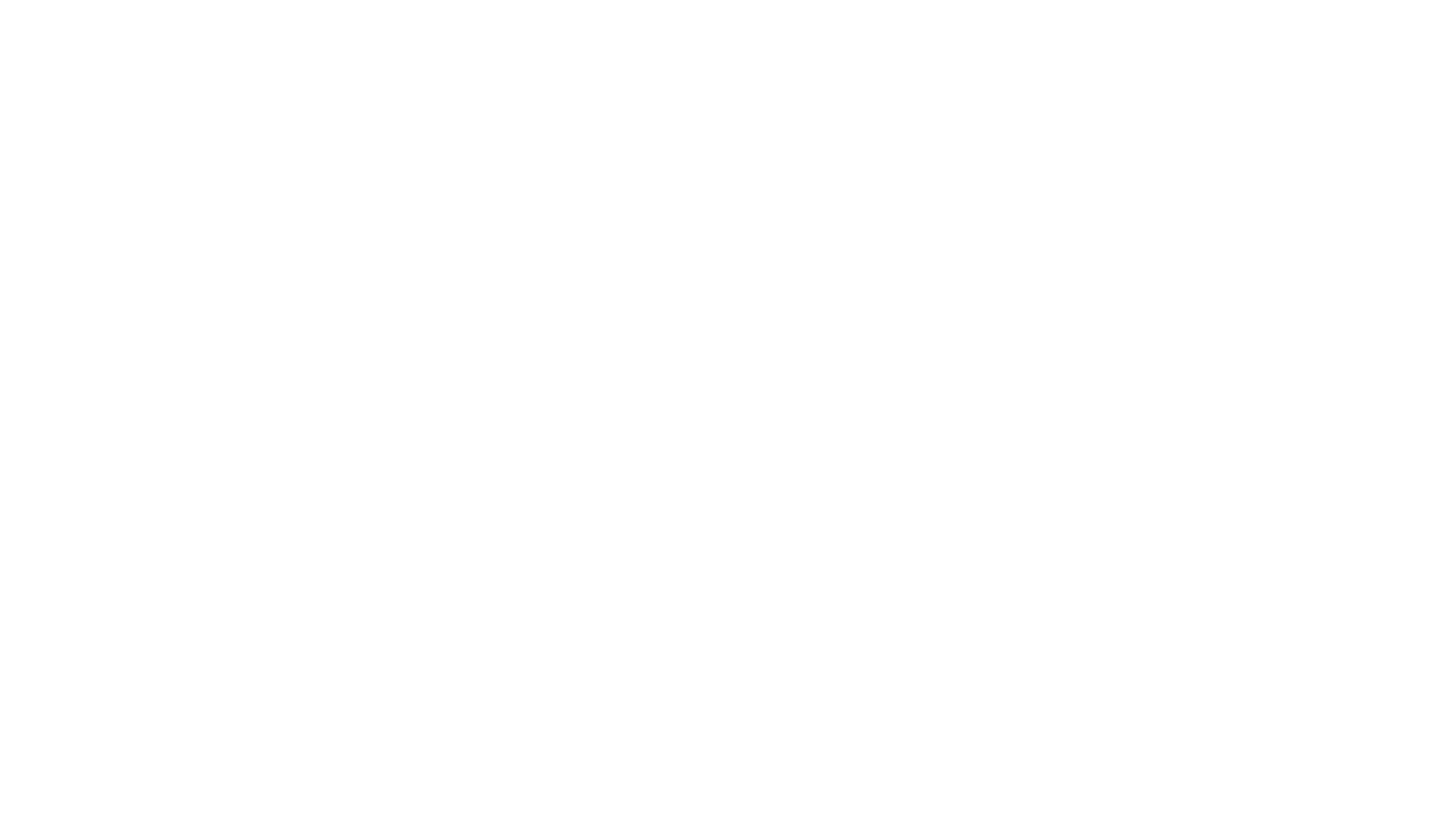

Supplement: S1 File — (ZIP) [file pone.0233263.s006.zip › Data/IHC/IHC figures 1-9-19.pptx]
