# Supplementary figures and images for: Human adipose-derived mesenchymal stem cells for acute and sub-acute TBI
Source: PLoS One. 2020 May 26;15(5):e0233263. doi: 10.1371/journal.pone.0233263 (PMC7250455; doi:10.1371/journal.pone.0233263)

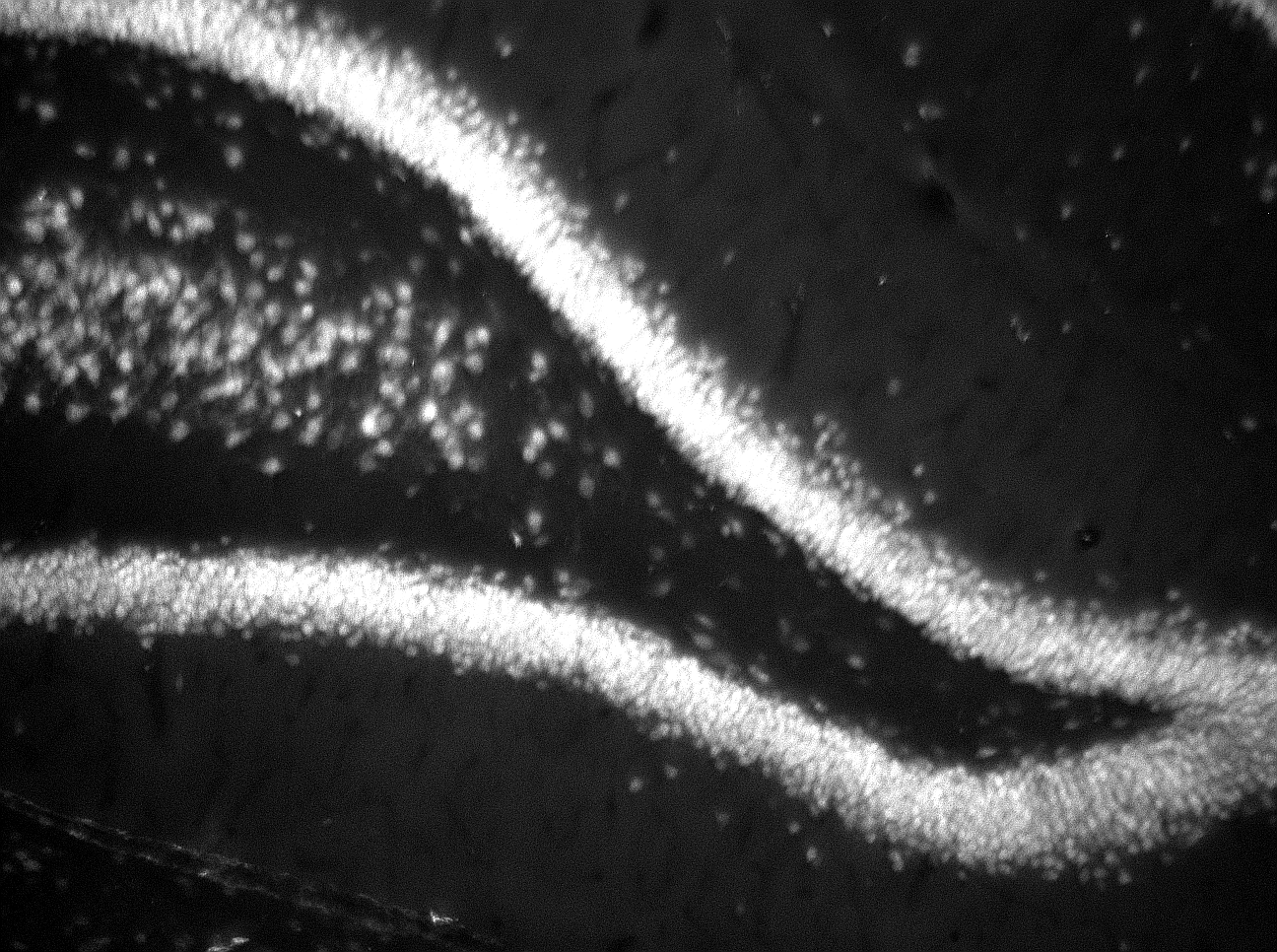

Supplement: S1 File — (ZIP) [file pone.0233263.s006.zip › Data/IHC/S17 2 ipsi 2 10x comp.tif]

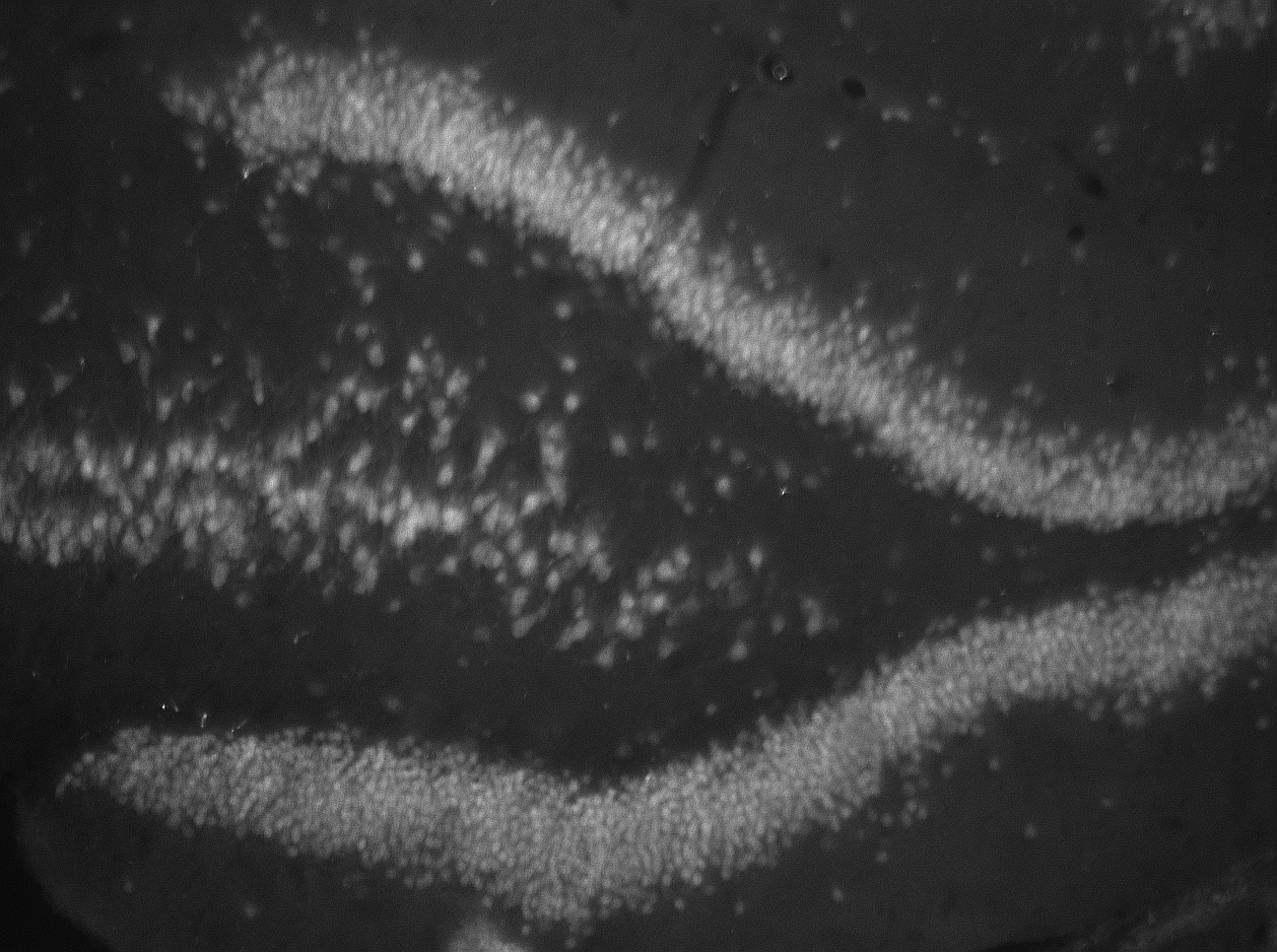

Supplement: S1 File — (ZIP) [file pone.0233263.s006.zip › Data/IHC/T11 2 ipsi 1 comp.tif]

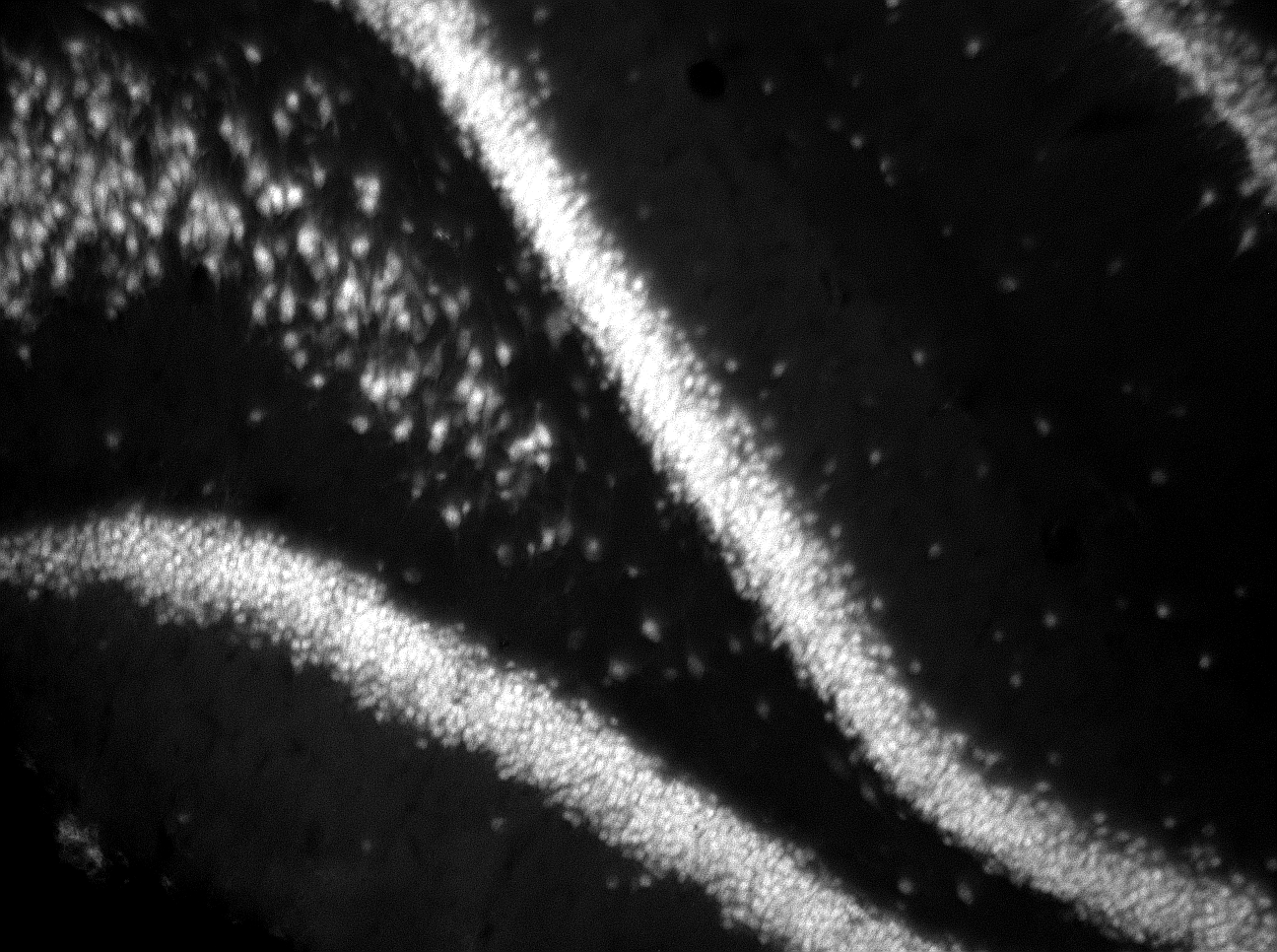

Supplement: S1 File — (ZIP) [file pone.0233263.s006.zip › Data/IHC/T9 1 ipsi 1 10x comp.tif]
